# First high resolution chronostratigraphy for the early North African Acheulean at Casablanca (Morocco)

**DOI:** 10.1038/s41598-021-94695-3

**Published:** 2021-07-28

**Authors:** Rosalia Gallotti, Giovanni Muttoni, David Lefèvre, Jean-Philippe Degeai, Denis Geraads, Andrea Zerboni, Valérie Andrieu-Ponel, Matteo Maron, Serena Perini, Mohssine El Graoui, Séverine Sanz-Laliberté, Camille Daujeard, Paul Fernandes, Mathieu Rué, Lionel Magoga, Abderrahim Mohib, Jean-Paul Raynal

**Affiliations:** 1 Université Paul Valéry Montpellier 3, CNRS, UMR 5140 Archéologie des Sociétés Méditerranéennes, Campus Saint Charles, 34199 Montpellier, France; 2 LabEx Archimède, Université Paul Valéry Montpellier 3, Campus Saint Charles, 34199 Montpellier, France; 3 Université Bordeaux, CNRS, UMR 5199 PACEA-PPP, Bâtiment B18 Allée Geoffroy Saint-Hilaire CS 50023, 33615 Pessac Cedex, France; 4 Dipartimento di Scienze della Terra “Ardito Desio”, Università degli Studi di Milano, Via L. Mangiagalli 34, 20133 Milan, Italy; 5 CR2P-UMR 7207, CNRS, MNHN, Sorbonne Université, CP 38, 8 rue Buffon, 75231 Paris Cedex 05, France; 6 Aix Marseille Université, CNRS, IRD, IMBE - Institut Méditerranéen de Biodiversité et d’Ecologie Marine et Continentale, Technopôle Arbois-Méditerranée, Bât. Villemin, BP 80, 13545 Aix-en-Provence Cedex 04, France; 7 Dipartimento di Ingegneria e Geologia, Università “G. d’Annunzio” di Chieti-Pescara, Via dei Vestini 31, 66100 Chieti, Italy; 8 Institut National des Sciences de l’Archéologie et du Patrimoine, Rabat-Institut, Madinat Al Irfane, Angle rue N°5 et rue N°7, BP 6828, Rabat, Morocco; 9 HNHP-UMR 7194, CNRS, MNHN, UPVD, Institut de Paléontologie Humaine, Sorbonne Université, 1 rue René Panhard, 75013 Paris, France; 10 Paléotime, 75 avenue Jean Séraphin Achard-Picard, 38250 Villard-de-Lans, France; 11 Mission archéologique Littoral-Maroc, 5 Rue Alquié, 03200 Vichy, France; 12 Direction Provinciale de la Culture, Centre d’interprétation du Patrimoine du Gharb, Quartier administratif, Bd Mohamed V, Kenitra, Morocco; 13 Department of Human Evolution, Max Planck Institute for Evolutionary Anthropology, Deutscher Platz 6, 04103 Leipzig, Germany

**Keywords:** Evolution, Archaeology

## Abstract

The onset of the Acheulean, marked by the emergence of large cutting tools (LCTs), is considered a major technological advance in the Early Stone Age and a key turning point in human evolution. The Acheulean originated in East Africa at ~ 1.8–1.6 Ma and is reported in South Africa between ~ 1.6 and > 1.0 Ma. The timing of its appearance and development in North Africa have been poorly known due to the near-absence of well-dated sites in reliable contexts. The ~ 1 Ma stone artefacts of Tighennif (Algeria) and Thomas Quarry I-Unit L (ThI-L) at Casablanca (Morocco) are thus far regarded as documenting the oldest Acheulean in North Africa but whatever the precision of their stratigraphical position, both deserve a better chronology. Here we provide a chronology for ThI**-**L, based on new magnetostratigraphic and geochemical data. Added to the existing lithostratigraphy of the Casablanca sequence, these results provide the first robust chronostratigraphic framework for the early North African Acheulean and firmly establish its emergence in this part of the continent back at least to ~ 1.3 Ma.

## Introduction

**Figure 1:**
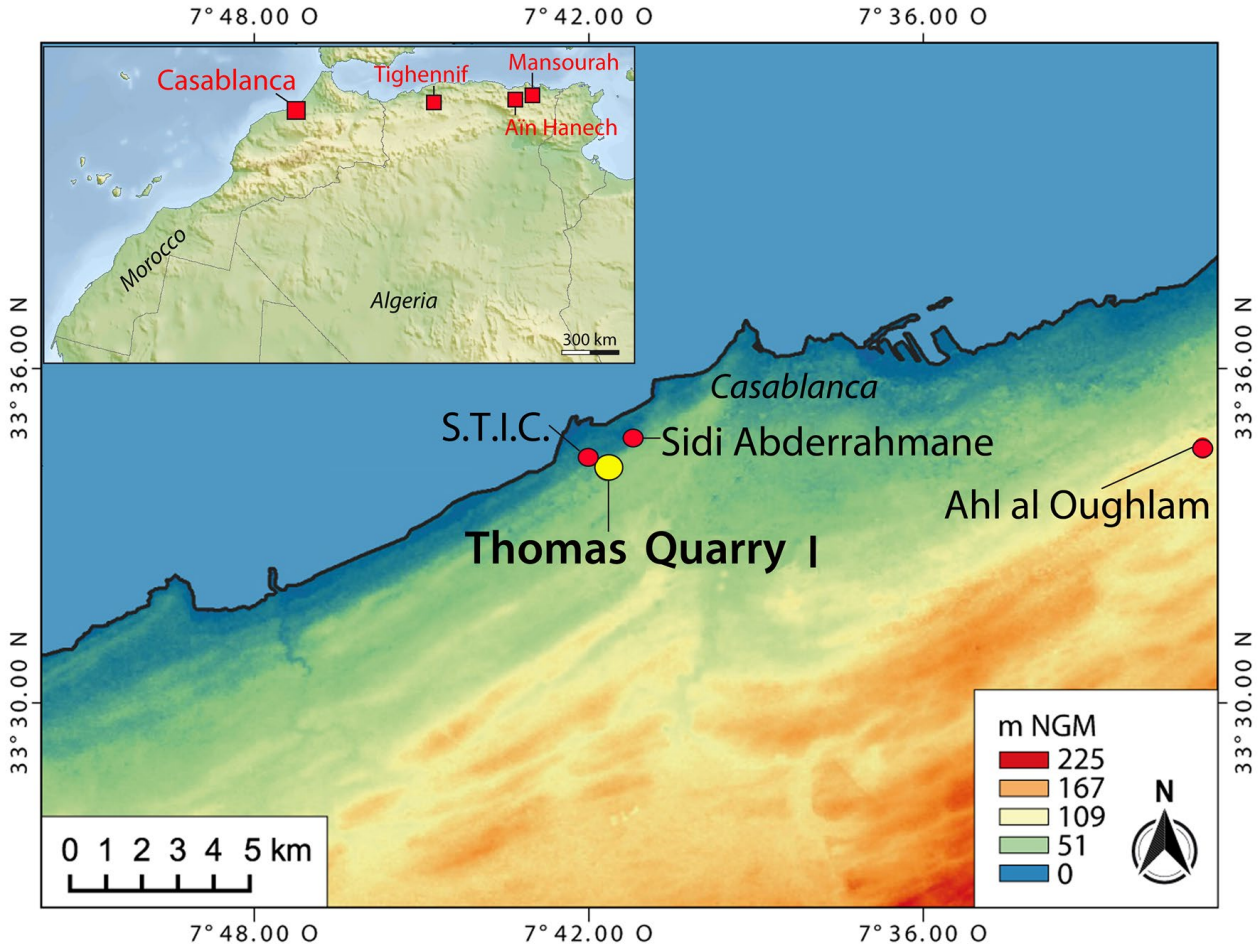
Location of Thomas Quarry I and all the archaeological sites cited in the text. North-Africa map: modified by R. Gallotti after NASA/JPL/NIMA, public domain, via Wikimedia Commons. Casablanca map: modified by M. Rué and R. Gallotti after NASA/SRTM, 1 arc second global elevation data, created using the free and open source software QGIS v3.18.2 (http://www.qgis.org).

Over the last two decades, several efforts have been made to refine the chronostratigraphic framework of the Acheulean emergence in Africa. In East Africa, well-dated records place the emergence of the Acheulean at ~ 1.8–1.6 Ma^1–5^. The earliest Acheulean in South Africa seems to be nearly as old as in some East African sites and is reported to fall between ~ 1.6 and > 1.0 Ma^6^. North Africa is also rich in Acheulean sites, but most of them are within units of unknown stratigraphic position^7^. As regards the Early Pleistocene, the only North African Acheulean sites in known stratigraphic context are Tighennif near Mascara in Algeria and Thomas Quarry I (ThI) in Morocco^7,8^ (Fig. 1).

**Figure 2:**
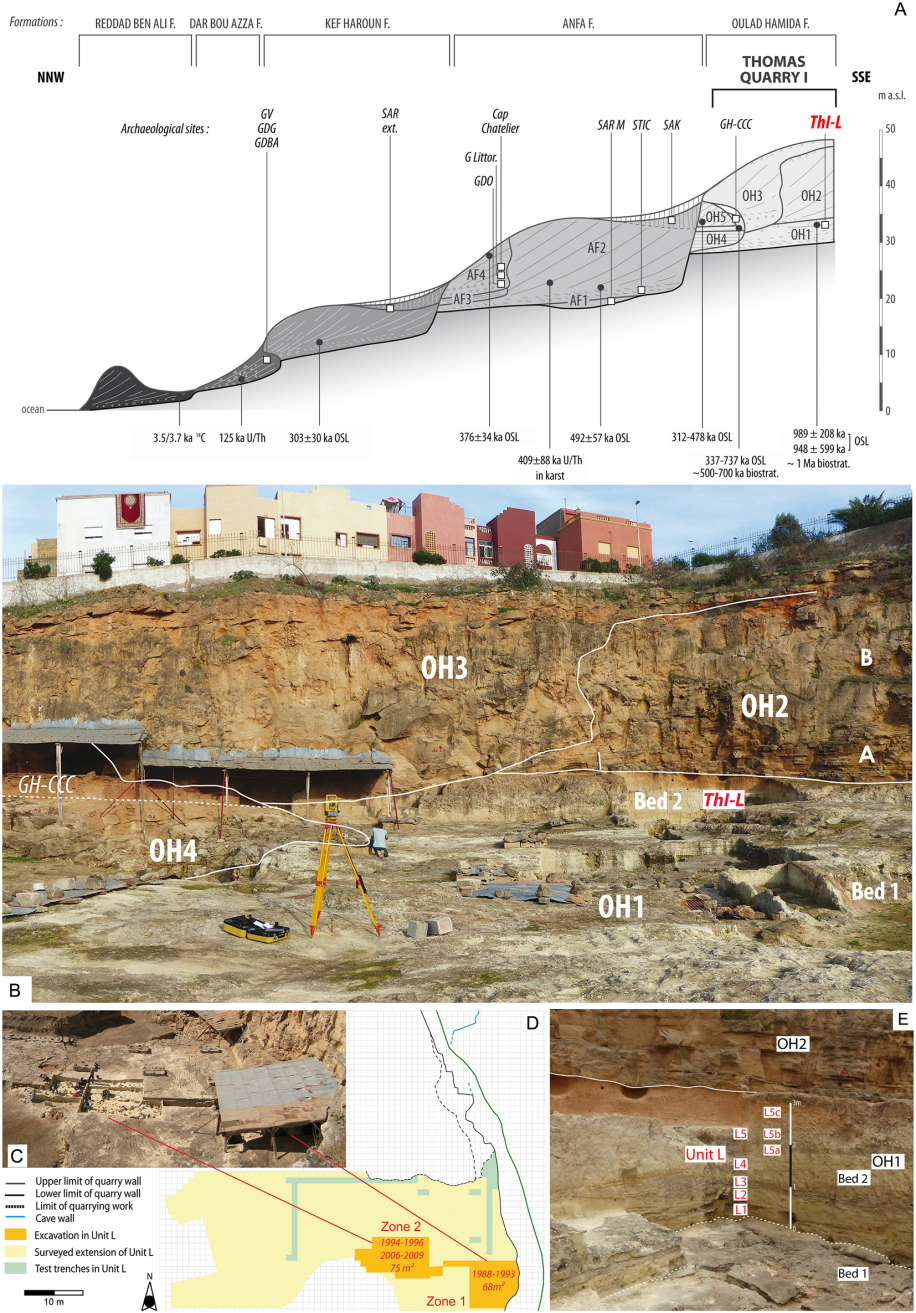
(**A**) OHF and its Members (late Early Pleistocene-Middle Pleistocene) exposed at Thomas and Sidi Abderrahmane quarries, South-West of Casablanca (drawing D. Lefèvre). Archaeological sites: GDBA: *Grotte de* Dar Bou Azza; GDG: *Grotte des Gazelles*; GDO: *Grotte des Ours;* GH-CCC: *Grotte à Hominidés—*complex of continental deposits; G Littor.: *Grotte des Littorines;* GV: *Grotte Velozzo*; SAR ext.: *Sidi Abderrahmane—Extension*; SAK: *Sidi Al Khadir*; SAR M: *Sidi Abderrahmane—*Unit M; S.T.I.C.: *Société de Transformation Industrielle et de Construction* Quarry; ThI-L: Thomas Quarry I—Unit L. OSL and ESR ages, and biochronological data. (**B**) Members 1 to 4 of the OHF at ThI (photo and drawing D. Lefèvre); (**C**) ThI-L1 during 2007 excavation (photo J.-P. Raynal); (**D**) map of ThI with extension of unit L and location of the excavation zones (drawing R. Gallotti); E: ThI-L, Zone 1: OH1 Bed 2—Unit L—L1 to L5 deposits. Photo and drawing D. Lefèvre. Figures were generated with Adobe Photoshop CC version 2015-1, 20151114.r301 X64. Copyright 1990 2015 Adobe Systems Incorporated and its licensors. All rights reserved.

Thomas Quarry I is in the south-western suburbs of Casablanca (Fig. 1) and exposes an Early–Middle Pleistocene stratigraphic sequence. In this area, four formations including several members are associated with four raised marine platforms that can be assigned to the late Early to the Late Pleistocene. They revealed a rich complex of archaeological sites (Fig. 2A): the *Oulad Hamida Formation* (OHF, late Early Pleistocene-early Middle Pleistocene), the *Anfa* (AF) and *Kef Haroun Formations* (Middle Pleistocene), and the *Dar Bou Azza Form*ation (Late Pleistocene)^9,10^.

Thomas Quarry I became famous worldwide thanks to the discovery in 1969 of a Middle Pleistocene human fossil^7^. Further research yielded more human remains and a rich archaeological complex at *Grotte à Hominidés* (GH), a cave preserved in the north-eastern wall of the same quarry^7^. In 1985, research in the lower deposits of the quarry revealed the presence of an early Acheulean industry with a clear stratigraphic context (Unit L; ThI-L)^11^. Lithostratigraphy, biostratigraphy, and OSL ages placed ThI-L at approximately 1 Ma, an age similar to that of Tighennif, however, the chronology of these two sites remained poorly defined^8,12,13^ as was the age of emergence of the Acheulean and its subsequent development in this part of the African continent.

Here we present new chronostratigraphic data based on magnetostratigraphy and geochemistry for ThI-L, which constrain its age and provide a robust high-resolution framework for the earliest Acheulean in North Africa, pushing the time of its first appearance in this part of the continent back at least to ~ 1.3 Ma.

## ThI-L context

The Thomas Quarry I Pleistocene deposits belong to OHF that unconformably overlies the Cretaceous and Cambro-Ordovician basement with the contact placed at a current altitude of ~ 28 m above sea level (asl) (Fig. 2A**)**. From bottom to top, OHF includes four allostratigraphic units—OH1 to OH4 Members—each having unconformities at their bases and tops (Fig. 2A,B, see Supplementary Information text and Supplementary Fig. S1)^9,14,15^. OH1-Bed 1, OH2, OH3, and OH4 are mainly composed of calcirudites and/or coquinoid and coarse-grained biocalcarenites overlying abrasion platforms associated either with a cliff palaeoshoreline (OH3) or a deep cavity (OH4) and pass vertically from coarse to medium-grained biocalcarenites^7,9^. These sedimentary sequences of genetically related intertidal, supratidal and aeolian depositional environments record sea-level high-stands associated with shoreline formation. The unconformities or sequence boundaries mark the shoreline regression due to sea-level fall. Thus, OH1 to OH4 correlate with sea-level high-stands of the main global glacio-eustatic cycles as inferred from the marine isotope stage (MIS) record and are preserved at positive elevations due to the regional tectonic uplift affecting Atlantic Morocco^14,16^. OHF predates the Anfa Formation (Fig. 2A) whose Members AF1 and AF2 are correlated to MIS 13 and MIS 15^9,13,14^. In the GH cave, OH4 deposits are covered by a complex of continental deposits (GH-CCC) subdivided into two parts separated by an aeolianite (OH5)^7,9^. As GH-CCC/unit 4 overlying OH4 has been dated to 0.5–0.6 Ma^13,15^ in accordance with biostratigraphy^17^, the OH4 marine high-stand associated with the palaeoshoreline is undoubtedly much older than MIS 15. Thus, OH4 to OH1 Members should record sea-level high-stands from at least MIS 17 and older.

Thomas Quarry I-Unit L belongs to OH1-Bed 2 whose deposits form a 2 to 3 m-thick succession of locally cross-bedded coarse siliciclastic to bioclastic sands and calcareous mudstones—L1 to L5. In the uppermost part of the sequence, evidence of pedogenesis during warm episodes of subaerial exposure are preserved^10,14^ (Fig. 2E, see Supplementary Information Text, Supplementary Table S1, Supplementary Figs S2–S5). The formation of the sequence is probably related to the interplay of littoral processes that include sedimentation in low energy fluvial channels interchanged to swamps along backshore environments, alternating with periods of enhanced aeolian sedimentation. ThI-L was dated using the OSL signal of quartz grains to 989 ± 208 ka and 948 ± 599 ka^13^, with large uncertainties.

Two archaeo-stratigraphic sub-units have been recorded at ThI-L, L1 at the base and L5 at the top. About 1000 m^2^ of ThI-L1 has been surveyed and systematically test-pitted, and it was excavated in 1988–1996 and 2006–2008 in two main areas of 68 m^2^ (zone 1) and 75 m^2^ (zone 2), respectively (Fig. 2C,D). ThI-L5 has been excavated only in zone 1. Both sub-units yielded a rich corpus of lithic artefacts together with faunal remains^7^.

The fauna from ThI-L is poor and fragmentary, and the bone surfaces are badly preserved (see Supplementary Information Text, Supplementary Figs S6, S7). Large mammals are dominated by hippos (*Hippopotamus sirensis*, akin to *H.* *gorgops*). Bovids include a bovin, a gazelle, a medium-sized alcelaphin, and a medium-sized *Kobus* sp. An *Equus* and a rhino are also represented, together with an elephant^17^. A tooth of the suid *Kolpochoerus* is reminiscent of *K.* *majus* from East Africa. Among rodents, the most significant differences from GH-CCC/unit 4 are the absence of the mole vole *Ellobius* and the presence of distinct species of *Paraethomys* and *Gerbillus* s.l. Overall, the mammalian fauna from ThI-L is too poor to be confidently dated by biochronology, but it is significantly older than GH-CCC/unit 4 and Tighennif (see Supplementary Information text).

## ThI-L technical behaviours

The artefact assemblages of ThI-L1 (n = 3845) and ThI-L5 (n = 1228) are composed of Paleozoic tenacious rocks, mainly quartzites abundantly available in local primary and secondary sources, and flint derived from the phosphatic plateau in the hinterland of the Meseta and available in secondary deposits near the site^7^.

Two main quartzite production systems coexisted in both levels^7^: one is focused on the production of small to medium-sized flakes, the other is devoted to the manufacture of Large Cutting Tools (LCTs).

**Figure 3:**
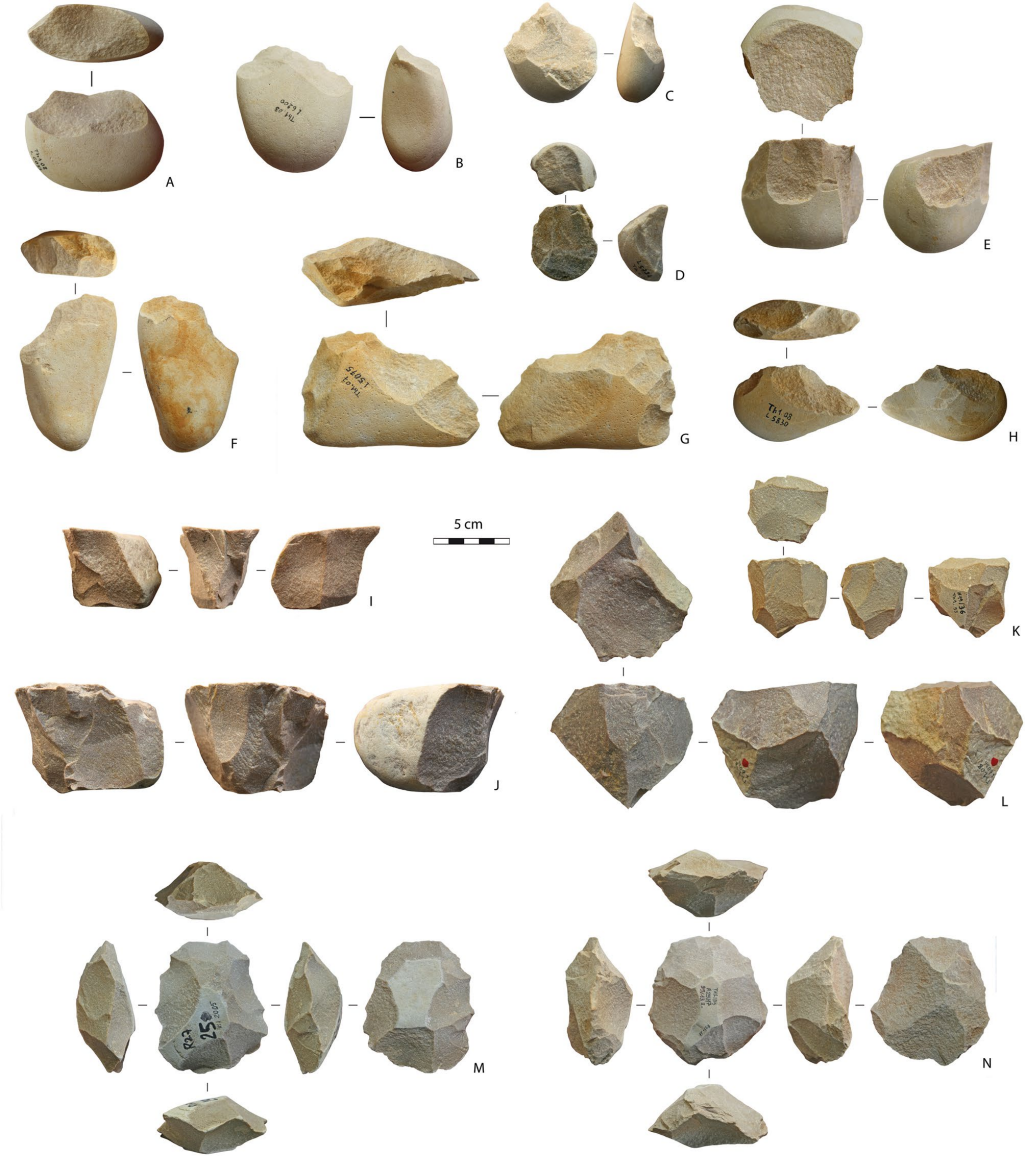
Quartzite cores. (**A–E**) unifacial unidirectional cores; (**F–H**) bifacial partial cores; (**I,J**) peripheral unidirectional cores; (**K,L**) multifacial multidirectional cores; (**M,N**) discoid cores. This figure was generated on the basis of photos made by R. Gallotti with Adobe Photoshop CC version 2015-1, 20151114.r301 X64. Copyright 1990 2015 Adobe Systems Incorporated and its licensors. All rights reserved.

Small-medium sized flakes of quartzite were produced by a diversity of methods (unifacial unidirectional, bifacial partial, peripheral unidirectional, multifacial multidirectional, and discoid) and were not retouched (Fig. 3). All cores (n = 605) are on cobbles and devoted to small flaking. Cores for the extraction of large flake with a length or width > 10 cm are lacking in the excavated area.

**Figure 4:**
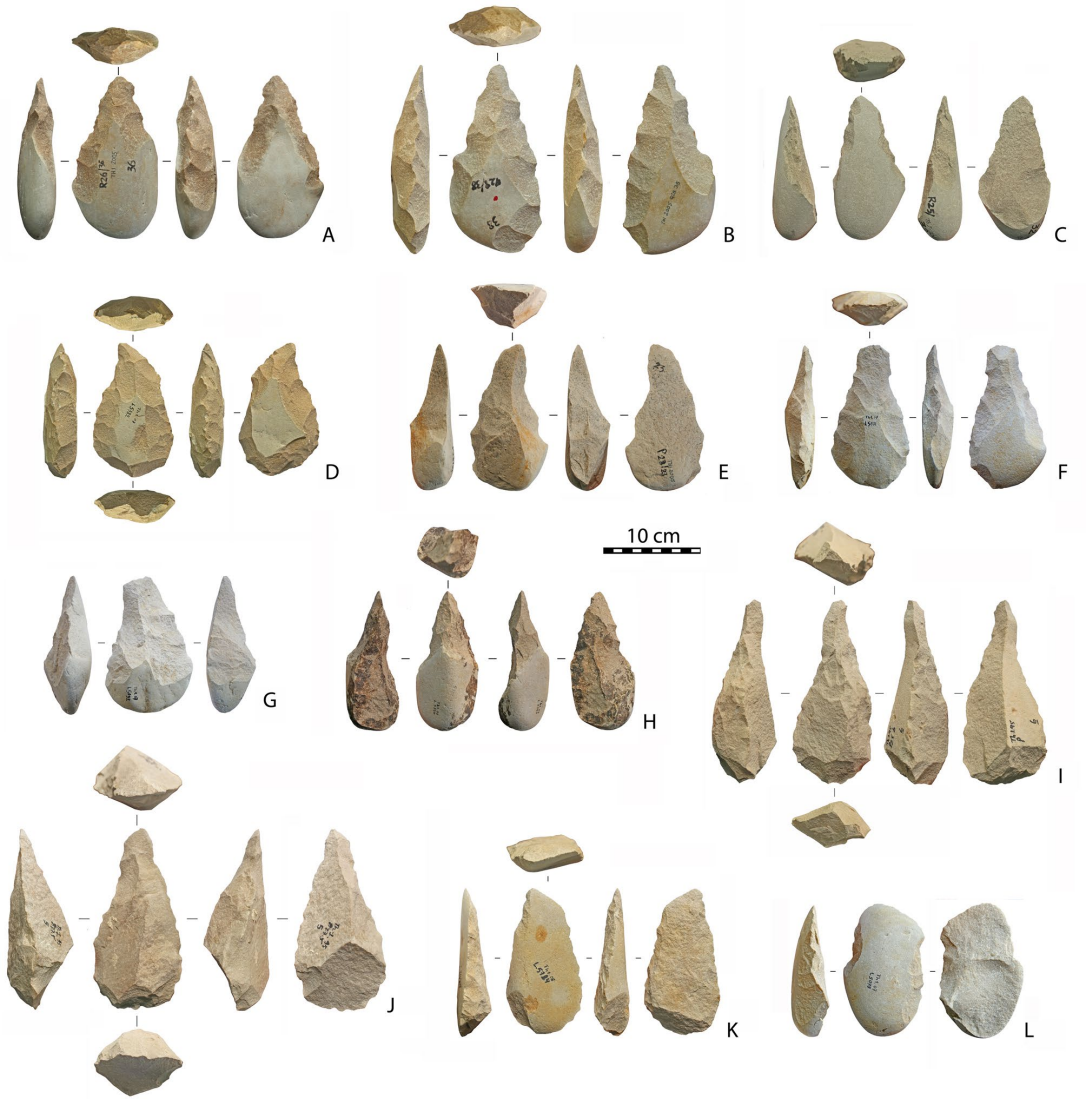
LCTs. (**A,B**) pointed handaxes on cobble; (**C**) pointed handaxe on *entame* flake; (**D,E**) pointed handaxes on cobble with a skewed point; (**F,G**) beveled handaxes; (**H**) pick on cobble; (**I,J**) picks on core edge flake from block; (**K,L**) cleavers on *entame* flake. This figure was generated on the basis of photos made by R. Gallotti with Adobe Photoshop CC version 2015-1, 20151114.r301 X64. Copyright 1990 2015 Adobe Systems Incorporated and its licensors. All rights reserved.

Large Cutting Tools (n = 230) were mainly produced from large cobbles, less often from large flakes (22%) detached mainly using the *entame* core method^18^*.* At ThI-L, this method has been used to produce large flake blanks for the 12 cleavers^19^ identified (Fig. 4K,L) and for eight pointed handaxes (Fig. 4C). Several shaping processes are repeated on several specimens that all tend towards similar morphologies and producing several types of LCTs, the most common of which are pointed handaxes (n = 107; Fig. 4A–C). Shaping proceeds in two steps: the location of the apical portion followed by one to three series of removals that creates lateral edge convergence without further trimming. Variations of these schemes produce pointed handaxes with a skewed point (n = 18) shaped by notches that penetrate one or both edges (Fig. 4D,E) as well as various beveled handaxes (n = 16) with a transversal edge manufactured using marginal retouch (Fig. 4F, G). Other common LCTs are picks on cobbles (n = 38), mostly with a trihedral section at the tip portion, and some on flakes (n = 8; Fig. 4H). In some large specimens (n = 5) the trihedral section includes the whole object and is due to the use of a large edge flake from a block as a core (Fig. 4I,J). The remaining LCTs have broken apical parts (n = 18) or are massive scrapers on *entame* flakes (n = 7). Only one massive scraper on a Kombewa flake is present in the assemblage, but it is insufficient to document a true Kombewa strategy.

Flint production (15% of the artefacts assemblage) is entirely devoted to the extraction of small flakes, adopting some of the flaking methods documented for tenacious rocks (unifacial unidirectional, bifacial partial, multifacial multidirectional) and mainly the free-hand technique. However, flint production at ThI-L1 shows evidence of one specific technical process, that of bipolar-on-anvil semi-peripheral exploitation to produce bladelet-like flakes. This technical process was hitherto unknown for periods in question and demonstrates the diversification and the flexibility within the African Acheulean^20^.

## Magnetochronology

**Figure 5:**
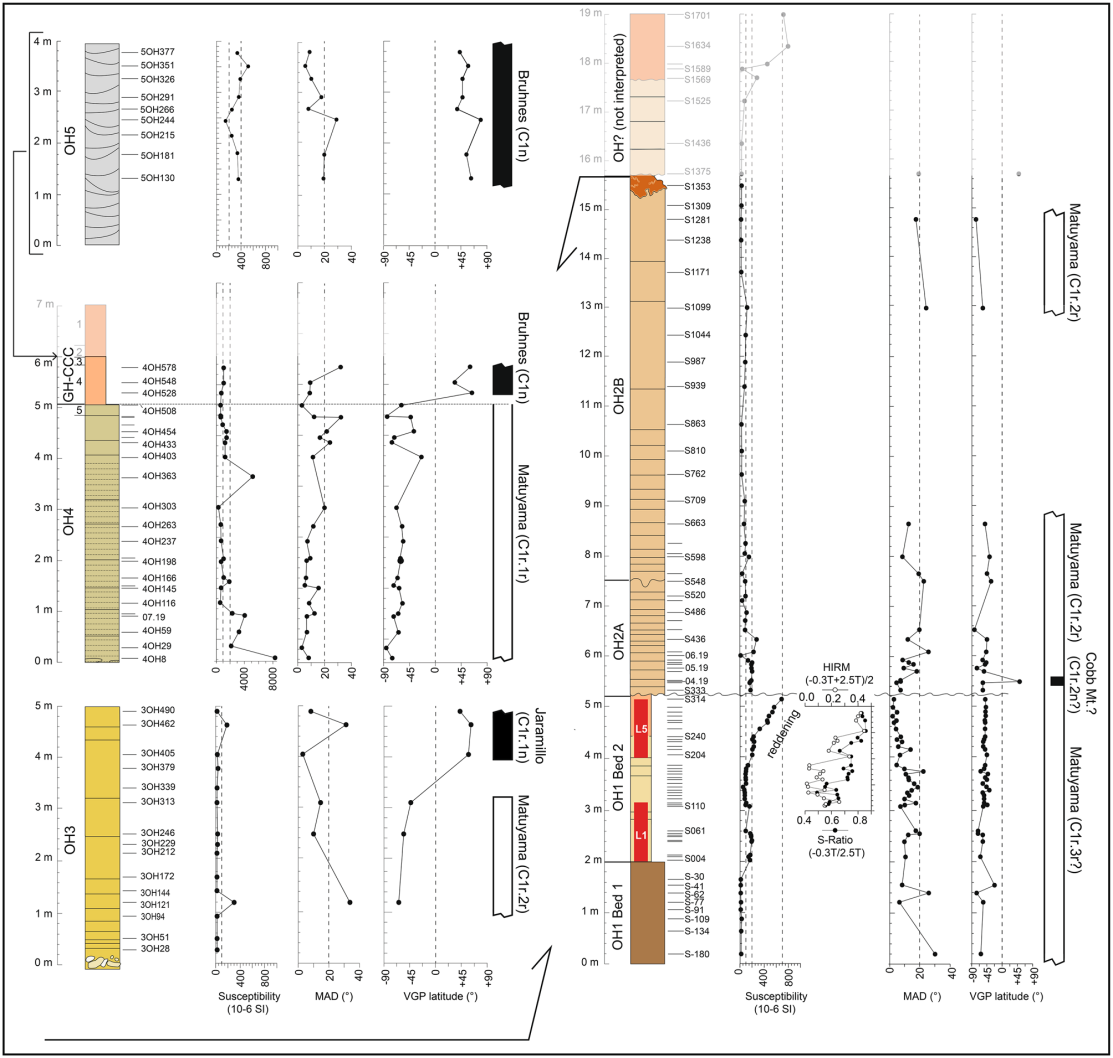
Generalized logs of the stratigraphic sections investigated (youngest at top left, oldest at bottom right, but see description for details) with indication of paleomagnetic samples placed aside initial magnetic susceptibility, maximum angular deviation (MAD) values of the C component directions, virtual geomagnetic pole (VGP) latitudes, and polarity stratigraphy where black bars represent normal polarity and the white bar is reverse polarity. Cobb Mt.? is a possible record of the Cobb Mountain subchron. Red bars indicate archeological units ThI-L1 (bottom) and ThI-L5 (top) within OH1 Bed 2. Drawing G. Muttoni, S. Perini, and S. Sanz-Laliberté. This figure was created with Adobe Illustrator CS6 version (http://www.adobe.com).

Laboratory experiments have been conducted on a total of 179 oriented standard (10 cc) core samples (plus an additional suite of 20 rock chip samples) taken along stratigraphic profiles of OHF straddling OH1 Bed 1, OH1 Bed 2, OH2A and OH2B, OH3, OH4 plus GH-CCC, and OH5. We also sampled Member 2 of the Anfa Formation (AF2) at the Sidi Abderrahmane Quarry. Samples have been taken in order to establish the fundamental rock-magnetic properties of the sediments and unravel the structure and orientation of the natural remanent magnetization therein contained for magnetic polarity determination and correlation to the standard geomagnetic polarity time scale (see “Materials and methods” section, Fig. 5, Supplementary Figs S8–S14, Supplementary Tables S2, S3).

The mean initial magnetic susceptibility (SUS) of samples from OH1 Bed 1 is of ~ 10 × 10^–6^ SI while that of samples from OH1 Bed 2 varies between ~ 100 × 10^–6^ SI and ~ 200 × 10^–6^ SI up to sample S240 to then display a progressive increasing trend up to maximum values of ~ 700 × 10^–6^ SI at the top of OH1 Bed 2 (sample S314) (Fig. 5, Supplementary Table S3). This trend corresponds to a visible reddening of the sediment as observed along the south wall of the excavation trench exposing OH1 Bed 2 (Supplementary Fig. S8). The SUS then drops to ~ 200 × 10^–6^ SI at the base of the coarse-grained quartz-bioclastic sandstones of OH2A to further decreases to a mean of ~ 100 × 10^–6^ SI in the ensuing aeolian sandstones of OH2B (Fig. 5, Supplementary Table S3). Samples S1375 to S1701 belong to aeolian sandstones of uncertain stratigraphic attribution and will not be further discussed (OH? in Fig. 5). The mean SUS of OH3 is of ~ 50 × 10^–6^ SI, that of OH4 plus GH-CCC/unit 4 is much higher and often above 1000 × 10^–6^ SI, while the SUS of OH5 and AF2 (not shown) are on the order of ~ 350 × 10^–6^ SI and ~ 25 × 10^–6^ SI, respectively (Fig. 5, Supplementary Table S3). Units 1–2 of GH-CCC have not been sampled for magnetostratigraphy as they are attributed to the Late Pleistocene–Holocene.

Thermal demagnetization of a three-component isothermal remanent magnetization (IRM) shows for all studied samples a dominant signal carried by the 0.12 T curves with maximum unblocking temperatures of about 575 °C, interpreted as due to magnetite, coexisting with a subsidiary higher coercivity (2.5 T) component with maximum unblocking temperatures of about 680 °C, interpreted as hematite (Supplementary Fig. S9). IRM acquisition curves (not shown) confirm this composite mineralogy. The heating branches of the thermomagnetic (heating–cooling) cycles of selected samples from OH1 Bed 2 show a marked decline in susceptibility generally between 500 and 550 °C attributed to magnetite to then flatten-out reaching temperatures of up to 660 °C due to the presence of hematite (Supplementary Fig. S10A–E). The cooling branches of the thermomagnetic cycles generally show marked increases in susceptibility below about 550 °C due to the creation in the laboratory of new magnetite phases. Hysteresis parameters calculated on the same OH1 Bed 2 samples after correcting for paramagnetic components appear broadly compatible with dominant magnetite in the pseudo-single domain grain-size range (Supplementary Fig. S10F–L). Similar hysteresis data dominated by a low-coercivity magnetic phase (magnetite) have been obtained also for samples from OH4 and OH5 (Supplementary Fig. S11). From the analyses above, it is concluded that the main magnetic phases present in the studied rock record are magnetite in association with hematite. The reddening of the sediment observed in OH1 Bed 2 and corresponding to a marked increase in SUS (Fig. 5; see also above) correlates also to positive and progressively increasing S-ratios and HIRM values (see Materials and Methods for definition). This is tentatively interpreted as due to progressively higher contents of magnetite (S-ratios approaching + 1 and high SUS values) accompanied also by higher contents of high coercivity phases such as hematite and/or goethite (high HIRM values) that are probably responsible for the (chemical) reddening of the sediment associated with soil development (see “Discussion” below).

The anisotropy of magnetic susceptibility (AMS) of the investigated samples is in general more oblate (foliation, F = k_int_/k_min_ > lineation, L = k_max_/k_int_) than prolate (L > F) albeit the degree of anisotropy P = k_max_/k_min_ is in general very low (Supplementary Fig. S12). The k_min_ axes of these weakly anisotropic ellipsoids result broadly perpendicular to the bedding planes especially in OH1 Bed 1, OH1 Bed 2, and OH4 plus GH-CCC, a disposition interpreted as indicating a dominant sedimentary (gravitational) settling of magnetic grains, while the k_min_ axes of the aeolian sandstones of OH2B, OH3, and OH5 tend to lie off the normal to bedding planes, a disposition consistent with (wind-induced) imbrication of clasts (Supplementary Fig. S12).

The intensity of the natural remanent magnetization (NRM) at room temperature varies between 0.02 × 10^–3^ A/m and 40 × 10^–3^ A/m (10 cc volume). Vector end-point demagnetization diagrams of NRM thermal demagnetization data (Supplementary Table S2) show the presence of a soft component of the remanent magnetization termed A that is removed between room temperature and ~ 150–200 °C (Supplementary Fig. S13) and is oriented downward (positive inclinations) with scattered, generally northerly declinations indicative of normal magnetic polarity (Supplementary Fig. S14 upper panel). This A component is virtually absent in samples from OH1 Beds 1 and 2 (Supplementary Fig. S13A for OH1 Bed 2) while it is variably evident in samples from OH2A–B to OH5 taking the form of an abrupt jump in remanence intensity from room temperature to 100–150 °C accompanied by a change in direction of magnetization in case the subsequent component of magnetization at higher temperatures (see below) is of reverse polarity (Supplementary Fig. S13B–E). A characteristic remanent magnetization component, termed C, was isolated in a total of 117 samples between ~ 200 and ~ 475 °C, occasionally up to 600–650 °C. These C component directions are bipolar whereby they are either oriented downward with scattered northwesterly declinations or upward with southerly declinations (Supplementary Figs S13, S14 lower panel), which especially in samples from OH1 Bed 2 (Supplementary Fig. S13A) and AF2 (Supplementary Fig. S13E) appear very shallow possibly due to post-depositional sediment compaction. These C component directions are interpreted as acquired during periods of either normal or reverse polarity of the Earth’s magnetic field (see age attribution below). The maximum angular deviation (MAD) values of these C components, generally < 20°, together with the virtual geomagnetic pole (VGP) latitude they provide, are used to delineate magnetic polarity stratigraphy (Fig. 5, Supplementary Table S3).

Persistent negative VGP values interpreted as recording reverse magnetic polarity characterize OH1 Beds 1 and 2 as well as OH2A up to the base of OH2B, whereas only a few samples from the remainder part of OH2B yielded usable C component directions (Fig. 5). Sample 04.19 in OH2A contains a clear high temperature C component direction of positive VGP value attributed to normal magnetic polarity (Fig. 5, Supplementary Fig. S13B). Next, OH3 (sampled along main section OH3-A) yielded three lower samples of reverse polarity (e.g. sample 3OH121 in Supplementary Fig. S13C) overlain by three samples of normal polarity (e.g., samples 3OH405 and 3OH490 in Supplementary Fig. S13C) (Fig. 5). Magnetic component directions from sister section OH3-B (not reported in Fig. 5) resulted mostly unstable except for two samples with C component directions of reverse polarity (e.g., sample 3BOH388 in Supplementary Fig. S13C). Samples from OH4/unit 5 yielded reverse magnetic polarity, while the three samples from GH-CCC/unit 4 as well as all samples from younger OH5 display positive VGP values interpreted as recording normal magnetic polarity (Fig. 5; examples in Supplementary Fig. S13D). Finally, 7 out of 16 samples from AF2 yielded northerly and very shallow C component directions (Supplementary Fig. S13E) interpreted as a record of normal magnetic polarity (not shown in Fig. 5).

Taking into account also the available OSL data (see also below), the observed polarity sequence is interpreted, from younger to older, as a record of the Brunhes Chron (C1n) in AF2, OH5, and GH-CCC/unit 4, with the Brunhes–Matuyama boundary (C1n–C1r) placed in the lowermost part of GH-CCC/unit 4 or at the boundary with underlying OH4. Next follows a record of the Jaramillo–Matuyama boundary (C1r.1r–C1r.1n) in OH3 (section OH3-A), and an extended record of the pre-Jaramillo Matuyama (C1r.2r) in OH2A and OH2B, OH1 Bed 2, and OH1 Bed 1 (Fig. 5). Tentatively, the one sample-based normal polarity in OH2A could represent a partial record of the Cobb Mountain subchron (C1r.2n). The Brunhes–Matuyama boundary and the Jaramillo–Matuyama boundary—representing the two most secure magnetochronologic time-lines traced within the ThI sequence—are commonly dated to 0.781 Ma and 1.072 Ma according to the Astronomically Tuned Neogene Time Scale (ATNTS2004)^21^, used in this study, or 0.773 Ma and 1.070 using high resolution deep sea piston core data and Ar–Ar data on lavas^22^. The Cobb Mountain subchron is dated to 1.186–1.221 Ma according to radioisotopic data from sections in the western U.S., or to 1.178–1.215 Ma straddling MIS 35 to 36 according to a recent record at IODP Site U1306^22^, which represents the age span adopted in this study.

**Figure 6:**
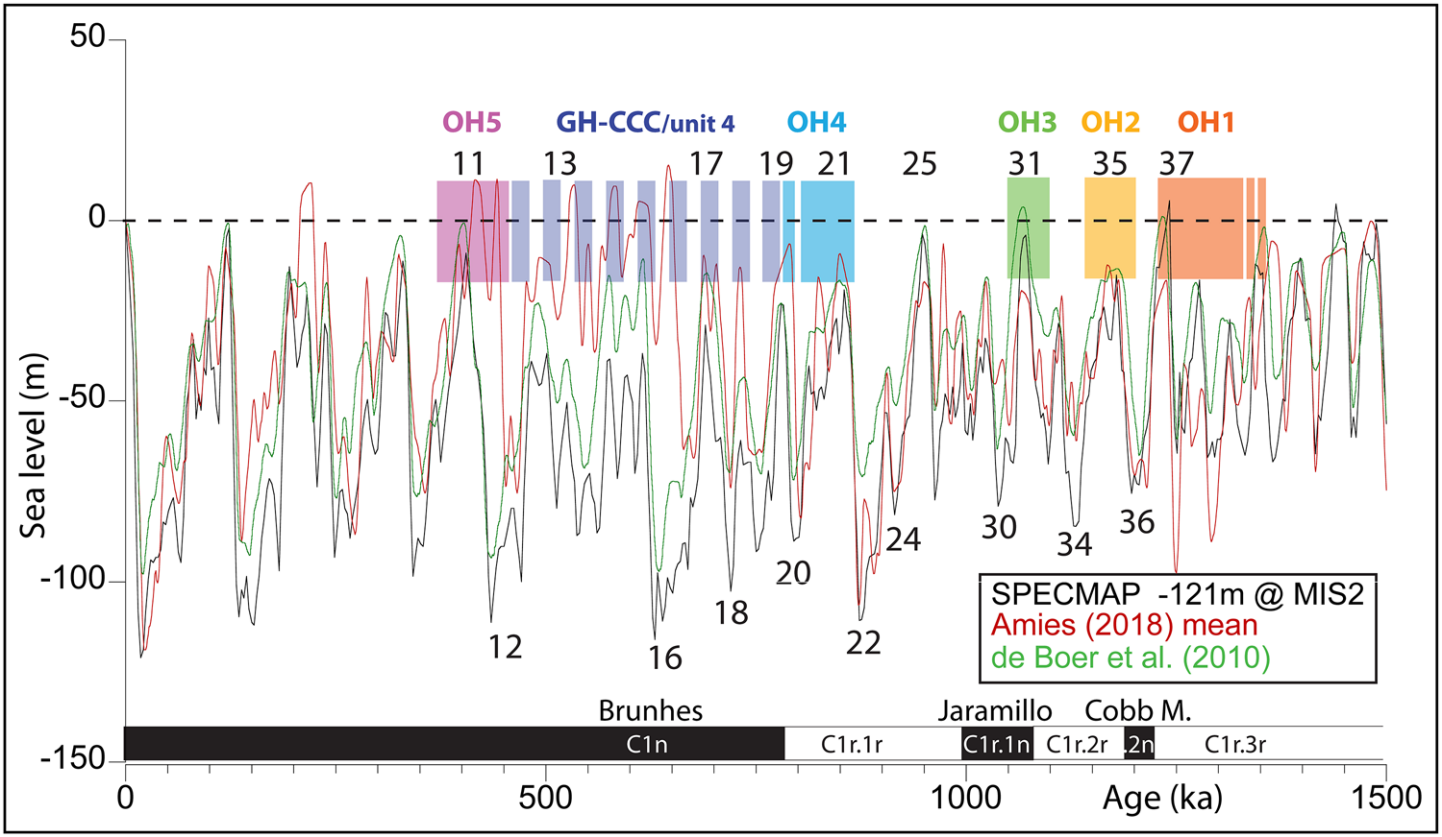
Various glacioeustatic solutions for the past 1.5 Myr as revealed from the ODP677/SPECMAP benthic δ^18^O record^24^ scaled to the 121 m glacioeustatic lowering at the last glacial maximum^25^, the solution of Rohling et al.^26^ based on Amies (2018) SST-corrected^27^ Mediterranean planktic δ^18^O data [solution termed ‘Amies (2018) mean’ in figure], and the solution of de Boer et al.^28^ based on the benthic δ^18^O stack of Lisiecki and Raymo^23^. Main marine isotope stages (MISs) are indicated by standard numbering whereby odd-numbered stages represent interglacials and even-numbered stages glacials. The geomagnetic polarity time scale of the Pleistocene is also reported together with the inferred ages of deposition of the studied units from OH5 to OH1. Drawing G. Muttoni. This figure was created with Adobe Illustrator CS6 version (http://www.adobe.com).

In order to improve the age model of deposition, we consider the effects of Pleistocene glacioeustatic variability on sedimentation according to various solutions from the literature based on δ^18^O records^23–25^ (Fig. 6). Deposition at ThI was most probably intermittent and occurring mainly during sea-level high-stands while erosional surfaces formed during sea-level falls. Therefore, considering magnetochronology, biochronology, and OSL dating, unit OH5 of normal (Brunhes) magnetic polarity with OSL ages from 370 ± 58 to 440 ± 38 ka^13^ can be correlated to MIS 11, GH-CCC/unit 4 of early Brunhes age provided with scattered OSL ages ranging from 391 ± 32 ka to 420 ± 34 ka, a US/ESR age of 501^+94^_–76_ ka, and biochronological constraints^15,17^, is of early Middle Pleistocene age broadly straddling MISs 12/13–19, while OH4 may have deposited during the high-stand of MIS 19 and possibly also MIS 21 (Fig. 6). Similarly, unit OH3 recording the Jaramillo–Matuyama boundary (1.072 Ma) may have deposited during MIS 31, units OH2A and OH2B during MIS 35, and OH1 (Beds 1 and 2) at least during MIS 37 (Fig. 6). A deposition of OH2A–B during MIS 35 would also be in agreement with an attribution of normal magnetic polarity sample 04.19 in OH2A to the Cobb Mountain subchron (Figs 5, 6).

## Geochemistry

Pleistocene climate change impacted the chemical weathering of sediments, with higher carbonate dissolution during warmer and wetter periods^29,30^. Hence, the weathering intensity of ThI-L was determined by geochemical analyses to characterize the climatic conditions of deposition (Supplementary Table S4). K/Ca and Rb/Sr elemental ratios were used to estimate the degree of decalcification of carbonate sediment (Supplementary Figs S15, S16). The high degree of weathering in subunit L1 seems to indicate a warm and humid climate, which is consistent with micropedological indicators and pollen data that suggest a woody environment or a wet depression (Supplementary Information Text, Supplementary Table S6). A shift to drier and cooler conditions could have occurred in L2-L3-L4 given the low values of the weathering index (Supplementary Fig. S17). Reddening and magnetic susceptibility increase in the upper part of OH1 Bed 2 (Fig. 5) which are typical of a soil developed in warm Mediterranean regions with seasonal contrast^31^. This also occurs in L5, which is generally characterized by a high degree of weathering, particularly in subunits L5a and L5c (Supplementary Fig. S17).

In addition, a specific group of elements composed of Si, Ti and Zr was detected by the principal component analysis of the geochemical data (Supplementary Fig. S15). These elements are typical components of Saharan dust transported by the northern branch of the Saharan Air Layers (NSAL) over northwestern Africa^32^. Therefore, the relative enrichment of Si, Ti and Zr in subunits L2-L3 is most likely be related to a period of enhanced Saharan dust flux (Supplementary Fig. S17).

**Figure 7:**
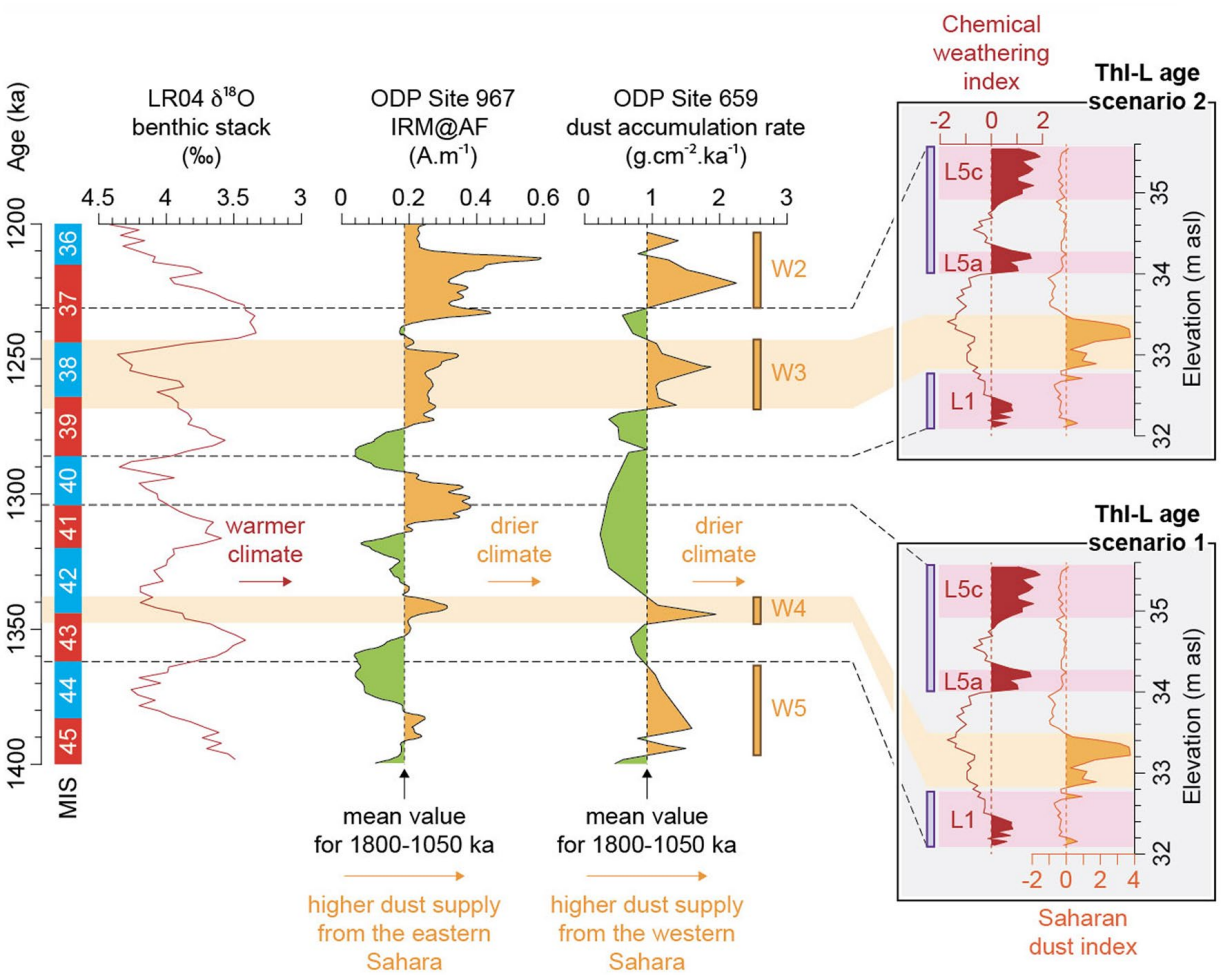
Age scenarios for unit L from the site of Thomas Quarry I. W2–W5: periods of higher than average dust supply from the western Sahara. L1, L5 (a,c): subunits with relatively high chemical weathering. Saharan dust proxy record from ODP Site 659 (dust accumulation rate)^33^. Saharan dust proxy record from ODP Site 967 (IRM@AF)^34,35^. Marine Isotopic Stages (MISs) and LR04 benthic stack^23^. Drawing J.-P. Degeai. This figure was created with Adobe Illustrator CS6 version (http://www.adobe.com).

Thirteen periods of above average dust supply arriving from the western Sahara (W1–W13) can be identified in marine sediments of northwestern Africa for the interval between the Jaramillo and Olduvai subchrons straddling the deposition of ThI-L (Supplementary Fig. S18). We attempt correlating the observed Si, Ti and Zr anomalies in subunits L2-L3 to this dust record by generating an integrated tectono-eustatic model (Supplementary Fig. S19, Supplementary Table S5) and palaeoelevation reconstruction (Supplementary Fig. S20) of ThI-L. Indeed, ThI-L is composed of continental sediments and therefore subunits L2–L3 cannot have been deposited when ThI was flooded by the sea. The site should not have been below sea level during W3 when considering a maximum uplift rate (Supplementary Fig. S20A), during W1, W3 and W12–W13 with a mean uplift rate (Supplementary Fig. S20B), and during W1–W4, W8 and W10–W13 with a minimum uplift rate (Supplementary Fig. S20C). Besides, dust input in subunits L2–L3 is unlikely to be related to W8–W13 because the latter are older than 1.5 Ma (Supplementary Fig. S18), while OSL dating shows that unit L was probably younger than 1.5 Ma^13^. Moreover, if the tentative correlation of the single sample of normal polarity at the bottom of OH2A to the Cobb Mountain subchron is correct, L2 and L3 were probably not associated with W1 and W2, given that unit L was deposited during a period of reverse polarity. Hence, the most consistent periods of high dust supply from the western Sahara which could have led to the Si, Ti and Zr anomalies in subunits L2-L3 should have occurred around either 1.35–1.34 Ma (W4) or 1.27–1.24 Ma (W3). The chronology of unit L could thus range between W5 and W2, i.e., from ca. 1.36 to 1.23 Ma, while chemically weathered sediment and human settlement could date to MIS 43 or MIS 39 for subunit L1 and to MIS 41 or early MIS 37 for subunit L5 (Fig. 7).

## Discussion

Bed 2 of the OHF Member 1 at Thomas Quarry I yielded in some of its sub-units rich lithic assemblages forming the L site and documenting the earliest Acheulean in North Africa. Previously, an age of c. 1 Ma falling either before or immediately after the Jaramillo sub-chronozone was the best date estimate for ThI-L. The site was dated to 989 ± 208 ka and 948 ± 599 ka, using the OSL signal of quartz grains with large uncertainties^13^. However, the lithostratigraphy of the Casablanca sequence showed that the OHF Member 1 lies below formations representing a minimum of three sea-level high-stands older than MIS 15, ranging at least from MIS 17 to 21 (if the sedimentary record is complete, or embracing further isotope stages if the record is incomplete), suggesting an age for OHF Member 1 of at least 1 Ma into the Early Pleistocene^9,10^. Biostratigraphy is in substantial agreement with an Early Pleistocene age older than that of Tighennif^17^.

This study uses magnetostratigraphy and geochemistry to provide a mean age of ~ 1.3 Ma broadly between early MIS 37 and MIS 43 for ThI-L, and in doing so, it establishes the first well-constrained chronostratigraphic framework for the early Acheulean in North Africa.

A reliable chronology for ThI-L expands our knowledge of the emergence of the Acheulean in this part of the African continent and raises several questions on its origin. Due to the scarcity of reliable Early Pleistocene stratified and well-dated sites in North Africa, the timing and mode(s) of the first settlement(s) of this region are poorly understood and many aspects related to the “Out of East Africa” models remain open questions^7^.

Although many archaeological investigations have been carried out in Morocco since the beginning of the nineteenth century, only a few purported pre-Acheulean sites were reported, and all are in disputable stratigraphic contexts^7^. The revision of the lithic collections referred to as the Moroccan Pebble Culture^36^ shows that all the materials are geofacts or younger reworked artefacts^11,37–39^. Furthermore, the rich paleontological site of Ahl al Oughlam dated to ~ 2.5 Ma (Fig. 1) yielded neither lithic artefacts nor evidence of hominid actions on bones^40^. Similarly, pre-Acheulean Algerian lithic series have been mostly collected on surface and reworked deposits^41–47^. The only pre-Acheulean evidence recorded from a stratigraphic context belongs to the Aïn Hanech Formation^48^ in Algeria (Fig. 1), where the last Oldowan occurrence dates to 1.7 Ma^49^. Another possible Early Pleistocene Algerian site with a similar age is Mansourah (Fig. 1), which yielded a lithic industry without LCTs^50^. This sporadic and low-resolution evidence, the absence of pre-Acheulean sites in Morocco, and the wide gap between the Oldowan in Algeria and the early Acheulean of Morocco enable a discussion hinting at a local origin of the ThI-L early Acheulean.

However, although the ThI-L early Acheulean has been discovered in a reliable chronostratigraphic context, the other lithic assemblages of Morocco ascribed to the early Acheulean are from disputable stratigraphic contexts. In layer D of the S.T.I.C. Quarry (Figs 1, 2A), Biberson^36^ discovered a rich quartzite assemblage typologically like that of ThI-L and composed of various types of handaxes, cleavers, trihedrons, bifacial and multifacial cores^36^. Unfortunately, S.T.I.C. Quarry has been completely backfilled and it is impossible to establish the precise relationships between the two sites^7,51^. Also, Biberson ascribed the industry from layer M of Sidi Abderrahmane Quarry (now destroyed by quarrying activity) to the early Acheulean (Figs 1, 2A)^36^. Nevertheless, this layer is younger than ThI-L because it actually belongs to the Member 1 of the Anfa Formation. This member is certainly older than 0.5 Ma^7,51^ as confirmed by the age of 492 ± 57 Ka obtained from the bottom of Member 2 of the Anfa Formation on backshore sands^13^ (Fig. 2A).

The only other early Acheulean site recorded in a secure stratigraphic context in North Africa is Tighennif, in Algeria (Fig. 1). It is difficult to correlate ThI-L with Tighennif: paleomagnetic samples yielded a normal polarity but this site was regarded as problematic for paleomagnetic study^8^. Based on biochronology, Geraads et al.^8^ accepted an age close to 0.7 Ma, but it is becoming increasingly clear that this age was underestimated^12^. Pickford^52^ estimated its age at 1.4 ± 0.3 Ma, based upon his reassignment of the suid from this locality to *Metridiochoerus andrewsi* but his biochronological conclusion is debatable as the sample is similar to the Daka one, well-dated to 1 Ma^53^.

From a technological perspective, LCTs from ThI-L show similar technical traits to those from Tighennif, such as the use of cobbles and *entame* flakes as blanks, the short shaping sequences, and the pointed forms^18^. On the other hand, the Kombewa strategy on entame flakes documented at Tighennif^18^ is absent at ThI-L as is a massive cleaver production. These technical innovations documented in the African Acheulean from 1 Ma in Algeria, introducing a next stage within the Acheulean, i.e. the Large Flake Acheulean^54^, are not present at ThI-L, an absence that is now explained thanks to the new ~ 1.3 Ma dating for the Moroccan site. These novelties appear later at Casablanca in several early Middle Pleistocene sites of the Second Acheulean^7^.

The late Early and Middle Pleistocene of the Late Neogene and Quaternary Casablanca sequence—now provided with a robust chronostratigraphic framework—will undoubtedly be called upon to play a major role in the understanding of the Acheulean emergence and its pan-African dynamics.

## Materials and methods

### Magnetochronology

We sampled a total of 179 stratigraphically superposed core samples in sections straddling the Oulad Hamida Formation as follows: one section in OH1 Bed 1 (8 samples), two sections in OH 1 Bed 2 (47 samples, see also below), one composed section in OH2A and OH2B (44 samples), two overlapping sections in OH3 (15 samples in section OH3-A and 13 samples in section OH3-B), one section in OH4 and GH-CCC (26 samples), and one section in OH5 (9 samples) (Fig. 5, Supplementary Table S2 for thermal demagnetization data and Supplementary Table S3 for data interpretation). Of the 47 samples taken in OH1 Bed 2, 37 samples were drilled along the south wall of the excavation trench (33.568445° N, 7.696421° W) from 4 to 314 cm from the exposed top of OH1 Bed 1, while an additional 10 samples were drilled along the north wall of the same trench from 7 to 82 cm from the exposed top of OH1 Bed 1. We also took 16 core samples in the Anfa Formation (AF2) at the site of Sidi Abderrhamane. 170 out of 179 core samples were taken in the field using a water-cooled electric drill and oriented with a magnetic compass, while the remainder 9 core samples derived from 6 hand samples taken in section OH2A and 3 hand samples in section OH4. All core samples were reduced in the laboratory to standard 10 cc paleomagnetic core samples for analyses.

The initial susceptibility of all standard 10 cc samples was measured with a KLY-2 Kappabridge (Fig. 5). A representative suite of samples has been subjected to backfield acquisition curves of isothermal remanent magnetization (IRM) up to 2.5 T and thermal demagnetization of a three component IRM using fields of 2.5 T, 0.4 T, and 0.12 T (three component IRM in Supplementary Fig. S9). A suite of rock fragments from OH1 Bed 2 (trench south wall) has been studied for S-Ratio =  − IRM_−0.3 T_/SIRM_2.5 T_, obtained by applying a saturation IRM at 2.5 T followed by an antipodal IRM at 0.3 T, and HIRM = (IRM_−0.3 T_ + SIRM_2.5 T_)/2 in order to investigate the reddening of the sediment occurring along this section. S-Ratio approaching + 1 reveals the occurrence of dominant low coercivity magnetic phases (e.g. magnetite) while S-Ratio approaching − 1 high coercivity phases (e.g., hematite, goethite), while the higher the HIRM the higher the content of high coercivity phases (e.g., hematite, goethite) relative to low coercivity phases (e.g. magnetite). We also performed thermomagnetic (heating–cooling) cycles using a MFK-1 Kappabridge with a CS-3 furnace and hysteresis analyses using a MicroMag 2900/3900 Vibrating Sample Magnetometer (VSM) at ETH Zurich on selected samples from OH1 Bed 2 (Supplementary Fig. S10), and hysteresis analyses using a MicroSense EZ7 VSM at the University of Milan on samples from OH4, GH-CCC, OH5, and AF2 (Supplementary Fig. S11). In addition, we carried out anisotropy of magnetic susceptibility (AMS) analyses on virtually all samples with a KLY-3 Kappabridge from which we defined the maximum (k_max_), intermediate (k_int_), and minimum (k_min_) susceptibility axes of samples and the related standard parameters of foliation F = k_int_/k_min_, lineation L = k_max_/k_int_, degree of anisotropy P = k_max_/k_min_, and shape parameter T = 2ln(k_int_/k_min_)/ln(k_max_/k_min_) − 1 (Supplementary Fig. S12).

Samples for magnetostratigraphy have been thermally demagnetized in ~ 20 subsequent demagnetization steps from room temperature up to a maximum of 675 °C with an ASC furnace, and the natural remanent magnetization (NRM) measured after each demagnetization step with an AGICO JR-6  spinner magnetometer at the ALP paleomagnetics laboratory (Peveragno, Italy), except for samples from OH1 Bed 1 which were measured on a 2G Enterprises DC SQUID cryogenic magnetometer at ETH Zurich (thermal demagnetization data in Supplementary Table S2). Standard least-square analysis was used to calculate magnetic component directions from vector end-point demagnetization diagrams (Supplementary Fig. S13A–E), and standard Fisher statistics were used to analyze the mean component directions (Supplementary Fig. S14).

### Geochemistry

Seventy-one sediment samples were collected from ThI-L for geochemical analyses with a vertical sampling step of five centimeters on average. These samples were analyzed by energy-dispersive X-ray fluorescence (ED-XRF) spectrometry with a Delta Innov-X spectrometer equipped with a 4-W Au-tube. Each sample was dried then ground into a fine powder, which was placed in a crystal polystyrene tube and covered with an ultrafine polyethylene film. The parameters of voltage, amperage and counting times were as follows: 10 kV, 0.2 mA, 10 s for Si, K and Ca; 15 kV, 0.2 mA, 45 s for Ti and Mn; 40 kV, 0.1 mA, 5 s for Fe; 40 kV, 0.1 mA, 30 s for Zn, Rb, Sr and Zr. The elemental concentrations were obtained using the Compton Normalization calibration method^55^. The measurement uncertainty is lower than ± 5% for K, Ca, Ti, Mn, Fe, Rb, Sr, Zr, and between ± 5 and ± 10% for Si and Zn.

## Supplementary Information

Supplementary Information.
