# Supplementary material for: First high resolution chronostratigraphy for the early North African Acheulean at Casablanca (Morocco)
Source: Sci Rep. 2021 Jul 28;11:15340. doi: 10.1038/s41598-021-94695-3 (PMC8319413; doi:10.1038/s41598-021-94695-3)
Supplement: Supplementary file 1 — Supplementary Information. [file 41598_2021_94695_MOESM1_ESM.pdf]

Supplementary Information

First high resolution chronostratigraphy for the early North African Acheulean at Casablanca (Morocco)

Rosalia Gallotti[1-3]*, Giovanni Muttoni[4], David Lefèvre[1,2], Jean-Philippe Degeai[1,2], Denis Geraads[5], Andrea Zerboni[4], Valérie Andrieu-Ponel[6], Matteo Maron[7], Serena Perini[4], Mohssine El Graoui[8], Séverine Sanz-Laliberté[1,2], Camille Daujeard[9], Paul Fernandes[3,8,10], Mathieu Rué[1,10], Lionel Magoga[11], Abderrahim Mohib[8,12], Jean-Paul Raynal[3,8,13]

[1]Université Paul Valéry Montpellier 3, CNRS, UMR 5140 Archéologie des sociétés méditerranéennes, Campus Saint Charles, 34199 Montpellier, France.
[2]LabEx Archimède, Université Paul Valéry Montpellier 3, Campus Saint Charles, 34199 Montpellier, France.
[3]Université Bordeaux, CNRS, UMR 5199 PACEA-PPP, Bâtiment B18 allée Geoffroy Saint-Hilaire CS 50023 F - 33615 Pessac Cedex, France.
[4]Dipartimento di Scienze della Terra "Ardito Desio", Università degli Studi di Milano, Via L. Mangiagalli 34, I-20133, Milano, Italy.
[5]CR2P-UMR 7207, CNRS, MNHN, Sorbonne Université, CP 38, 8 rue Buffon, 75231, Paris cedex 05, France.
[6]Aix Marseille Université, CNRS, IRD, IMBE - Institut Méditerranéen de Biodiversité et d'Ecologie marine et continentale, Technopôle Arbois-Méditerranée, Bât. Villemin, BP 80, F-13545 Aix-en-Provence Cedex 04, France.
[7]Dipartimento di Ingegneria e Geologia, Università "G. d'Annunzio" di Chieti-Pescara, Via dei Vestini 31, 66100, Chieti, Italy.
[8]Institut National des Sciences de l'Archéologie et du Patrimoine, Madinat Al Irfane, Angle rue N°5 et rue N°7, Rabat-Institut, BP 6828, Rabat, Morocco.
[9]HNHP-UMR 7194, CNRS, MNHN, UPVD, Sorbonne Université, Institut de Paléontologie Humaine, 1 rue René Panhard, 75013, Paris, France.
[10]Paléotime, 75 avenue Jean Séraphin Achard-Picard, 38250, Villard-de-Lans, France.
[11]France-Morocco Casablanca project.
[12]Centre d'interprétation du Patrimoine du Gharb, Direction provinciale de la Culture, Quartier administratif, Bd Mohamed V, Kenitra, Morocco.
[13]Department of Human Evolution, Max Planck Institute for Evolutionary Anthropology, Deutscher Platz 6, 04103 Leipzig, Germany.

*Corresponding author: rosalia.gallotti@univ-montp3.fr (Rosalia Gallotti)

1. Litho-chronostratigraphy of Thomas Quarry I

The Casablanca hinterland shows a vast system of longitudinal dune ridges parallel to modern coast, composed of marine and aeolian calcarenites. It records an exceptional succession of palaeoshorelines since the end of the Miocene[1-4].

In the South-West of Casablanca, four Formations associated with four raised platforms and including several Members have been defined from the late Early to the Late Pleistocene: the Oulad Hamida Formation (late Early Pleistocene-early Middle Pleistocene), the Anfa and the Kef Haroun Formations (Middle Pleistocene), and the Dar Bou Azza Formation (Late Pleistocene) (Fig. 2A)[1,5,6].

The sedimentary formations of ThI were succinctly described by Biberson and considered as an extension of the Sidi Abderrahmane formations even if their altitude was higher[7]. The stratigraphic revision carried out in relation to the excavation of the *Grotte à Hominidés* (GH) and Thomas Quarry I-Unit L (ThI-L) archaeological sites showed that these deposits could not be continuous with those of the Sidi Abderahmane quarries, and belonged to an earlier morphostratigraphic unit that we have called the Oulad Hamida Formation (OHF)[1,2,6,8].

1.1 Lithostratigraphy. OHF sits on a major unconformity formed at an altitude of ~28 m above sea level (asl) at the expense of the Cretaceous and Palaeozoic substratum, ~10 m above the platform of the Anfa Formation (AF) in the Sidi Abderrahmane quarries.

OHF includes four allostratigraphic units or Members – OH1 to OH4 from bottom to top (Fig. 2B and Supplementary Fig. S1).

OH1 Member: *Bed 1* is composed of a coarse calcirudite at the base and a stratified coarse-grained biocalcarenite. The lower part of *Bed 2* consists of over 2 m succession of fine to coarse locally trough cross-bedded sands and calcareous mudstone, called L1 to L4. These beds form sequences into little inset channels. In the upper part of each micro-sequence, features due to hydratation-desiccation alternations (platy structure levels), to exundation (thin laminar calcareous crusts), to drying phenomena (fissural polygonal network emphasized by ferric oxides) and to bioturbation (rootlets marks associated to ferric coatings) occur. These deposits are interpreted as evidence of intermittently flowing braided streams in a nearshore fluviolacustrine hydrosystem[5].

Bioclastic sands more or less bioturbated, partially decarbonated and cemented form the upper part- L5- of Bed 2. L5 is subdivided in three sub-units: 5a, light brownish grey clay with a roughly polyhedral structure; 5b, whitish to light reddish sandy clayey silt and 5c, reddish sandy silt. They probably represent aeolian deposits which accumulated in damp vegetated depressions with evidence of pedogenesis during warm episodes of subaerial exposure.

In the limestone deposits of Bed 2 (ThI-L site), several levels contain early Acheulean lithic artefacts[9,10,11], as well as the remains of large mammals dominated by hippopotamus along with *Kobus; Equus* cf *mauritanicus; Elephas* sp. Among the microfauna, there are reptiles; amphibians and rodents, predominantly the *Praomys*; *Paraethomys*; *Meriones* and the *Gerbillus* species.

OH2 Member: Overlying an unconformity above the OH1 Member deposits, OH deposits are coarse biocalcarenites with a curved cross-bedding followed by a finer inclined planar-bedding biocalcarenites formed within intertidal depositional environments (OH2A); vertically there follows massive coarse bioclastic aeolianites about ten or so metres thick (OH2B), the upper part of which is affected by fersialsol pedogenesis.

OH3 Member is associated with a shoreline marked by a basal erosion platform and a cliff carved into OH1 and OH2 deposits, whose base is at an altitude of 37 m NGM. The OH3 deposits are composed from bottom to top of blocs and pebbles from OH1 and OH2 calcarenite and limestone, passing to coarse and/or coquinoid biocalcarenites with inclined planar bedding, overlying at the upper part by aeolian calcarenites.

OH4 Member is associated with a palaeoshoreline marked with cliff and deep cavities cut into OH1 and OH3. In the cavities, OH4 deposits are composed of 1) a calcirudite with a coarse coquinoid matrix and plurimetric collapsed blocks of calcarenites originating partly from OH3 Member which constitutes the cavity ceiling and blocks of calcirudite coming from earlier formations. These deposits are associated with a well-defined notch shaped at the expense of OH1 and OH3 Members at an altitude of about 34 m asl, 2) this chaos of blocks, whose surfaces are smoothed, is drowned out by a fine, planar-bedding calcarenite which is well developed laterally, which is inserted between the blocks and covers them; upwards, these sands become more massive. OH4 marine deposits constitute the lower set of the infilling of the GH large cavity[12,13]. The upper set of the infilling is composed of continental deposits (GH-Continental Cave Complex). Without any apparent disconformity with OH4, GH-CCC lower units 4-3 deposits are composed of bioclastic and quartzose sands containing lithic objects, macrofauna, microfauna and hominin fossils, hence the cavity's name, probably edified in several episodes. The upper part of the GH-CCC is composed from bottom to top of a multilayer dripstone interbedded with loose red sands (GH-CCC-unit 2) which laterally links up with a speleothem, overlying by massive bedded, rubefied sands rich in microfauna and fragments of gasteropods shells, originating from reworked superficials red soils (GH-CCC-unit 1)[12,13].

At the entrance of the cavity, the wide cross-bedding “gray” sands of an aeolianite (OH5) are intercalated between the lower (3-4) and the upper units (1-2) of the GH-CCC.

1.2 Chronostratigraphy. According to the sequence stratigraphy, OH1 to OH4 Member are allostratigraphic units defined by a sedimentary sequence characterized by a succession of genetically related deposits - intertidal, supratidal and aeolian/continental depositional environments - and bounded at its base and top by unconformities. This sequence records sea-levels high-stands associated with the formation of a shoreline marked by a cliff in the case of OH3 and OH4. The unconformities or sequence boundaries mark the regression of the shoreline due to sea-level fall and the continentalisation of the coast. Thus, OH1 to OH4 Members are correlative to sea-levels high-stands of the main global glacio-eustatic cycles as inferred from the marine isotope stage (MIS) record and are preserved at positive elevations due to the regional tectonic uplift affecting Atlantic Morocco.

OHF predates the Anfa Formation (AF) whose Members AF1 and AF2 are correlated to MIS 13 even 15 (Fig. 2A).

The OH4 deposits predates the continental cave deposits of the GH (Fig. 2A). OSL analyzes on GH-CCC-unit 4 yielded ages from 391±32 ka to 420±34[13]. A hominin tooth provided a laser abrasion ICP-MS age of 501 +89/-66 ka[13]. Biostratigraphic data point towards the first part of the Middle Pleistocene[14,15]. OSL dating of quartz provided an age range between 360 ka to 470 ka (370 ± 58 to 440 ± 38 ka) for the aeolianite OH5 intercalated between the lower units (3-4) and the upper (1-2) of the GH-CCC[16]. The stratigraphic position, the facies and the dating allow to correlate this aeolianite with MIS 11 AF3 Member of the Anfa Formation.

The OH1-Bed2 microfauna, particulary the rodents, suggest a placement of the faunal assemblage between the fauna of Aïn Hanech and that of Tighennif in Algeria, i.e, representing the second half of the Early Pleistocene, but probably not in its terminal part[14,15]. This attribution is in agreement with the results of the OSL dating, which provide an age between 0.8 and 1.2 Ma[16].

As continental infillings of the GH cavity have been dated to 0.5-0.6 Ma[12,13,16] in accordance with biostratigraphic data[14,15], OH4 sea-level high-stand associated with the palaeoshoreline is undoubtedly much older than MIS 15. Thus, the OH4 to OH1 Members records sea-level high-stands at least MIS 17 to older, much older if the record is not complete. Nevertheless, since it is difficult to estimate what the large hiatus corresponding to the embedding of about ten meters of the Anfa formation base platform in the Oulad Hamida Formation, and the quartzitic basement, may represent in terms of unrecorded cycles, it cannot be ruled out that these sea-levels high-stands of the OH formation are much older.

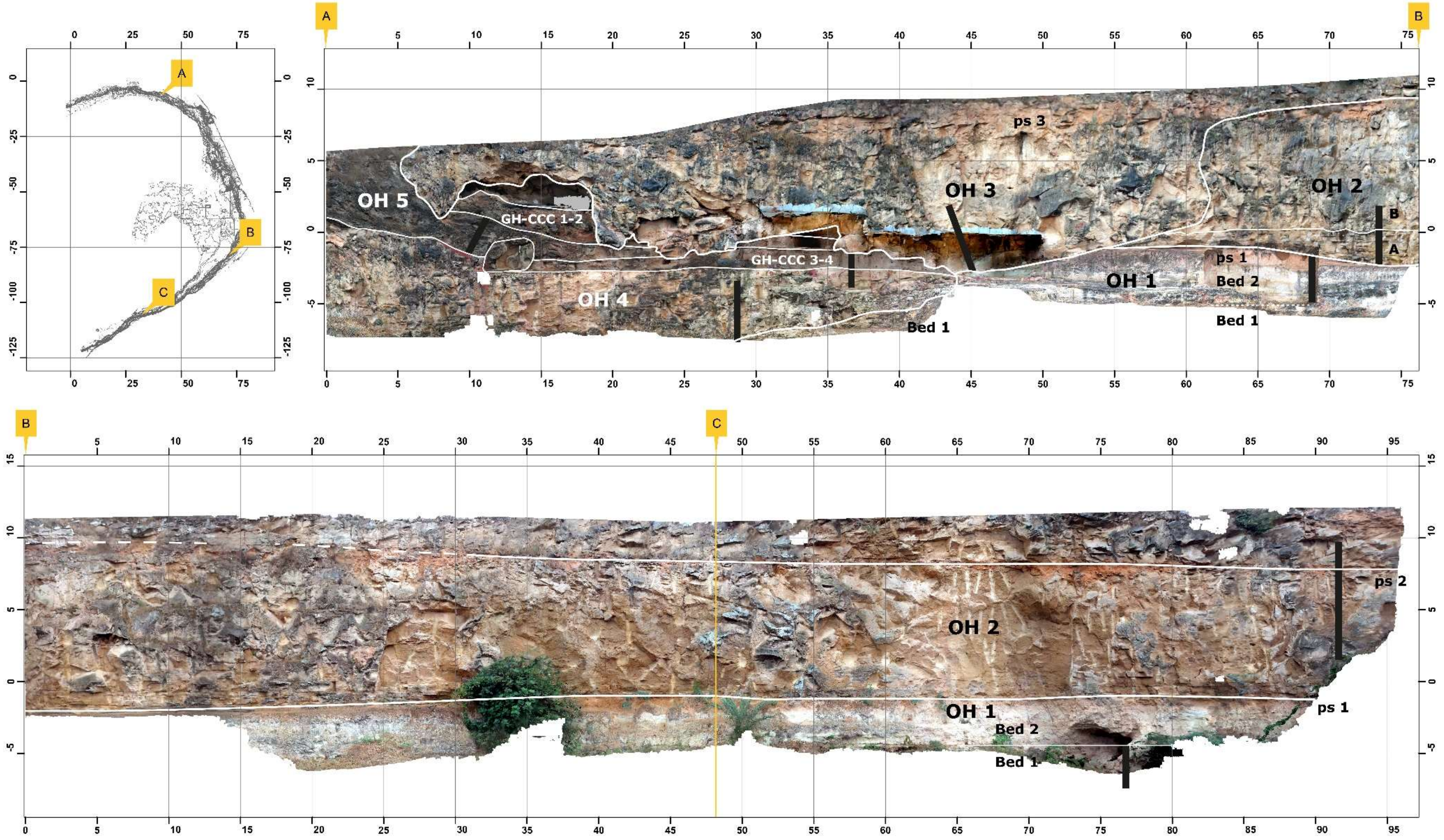

Fig. S1. Oulad Hamida Formation at ThI (photogrammetry and CAD S. Sanz-Laliberté and D. Lefèvre). The black bars indicate the location of magnetostratigraphical samples.

2. Thin sections' micromorphology of OH1 Bed2 - ThI-L

2.1 Methods. Twenty-seven samples were extracted during 1990 excavation from a test pit section in squares U25 (L1 to L5 base) and U28 (L5) of the lithic-bearing stratigraphic section of ThI-L site, OHF Member 1 (OH1), Bed 2. Five samples only were selected from archaeostratigraphic units L1 (9065), L2 (9062) and L5 (9039, 9043, 9054) to manufacture large thin sections (13,5 x 5,5 cm) at the micromorphology facility of Institut du Quaternaire from Bordeaux University (M. El Graoui). They were cut from oriented blocks of sediments vaccum-impregnated with polyester resin, following the process described by Guilloré[17,18].

This preliminary observation was intended first to give support to the interpretation of the field evidence about the depositional and post-depositional processes leading to the formation of each unit and second to search for the occurrence of mineral and organic anthropogenic debris related to human or animal activities[19-22]. Micromorphological studies of thin sections employed an optical petrographic microscope Olympus BX41 equipped with a digital camera Olympus E420. Thin sections were observed at various magnifications (20x, 40x, 100x, 200x, 400x) under plane polarized light (PPL), cross-polarized light (XPL), and oblique incident light (OIL). Additionally, thin sections have been observed under oblique incident radiation (epifluorescence) of ultraviolet (UV) and blue (BLF) lights using a mercury burner (HBO lamp) 100 W, mostly to detect the occurrence of phosphatic features and to assess the degree of bone fragments preservation[23]. The description of thin sections is summarized in Table S1 that follows the terminology suggested by Stoops[24]. Data interpretation follows the concepts on the formation of natural and anthropogenic layers summarized in Courty et al.[25], Nicosia and Stoops[26], and Stoops et al.[27].

2.2 Results. From the sedimentological point of view, Bed 2 presents distinct sedimentary environments that corresponds to facies related to the evolution of nearshore environments.

The lowermost part of the sequence (L1) consists of a packed accumulation of sand-sized, subangular quartz grains with occasional occurrence of glauconite grains, and common to scarce coarse bioclasts (Supplementary Fig. S2). The latter are in part dissolved and testified by moldic voids, pseudomorphic of bioclasts. In some cases, dissolution voids are partially filled by sparitic or micritic cement (Supplementary Fig. S3). The micromass consists of a variable quantity of micrite; in the lower part of L1 the micritic groundmass is almost absent and calcite nodules are present (Supplementary Fig. S2).

The following L2 unit alternates levels consisting of carbonatic mud with interspersed scarce sand-sized quartz grains and very rare bioclasts (Supplementary Fig. S2) displaying calcitic coatings, to levels with abundant sand-sized quartz grains, ghosts of carbonatic clasts, and calcitic pedorelicts (with altered margins) in a micromass of micritic to sparitic calcite (Supplementary Fig. S2). The porosity is generally low in L2 and related to construction voids and occasionally by bioturbation voids (common to scarce channels; Supplementary Fig. S2). Occasional compact microlayers are evident (Supplementary Fig. S4). In L2 altered bone fragments and phosphatic impregnation on the groundmass are occasionally present (Supplementary Fig. S4).

The uppermost part of the sequence (L5) consists of accumulations of coarse sand, which is constituted mostly by subangular quartz grains and occasionally other minerals including glauconite (Supplementary Fig. S2), interspersed in a calcitic micromass. Unit L5 displays a weak to moderate preferential orientation of the coarse constituents, which is detectable thanks to the occurrence of aligned dissolution voids after shell fragments (Supplementary Fig. S5). In the lower part of L5, a thin layer of laminar to stromatolithic calcite is present. Bioturbation is evident in this part of the sequence and the occurrence of Fe-rich oxy-hydroxides staining mineral grains and the rim of voids and impregnating the groundmass in the lower part of L5 is a further evidence of pedogenesis (Supplementary Fig. S3). Anthropogenic debris are occasionally present; in L5 they are represented by fragments of knapped lithic artifacts and bone fragments and phosphatic

impregnation of the groundmass, which can have an animal origin (Supplementary Fig. S5). A few, very small fragments of charcoal are also present, but their attribution to intentional human firing is not possible.

The sedimentary facies observed at the microscale suggest the interplay of distinct sedimentation mechanism in charge of the accumulation of Bed 2. All processes happened in nearshore environments thanks to the interplay between wind and fluvial sedimentation. The basal unit L1 formed after the accumulation of aeolian sand constituted by quartz grains, glauconite grains, and bioclasts (e.g., shell fragments, bryozoan, echinoderm, foraminifera, red algae) deflated presumably from the emerged shelf. The subsequent layer L2 shows a transition towards fluvial processes and the unit likely accreted in correspondence of a system of low to medium energy channels alternated to swamps. In such conditions, decantation of clay and precipitation of calcite occurred in swamp to lake environments, whereas coarse layers suggest increased energy of the system and the onset of a channel system. Likely, it was a braided system that accumulated carbonatic sand-sized grains, limestone fragments, and pedorelicts resulting from the dismantling of the carbonatic mud of the swamp environment. Finally, thin sections from layer L5 suggest a further shift in the sedimentary system and a new onset of wind sedimentation with the accretion of dunes. The shape and size of quartz grains is compatible with the aeolian sedimentary environment and the presence of glauconite grains and abundant bioclast moulds support the interpretation of this unit as eolianites.

All deposits identified in the L5 to L1 sequence suffered strong post-depositional modification. Evidence of bioturbation is evident along the whole sequence as much a huge mobilization and reprecipitation of calcite. Bioclasts in the L5 wind-accreted unit are completely dissolved resulting in abundant dissolution/moldic voids; the same happened to carbonatic grains from the L2 unit, which are represented by micrite envelopes[28]. Calcite reprecipitated as micrite cementing the micromass or occasionally as sparite; in many cases, secondary calcite formed bridges among grains and precipitated inside the voids of former mineral grains and bioclasts[19,28]. As common in carbonatic soils of the Mediterranean area, the typical calcitic crystallitic b-fabric is the combination between calcite inherited from sedimentary parent material and gradual precipitation of micrite in the micromass and filling of pore space between grains by precipitated silt- and clay-size pedogenic carbonate crystals[29-32]. Incipient formation of calcite nodules is evident in the basal part of the sequence (L1). Bioturbation is evident in deposits related to wind sedimentation suggesting a moderate vegetal cover. Also, carbonatic mud observed under the microscope display moderate to weak occurrence of bioturbation (channel voids) due to vegetation or invertebrates. L5 suffered initial pedogenesis under warm environmental conditions during episodes of subaerial exposure; this is suggested by the occurrence of Fe-oxyhydroxides impregnating the groundmass and forming coatings on grains and voids[33-35]. Warm conditions after the deposition of the sand deposit of L5 are further confirmed by the occurrence of micritic ondulated laminar layers that can be interpreted as thin calcrete[28,36]. Such processes (calcite redistribution and Fe-bearing pedofeatures) are common in Pleistocene eolianites deposits from the Mediterranean region and elsewhere[37-40] and likely promoted by the transpiration of dune plants[28,34,36].

Compact layers detected in L2 and repeated alignments of elongated constituents in L5 are syndepositional features likely related to runoff or eolian processes even if trampling associated to human occupation of the site could also be suggested[41,42]. The accumulation of bone fragments is also due to animal and/or human accumulators. Observation of bone fragments under BLF light suggests that they are moderately altered and partially impregnated by Fe-oxyhydroxides. The fluorescence light highlighted the presence of accumulation of phosphates in the groundmass of L5 and (less abundant) L2, likely formed after weathering under warm conditions of bone fragments[23,43,44]. Finally, the occurrence of lithic fragments[45,46] confirms an in situ knapping activity already revealed by the excavation.

2.3 Conclusion. The observation of thin sections from sediments of ThI-L under the microscope highlighted the major sedimentary properties of the deposit and strong post-depositional and

taphonomic processes affecting the whole sequence. The final product is a sedimentary sequence alternating coarse siliciclastic to bioclastic sands and calcareous mudstones. The formation of the sequence is likely related to the interplay of littoral processes that include sedimentation in low energy channel interchanged to swamps along backshore environments, alternated to periods of enhanced aeolian sedimentation. Moreover, the whole sequence displays strong evidence of pedogenesis and diagenesis, including huge calcite mobilization and redistribution and bioturbation, resulting in layers with huge accumulation of micritic cement in the groundmass and abundant dissolution cavities related to pristine calcitic constituents (mineral grains and bioclasts). In the uppermost part of the sequence (unit L5), evidence of pedogenesis during warm episodes of subaerial exposure and increased vegetation cover are preserved.

A limited human overprint on sedimentation and taphonomy of deposits is supposed in two distinct layers. In the uppermost part of L5, biogenic/anthropogenic indicators[47] are interspersed in the groundmass and include fragments of knapped lithic artefacts and minute fragments of altered bones. In the same unit, very small charcoal fragments of unknown origin have been identified. In the same layer a weak to moderate preferential orientation and alignment of dissolution voids (mould of shell fragments) was detected but more investigations are necessary before attributing them to repeating trampling on the same surface rather to ordinary bioturbation. Moderately preserved fragments of altered bones were also identified in layer L2; under fluorescence light and the same thin section displays evidence of phosphatic impregnation of the groundmass, which is generally related to the deep weathering of bone fragments and the same unit displays occasional evidence of compaction features that could be associated to trampled surfaces. However, one must keep in mind that these low energy channels and swamps environment was at least heavily frequented by hippos and crocodiles.

Table S1. Summary of micromorphological properties of samples.

Sample	Layer	Depth (cm)	Mineral consitutents	Micromass	Organic and anthropogenic constituents	Voids	c/f related distribution	b-fabric	Microstructure	Pedofeatures
9039	L5	-223–233	Common to abundant sand-sized quarts grains, subangular to poorly rounded; very scarce igneous grains; scarse altered bioclasts	Brownish grey clayey micromass impregnated of calcite	Scarce, small bone fragments, partially weathered; scarce fragments of knapped lithic; very rare small charcoal fragments	Common vughs; scarse channels and planes; common mouldic voids after bioclasts dissolution, occasionally aligned	Single spaced porphyric	Crystallitic to undifferentiated	Massive to complex	Common crystalline pedofeatures related to calcite dissolution and redistribution, including sparitic and micritic coatings on voids and clasts, infillings of mouldic voids, hypocoatings, and impregnation of the gorundmass; scarse phosphatic impregnation on the groundmass; weak evidence of Fe-oxyhydroxides impregnationon on the groundmass and around discrete elements; weak Mn-rich oxyhydroxides impregnation on bone fragments; scarce passage features
9043	L5	-283–273	Common to abundant sand-sized quarts grains, subangular to poorly rounded; scarce glauconite clasts; very scarce igneous grains; scarce altered bioclasts	Brownish grey clayey micromass impregnated of calcite	Scarce, small bone fragments, partially weathered	Common vughs; rare to scarce channels and planes; common mouldic voids after bioclasts dissolution	Single spaced to close porphyric	Undifferentiated	Massive to complex	Common crystalline pedofeatures related to calcite dissolution and redistribution, including sparitic and micritic coatings on voids and clasts, infillings of mouldic voids, hypocoatings, and impregnstion of the gorundmass; scarce calcite nodules; weak evidence of Fe-oxyhydroxides impregnation on on the groundmass and around discrete elements; weak Mn-rich oxyhydroxides impregnation on bone fragments; scarce passage features
9054	L5-L4	-326–335	Common sand-sized quarts grains, subangular to poorly rounded; common carbonatic clasts; very scarce altered bioclasts	Brownish grey clayey micromass impregnated of calcite; in the upper part, weakly micritic ondulated laminar layers		Common vughs; scarce to common channels and planes; common mouldic voids after bioclasts dissolution	Single to double spaced porphyric; occasionally close porphyric	Crystallitic to undifferentiated	Massive to complex	Common crystalline pedofeatures related to calcite dissolution and redistribution, including sparitic and micritic coatings on voids and clasts, infillings of voids, hypocoatings, and impregntion of the gorundmass; scarce calcite nodules; common Fe-oxyhydroxides impregnationon on the groundmass and around discrete elements
9062 haut	L23	-402–414	Scarce to common sand-sized quarts grains, subangular to poorly rounded; common to scarce carbonatic clasts; very rare bioclasts	very scarce micritic micromass	Scarce, small bone fragments, partially weathered	Common vughs; scarce channels and planes; scarce mouldic voids after limestone clasts and bioclasts dissolution	Close porphyric	Crystallitic	Massive to complex	Common crystalline pedofeatures related to calcite dissolution and redistribution, including sparitic and micritic coatings on voids and clasts, infillings of voids (including mouldic voids), hypocoatings, and impregnation of the gorundmass; scarce calcite nodules; ripening layers; in the lower part of the sample, calcitic pedorelicts with erosional margins and Fe-impregnation; tion of the gorundmass;
9062 bas	L23	-417–429	Scarce to common sand-sized quarts grains, subangular to poorly rounded; common to scarce carbonatic clasts; scarce rock fragments (limestone); very rare bioclasts	Brownish grey clayey micromass impregnated of calcite		Common vughs; scarce channels and planes; scarce mouldic voids after limestone clasts and bioclasts dissolution	Close to sulgle spaced porphyric	Crystallitic	Massive to complex	Common crystalline pedofeatures related to calcite dissolution and redistribution, including sparitic and micritic coatings on voids and clasts, infillings of voids (including mouldic voids), hypocoatings, and impregnation of the gorundmass; scarce calcite nodules; calcitic pedorelicts with erosional margins and Fe-impregnation; tion of the gorundmass; scarce lithorelicts
9065 haut	L1 top	-455–467	Common to abundant sand-sized quarts grains, subangular to poorly rounded; very scarse igneous grains; scarce altered bioclasts	Brownish grey clayey micromass impregnated of calcite		Common vughs; scarse channels and planes; common mouldic voids after bioclasts dissolution	Single spaced to close porphyric	Crystallitic to undifferentiated	Massive to complex	Common crystalline pedofeatures related to calcite dissolution and redistribution, including sparitic and micritic coatings on voids and clasts, infillings of mouldic voids, hypocoatings, and impregnstion of the gorundmass; scarce calcite nodules; very weak Fe-oxyhydroxides impregnation on the groundmass
9065 bas	L1 top	-460–472	Common to abundant sand-sized quarts grains, subangular to poorly rounded; scarce glauconite clasts; very scarce igneous grains; scarce altered bioclasts	Scarce brownish grey clayey micromass impregnated of calcite		Common vughs; scarse channels and planes; common mouldic voids after bioclasts dissolution	Close porphyric	Crystallitic to undifferentiated	Massive to complex	Common crystalline pedofeatures related to calcite dissolution and redistribution, including sparitic and micritic coatings on voids and clasts, infillings of mouldic voids, hypocoatings, and impregnstion of the gorundmass; scarce calcite nodules; common abundant calcitic nodules

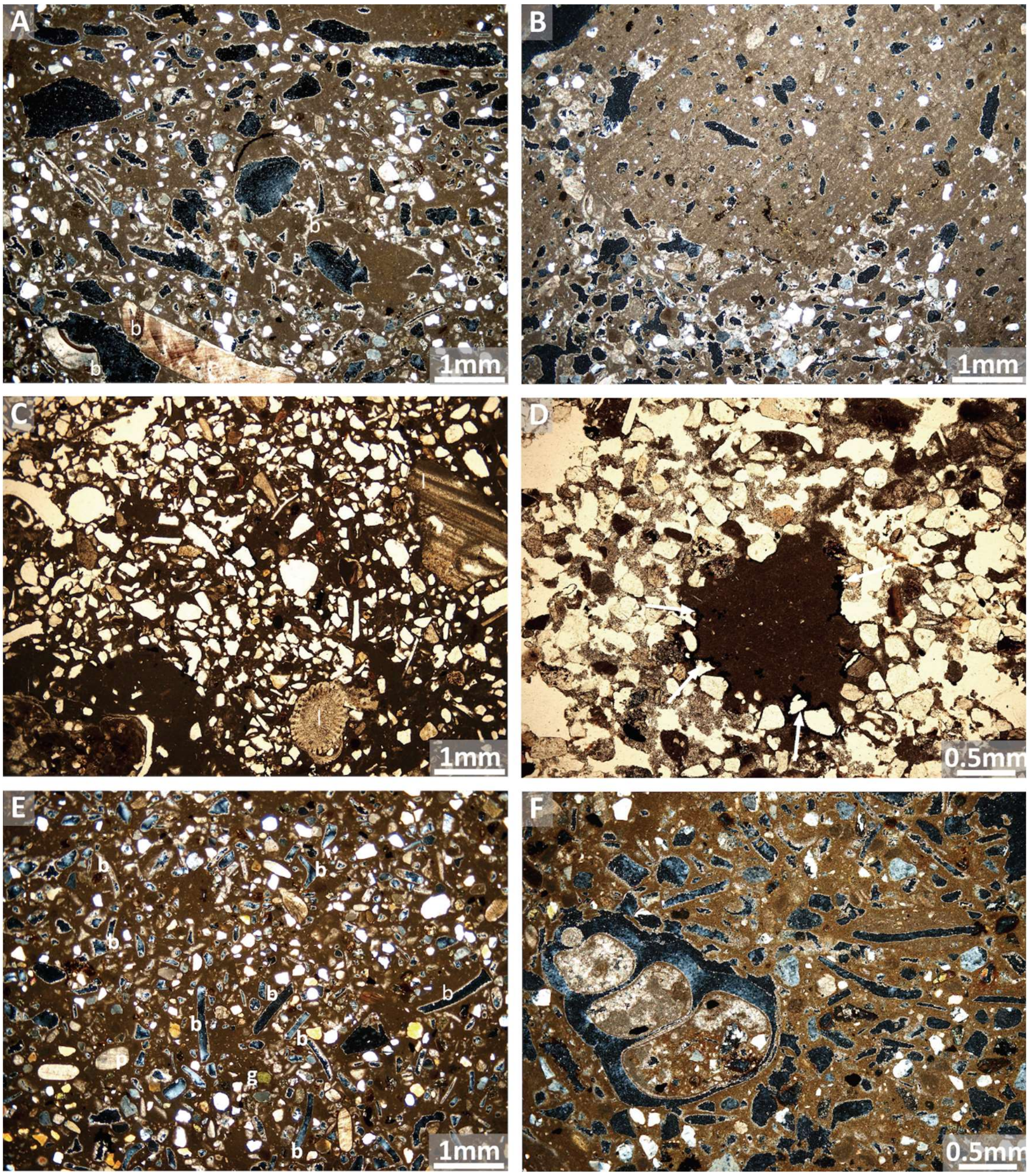

Fig. S2. General aspect of the observed thin sections from layers L1–L5 illustrating variations in the groundmass and constituents that reflects changes in sedimentary environments. (A) Single spaced porphyric c/f related distribution of the sandy L1 unit; notice the occurrence of common bioclasts (B) (XPL). (B) Open porphyric c/f related distribution of the carbonatic mud in L2 (XPL). (C) Close to single spaced porphyric c/f related distribution of the sandy layer in L1 related to moderate energy fluvial system; notice the occurrence of lithorelicts (l) (PPL). (D) A pedorelict (a micrite nodule with altered margins indicated by the arrows) in the L1 unit (PPL). (E) Close to single spaced porphyric c/f related distribution of the sandy L5 unit; notice the occurrence of subangular quartz grains, glauconite grains (g), and common bioclasts (B) (XPL). (F) A detail of the voids pseudomorphs of shell fragments with partial reprecipitation of micrite in L5 (XPL).

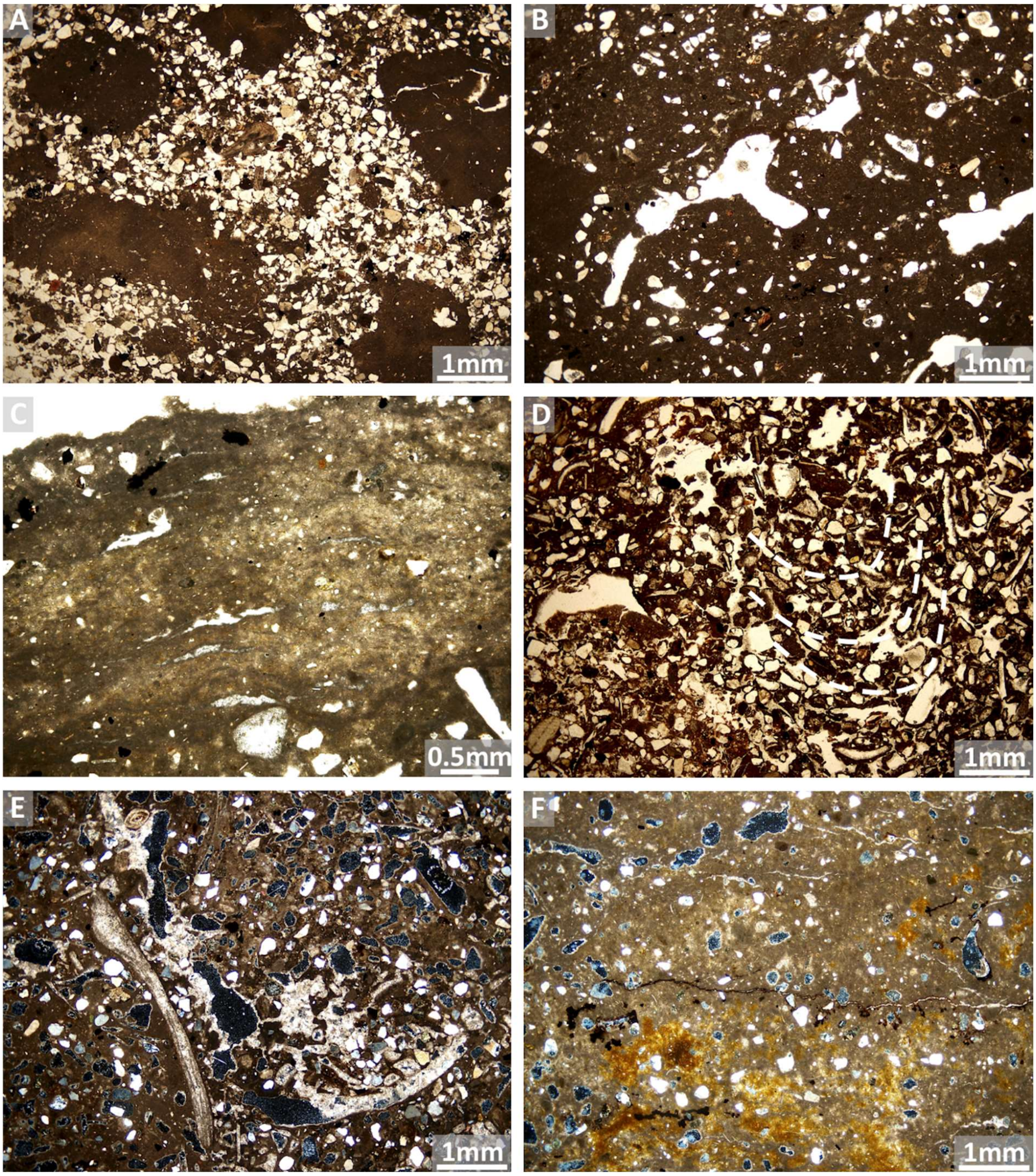

Fig. S3. Photomicrographs illustrating post-depositional processes (pedogenesis and diagenesis) affecting the sedimentary sequence of Bed 2. (A) Calcitic nodules in the sandy groundmass of L1 (PPL). (B) Channel voids due to bioturbation in the mudstone of L2 (PPL). (C) Micritic undulated laminar layer (calcrete) in L5 (PPL). (D) Dashed lines highlight a passage pedofeature (bioturbation) in L5 (PPL). (E) Strong dissolution of bioclast and reprecipitation of micrite and sparite in L5 (XPL). (F) Fe-rich oxy-hydroxides impregnating the groundmass and coating voids and grains in the lower part of L5 (XPL).

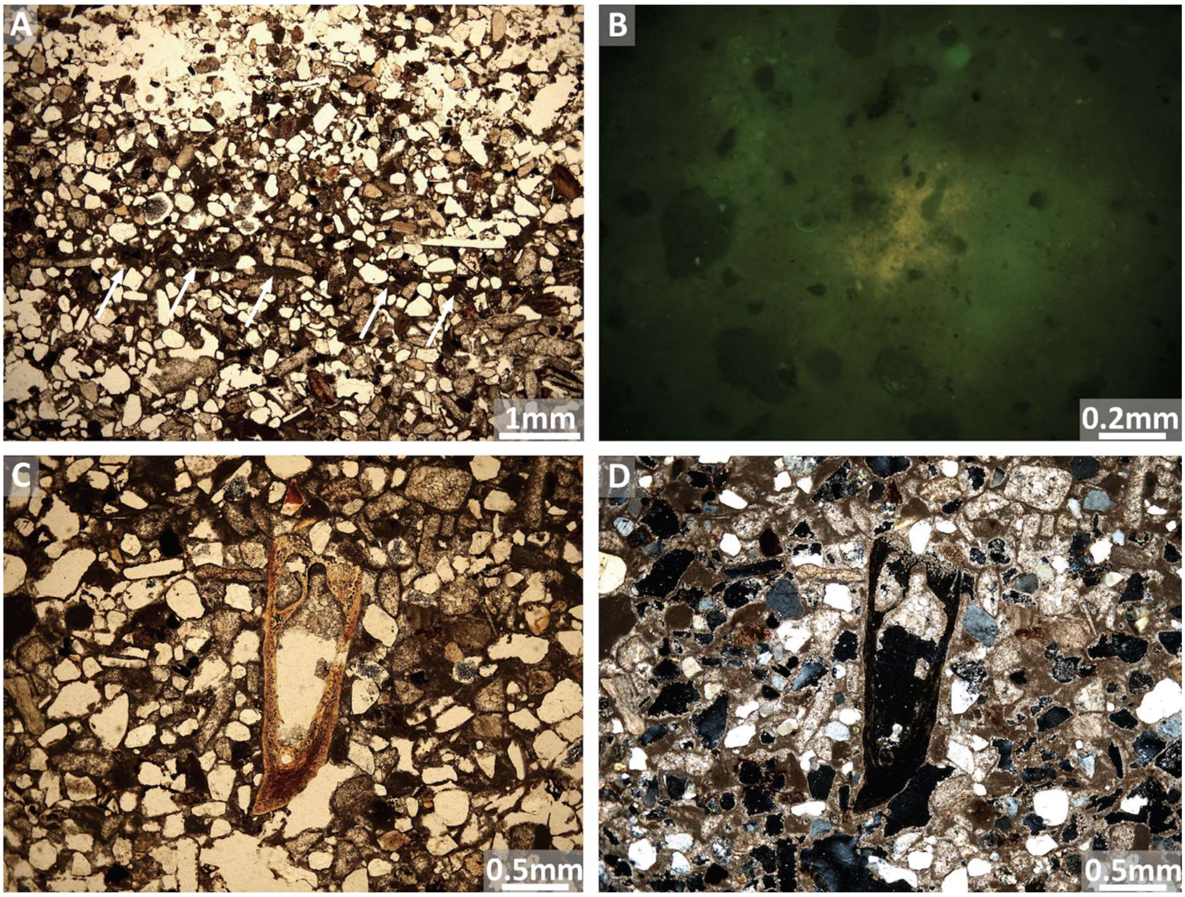

Fig. S4. Photomicrographs of some microscopic features detected unit L2. (A) Evidence of compaction (indicated by arrows) in the upper part of L1 (PPL). (B) Phosphatic impregnation on the groundmass (BLF). (C) A weakly altered bone fragment in L2; notice the abundant occurrence of moldic voids after grains dissolution with micrite envelope or secondary sparite/micrite precipitation (PPL). (D) the same of (C) in XPL.

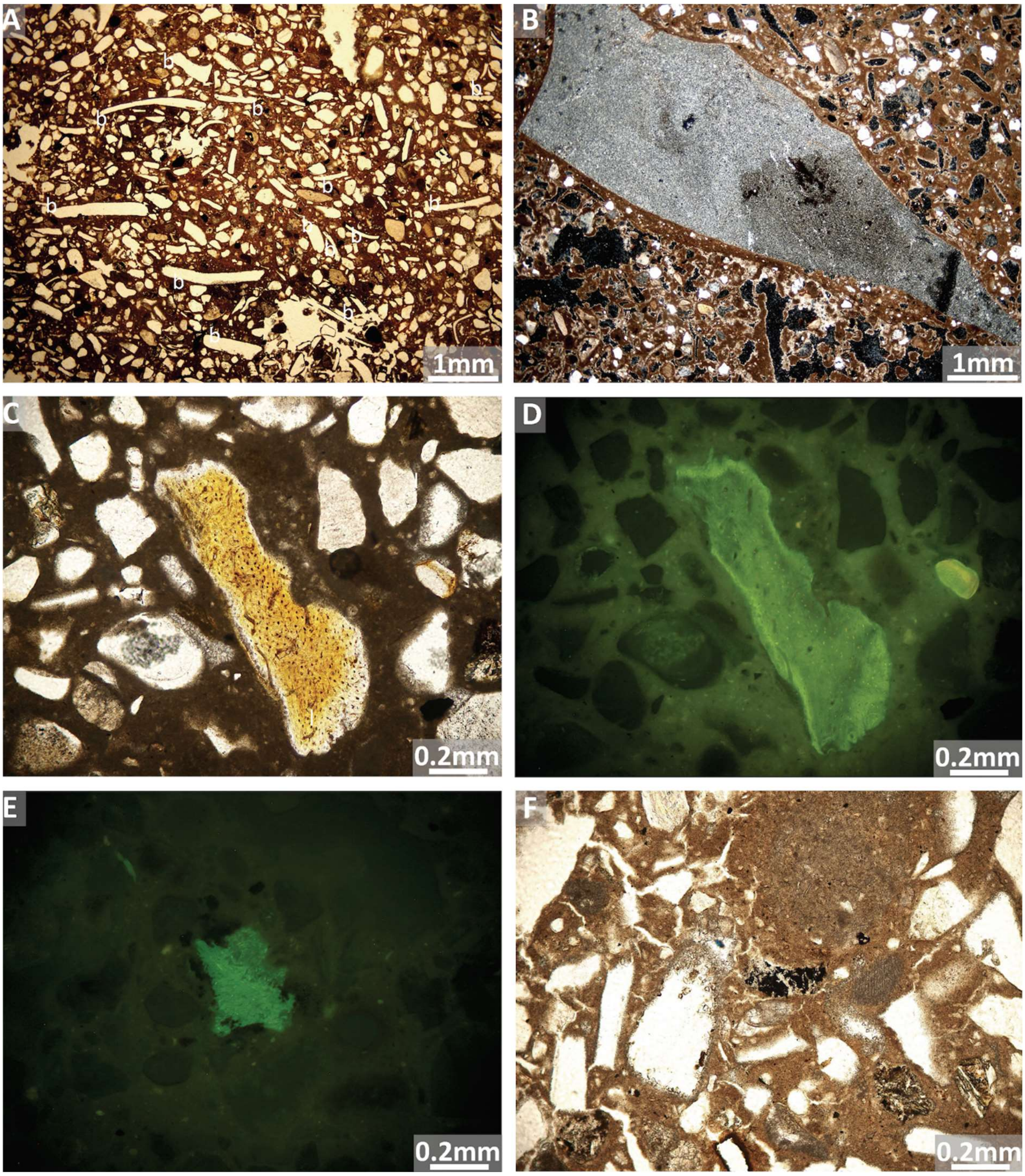

Fig. S5. Photomicrographs of some microscopic features detected in unit L5. (A) Preferential alignment of moldic voids, pseudomorphic of bioclasts (b) in the upper part of L5 (PPL). (B) A knapped lithic artifact (XPL). (C) A weakly altered bone fragment (PPL). (D) the same of (C) in BLF. (E) A bone fragment in BLF with Fe-oxyhydroxides impregnation on its margins. (F) A small fragment of charcoal (PPL).

3. Paleontology, biochronology, and zooarchaeology

3.1 Paleontology and biochronology. Large mammal remains are rare and fragmentary at ThI-L. Not unexpectedly in this environment, they are dominated by a large hippo probably identical with *Hippopotamus sirensis* from Tighennif (itself probably identical with *H. gorgops* from East Africa). A complete suid upper M3 was assigned to *Kolpochoerus maroccanus* by Geraads[15], but if this species belongs in fact to *Metridiochoerus* as hypothesized by Souron[48], this tooth must in fact belong to either *K. majus* or to *K. limnetes/olduvaiensis*, all known in the East African Early Pleistocene. It is more simply built and smaller than most *K. olduvaiensis* teeth from the Early Pleistocene, slightly larger than *K. majus* teeth from the upper Konso levels[49], and more like teeth from Asbole[50] or Daka[51] but we refrain from drawing biochronological conclusions from a single tooth. Bovids include a bovin, a gazelle, a medium-size alcelaphin, and a medium-size *Kobus* sp. An *Equus* and a rhino are also present, together with an Elephantidae, probably *Elephas*, although the poorly loxodont pattern of some *Loxodonta atlantica* teeth from Tighennif makes genus identification difficult.

A single tooth of *Crocidura* sp. is documented. An upper anterior premolar of Lagomorph (*Lepus* sp.?) is too primitive to belong to the common hare of the Middle Pleistocene of these quarries, *Trischizolagus raynali,* but could be of *Lepus*, already present at Ahl al Oughlam.

Rodents are represented by 15 teeth of which only three belong to murins, and 12 to gerbillins, suggesting rather arid environment. The absence of the dormouse *Eliomys* can be due to incomplete sampling, but that of the mole vole *Ellobius* is perhaps significant, as this genus is usually common when present. This genus is an Asian immigrant that first appears at Tighennif, suggesting that this Algerian site might be younger than ThI-L. A molar fragment of the murin *Paraethomys* differs from all other *Paraethomys* found in Morocco, including the *P. tighennifae* from other levels in Thomas and Oulad Hamida Quarries, but is reminiscent of *P. mellahe* Ameur, 1988 from Oued Mellah, an Algerian site earlier than Tighennif, according to Ameur[52]. A few teeth document two unnamed species of *Gerbillus*, both different from the species known at *Grotte à Hominidés,* at *Grotte des Rhinoceros* of the nearby Oulad Hamida 1 Quarry and at Tighennif [8]. The evolutionary grade of the smaller form is intermediate between those of G. *minutus* from the Plio-Pleistocene and of *G. campestris* of the Middle Pleistocene.

On the whole, the mammalian fauna from ThI-L is too poor to be confidently dated by biochronology, but it significantly differs from those of GH-CCC-unit 4, and also from that of Tighennif.

It is well-known that the rodent fauna of the northwestern African Pleistocene is highly endemic[53], but the large mammals have strong affinities with Eastern Africa[54], although temporal resolution is too low to estimate the dates of possible dispersal events. Most of the taxa present at ThI-L may have local ancestors, except perhaps *K. majus*, which must be of East African origin, because the earlier sites of Mansourah[55] and Aïn Hanech[56] yielded instead *K. olduvaiensis*, which belongs to another lineage[48].

3.2 Remarks on biochronology of Tighennif site (Algeria). The age of this site remains poorly supported. Geraads et al.[57] accepted an age close to 0.7 Ma, but it is becoming increasingly clear that this age was underestimated[58]. Pickford[59] estimated its age at 1.4 ±0.3 Ma, based upon his reassignment of the only suid from this locality to *Metridiochoerus andrewsi*, a species older than the South African *M. compactus*. This re-identification may be correct, but Pickford's biochronological conclusion is debatable, as the East African chronological range of *M. andrewsi* extends after 1 Ma. For instance, at Daka, a site well-dated at 1 Ma[60] the range of M3 length (63.7–88.1 mm, N = 4)[60] is almost the same as at Tighennif (64–83.5 mm, N = 6). We agree that Tighennif cannot be much younger than 1 Ma, but on the basis of bovids[61], an age older than 1.5 is very unlikely.

3.3 Taphonomy. The faunal assemblage is highly fragmented and the bone surfaces are badly preserved. The main alterations are natural abrasion striations, desquamation, fissuring and polishing (Supplementary Fig. S6). Trampling, weathering and water flowing could be the main post-depositional agents of alteration, notably in this context of open-air deposits in wetland environments. Besides, we notice a total absence of anthropogenic and carnivorous marks, possibly due to this scarcity of legible surfaces. However, crocodile tooth marks (scores and punctures) could be identified, some on hippo remains (Supplementary Fig. S7)[62-65].

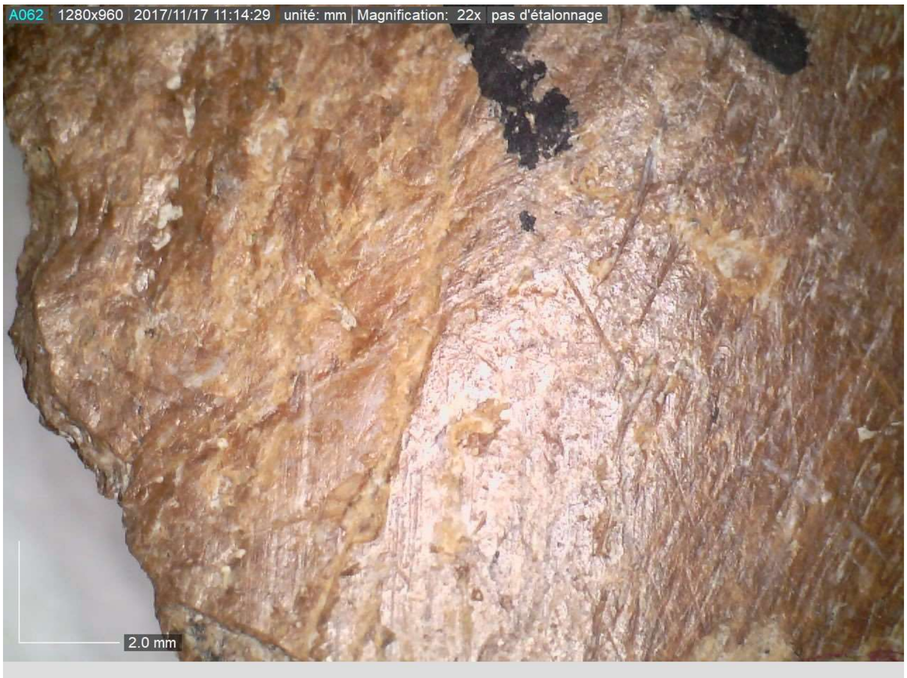

Fig. S6. Natural notches and abrasion striations on an unidentified bone fragment (ThI-L- R27-16). Digital microscope images C. Daujeard.

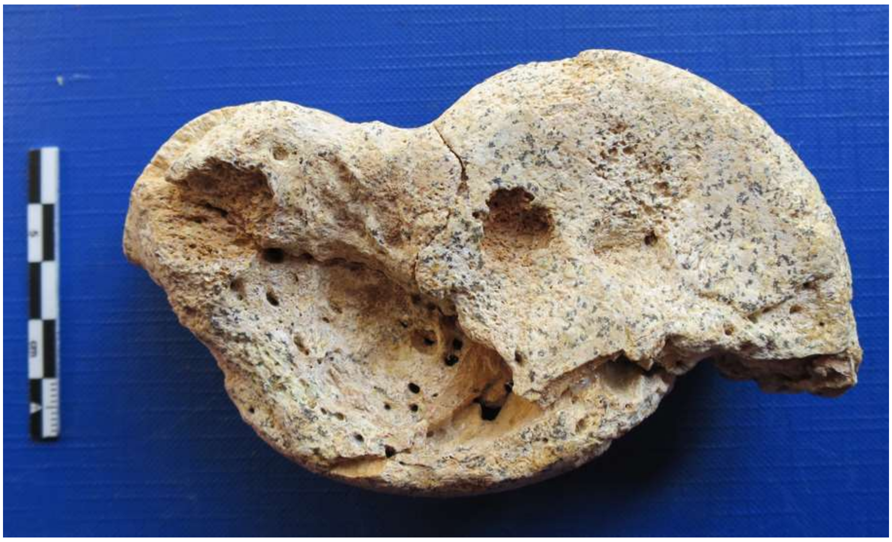

Fig. S7. Crocodile tooth punctures on a fragment of a hippo talus (ThI-L-5381). Photo C. Daujeard.

4. Magnetostratigraphy

Table S2. Thermal demagnetization data.

(1) Sample = sample name
(2) Temperature (°C)
(3) NRM intensity (A/m)
(4) Core Declination (°E)
(5) Core Inclination (°)
(6) Geographic Declination (°E)
(7) Geographic Inclination (°)

(1)	(2)	(3)	(4)	(5)	(6)	(7)
OH1 Bed 1 (samples S-)						
S-30	0	6,56E-05	183,24	19,319	66,2	83,8
S-30	100	4,96E-05	221,93	25,068	28,3	49,1
S-30	150	1,43E-04	51,746	43,545	132,2	-15,6
S-30	200	1,19E-04	55,921	43,793	133,9	-13
S-30	250	1,51E-04	52,949	32,858	141,9	-21,1
S-30	300	1,02E-04	326,5	42,447	69,1	-25,7
S-30	350	2,04E-04	17,071	-49,4	257,9	-51,9
S-30	400	2,74E-04	349,31	22,508	79,8	-52
S-30	425	7,63E-05	52,903	48,666	128,6	-11,8
S-30	450	1,78E-04	286,1	55,519	63	2,7
S-30	475	1,71E-04	309,72	40,968	58,4	-18
S-30	500	1,41E-04	331,67	6,9367	40,9	-54,9
S-30	550	1,35E-04	340,67	54,428	84,2	-19,6
S-30	575	1,42E-04	264,72	22,317	26,7	10
S-30	600	1,19E-04	207,77	55,087	74,4	43,6
S-41	0	3,01E-04	35,838	27,55	138,1	-41,8
S-41	100	2,97E-04	60,544	-41,63	227,7	-25,8
S-41	150	2,26E-04	54,766	-17,978	201,8	-35,3
S-41	200	1,70E-04	45,599	-4,5613	184,5	-44,6
S-41	250	1,80E-04	23,016	38,678	117,7	-40,5
S-41	300	1,70E-04	30,743	49,216	116,4	-28,6
S-41	350	1,94E-04	31,304	63,538	108	-16,6
S-41	400	1,69E-04	19,47	46,939	110	-34,3
S-41	425	1,87E-04	357,22	61,607	92,6	-22,4
S-41	450	1,67E-04	24,624	50,912	111,5	-29,3
S-41	475	5,47E-04	279,59	58,225	62,7	0,1
S-41	500	1,57E-04	325,01	76,54	86,3	-5
S-41	525	3,05E-04	291,17	27,127	34,4	-15,8
S-41	550	4,11E-04	311,52	20,784	34,8	-35,4
S-41	575	7,77E-05	359,13	40,142	93,1	-43,9
S-62	0	3,11E-04	347,66	12,248	53,6	-61,9
S-62	100	3,90E-04	32,46	6,6232	140,6	-52,3
S-62	150	7,09E-04	26,858	9,257	131,5	-55,3
S-62	200	3,99E-04	344,08	25,226	57,9	-48,7
S-62	250	3,44E-04	356,65	5,6678	69,7	-71
S-62	300	1,86E-04	348,98	32,252	67,1	-43,5
S-62	350	2,85E-04	346,12	15,447	53,8	-58,4
S-62	400	3,89E-04	324,2	39,272	49,1	-28
S-62	425	3,95E-04	297,81	27,775	24,9	-17,3
S-62	450	4,23E-04	337,44	2,8468	23,7	-62,6
S-62	475	6,18E-04	315,29	25,295	31,4	-32
S-62	500	3,86E-04	335,91	15,459	39,3	-52,9
S-62	525	4,04E-04	292,14	39,272	33,6	-8,2
S-62	550	1,51E-04	306,56	36,28	36,6	-19,6
S-62	575	6,75E-04	279,71	16,872	8,7	-5,3
S-77	0	2,52E-04	59,271	-33,954	211,8	-31
S-77	100	3,95E-04	56,917	-21,861	197,7	-34,3
S-77	150	2,86E-04	61,18	-7,2193	180,7	-29,5
S-77	200	3,59E-04	69,463	-16,321	191,7	-22,4
S-77	250	2,72E-04	69,775	-14,28	189,5	-21,9
S-77	300	4,33E-04	20,407	-20,972	209,6	-67,5
S-77	350	4,85E-04	60,719	-40,651	219,1	-28,6
S-77	400	3,53E-04	301,93	12,773	18,4	-28
S-77	425	1,87E-04	30,735	-76,693	260,7	-21,3
S-77	450	1,13E-04	23,278	3,8441	148,3	-63
S-77	475	1,99E-04	354,78	-20,545	292,9	-78,3
S-77	500	2,51E-04	119,93	62,875	113,3	22,2
S-77	550	2,44E-04	359,52	-12,324	279,4	-87,6
S-77	575	1,42E-04	50,843	-22,097	198,5	-39,9
S-91	0	1,59E-04	15,343	14,612	119	-62,7
S-91	100	3,23E-04	9,3129	-12,696	202,9	-79,7
S-91	150	3,09E-04	8,6568	-19,06	228,7	-76,1
S-91	200	1,46E-04	351,36	7,1082	55,1	-72,6
S-91	250	1,36E-04	333,62	24,072	46,9	-48,9
S-91	300	1,27E-04	356,13	58,186	82,8	-23,7
S-91	350	1,37E-04	333,03	38,372	58,5	-37,3
S-91	400	2,28E-04	328,99	43,139	58,8	-31,6
S-91	425	4,11E-04	310,72	40,492	46	-23,6
S-91	450	3,64E-04	298,06	19,769	20,5	-23
S-91	475	3,15E-04	298,78	-32,445	322,3	-28,5
S-91	500	5,34E-04	300,13	33,376	34,9	-19,8
S-91	525	3,85E-04	328,96	4,1706	15,4	-56,7
S-91	550	2,62E-04	322,23	28,499	41,6	-38,4
S-109	0	3,35E-04	91,107	-51,79	217,5	-21
S-109	100	3,40E-04	88,882	-52,889	218,2	-22,6
S-109	150	5,13E-04	91,045	-57,73	223,6	-22,9
S-109	200	3,09E-04	123,29	-50,04	226,5	-2,8
S-109	250	3,77E-04	99,302	-51,448	219,2	-16,2
S-109	300	3,88E-04	140,85	-18,506	215,3	30
S-109	350	4,93E-04	85,477	-50,017	214,5	-23,9
S-109	400	3,78E-04	173,99	-40,758	254,1	21
S-109	425	3,57E-05	126,94	23,422	161,8	42,3
S-109	450	3,09E-04	221,05	37,019	14,4	54,5
S-109	475	2,56E-04	19,312	14,473	105,2	-43,6
S-109	500	3,07E-04	247,35	-26,128	315,4	5,7
S-109	550	3,10E-04	205,36	-15,702	291,5	39,9
S-109	575	4,07E-04	333,27	38,202	57	-19,2
S-109	600	8,29E-05	61,39	-24,964	181	-35,6
S-134	0	2,18E-04	18,034	51,409	99	-21,8
S-134	100	2,61E-04	46,121	16,861	143,8	-34,4
S-134	150	3,09E-04	53,986	-5,7505	173,1	-36,2
S-134	200	1,49E-04	21,617	48,943	102,3	-23,2
S-134	250	2,69E-04	12,615	36,973	99,6	-36,7
S-134	300	2,72E-04	113,32	48,003	130,4	26,6
S-134	350	2,12E-04	17,223	-8,4214	157,6	-71,9
S-134	400	6,97E-04	356,57	10,742	79,3	-64
S-134	425	2,94E-04	5,0883	4,0973	102,2	-70,2
S-134	450	2,01E-04	310,06	26,568	37,3	-26,1
S-134	475	6,11E-04	298,51	32,897	37,4	-14,3
S-134	500	5,08E-04	301,83	36,221	41,8	-15
S-134	550	3,00E-04	287	22,539	23,5	-9,3
S-180	0	3,76E-04	65,695	-50,709	243	-32,1
S-180	100	3,76E-04	59,88	-36,361	225,8	-36,6
S-180	150	3,51E-04	66,83	-41,152	231,8	-31,4
S-180	200	2,90E-04	65,624	-43,352	234,4	-32,4
S-180	250	2,93E-04	72,072	-52,398	244,8	-28,1
S-180	300	2,93E-04	45	-48,294	243,6	-45,7
S-180	350	3,23E-04	62,103	-72,658	268,4	-29,1
S-180	400	2,49E-04	55,797	-51,562	245,2	-38,1
S-180	425	4,27E-04	6,6799	-38,217	268	-72,8
S-180	450	1,07E-04	12,653	-20,724	192,2	-78,2
S-180	475	2,71E-04	231,78	-67,918	303,3	-7,6
S-180	500	5,29E-04	257,35	-18,394	354,2	4,3
S-180	525	3,59E-04	235,24	4,6332	6,4	33,9
S-180	550	1,23E-04	80,518	-39,873	231,9	-20,9
OH1 Bed 2 (trench North Wall, samples N)						
N007	0	8,60E-05	187,25	-37,75	175,9	40,8
N007	100	8,59E-05	214,85	-38,8	199,4	30,5
N007	150	1,03E-04	228,52	-41,9	204,9	20,9
N007	200	9,14E-05	219,6	-39,72	201,8	27,4
N007	225	9,54E-05	225,09	-30,91	212,7	29,8
N007	250	9,18E-05	225,37	-26,04	217,5	32,4
N007	275	7,84E-05	225,12	-27,27	216,2	31,9
N007	300	7,39E-05	226,72	-30,56	214	28,8
N007	325	7,34E-05	222,15	-23,7	217,8	36,1
N007	350	7,08E-05	215,72	-20,76	216,2	42,7
N007	375	7,64E-05	231,75	-17,03	230,2	31,7
N007	400	7,98E-05	229,81	-8,79	238,5	36,6
N007	425	7,69E-05	230,58	-2,75	246	37,8
N007	450	6,10E-05	230,6	-7,56	240,3	36,3
N007	475	6,99E-05	225,86	-3,89	242,7	42
N007	500	8,25E-05	233,14	-1,02	248,9	35,8
N007	525	6,70E-05	237,11	-0,55	250,6	32,1
N007	550	7,16E-05	255,12	2,97	258,4	15,2
N007	575	8,30E-05	257,25	3,66	259,5	13,2
N007	600	5,70E-05	246,14	9	263,2	25
N007	625	4,72E-05	259,13	-19,09	237,3	6,5
N007	650	6,81E-05	238,39	-9,98	240,5	28,3
N007	675	9,95E-05	264,65	17,1	274,2	8,3
N011	0	2,06E-05	207,17	30,22	294,7	60,2
N011	100	9,20E-06	277,26	-6,7	252,5	-8,6
N011	150	1,64E-05	309,26	-18,37	245	-41,2
N011	200	1,54E-05	291,16	-23,07	238	-24,6
N011	225	1,58E-05	303,19	-12,17	252	-34,8
N011	250	2,18E-05	291,05	-14,88	246,9	-23,5
N011	275	1,91E-05	329,58	-15,09	251,3	-60,6
N011	300	3,23E-05	298,47	-12	251,4	-30,2
N011	325	3,68E-05	295,09	-19,14	242,8	-27,9
N011	350	3,76E-05	302,09	-14,48	249,1	-34
N011	375	3,75E-05	306,94	-16,46	247,3	-38,9
N011	400	3,73E-05	301,98	-19,92	242,5	-34,4
N011	425	3,69E-05	288,17	-27,7	232,7	-22,4
N011	450	4,75E-05	300,22	-36,77	222,4	-32,4
N011	475	4,12E-05	321,42	-45,32	205,5	-44,9
N011	500	3,58E-05	305,58	-31,03	228,7	-37,5
N011	525	2,98E-05	300,08	-29,7	230,7	-32,8
N011	550	4,53E-05	304,58	-27,1	233,7	-36,9
N011	575	5,45E-05	319,77	-12,84	254,1	-50,9
N011	600	2,31E-05	295,57	-16,39	245,9	-28
N011	625	2,84E-05	66,92	-22,77	96	-26,4
N011	650	1,38E-05	85,66	-26,42	102,2	-10
N011	675	4,34E-05	270,69	55	311,5	11
N015	0	1,00E-04	89,81	-62,28	141,8	-12,5
N015	100	1,39E-04	72,04	-71,63	151,8	-18,9
N015	150	1,37E-04	52,7	-74,59	157	-22,9
N015	200	1,27E-04	68,21	-75,94	156,5	-18,8
N015	225	1,23E-04	61,08	-73,7	155	-21,3
N015	250	1,25E-04	65,4	-68,15	148,9	-22
N015	275	1,21E-04	66,48	-64,49	145	-22,6
N015	300	1,17E-04	67,26	-63,39	143,7	-22,6
N015	325	1,32E-04	65,51	-57,53	137,7	-24,8
N015	350	1,33E-04	69,19	-61,99	142	-22,1
N015	375	1,22E-04	59,49	-56,52	137,6	-28,3
N015	400	1,34E-04	66,25	-63,74	144,2	-22,9
N015	425	1,28E-04	60,43	-63,7	145	-25,4
N015	450	1,25E-04	58,92	-64,89	146,5	-25,6
N015	475	1,32E-04	57,19	-61,17	143	-27,7
N015	500	1,16E-04	32,8	-61,82	151,7	-36,8
N015	525	1,20E-04	47,43	-65,71	149,9	-29,4
N015	550	1,22E-04	43,96	-60,24	145,8	-33,8
N015	575	9,52E-05	54,59	-60,66	143,1	-29,1
N015	600	1,23E-04	89,8	-63,48	143,1	-12,6
N015	625	1,12E-04	63,88	-74,42	155,4	-20,4
N015	650	1,18E-04	126,73	-61,4	147,7	3,7
N015	675	1,21E-04	90,74	-43,91	123,4	-9,1
N019	0	1,14E-04	348,52	-69,98	171,9	-32,6
N019	100	1,46E-04	334,82	-72,51	175,7	-28,7
N019	150	1,65E-04	332,56	-66,49	180	-33,5
N019	200	1,78E-04	326,59	-58,24	188,9	-38,3
N019	225	1,84E-04	319,63	-58,87	191,5	-35,2
N019	250	1,90E-04	319,12	-56,58	193,9	-36,4
N019	275	1,91E-04	315,71	-56,86	195	-34,7
N019	300	2,05E-04	319,54	-49,42	201,2	-40,8
N019	325	1,90E-04	318,13	-51,71	199,3	-38,8
N019	350	1,89E-04	317,38	-52,88	198,4	-37,7
N019	375	1,85E-04	317,36	-52,04	199,3	-38,2
N019	400	1,86E-04	309,87	-53,71	200,3	-33,4
N019	425	1,79E-04	310,77	-51,96	201,9	-34,7
N019	450	1,73E-04	307,04	-45,52	210,3	-34,9
N019	475	2,02E-04	309,42	-48,11	206,6	-35,5
N019	500	2,01E-04	313,1	-45,05	208,9	-39
N019	525	1,78E-04	311,09	-41,37	214	-39
N019	550	1,88E-04	314,27	-46,67	206,6	-39,1
N019	575	1,88E-04	316,6	-36,7	218	-44,6
N019	600	1,66E-04	315,85	-45,97	206,7	-40,4
N019	625	1,51E-04	308,47	-45,88	209,4	-35,7
N019	650	1,67E-04	314,92	-36,51	218,8	-43,4
N019	675	1,75E-04	304,79	-35,35	222,9	-35,7

Table S2. Thermal demagnetization data.

(1) Sample = sample name
(2) Temperature (°C)
(3) NRM intensity (A/m)
(4) Core Declination (°E)
(5) Core Inclination (°)
(6) Geographic Declination (°E)
(7) Geographic Inclination (°)

N023	0	4,97E-05	265,99	26,3	286,3	9,3
N023	100	6,16E-05	297,34	11,49	280	-23,2
N023	150	8,03E-05	309,76	-4,55	266,2	-39,7
N023	200	8,94E-05	311,06	2,41	275,3	-39
N023	225	9,45E-05	303,91	4,2	274,6	-31,7
N023	250	1,04E-04	299,21	2,54	271,2	-27,7
N023	275	1,13E-04	301,62	10,98	281,1	-27,3
N023	300	1,22E-04	301,27	13,19	283,3	-26,2
N023	325	1,10E-04	303,15	17,88	288,9	-26
N023	350	1,11E-04	296,47	4,68	272,7	-24,5
N023	375	1,19E-04	303,14	10,93	281,7	-28,7
N023	400	1,19E-04	296,51	15,16	283,4	-21,2
N023	425	1,34E-04	295,17	18,96	286,7	-18,6
N023	450	1,45E-04	300,46	17,6	287,4	-23,8
N023	475	1,43E-04	304,41	17,06	288,6	-27,4
N023	500	1,41E-04	290,14	15,11	281,2	-15,4
N023	525	1,56E-04	295,42	16,12	284	-19,8
N023	550	1,55E-04	291,76	17,34	283,9	-16,1
N023	575	1,31E-04	301	19,01	289,1	-23,7
N023	600	1,42E-04	302,94	18,23	289,1	-25,6
N023	625	1,55E-04	298,66	22,96	292	-20
N023	650	1,37E-04	317,18	28,4	306,8	-31,4
N023	675	1,16E-04	310,81	19,15	294	-31,9
N069	0	2,50E-04	87,06	24,07	149,5	4,7
N069	100	2,27E-04	83,14	7,58	163,9	-4,1
N069	150	2,09E-04	78,42	1,42	168,3	-10,6
N069	200	1,87E-04	82,32	-12,98	183,5	-11,1
N069	225	1,72E-04	77,4	-18,37	187,6	-17,1
N069	250	1,72E-04	84,77	-23,37	194,3	-11,7
N069	275	1,70E-04	81,64	-23,8	194	-14,5
N069	300	1,54E-04	83,8	-24,54	195,2	-12,8
N069	325	1,52E-04	91,98	-31,5	204	-7,7
N069	350	1,52E-04	89,04	-32,92	204,7	-10,4
N069	375	1,52E-04	93,77	-27,99	201,1	-5,2
N069	400	1,52E-04	98	-29,62	203,8	-2,2
N069	425	1,43E-04	99,92	-33,72	208,2	-2
N069	450	1,44E-04	95,87	-37,98	211,2	-6,5
N069	475	1,36E-04	90,46	-41,63	213,6	-11,5
N069	500	1,35E-04	117,39	-46,49	225,5	4,4
N069	525	1,66E-04	95,78	-45,73	218,6	-8,9
N069	550	1,75E-04	88,91	-54,66	226,5	-15,2
N069	575	1,55E-04	104,81	-62,44	236,3	-9,3
N069	600	1,48E-04	114,22	-62,53	238,3	-5,4
N069	625	1,33E-04	117,69	-67,53	243,4	-6,7
N069	650	1,14E-04	102,87	-68,45	241,8	-12,1
N069	675	9,00E-05	155,72	-61,74	251,9	7,9
N072	0	1,39E-04	72,66	66,39	103	8,8
N072	100	1,20E-04	57,76	60,28	105,1	0,1
N072	150	8,57E-05	68,33	50	117	-0,2
N072	200	5,92E-05	41,23	42,59	111,2	-19,4
N072	225	5,19E-05	39,72	41,58	111,1	-20,9
N072	250	5,53E-05	18,02	54,19	91,2	-17,2
N072	275	4,10E-05	3,29	45,13	82,9	-27,8
N072	300	3,11E-05	332,73	63,97	68,6	-6,3
N072	325	3,58E-05	327,5	59,15	64,1	-9,4
N072	350	3,60E-05	312,25	52,2	52,9	-9,4
N072	375	3,54E-05	287,97	33,97	28	-4,7
N072	400	3,84E-05	289,06	33,84	28,2	-5,5
N072	425	3,40E-05	278,45	30,6	21,9	1,6
N072	450	4,59E-05	265,62	10,71	359,3	7,2
N072	475	3,79E-05	284,89	53,32	44,9	5
N072	500	2,47E-05	236,55	-3,32	335,7	30,6
N072	525	3,73E-05	255,04	-21,76	325	6,9
N072	550	3,04E-05	291,81	34,46	29,8	-7,3
N072	575	3,53E-05	285,04	24,19	17,9	-6,1
N072	600	3,84E-05	289,96	26,34	21,7	-9,4
N072	625	3,17E-05	260,45	4,34	351,7	10,4
N072	650	3,55E-05	1,82	36,79	82,1	-36,2
N072	675	1,97E-05	235,52	9,58	350	35,6
N074	0	1,17E-04	60,18	2,45	158,7	-28,1
N074	100	1,13E-04	44,21	-9,02	167,7	-46,5
N074	150	1,30E-04	31,93	-22,64	189,9	-58,6
N074	200	1,38E-04	70,58	-0,11	164,5	-18,8
N074	225	9,96E-05	22,34	-38,67	224,8	-58,4
N074	250	9,19E-05	23,35	-43,77	230	-54,2
N074	275	8,57E-05	13,81	-40,88	237,8	-60,5
N074	300	8,03E-05	11,37	-47,22	245,6	-55,5
N074	325	6,85E-05	8,09	-39,21	245	-63,8
N074	350	6,36E-05	1,75	-42,85	256,6	-61,1
N074	375	5,75E-05	327,61	-40,82	299,5	-51,1
N074	400	5,32E-05	355,82	-42,72	265,6	-61,1
N074	425	4,80E-05	324,13	-17,72	338,3	-55,3
N074	450	7,05E-05	306,44	-26,96	325,8	-38,6
N074	475	5,32E-05	324,81	-15,43	342,3	-56
N074	500	6,81E-05	315,54	-37,91	309,5	-44
N074	525	8,12E-05	327,53	-42,9	296,9	-49,8
N074	550	9,19E-05	312,36	-63,57	281,7	-30,5
N074	575	7,57E-05	293,2	-64,26	284,9	-22,6
N074	600	8,44E-05	298,23	-36,2	315,2	-30,9
N074	625	4,14E-05	354,38	-75,74	260,9	-28,2
N074	650	3,76E-05	250,95	-78,36	270,4	-10
N074	675	4,89E-05	323,49	-10,78	350,2	-54,2
N077	0	1,59E-04	83,88	16,71	150,6	-1,7
N077	100	1,71E-04	81,19	17,61	149	-3,9
N077	150	1,63E-04	72,45	5,72	158,2	-15,5
N077	200	1,10E-04	30,15	-33,58	209,2	-56,4
N077	225	1,22E-04	69,45	-0,57	163,7	-20,1
N077	250	1,14E-04	70	0,57	162,7	-19,2
N077	275	1,05E-04	68,47	-11,93	175,4	-23,5
N077	300	9,75E-05	60,68	-15,03	177,5	-31,4
N077	325	8,78E-05	57,61	-10,56	171,8	-33,7
N077	350	7,83E-05	61,34	-2,22	163,2	-28,3
N077	375	6,44E-05	69	-9	172,4	-22,4
N077	400	5,30E-05	72,19	0,06	163,8	-17,2
N077	425	4,54E-05	67,86	-27,96	192,9	-25,9
N077	450	4,23E-05	68,87	-12,32	175,9	-23,2
N077	475	3,51E-05	99,85	-30,5	200,2	1,2
N077	500	4,26E-05	73,15	1,42	162,7	-16
N077	525	1,15E-05	115,64	-12,99	188,3	20,8
N077	550	2,11E-05	61,93	-57,11	225,8	-26,8
N077	575	3,64E-05	211,38	-58,43	274,5	13,2
N077	600	3,39E-05	159,62	-52,67	245,2	21,1
N077	625	7,55E-05	297,93	-72,25	275,1	-21,7
N077	650	4,78E-05	238,3	-49	292,7	8,7
N077	675	4,81E-05	235,18	-50,61	290,2	9,5
N082	0	1,55E-04	71,86	11,63	112,7	-13,7
N082	100	1,50E-04	75,02	0,15	124,9	-14,3
N082	150	1,42E-04	60,16	-10,71	132,2	-31,4
N082	200	1,29E-04	68,9	-11,4	135,1	-23,2
N082	225	1,13E-04	66,75	-4,43	127,1	-23,6
N082	250	9,58E-05	63,93	-8,15	130,3	-27,2
N082	275	8,25E-05	73,59	-8,28	133	-18
N082	300	7,37E-05	84,28	-24,43	151,6	-11,6
N082	325	7,35E-05	63,92	-24,59	148,8	-29,9
N082	350	6,10E-05	71,77	-28,9	154,4	-23,4
N082	375	5,00E-05	95,05	-19,13	149	-0,6
N082	400	6,26E-06	257,42	-9,15	296,9	9,4
N082	425	4,08E-05	62,47	-17,14	140,1	-30,4
N082	450	4,71E-05	50,86	-11,75	131,2	-40,6
N082	475	4,03E-05	86,01	-3,51	131,6	-4,8
N082	500	3,86E-05	80,85	-34,47	161,2	-16,4
N082	525	2,32E-05	65,04	-51,35	179,4	-27,9
N082	550	4,63E-05	68,82	-4,77	128,1	-21,7
N082	575	1,03E-05	46,91	-14,83	134,5	-44,9
N082	600	3,52E-05	178,41	-50,18	218,2	23,8
N082	625	3,85E-05	129,21	-28,04	170,8	24
N082	650	6,50E-05	102,46	-37,54	168,5	-0,2
N082	675	5,67E-05	96,58	-32,52	162,3	-3,2

OH1 Bed 2 (trench South Wall, samples S004–S314)

S004	0	1,72E-04	330,87	0,15	78,6	-60,9
S004	100	1,70E-04	326,67	-4,75	69,7	-56,4
S004	150	1,88E-04	329,76	-20,6	41,5	-54
S004	200	1,80E-04	331,39	-18,48	43,4	-56,4
S004	225	1,74E-04	335,48	-17,35	41,3	-60,3
S004	250	1,70E-04	338,63	-23,02	28,9	-59
S004	275	1,57E-04	337,09	-21,12	33,5	-59,2
S004	300	1,57E-04	335,34	-26,61	28,1	-54,3
S004	325	1,44E-04	328,73	-25,22	36,1	-50,6
S004	350	1,42E-04	326,78	-27,51	34,7	-47,9
S004	375	1,27E-04	321,21	-20,72	47,2	-46,8
S004	400	1,33E-04	327,91	-12,58	55,5	-55,8
S004	425	1,33E-04	326,97	-7,92	64	-56,1
S004	450	1,39E-04	324,08	-8,97	63,2	-53,1
S004	475	1,30E-04	318,47	-10,8	62,2	-47,3
S004	500	1,10E-04	306,29	-10,25	65,6	-35,6
S004	525	1,28E-04	303,07	-16,39	58,9	-31,6
S004	550	1,19E-04	322,48	-2,35	74,4	-52,4
S004	575	1,19E-04	323,24	-22,69	43,3	-47,7
S004	600	1,48E-04	327,84	-20,92	42,6	-52,3
S004	625	1,38E-04	327,95	-30,92	29,8	-46,6
S004	650	1,42E-04	335,51	-51,45	6,6	-34,6
S004	675	1,51E-04	317,09	-43,74	23,7	-31,9
S010	0	2,76E-04	316,18	76,46	161,8	-9,7
S010	100	2,60E-04	293,42	74,97	157,4	-5,9
S010	150	2,39E-04	302,09	72,58	156,4	-9,2
S010	200	2,22E-04	321,24	70,78	159	-14,9
S010	225	2,14E-04	320,01	70,92	158,8	-14,5
S010	250	2,16E-04	322,53	71,67	159,9	-14,5
S010	275	1,97E-04	325,54	70,88	160,2	-15,7
S010	300	1,89E-04	330,75	69,85	161,1	-17,5
S010	325	1,83E-04	342,74	70,43	165,3	-18,7
S010	350	1,73E-04	352,25	65,85	167,8	-23,9
S010	375	1,60E-04	354,75	67,48	169,1	-22,4
S010	400	1,55E-04	348,35	71,22	167,4	-18,4
S010	425	1,42E-04	351,65	69,87	168,2	-19,9
S010	450	1,23E-04	346,74	66,33	165,5	-23
S010	475	1,08E-04	345,84	63,25	164,3	-25,9
S010	500	1,08E-04	339,23	67,08	162,8	-21,4
S010	525	1,15E-04	330,98	63,95	157,9	-22,6
S010	550	8,64E-05	339,05	63	161	-25,1
S010	575	7,37E-05	338,18	63,64	160,8	-24,3
S010	600	6,87E-05	301,97	87,28	169	-1,4
S010	625	5,83E-05	321,69	77,78	163,6	-9,6
S010	650	5,52E-05	232,22	68,98	154,4	12,7
S010	675	5,29E-05	290,11	71,74	154,1	-6,2
S040	0	2,72E-04	341,23	71,87	142,3	-17,1
S040	100	2,61E-04	347,22	67,98	143,2	-21,4
S040	150	2,71E-04	350,56	64,81	143,9	-24,8
S040	200	2,61E-04	344,08	61,32	139,8	-27,5
S040	225	2,49E-04	347,52	60,92	141,4	-28,3
S040	250	2,44E-04	341,12	58,94	137,3	-29,2
S040	275	2,34E-04	340,97	64,2	139,3	-24,3
S040	300	2,08E-04	341,64	63,26	139,3	-25,3
S040	325	1,92E-04	334,95	58,37	133,7	-28,4
S040	350	1,81E-04	337,46	59,99	135,8	-27,5
S040	375	1,71E-04	328,94	58,38	130,7	-26,7
S040	400	1,49E-04	332,5	57,28	131,8	-28,7
S040	425	1,37E-04	332,86	56,12	131,3	-29,7
S040	450	1,34E-04	321,08	54,56	124,2	-26,8
S040	475	1,24E-04	315,53	54,48	121,7	-24,5
S040	500	1,00E-04	311,36	42,94	109,4	-28,9
S040	525	1,29E-04	303,88	45,61	109,2	-23
S040	550	1,61E-04	312,2	42,67	109,5	-29,6
S040	575	1,31E-04	315,34	38,29	106,6	-33,9
S040	600	1,23E-04	305,15	34,43	98,3	-28,4
S040	625	9,86E-05	303,34	31,17	94,2	-28,1
S040	650	1,14E-04	299,53	29,1	90,9	-25,5
S040	675	1,04E-04	301,44	38,57	101,3	-24,1
S054	0	1,34E-04	270,27	25,39	98,7	-0,2
S054	100	1,26E-04	274,21	23,15	96,5	-3,9
S054	150	1,18E-04	281,43	12,48	86	-11,2

Table S2. Thermal demagnetization data.

(1) Sample = sample name
(2) Temperature (°C)
(3) NRM intensity (A/m)
(4) Core Declination (°E)
(5) Core Inclination (°)
(6) Geographic Declination (°E)
(7) Geographic Inclination (°)

S054	200	1,20E-04	279,57	4,35	77,7	-9,5
S054	225	1,19E-04	279,17	4,7	78	-9,1
S054	250	1,15E-04	275,91	1	74,3	-5,9
S054	275	1,18E-04	274,44	0,24	73,5	-4,4
S054	300	1,11E-04	273,53	1,76	75	-3,5
S054	325	1,04E-04	272,52	-4,3	69	-2,5
S054	350	1,09E-04	273,8	-6,89	66,4	-3,8
S054	375	1,09E-04	267,85	-12,77	60,5	2,1
S054	400	1,07E-04	266,54	-12,16	61,1	3,4
S054	425	1,14E-04	264,99	-16,22	57	4,8
S054	450	9,75E-05	279,53	-33,57	39,3	-7,9
S054	475	1,19E-04	271,53	-34,12	39,2	-1,3
S054	500	7,58E-05	276,28	-29,8	43,3	-5,4
S054	525	1,29E-04	259,19	-19,92	53	10,2
S054	550	6,82E-05	247,45	-28,8	42,5	19,6
S054	575	6,65E-05	256,12	-35,61	36,9	11,2
S054	600	9,09E-05	204,97	-44,31	6,7	40,4
S054	625	8,10E-05	241,92	-75,97	355,7	6,6
S054	650	8,66E-05	242,88	-37,06	33	21,3
S054	675	8,71E-05	220,65	-33,31	28	39,4
S058	0	1,57E-04	55,19	85,55	173,9	-2,5
S058	100	1,48E-04	7,45	77,98	171,9	-11,9
S058	150	1,24E-04	353,32	66,35	167,4	-23,5
S058	200	1,24E-04	3,99	68,68	171,8	-21,3
S058	225	1,25E-04	10,64	71,02	173,9	-18,6
S058	250	1,27E-04	15,91	70,79	175,7	-18,4
S058	275	1,22E-04	2,65	76,04	170,9	-13,9
S058	300	1,21E-04	12,5	74,15	173,8	-15,5
S058	325	1,15E-04	21,49	70,38	177,7	-18,2
S058	350	1,22E-04	354,55	71,63	168,5	-18,3
S058	375	1,22E-04	354,83	71,05	168,5	-18,9
S058	400	1,04E-04	356,98	68,41	169,1	-21,6
S058	425	9,00E-05	318,99	74,17	159,7	-11,9
S058	450	7,65E-05	306,95	58,2	143,9	-18,5
S058	475	6,71E-05	284,6	71,46	152,3	-4,6
S058	500	3,21E-05	208,82	74,92	162,9	13,2
S058	525	6,16E-05	344,47	30,7	146	-55,9
S058	550	5,62E-05	331,81	45,85	145,6	-37,9
S058	575	5,54E-05	273,68	52,43	132,8	-2,2
S058	600	3,76E-05	41,95	28,03	221,8	-41
S058	625	3,38E-05	98,4	22,33	237,7	7,8
S058	650	3,73E-05	336,41	50,15	151,8	-36
S058	675	6,85E-05	39,89	26,27	222,7	-43,5
S061	0	1,01E-04	193,06	73,85	166,5	15,7
S061	100	9,71E-05	153,78	79,7	174,9	9,2
S061	150	7,89E-05	188,46	82,89	169,2	7
S061	200	7,89E-05	225,47	78,3	161,9	8,2
S061	225	7,88E-05	210,16	75,14	162,7	12,8
S061	250	8,28E-05	220,22	78,93	163,1	8,4
S061	275	7,39E-05	208,36	78,09	164,6	10,5
S061	300	6,66E-05	171,31	73,58	172,8	16,2
S061	325	6,00E-05	163,43	71,08	175,9	18,1
S061	350	6,64E-05	166,69	64,44	176,6	24,8
S061	375	6,63E-05	178,53	52,96	171,4	37
S061	400	5,50E-05	176,82	36,65	174,5	53,2
S061	425	6,25E-05	172,91	42,02	178,1	47,5
S061	450	5,60E-05	169,33	41,62	182,1	47,3
S061	475	5,56E-05	181,59	15,85	164,7	74,1
S061	500	4,24E-05	151,79	17,67	226,3	57,1
S061	525	6,31E-05	169,74	10,49	214,2	75,4
S061	550	5,90E-05	170,4	36,28	183,1	52,6
S061	575	4,27E-05	172,47	52,6	176	37
S061	600	5,44E-05	153,17	30,11	208,2	50,5
S061	625	5,61E-05	148,02	40,4	202,2	40,2
S061	650	4,73E-05	130,67	21,96	232,3	37,2
S061	675	4,86E-05	155,63	30,43	205,4	51,8
S110	0	2,30E-04	54,2	83,12	170,9	7,9
S110	100	2,43E-04	60,26	78,62	175,2	6,2
S110	150	2,19E-04	61,65	73,97	179,4	4,1
S110	200	2,10E-04	44,91	73,65	176,7	0,3
S110	225	2,01E-04	45,34	74,29	176,4	0,8
S110	250	1,91E-04	51,87	75,18	176,9	2,7
S110	275	1,83E-04	63,87	76,37	177,6	5,8
S110	300	1,79E-04	77,64	72,79	182,2	7,9
S110	325	1,85E-04	63,16	72,3	181,1	3,7
S110	350	1,78E-04	50,23	70,93	179,8	-0,5
S110	375	1,69E-04	52,59	70,69	180,5	0
S110	400	1,57E-04	57,32	71,77	180,6	1,8
S110	425	1,46E-04	57,1	63,86	187	-2,7
S110	450	1,38E-04	46,47	68,94	180,4	-2,8
S110	475	1,52E-04	49,39	65,39	183,8	-4,4
S110	500	1,34E-04	76,68	63,83	190,8	5
S110	525	1,66E-04	54,54	66,19	184,5	-2,2
S110	550	1,55E-04	72,91	68,43	185,9	5
S110	575	1,42E-04	49,42	65,55	183,7	-4,2
S110	600	1,21E-04	67,47	52,63	199,5	-3,6
S110	625	8,12E-05	24,24	49,95	182,2	-24,5
S110	650	6,97E-05	146,15	47,34	197,4	44,7
S110	675	9,11E-05	36,9	32,07	202,9	-33,5
S113	0	1,40E-04	177,93	49,39	173,6	53,6
S113	100	1,12E-04	189,3	51,77	162,2	50,5
S113	150	9,47E-05	175,63	55,83	174,9	47
S113	200	8,05E-05	175,75	50,88	175,6	52
S113	225	7,03E-05	171,77	48,27	180,6	54,1
S113	250	7,29E-05	166,58	51,85	184,1	49,7
S113	275	6,66E-05	172,17	50,03	179,5	52,4
S113	300	5,84E-05	164,94	40,23	194,5	59,7
S113	325	5,26E-05	159,24	28,1	220,1	65,5
S113	350	5,42E-05	162,78	30,32	211,1	66,5
S113	375	5,08E-05	162,02	20,73	234,7	71,2
S113	400	5,38E-05	160,07	14,66	254	70,6
S113	425	5,04E-05	152,14	15,13	253,4	62,9
S113	450	4,91E-05	151,81	3,9	276,9	60,7
S113	475	4,97E-05	147,65	13,91	255,8	58,5
S113	500	5,43E-05	157,71	10,44	265,6	68
S113	525	4,35E-05	167,9	-1,5	310,8	71,2
S113	550	3,97E-05	149,25	9,14	265,4	59,6
S113	575	3,23E-05	156,16	-32,35	325,1	39,2
S113	600	2,75E-05	197,98	-9,23	30,9	61,5
S113	625	6,88E-05	166,43	-36,58	337,3	38,8
S113	650	3,76E-05	358,77	-59,63	352,1	-43,4
S113	675	5,80E-05	133,57	-26,24	302,5	30,2
S117	0	2,44E-04	141,31	67,92	182,4	25,8
S117	100	2,08E-04	143,51	66,2	183	27,6
S117	150	1,80E-04	128,59	67,58	186,1	22,3
S117	200	1,50E-04	131,45	64,14	188,5	25,2
S117	225	1,27E-04	126,97	65,86	188	22,7
S117	250	1,09E-04	123,91	57,61	196,7	25,3
S117	275	9,75E-05	117,88	51,92	203,9	24,1
S117	300	9,91E-05	124,6	53,76	200,5	27,2
S117	325	9,49E-05	120,26	47,42	208,2	26,9
S117	350	7,91E-05	122,69	46,7	208,4	28,7
S117	375	7,54E-05	111,95	39,83	217,8	22,6
S117	400	6,94E-05	117,11	40,14	216,7	26,4
S117	425	6,96E-05	118,91	36,91	220	28,4
S117	450	7,03E-05	113,29	30,81	227,4	24,6
S117	475	5,89E-05	112,2	39,87	217,7	22,7
S117	500	5,31E-05	152,87	35,25	206,5	53,9
S117	525	5,60E-05	113,77	19,8	239,6	25,3
S117	550	3,89E-05	142,33	15,55	243,4	52,7
S117	575	6,09E-05	155,38	29,09	212,9	59,4
S117	600	2,94E-05	152,12	29,56	215,2	56,8
S117	625	1,17E-04	77,84	4,56	250,8	-11,2
S117	650	7,89E-05	56,75	51,38	199,6	-12,5
S117	675	2,85E-05	231,42	13,26	87,1	39,4
S124	0	1,23E-04	256,76	84,76	163,1	10,2
S124	100	1,01E-04	302,12	80,77	160,5	4
S124	150	8,65E-05	337,65	73,3	162	-6,5
S124	200	7,54E-05	337,34	70,36	160,7	-9,1
S124	225	6,98E-05	345,01	66,21	162,1	-14
S124	250	6,82E-05	348,17	66,37	163,4	-14,1
S124	275	5,92E-05	353,09	65,21	165,3	-15,6
S124	300	5,31E-05	352,5	61,18	164,5	-19,6
S124	325	4,59E-05	355,37	54,14	165,2	-26,7
S124	350	4,17E-05	340,54	48,01	153,3	-30,4
S124	375	3,94E-05	334,5	48,73	149,5	-28,1
S124	400	4,02E-05	342,29	38,29	150,1	-39,9
S124	425	3,48E-05	341,43	32,73	146,2	-44,7
S124	450	4,20E-05	320,12	55,6	146	-17,4
S124	475	3,20E-05	285,99	72,49	151,4	3,9
S124	500	2,93E-05	311,71	46,09	134,8	-20,1
S124	525	3,03E-05	335,46	62,94	157	-15,6
S124	550	3,31E-05	272,23	30,11	108,4	2,6
S124	575	4,01E-05	337,99	27,59	138,7	-47,7
S124	600	4,33E-05	323,98	10,64	106,2	-49,1
S124	625	4,37E-05	52,22	5,2	245,1	-36
S124	650	5,05E-05	329,34	8,86	107,8	-54,6
S124	675	2,44E-05	58,18	-38,81	298,2	-30,3
S131	0	1,57E-04	219,42	74,67	160,1	18,7
S131	100	1,25E-04	237,62	78,16	160	13,2
S131	150	1,09E-04	282,97	72,28	153	2,8
S131	200	9,21E-05	297,21	68,24	151	-3,2
S131	225	8,38E-05	298,39	67,52	150,6	-3,9
S131	250	7,15E-05	287,69	72,17	153,3	1,4
S131	275	6,30E-05	282,77	73,76	154,4	3,2
S131	300	6,21E-05	295,59	78,42	159,8	1,9
S131	325	5,30E-05	268,42	71,91	152,1	7,1
S131	350	5,57E-05	256,49	76,38	156,8	10
S131	375	5,34E-05	279,02	75,48	155,9	4,5
S131	400	4,57E-05	242,64	76,64	158,1	12,9
S131	425	3,55E-05	258,38	70,94	151,3	10,4
S131	450	3,69E-05	221,8	53,63	142,3	32,5
S131	475	3,26E-05	206,02	49,74	147,8	42
S131	500	4,40E-05	167,57	56,66	179,1	39,4
S131	525	3,61E-05	173,38	47,55	177,1	49,1
S131	550	4,45E-05	127,85	43,61	212,5	31,7
S131	575	3,79E-05	152,23	42,19	200,8	47,1
S131	600	4,26E-05	125,64	-26,06	294,7	27,8
S131	625	9,08E-05	88,48	5,22	254,9	-0,9
S131	650	2,29E-05	75,65	-58,49	318,9	-13,4
S131	675	1,42E-05	354,89	18,3	159,1	-64,2
S135	0	1,08E-04	226,04	64,82	147,7	23,8
S135	100	1,09E-04	246,22	68,8	147,3	15
S135	150	9,37E-05	257,5	68,53	145,9	11,1
S135	200	7,80E-05	270,32	63,74	140,9	6,1
S135	225	6,73E-05	269,8	66,84	144	6,5
S135	250	6,33E-05	264,04	59,57	136,6	9
S135	275	5,74E-05	262,78	59,56	136,6	9,7
S135	300	5,09E-05	259,84	54,1	131,2	11,6
S135	325	4,92E-05	263,63	51,45	128,4	9,4
S135	350	4,60E-05	267,88	53,13	130,1	6,9
S135	375	5,10E-05	269,1	47,19	124,2	5,7
S135	400	4,53E-05	285,13	43,26	122,3	-6
S135	425	4,61E-05	292,04	40,79	121,5	-11,7
S135	450	4,51E-05	290,42	30,22	110,8	-13,8
S135	475	3,29E-05	301,54	25,07	109,1	-24,7
S135	500	3,98E-05	302,77	45,54	129,3	-16,8
S135	525	2,73E-05	314,25	43,05	132	-25
S135	550	2,54E-05	312,62	35,27	124,1	-28,6
S135	575	4,32E-05	285,7	24,87	104,4	-11,1
S135	600	1,67E-05	157,74	58,36	181,5	35,8
S135	625	5,39E-05	52,15	-1,94	254,3	-37,8
S135	650	3,08E-05	265,35	-24,97	51,9	1,2
S135	675	2,62E-05	272,38	8,94	86,4	-1,2
S140	0	7,68E-05	253,12	76,8	156,6	5,8
S140	100	7,48E-05	267,08	74,01	153,3	2,7
S140	150	6,19E-05	315,87	64,2	150,9	-16,3
S140	200	4,20E-05	320,43	47,86	139,9	-29,4
S140	225	3,73E-05	330,52	48,34	146,2	-33,5
S140	250	3,37E-05	329,01	46,7	144	-34,2
S140	275	3,11E-05	336,93	18,65	122,6	-59,3
S140	300	3,35E-05	349,13	13,56	134,9	-71,1

Table S2. Thermal demagnetization data.

(1) Sample = sample name
(2) Temperature (°C)
(3) NRM intensity (A/m)
(4) Core Declination (°E)
(5) Core Inclination (°)
(6) Geographic Declination (°E)
(7) Geographic Inclination (°)

S140	325	3,56E-05	356,22	12,7	155,1	-74,8
S140	350	3,16E-05	0,39	2,65	174,1	-85,3
S140	375	3,35E-05	9,53	-16,34	316,7	-72,9
S140	400	2,81E-05	339,28	-17,78	40,2	-64,3
S140	425	4,71E-05	354,62	-34,65	357,4	-57
S140	450	4,45E-05	17,36	-35,2	325,3	-53,1
S140	475	5,18E-05	24,85	-41,66	323,2	-44,5
S140	500	5,09E-05	1,6	-40,67	347,3	-51,3
S140	525	5,24E-05	29,23	-43,5	321,3	-41
S140	550	5,04E-05	358,04	-40,14	351,7	-51,8
S140	575	5,59E-05	4,67	-37,79	343	-54
S140	600	3,78E-05	5,15	-49,95	344,8	-41,9
S140	625	5,31E-05	33,16	-25,08	298	-50,6
S140	650	5,70E-05	28,76	-76,56	342,7	-13,7
S140	675	4,32E-05	21,31	-34,05	319,8	-52,3
S146	0	1,20E-04	237,54	75,41	156,8	9,7
S146	100	1,09E-04	262,54	75,26	154,6	3,8
S146	150	9,96E-05	296,85	75,24	156,1	-4,7
S146	200	9,00E-05	299,7	71,72	153,4	-7
S146	225	8,31E-05	317,27	67,95	154,1	-14,1
S146	250	7,98E-05	314,49	65,42	151,4	-15
S146	275	6,54E-05	321,35	64,06	152,6	-18,1
S146	300	6,45E-05	329,77	63,55	155,4	-20,7
S146	325	5,76E-05	325,38	56,82	149,2	-24,9
S146	350	4,95E-05	321,1	52,12	143,7	-26,7
S146	375	4,59E-05	338,88	47,8	151,7	-36,9
S146	400	4,14E-05	332,43	41,51	142,5	-39,8
S146	425	4,16E-05	333,86	46,98	147,5	-35,9
S146	450	4,78E-05	353,32	45,21	162,9	-42,4
S146	475	3,95E-05	337,17	40,33	145,5	-42,8
S146	500	4,54E-05	322,56	15,18	105,5	-49,2
S146	525	3,36E-05	352,72	16,93	148,8	-69,8
S146	550	4,53E-05	329,66	32,9	132,5	-44,8
S146	575	3,55E-05	350,54	13,25	137,9	-72,1
S146	600	2,34E-05	14,03	12,02	213,8	-70,2
S146	625	2,67E-05	343,82	-9,28	54,3	-72,3
S146	650	1,54E-05	305,23	-39,63	34,6	-27,8
S146	675	3,96E-05	354,81	15,39	153	-71,9
S152	0	1,97E-04	135,72	74,39	176,7	18
S152	100	1,73E-04	129,36	76,94	175,7	15,1
S152	150	1,44E-04	98,05	74,61	180,7	8,9
S152	200	1,19E-04	85,95	70,66	184,7	5,3
S152	225	1,08E-04	87,99	65,12	190,3	5,5
S152	250	1,03E-04	84,62	65,74	189,5	4,2
S152	275	9,39E-05	88,73	66,81	188,6	5,9
S152	300	9,08E-05	92,59	64,83	190,7	7,4
S152	325	8,94E-05	73,69	65,84	188,4	-0,2
S152	350	7,88E-05	78,66	64,48	190,3	1,5
S152	375	7,11E-05	80,8	60,5	194,4	1,6
S152	400	6,37E-05	136,38	60,29	188	27,5
S152	425	6,02E-05	101,39	59,49	195,8	11,8
S152	450	4,96E-05	94,54	35,02	220,8	7,7
S152	475	5,72E-05	126,97	29	224,5	35,5
S152	500	4,30E-05	94,5	1,47	254,4	4,6
S152	525	5,61E-05	101,57	-0,19	256,9	11,5
S152	550	4,16E-05	124,92	-12,11	274,3	32
S152	575	5,55E-05	140,14	-14,98	284,5	44,8
S152	600	5,85E-05	142,96	-0,28	264,9	52,3
S152	625	6,02E-05	116,35	-12,4	272,2	23,8
S152	650	4,08E-05	63,03	-3,66	255,8	-27,2
S152	675	7,16E-05	102,92	5,46	251,3	13,4
S160	0	1,83E-04	173,72	60,87	172,2	37,9
S160	100	1,56E-04	180,7	59,06	167,8	39,9
S160	150	1,12E-04	177,77	57,05	169,9	41,9
S160	200	9,19E-05	192,75	52,55	157,3	45,2
S160	225	8,35E-05	194,22	48	153,7	49,2
S160	250	7,62E-05	185,07	38,6	160,3	60,1
S160	275	7,24E-05	178,49	34,11	171,2	64,9
S160	300	6,87E-05	168,07	23,25	205,6	71,7
S160	325	6,52E-05	159,01	2,84	273,4	68,2
S160	350	6,52E-05	161,38	6,82	263,7	71,4
S160	375	6,96E-05	157,85	3,77	270,2	67,4
S160	400	6,42E-05	169,01	0,11	296,8	75,9
S160	425	6,62E-05	170,13	-11,14	322,1	67,6
S160	450	8,17E-05	165,88	-22,99	325,1	55,2
S160	475	8,53E-05	165,65	-24,32	325,8	53,9
S160	500	8,41E-05	167,96	-10,89	317	66,8
S160	525	8,02E-05	161,8	-24,75	320,9	51,9
S160	550	8,43E-05	166,95	-38,78	334,9	40,7
S160	575	9,37E-05	183,08	-31,11	352,4	49,8
S160	600	1,02E-04	168,81	-31,24	333,8	48,4
S160	625	1,12E-04	144,54	-45,95	321,5	26,5
S160	650	1,04E-04	159,31	-47,07	332	31
S160	675	8,35E-05	168,6	-72,32	344,8	8,3
S164	0	1,17E-04	220,03	75,45	157,3	20,9
S164	100	9,68E-05	247,71	80,89	158,6	13,3
S164	150	7,19E-05	289,54	72,98	151,2	4
S164	200	5,45E-05	293,09	65,99	145,3	0,1
S164	225	4,74E-05	277,77	66,38	143,8	6,1
S164	250	4,01E-05	282,92	60,33	138,4	2,4
S164	275	3,45E-05	291,63	54,77	134,8	-3,9
S164	300	2,58E-05	330,18	60,71	152,7	-15,5
S164	325	3,11E-05	292,25	51,94	132,3	-5,3
S164	350	2,54E-05	258,76	58,3	135,1	14,4
S164	375	1,25E-05	320,93	48,62	140,6	-22
S164	400	1,45E-05	306,96	51,47	136,5	-13,5
S164	425	2,72E-05	343,1	48,7	154,6	-29,4
S164	450	1,79E-05	324,58	46,27	141	-25,4
S164	475	2,15E-05	335,24	37,07	142,4	-37,5
S164	500	4,05E-05	354,32	28,39	159,3	-51,2
S164	525	1,80E-05	336,73	5,05	109,3	-62,4
S164	550	2,55E-05	284,21	12,53	92,4	-11,4
S164	575	1,73E-05	241,61	28,32	104,3	29,6
S164	600	3,93E-05	303,88	14,17	99,9	-29,3
S164	625	2,49E-05	256,2	-10,03	64,8	11,6
S164	650	1,66E-05	235,3	12,2	85,2	35,8
S164	675	3,94E-05	218,19	4,57	71,9	51,8
S171	0	1,46E-04	226,45	78,03	163,1	19,1
S171	100	1,18E-04	267,35	79,93	162	11,3
S171	150	8,95E-05	315,35	72,81	160,3	-1,4
S171	200	7,00E-05	318,32	65,58	156,2	-7,4
S171	225	6,41E-05	326,83	65,4	158,9	-9,7
S171	250	4,86E-05	344,41	55,94	163	-21,8
S171	275	4,17E-05	18,96	57,02	183,1	-20,2
S171	300	2,96E-05	14,32	32,8	189,1	-44,1
S171	325	2,16E-05	19,15	50,4	185,8	-26,4
S171	350	2,68E-05	359,2	41,49	171,5	-37,5
S171	375	2,61E-05	359,26	39,95	171,6	-39
S171	400	2,29E-05	18,02	37,76	190,5	-38,4
S171	425	2,98E-05	13,55	20,62	195,2	-55,7
S171	450	2,35E-05	30,49	11,36	226,8	-52,3
S171	475	3,12E-05	18,43	4,15	223,7	-66,2
S171	500	4,23E-05	21,22	4,22	227,6	-64
S171	525	2,66E-05	37,91	-18,81	278,9	-52,6
S171	550	4,95E-05	42,62	-25,78	288,5	-47,2
S171	575	4,89E-05	31,84	-23,95	289,7	-57,1
S171	600	5,42E-05	64,25	-29,35	289,8	-27,7
S171	625	5,29E-05	49,15	-18,56	277,4	-42
S171	650	3,94E-05	10,62	-45,93	339,7	-53,9
S171	675	3,72E-05	311,45	-43,17	35,6	-37,2
S176	0	1,76E-04	190,01	77,84	170	25
S176	100	1,38E-04	229,78	77,17	161,8	21
S176	150	9,64E-05	270,41	71,27	153,1	12,2
S176	200	6,78E-05	267,6	59,17	140,7	12,4
S176	225	6,35E-05	276,27	51,96	134,2	6,4
S176	250	5,54E-05	287,46	53,87	138,1	0,5
S176	275	4,78E-05	267,18	49,3	130,6	11,6
S176	300	3,20E-05	251,28	51,16	132,5	21,8
S176	325	3,91E-05	248,98	40,22	120,8	24,3
S176	350	4,23E-05	253,54	27,9	107,5	20,4
S176	375	4,45E-05	247,81	25,24	104,3	25,4
S176	400	3,41E-05	243,14	22,59	101,1	29,5
S176	425	3,54E-05	259,22	18,72	98,6	14,2
S176	450	3,54E-05	233,56	-10,33	61,1	31,9
S176	475	3,80E-05	233,4	-7,24	64,5	33,2
S176	500	6,43E-05	242,44	-15,06	59,8	22,1
S176	525	5,04E-05	237,91	-33,77	40	17,8
S176	550	7,42E-05	232,8	-27,1	43,7	25
S176	575	6,69E-05	215,02	-50,95	14,8	19,1
S176	600	6,88E-05	259,79	-58,5	23,4	-5,8
S176	625	8,67E-05	229,35	-55,95	17,8	9,7
S176	650	7,95E-05	244,39	-54,31	24,1	3,6
S176	675	7,68E-05	244,59	-63,05	16,5	-0,6
S181	0	3,96E-04	159,14	68,18	175,6	35,2
S181	100	3,55E-04	139,6	66,51	184	32
S181	150	3,13E-04	136,11	65,4	186,1	31,7
S181	200	2,87E-04	132,35	62,34	190,2	32,1
S181	225	2,70E-04	138,81	62,07	188,3	34,7
S181	250	2,60E-04	127,96	62,58	191,1	30,2
S181	275	2,41E-04	137,59	60,68	190,1	35,1
S181	300	2,34E-04	131,96	59,98	192,7	33,2
S181	325	2,18E-04	134,33	58,25	193,7	35,1
S181	350	2,10E-04	138,48	56,42	194	38
S181	375	2,03E-04	140,67	53,4	196,2	40,8
S181	400	2,04E-04	141,6	55,18	193,9	40,1
S181	425	1,89E-04	137,34	52,07	199,1	39,9
S181	450	1,91E-04	142,32	53,17	195,7	41,7
S181	475	1,83E-04	144,49	51,63	196,2	43,7
S181	500	1,72E-04	144,18	50,39	197,7	44,3
S181	525	1,72E-04	134,27	51,71	200,8	38,4
S181	550	1,74E-04	156,77	47,52	192,1	52,2
S181	575	1,51E-04	158,4	48,59	189,6	52
S181	600	5,96E-05	13,17	74,34	169,8	-0,3
S181	625	1,12E-04	149,35	44,19	201,7	50,9
S181	650	1,10E-04	157,86	40,21	199,4	58,3
S181	675	9,75E-05	185,09	54,38	161,6	50,4
S187	0	3,70E-04	264,62	74,95	161,5	17,8
S187	100	3,43E-04	276,53	75,2	162,1	14,8
S187	150	2,85E-04	303,66	72,38	162,6	6,8
S187	200	2,36E-04	297,36	68,8	158,4	6,5
S187	225	2,19E-04	305,44	66,7	158,5	2,8
S187	250	2,04E-04	295,76	66,34	156	5,8
S187	275	1,78E-04	309,54	63,98	157,5	-0,2
S187	300	1,66E-04	307,84	62,32	155,8	-0,8
S187	325	1,57E-04	308,53	60,84	154,9	-2
S187	350	1,48E-04	301,56	61,74	153,5	1,2
S187	375	1,41E-04	300,42	59,91	151,7	0,6
S187	400	1,34E-04	301,24	60,68	152,5	0,7
S187	425	1,28E-04	312,3	58,23	154,3	-5,2
S187	450	1,19E-04	324,85	60,93	160,9	-7,1
S187	475	1,17E-04	320,23	66,79	162,7	-1,2
S187	500	9,65E-05	315,32	71,78	164,6	3,7
S187	525	1,00E-04	277,82	64,43	151,4	12
S187	550	8,26E-05	274,34	65,98	152,6	13,7
S187	575	6,75E-05	263,83	56,29	141,9	17,5
S187	600	1,29E-04	160,63	58,69	191,6	45,9
S187	625	8,41E-05	282,05	56,7	144,5	7,7
S187	650	8,00E-05	183,97	65,26	175,1	41,7
S187	675	7,10E-05	315,21	50,47	150	-11,9
S204	0	5,88E-04	213,9	74,16	160,7	24
S204	100	5,10E-04	226,35	75,44	159,1	20,8
S204	150	4,13E-04	242,86	74,9	156,2	17,5
S204	200	3,29E-04	250,32	72,69	153,3	16,3
S204	225	3,00E-04	255,19	71,54	151,8	15,1
S204	250	2,83E-04	256,23	68,47	148,6	15,3
S204	275	2,36E-04	249,51	66,51	147,1	18,2
S204	300	2,07E-04	255,47	62,82	142,8	16,4
S204	325	1,86E-04	252	63,44	143,8	17,8
S204	350	1,58E-04	250,97	64,11	144,5	18,1
S204	375	1,42E-04	258,36	63,13	143	15,1
S204	400	1,18E-04	250,51	52,43	132,4	20,5
S204	425	1,11E-04	253,83	50,81	130,4	18,7

Table S2. Thermal demagnetization data.

(1) Sample = sample name
(2) Temperature (°C)
(3) NRM intensity (A/m)
(4) Core Declination (°E)
(5) Core Inclination (°)
(6) Geographic Declination (°E)
(7) Geographic Inclination (°)

S204	450	1,05E-04	266,74	33,72	113,1	8,8
S204	475	9,93E-05	261,75	36,27	115,3	13,1
S204	500	7,76E-05	279,99	30,76	112,4	-2,8
S204	525	7,60E-05	266,07	9,96	89,4	5,7
S204	550	7,53E-05	251,43	17,21	94,7	20,8
S204	575	8,71E-05	252,4	-3,43	73,3	16,6
S204	600	8,89E-05	266,88	-0,11	79,6	3
S204	625	8,61E-05	250,81	-1,11	75,3	18,6
S204	650	6,63E-05	263,55	-28,47	51,2	0,3
S204	675	4,50E-05	300,38	-49,83	29,3	-27,8
S215	0	5,35E-04	217,96	74,52	166,4	17,1
S215	100	5,04E-04	229,75	77,4	166,4	13
S215	150	4,21E-04	245,33	79,08	166,2	9,5
S215	200	3,25E-04	235,39	81,76	169,4	9,6
S215	225	2,81E-04	255,41	78,51	165,1	7,8
S215	250	2,43E-04	213,01	87,36	174,8	7,2
S215	275	2,02E-04	253,29	81,76	168,3	7,3
S215	300	1,68E-04	251,23	81,33	168	7,7
S215	325	1,22E-04	186,03	81,59	175,4	13,4
S215	350	1,21E-04	128,5	80,81	183,6	10,7
S215	375	1,08E-04	147,61	77,54	183,2	15,5
S215	400	9,89E-05	210,51	78,42	170,2	14,9
S215	425	1,07E-04	197,73	80,92	173,4	13,6
S215	450	7,61E-05	164,24	65,8	183,5	28,2
S215	475	8,58E-05	98,73	62,15	204,1	8,5
S215	500	6,37E-05	99,02	59,24	207	8,9
S215	525	6,08E-05	66,54	62,51	201,5	-6,1
S215	550	3,48E-05	52,86	37,64	220,4	-25
S215	575	4,22E-05	79,39	16,57	248,6	-8,7
S215	600	4,21E-05	73,54	23,89	240,4	-12,9
S215	625	8,33E-05	61,77	-10,44	275,5	-28,6
S215	650	2,93E-05	50,01	-40,23	312	-33
S215	675	1,99E-05	352,33	-56,41	1,7	-38,2
S221	0	7,65E-04	208,86	79,74	165,2	13
S221	100	6,49E-04	211,06	83,52	166,9	9,5
S221	150	5,06E-04	257,45	87,62	168	4,5
S221	200	4,02E-04	311,47	87,09	168,1	2,1
S221	225	3,36E-04	343,99	85,29	169	-0,5
S221	250	2,84E-04	357,18	87,42	170,2	1,4
S221	275	2,37E-04	29,72	81,78	174,4	-3,1
S221	300	2,17E-04	25,79	80,46	174,4	-4,6
S221	325	1,83E-04	10,76	83,67	171,5	-2,2
S221	350	1,49E-04	0,34	79,6	170,3	-6,4
S221	375	1,26E-04	32,42	70,96	180,6	-12,1
S221	400	1,10E-04	33,51	71	180,9	-11,8
S221	425	9,89E-05	38,62	56,42	192,1	-21,9
S221	450	1,14E-04	57,61	54,6	200,7	-14,6
S221	475	9,32E-05	68,78	42,91	214,7	-12,5
S221	500	6,88E-05	54,19	30,77	222,2	-27,8
S221	525	5,03E-05	32,75	24,52	216,8	-47,3
S221	550	8,66E-05	55,16	10,05	245,5	-33,3
S221	575	7,72E-05	51,27	11,79	242,4	-36,6
S221	600	7,03E-05	43,45	-13,45	275,6	-46,1
S221	625	8,57E-05	73,36	-18,88	278,8	-17
S221	650	8,72E-05	40,54	-26,78	295	-45,1
S221	675	1,11E-04	41,42	-16,98	281,2	-47,4
S230	0	7,58E-04	243,7	74,17	162,4	17,6
S230	100	7,81E-04	255,42	78,41	165,7	13,7
S230	150	5,94E-04	272,58	76,79	163,9	10,1
S230	200	4,41E-04	289,83	75,27	163,4	5,7
S230	225	3,70E-04	295,8	72,86	161,9	3,2
S230	250	3,10E-04	286,56	71,1	159,1	5,2
S230	275	2,16E-04	301,52	66,03	157	-2
S230	300	2,07E-04	307,02	63,66	156,4	-5,2
S230	325	1,51E-04	306,66	55,69	150	-9,9
S230	350	1,24E-04	300,65	46,4	140	-11,9
S230	375	1,02E-04	294,3	35,76	128	-12,5
S230	400	7,33E-05	295,75	31,02	124,1	-15,5
S230	425	6,76E-05	287,24	30,45	120,8	-8,9
S230	450	5,85E-05	314,66	30,58	132,4	-29,8
S230	475	4,21E-05	319	15,45	119,6	-41,5
S230	500	6,43E-05	318,61	-4,76	92,6	-48,6
S230	525	5,44E-05	330,4	-23,84	58,8	-59,1
S230	550	8,55E-05	333,64	-61,03	12,8	-36,4
S230	575	8,01E-05	310,27	-53,76	29,4	-31,9
S230	600	9,10E-05	290,31	-64,59	22,4	-18,6
S230	625	8,54E-05	359,66	-60,56	357,5	-40,4
S230	650	1,15E-04	256,56	-75,87	11,1	-7,4
S230	675	9,23E-05	342,95	-51,01	13,2	-47,6
S234	0	8,40E-04	241,06	80,5	168,6	16,5
S234	100	7,78E-04	245,93	81,6	169,3	15,3
S234	150	6,23E-04	267,84	82,25	169,4	12,2
S234	200	4,94E-04	273,27	82,75	169,9	11,5
S234	225	4,40E-04	274,14	83,17	170,3	11,4
S234	250	3,99E-04	266,28	85,93	173,1	12,2
S234	275	3,22E-04	263,51	83,09	170,2	12,7
S234	300	2,82E-04	216,91	86,02	174,8	15,2
S234	325	2,54E-04	237,82	83,28	171,4	15,5
S234	350	2,13E-04	240,43	82,34	170,4	15,7
S234	375	1,92E-04	253,6	80,87	168,2	14,4
S234	400	1,77E-04	264,03	82,46	169,6	12,7
S234	425	2,11E-04	214,79	85,29	174,5	15,9
S234	450	1,46E-04	3,78	88,5	177,4	10,5
S234	475	1,53E-04	51,15	87,16	179,5	10,2
S234	500	1,26E-04	206,74	85,07	175	16,4
S234	525	1,32E-04	68,82	81,5	185,3	8,8
S234	550	1,40E-04	89,29	81,34	186,1	11,8
S234	575	1,06E-04	34,9	67,42	190,1	-6,7
S234	600	1,00E-04	64,77	61,45	202,9	-1
S234	625	1,18E-04	62,37	41,87	219,6	-11,5
S234	650	9,67E-05	63,11	52,19	210,6	-6,1
S234	675	8,63E-05	38,2	68,39	190,5	-5,2
S240	0	1,10E-03	240,82	74,91	159,8	13,1
S240	100	1,09E-03	227,45	79,74	165,5	12,9
S240	150	8,99E-04	251,59	79,38	163,1	9,2
S240	200	7,14E-04	261,54	79,66	163	7,4
S240	225	6,49E-04	269,37	78,42	161,6	6
S240	250	5,85E-04	259,07	78,29	161,7	8,1
S240	275	4,96E-04	277,12	77,57	160,9	4,3
S240	300	4,55E-04	298,26	78,87	163,5	0,7
S240	325	4,19E-04	275,7	78,54	161,8	4,8
S240	350	3,47E-04	274,61	77,36	160,6	4,8
S240	375	3,25E-04	276,48	78,26	161,6	4,6
S240	400	2,77E-04	259,3	76,82	160,2	8,3
S240	425	2,62E-04	271,13	79,45	162,7	5,7
S240	450	2,41E-04	254,29	81,31	164,8	8,3
S240	475	2,34E-04	241,19	80,92	165,2	10,3
S240	500	1,71E-04	246,45	72,14	156,5	12,8
S240	525	1,83E-04	251,28	80,26	163,9	9
S240	550	1,46E-04	241,15	74,41	159,3	13,3
S240	575	1,17E-04	248,9	64,89	149,2	14,3
S240	600	9,32E-05	262,19	65,52	148,7	8,7
S240	625	4,75E-05	184,14	64,13	171,2	31,8
S240	650	8,98E-05	252,38	85,38	168,8	7,4
S240	675	5,23E-05	299,94	75,41	160,7	-1,4
S253	0	1,43E-03	312,96	83,78	163,6	12,7
S253	100	1,52E-03	317,5	82,57	163,2	11,5
S253	150	1,25E-03	338,49	79,14	164,3	6,9
S253	200	1,02E-03	343,78	78,51	165,1	5,9
S253	225	9,16E-04	347,97	77,97	165,8	5,2
S253	250	8,16E-04	346,41	78,02	165,5	5,3
S253	275	6,95E-04	339,43	77,6	163,9	5,4
S253	300	6,19E-04	346,74	78,42	165,6	5,7
S253	325	5,13E-04	7,37	76,78	170	3,9
S253	350	4,29E-04	2,41	77,42	168,8	4,4
S253	375	3,79E-04	357,85	74,2	167,7	1,2
S253	400	3,32E-04	10,07	77,05	170,5	4,2
S253	425	2,98E-04	1,84	80,71	168,6	7,7
S253	450	2,43E-04	32,95	78	174,8	6,8
S253	475	2,41E-04	32,24	79,17	174,1	7,8
S253	500	1,88E-04	69,09	82,48	175,5	14,2
S253	525	1,98E-04	72,69	81,14	177	14,2
S253	550	1,54E-04	89,07	79,48	179,3	16,5
S253	575	1,14E-04	109,36	47,88	212,8	25,4
S253	600	7,78E-05	127,95	44,01	214,9	38,8
S253	625	8,85E-05	110,31	9,44	254,8	22
S253	650	5,72E-05	101,85	17,77	244,2	16
S253	675	4,63E-05	159,31	24,49	233,6	69,3
S268	0	2,74E-03	174,33	88,2	160,5	13,8
S268	100	2,51E-03	81,44	88,77	161,5	11,8
S268	150	2,09E-03	4,53	85,93	160,6	7,9
S268	200	1,67E-03	12,14	84,68	161,4	6,8
S268	225	1,48E-03	9,44	84,67	161,2	6,7
S268	250	1,33E-03	8,86	83,47	161,3	5,5
S268	275	1,09E-03	11,33	81,55	161,9	3,7
S268	300	9,97E-04	8,06	82,69	161,3	4,8
S268	325	8,50E-04	7,39	82,29	161,3	4,4
S268	350	7,21E-04	5,64	81,62	161,1	3,7
S268	375	6,19E-04	11,65	82,17	161,9	4,3
S268	400	5,66E-04	13,15	80,08	162,5	2,3
S268	425	5,04E-04	17,98	80,32	163,3	2,8
S268	450	4,40E-04	4,59	78,83	161,2	0,9
S268	475	4,22E-04	11,98	82,35	161,9	4,5
S268	500	3,12E-04	15,31	80,06	162,9	2,4
S268	525	3,22E-04	32,25	83,26	163,9	6,3
S268	550	2,41E-04	44,35	79,62	167,5	4,5
S268	575	1,99E-04	53,46	68,82	177,2	-0,9
S268	600	1,11E-04	26,94	64,23	171,9	-11,1
S268	625	1,43E-04	49,44	44,57	194,9	-17,9
S268	650	8,84E-05	67,63	54,7	192,6	-2,6
S268	675	1,09E-04	27,98	48,45	180,3	-24,7
S273	25	2,26E-03	304,13	84,34	161,5	9,8
S273	100	2,18E-03	310,82	83,56	161,4	8,7
S273	150	1,77E-03	331,15	80,21	161,6	4,4
S273	200	1,47E-03	337,1	78,33	161,8	2,2
S273	225	1,33E-03	342,19	77,8	162,6	1,4
S273	250	1,20E-03	350,19	77,16	164,1	0,3
S273	275	9,68E-04	356,45	75,26	165,4	-1,7
S273	300	8,48E-04	356,41	74,32	165,3	-2,7
S273	325	7,41E-04	354,45	72,79	164,6	-4,1
S273	350	6,59E-04	3,24	72,16	167,3	-4,8
S273	375	5,82E-04	3,98	70,59	167,6	-6,4
S273	400	5,09E-04	15,15	67,96	172	-8,3
S273	425	4,66E-04	12,53	67,51	171,1	-9
S273	450	3,97E-04	14,85	70,36	171,3	-6
S273	475	3,88E-04	13,34	69,91	170,9	-6,6
S273	500	3,30E-04	23,45	67,59	175,1	-7,6
S273	525	2,67E-04	32,08	62,09	180,9	-10,8
S273	550	2,53E-04	17,77	58,1	176	-17,4
S273	575	1,41E-04	63,06	58,58	194	-2,2
S273	600	9,80E-05	65,65	41,07	210,3	-8,9
S273	625	1,33E-04	79,81	7,89	246,2	-8
S273	650	1,18E-04	44,16	22,53	217,2	-34
S273	675	1,16E-04	54,74	13,74	232	-29,5
S281	0	2,68E-03	174,05	88,75	162,4	14,2
S281	100	2,74E-03	336,61	88,14	161,5	11,3
S281	150	2,25E-03	342,64	84,65	160,7	7,9
S281	200	1,81E-03	343,79	83,08	160,3	6,3
S281	225	1,60E-03	344,85	82,65	160,4	5,9
S281	250	1,41E-03	339,06	82,08	159,4	5,6
S281	275	1,20E-03	346,96	81,97	160,5	5,2
S281	300	1,09E-03	344,19	81,39	159,9	4,7
S281	325	9,44E-04	348,74	81,45	160,6	4,6
S281	350	7,61E-04	359,05	82,01	162,2	5
S281	375	6,78E-04	345,4	80,23	159,8	3,5
S281	400	6,26E-04	351,82	80,47	160,9	3,6
S281	425	5,69E-04	346	80,24	159,9	3,5
S281	450	5,37E-04	344,94	78,24	159,2	1,6
S281	475	4,99E-04	343,77	79,63	159,4	3
S281	500	3,99E-04	343,09	78,26	158,9	1,8
S281	525	3,52E-04	3,89	78,22	163,1	1,2
S281	550	2,80E-04	7,63	75,67	164,2	-1,2

Table S2. Thermal demagnetization data.

(1) Sample = sample name
(2) Temperature (°C)
(3) NRM intensity (A/m)
(4) Core Declination (°E)
(5) Core Inclination (°)
(6) Geographic Declination (°E)
(7) Geographic Inclination (°)

S281	575	1,74E-04	346,35	77,88	159,4	1,2
S281	600	1,04E-04	314,17	70,38	148,3	-0,9
S281	625	8,19E-05	21,75	47,4	178,6	-26,6
S281	650	8,46E-05	11,17	59,31	168,2	-17,1
S281	675	1,06E-04	20,31	73,59	167,9	-2,4
S287	25	3,63E-03	296,15	84,49	158,2	11,5
S287	100	3,20E-03	305,93	81,76	156,5	9,1
S287	150	2,56E-03	321,84	78,72	156,3	5
S287	200	2,11E-03	326,66	77,48	156,4	3,5
S287	225	1,92E-03	326,03	76,21	155,6	2,5
S287	250	1,71E-03	323,47	75,88	154,9	2,5
S287	275	1,38E-03	328,01	73,52	154,6	-0,1
S287	300	1,21E-03	331,04	67,87	152,7	-5,5
S287	325	1,10E-03	322,42	71,6	152,2	-0,8
S287	350	9,24E-04	316,77	70,12	149,8	-0,7
S287	375	8,25E-04	319,7	69,19	150	-2,1
S287	400	7,30E-04	314,67	69,1	148,6	-1
S287	425	6,72E-04	312,4	67,82	147,1	-1,3
S287	450	6,21E-04	316,49	67,76	148,2	-2,4
S287	475	5,99E-04	319,89	66,48	148,3	-4,3
S287	500	5,44E-04	318,44	63,92	146,2	-5,8
S287	525	4,53E-04	306,18	62,31	141,2	-3
S287	550	3,42E-04	318,38	63,48	145,9	-6,2
S287	575	2,47E-04	305,87	56,01	136,1	-6,7
S287	600	1,88E-04	294,56	47,51	125,2	-5,4
S287	625	1,10E-04	298,61	56,06	133,9	-3,4
S287	650	1,57E-04	309,01	36,49	121,5	-20,3
S287	675	1,12E-04	290,21	40,25	117,2	-5,7
S299	25	3,94E-03	223,48	85,18	159,9	13,5
S299	100	3,74E-03	253,91	85,7	159,1	11,2
S299	150	3,02E-03	295,17	84,12	157,9	7,5
S299	200	2,56E-03	305,17	83,8	158,2	6,4
S299	225	2,28E-03	309,36	82,54	157,5	5,2
S299	250	2,02E-03	310,46	82,67	157,7	5,2
S299	275	1,56E-03	312,13	82,76	157,9	5,1
S299	300	1,39E-03	309,23	82,05	157,1	4,9
S299	325	1,27E-03	310,31	82,25	157,4	4,9
S299	350	1,04E-03	304,53	79,96	155	4,2
S299	375	9,33E-04	302,61	79,76	154,6	4,4
S299	400	7,78E-04	287,61	77,89	151,7	6,2
S299	425	1,00E-03	330,22	72,34	154,6	-5,4
S299	450	6,18E-04	285,38	79,5	153,1	7,1
S299	475	6,22E-04	294,14	76,34	150,8	4,2
S299	500	4,68E-04	275,36	76,65	149,8	8,5
S299	525	4,18E-04	280,61	76,17	149,6	7,2
S299	550	3,74E-04	281,5	73,61	147,1	6,4
S299	575	2,40E-04	271,06	71,68	144,7	9,2
S299	600	1,30E-04	279,82	61,46	135,1	4,1
S299	625	8,44E-05	290,31	64,78	139,7	0,7
S299	650	1,16E-04	255,72	45,44	117,9	17,1
S299	675	1,45E-04	280,28	29,25	103,9	-3,9
S314	25	6,04E-03	31,25	82,48	162,2	8,5
S314	100	5,43E-03	5,19	80,61	159,1	5,6
S314	150	4,41E-03	349,63	79,38	156,4	4,5
S314	200	3,72E-03	3,28	77,59	159	2,6
S314	225	3,27E-03	0,12	77,7	158,3	2,7
S314	250	3,01E-03	352,15	77,14	156,5	2,3
S314	275	2,40E-03	342,97	77,53	154,7	3,1
S314	300	2,02E-03	345,6	75,71	154,8	1,1
S314	325	1,74E-03	342,76	75,54	154	1,2
S314	350	1,48E-03	344,94	73,99	154,2	-0,5
S314	375	1,30E-03	332,58	74,66	151,3	1,3
S314	400	1,11E-03	334,94	73,8	151,5	0,3
S314	425	6,81E-04	288,31	78,92	147,6	11,3
S314	450	8,82E-04	325,14	74,85	149,7	2,4
S314	475	8,68E-04	323,35	73,85	148,7	1,9
S314	500	6,57E-04	322,02	69,97	146,1	-1
S314	525	5,44E-04	301,07	68,33	139,8	3,2
S314	550	4,59E-04	305,36	69,22	141,4	2,5
S314	575	2,80E-04	290,39	50,26	121,5	-0,9
S314	600	1,63E-04	272,16	13,49	81,9	1,4
S314	625	1,45E-04	274,63	-2,94	66,6	-5,2
S314	650	1,85E-04	268,92	2,71	70,6	1,7
S314	675	1,98E-04	268,58	-7,66	60,5	-0,6

OH2 (samples S333– S1701; hand samples oh2_)

S333	25	7,98E-04	64	47,17	177	-2,5
S333	100	6,84E-04	60,12	42,12	179,7	-7,5
S333	150	5,86E-04	59,01	37,17	183,4	-11
S333	200	4,89E-04	51,84	36,16	180,7	-16,2
S333	225	4,43E-04	55,01	34,67	183,5	-15,1
S333	250	3,73E-04	53,01	34,6	182,6	-16,5
S333	275	3,05E-04	38,89	28,09	178,9	-29,7
S333	300	2,54E-04	44,98	24,08	186,6	-28,5
S333	325	1,98E-04	44,62	24,81	185,7	-28,3
S333	350	1,99E-04	35,93	23,27	180,5	-35,1
S333	375	1,78E-04	24,17	15,7	175,3	-47,9
S333	400	1,67E-04	29,68	8,17	189,1	-50,1
S333	425	1,58E-04	33,42	13,42	187,2	-43,8
S333	450	1,36E-04	30,93	4,93	194,2	-51,3
S333	475	1,52E-04	27,67	5,56	190	-53,3
S333	500	1,44E-04	26,49	-1,38	198,2	-58,6
S333	525	1,17E-04	10,54	-7,87	183,3	-74,9
S333	550	1,09E-04	3,61	2,15	149,2	-68,6
S333	575	1,09E-04	351,2	-9,95	94,8	-77,6
S333	600	9,71E-05	322,06	-24,67	33,2	-54,4
S333	625	1,43E-04	352,6	-25,15	6,1	-80,8
S333	650	1,18E-04	326,42	-32,78	17,9	-57
S333	675	1,37E-04	5,26	-24,72	279,8	-82,5
oh2_3	0	1,10E-02	174,5	2,4	174,5	2,4
oh2_3	100	1,04E-02	173,6	-3,2	173,6	-3,2
oh2_3	150	8,83E-03	172,3	-5,2	172,3	-5,2
oh2_3	200	7,71E-03	173	-6,1	173	-6,1
oh2_3	225	7,29E-03	173,6	-5,8	173,6	-5,8
oh2_3	250	6,66E-03	172,6	-9,4	172,6	-9,4
oh2_3	275	6,03E-03	170,1	-10,3	170,1	-10,3
oh2_3	300	5,62E-03	167,4	-10,8	167,4	-10,8
oh2_3	325	5,00E-03	170	-11,4	170	-11,4
oh2_3	350	4,76E-03	170	-7,9	170	-7,9
oh2_3	375	4,61E-03	170,4	-12,6	170,4	-12,6
oh2_3	400	4,57E-03	172,3	-8,8	172,3	-8,8
oh2_3	425	4,33E-03	172,9	-4,3	172,9	-4,3
oh2_3	450	4,10E-03	175,5	6,5	175,5	6,5
oh2_3	475	4,46E-03	168,2	-8,7	168,2	-8,7
oh2_3	500	4,40E-03	161	-14,7	161	-14,7
oh2_3	525	4,03E-03	164,1	-6,9	164,1	-6,9
oh2_3	550	3,26E-03	148,6	-2,9	148,6	-2,9
oh2_3	575	3,39E-03	163,4	-13,6	163,4	-13,6
oh2_3	600	3,05E-03	156,7	-3,4	156,7	-3,4
oh2_3	625	3,21E-03	162,1	-18,5	162,1	-18,5
oh2_3	650	3,34E-03	154,2	11	154,2	11
oh2_3	675	2,81E-03	146,5	-7,5	146,5	-7,5
oh2_4	0	5,89E-03	341,1	10,7	341,1	10,7
oh2_4	100	5,54E-03	341,2	7,5	341,2	7,5
oh2_4	150	4,66E-03	343	2,6	343	2,6
oh2_4	200	4,34E-03	345,2	1,6	345,2	1,6
oh2_4	225	4,00E-03	344,3	1,9	344,3	1,9
oh2_4	250	3,61E-03	340,6	0,3	340,6	0,3
oh2_4	275	3,49E-03	344,6	-3,1	344,6	-3,1
oh2_4	300	3,00E-03	344,7	-8	344,7	-8
oh2_4	325	2,61E-03	346,3	-6,2	346,3	-6,2
oh2_4	350	2,38E-03	345,4	-9,3	345,4	-9,3
oh2_4	375	2,24E-03	338,4	-11,5	338,4	-11,5
oh2_4	400	2,44E-03	342	-8,4	342	-8,4
oh2_4	425	2,18E-03	339,1	-13,6	339,1	-13,6
oh2_4	450	1,57E-03	348,5	21,3	348,5	21,3
oh2_4	475	1,74E-03	339,2	-20	339,2	-20
oh2_4	500	2,00E-03	324,3	-28,6	324,3	-28,6
oh2_4	525	2,08E-03	323,7	-21	323,7	-21
oh2_4	550	1,21E-03	343,6	-32,6	343,6	-32,6
oh2_4	575	1,14E-03	350,7	-22,9	350,7	-22,9
oh2_4	600	4,17E-04	17,3	5,6	17,3	5,6
oh2_4	625	3,78E-04	27,4	21,2	27,4	21,2
oh2_4	650	4,78E-04	256,3	9,1	256,3	9,1
oh2_4	675	1,59E-03	321,7	-63,8	321,7	-63,8
S352	0	1,08E-02	98,956	36,225	181,5	7,8
S352	100	9,88E-03	103,61	31,562	185,9	12,1
S352	150	7,09E-03	98,659	32,51	185,3	7,8
S352	200	5,41E-03	92,591	35,103	182,9	2,7
S352	250	5,37E-03	92,869	35,02	183	2,9
S352	300	4,05E-03	95,277	38,851	179,1	4,7
S352	350	3,88E-03	93,479	31,88	186,1	3,5
S352	400	3,00E-03	105,76	45,689	171,4	11,7
S352	425	2,90E-03	90,225	28,421	189,6	0,7
S352	475	2,74E-03	94,217	37,34	180,6	4
S352	500	2,63E-03	99,97	42,17	175,5	8
S352	550	2,66E-03	105,02	43,246	173,9	11,6
S352	575	1,95E-03	112,34	37,567	178,5	18,2
S352	600	2,19E-03	106,99	37,307	179,6	14,1
S352	625	1,23E-03	186,73	16,591	105,3	73,1
S352	650	4,91E-04	311,91	62,958	107,3	-16,7
S352	675	6,83E-04	105,22	51,783	165,3	10,1
S372	0	6,90E-03	80,401	0,6646	168,2	-9,4
S372	100	7,30E-03	79,787	-4,2449	173	-10,6
S372	150	5,95E-03	71,596	-8,0193	176,1	-19,1
S372	200	5,37E-03	57,348	-4,5896	170,9	-32,9
S372	250	5,14E-03	53,644	-4,46	170,4	-36,6
S372	300	4,57E-03	46,253	-8,0476	174,4	-44,2
S372	350	4,54E-03	46,969	-4,1682	169,2	-43,2
S372	400	4,45E-03	43,452	0	162,7	-46,1
S372	425	4,39E-03	42,036	3,922	156,7	-46,6
S372	450	4,25E-03	42,992	4,3179	156,5	-45,6
S372	475	4,22E-03	39,806	2,1726	158,4	-49,2
S372	500	3,98E-03	37,938	4,8966	153,6	-50,3
S372	525	4,03E-03	41,882	0,99526	160,8	-47,5
S372	550	4,21E-03	35,617	1,4978	157,9	-53,5
S372	575	4,00E-03	36,87	2,2906	157,1	-52
S372	600	2,88E-03	37,036	17,141	136,2	-46,2
S372	625	1,05E-03	6,3775	-14,019	218,8	-80,6
S372	650	1,10E-03	262,45	36,953	26,4	10,2
oh2_2	0	1,97E-02	170,8	0,2	170,8	0,2
oh2_2	100	1,77E-02	169,5	0,4	169,5	0,4
oh2_2	150	1,49E-02	170,8	-2,9	170,8	-2,9
oh2_2	200	1,30E-02	168,6	-3,3	168,6	-3,3
oh2_2	225	1,24E-02	170,6	-4	170,6	-4
oh2_2	250	1,15E-02	171	-5,8	171	-5,8
oh2_2	275	1,05E-02	174,4	-7,5	174,4	-7,5
oh2_2	300	9,25E-03	172,2	-6,7	172,2	-6,7
oh2_2	325	8,36E-03	172,7	-9	172,7	-9
oh2_2	350	7,94E-03	171,9	-9,2	171,9	-9,2
oh2_2	375	7,26E-03	174,6	-13,1	174,6	-13,1
oh2_2	400	6,92E-03	173,7	-9,9	173,7	-9,9
oh2_2	425	6,54E-03	175,8	-10,2	175,8	-10,2
oh2_2	450	6,00E-03	169,9	0,7	169,9	0,7
oh2_2	475	6,03E-03	181,2	-20,4	181,2	-20,4
oh2_2	500	5,58E-03	173,8	-14,9	173,8	-14,9
oh2_2	525	4,70E-03	178,6	-19	178,6	-19
oh2_2	550	6,38E-03	173,5	-22,6	173,5	-22,6
oh2_2	575	4,14E-03	187,3	-11,6	187,3	-11,6
oh2_2	600	4,02E-03	190,8	-33,5	190,8	-33,5
oh2_2	625	2,89E-03	179	-26,3	179	-26,3
oh2_2	650	3,63E-03	205,1	-24,2	205,1	-24,2
oh2_2	675	6,48E-03	261,2	7,2	261,2	7,2
oh2_5	0	4,42E-03	204,5	14,3	204,5	14,3
oh2_5	100	4,08E-03	207,6	14,4	207,6	14,4
oh2_5	150	3,77E-03	206,6	2,2	206,6	2,2
oh2_5	200	3,47E-03	212,2	9,4	212,2	9,4
oh2_5	225	3,02E-03	212,6	6,2	212,6	6,2
oh2_5	250	2,94E-03	211,1	5,6	211,1	5,6
oh2_5	275	2,80E-03	208,7	10,3	208,7	10,3
oh2_5	300	2,40E-03	210,4	10,9	210,4	10,9
oh2_5	325	2,28E-03	211,2	15,8	211,2	15,8

Table S2. Thermal demagnetization data.

(1) Sample = sample name
(2) Temperature (°C)
(3) NRM intensity (A/m)
(4) Core Declination (°E)
(5) Core Inclination (°)
(6) Geographic Declination (°E)
(7) Geographic Inclination (°)

oh2_5	350	2,14E-03	217,2	6,9	217,2	6,9
oh2_5	375	1,99E-03	217,6	7,4	217,6	7,4
oh2_5	400	1,67E-03	209	12,9	209	12,9
oh2_5	425	1,54E-03	212,2	1,9	212,2	1,9
oh2_5	450	1,49E-03	227,4	-30,4	227,4	-30,4
oh2_5	475	1,39E-03	201,6	5,6	201,6	5,6
oh2_5	500	1,27E-03	196,4	4,2	196,4	4,2
oh2_5	525	2,04E-03	200,6	25,9	200,6	25,9
oh2_5	550	1,31E-03	213,3	42	213,3	42
oh2_5	575	1,56E-03	166,7	44,7	166,7	44,7
oh2_5	600	7,31E-04	179	-16,3	179	-16,3
oh2_5	625	1,84E-03	144,3	53,8	144,3	53,8
oh2_5	650	1,35E-03	81,1	85,5	81,1	85,5
oh2_5	675	1,70E-03	214,6	64,1	214,6	64,1
S386	0	7,43E-03	87,982	6,183	161,6	-3,6
S386	100	7,45E-03	89,846	5,3893	161,9	-1,6
S386	150	4,97E-03	78,437	10,43	159,9	-13,9
S386	200	5,09E-03	76,929	8,3518	162,4	-14,8
S386	250	3,36E-03	78,791	6,3143	163,9	-12,5
S386	300	3,44E-03	73,333	10,552	161	-18,8
S386	350	3,00E-03	69,845	18,651	153,3	-23,7
S386	400	3,04E-03	68,235	18,222	154	-25,1
S386	450	2,93E-03	62,751	26,382	145,5	-31,1
S386	475	2,80E-03	62,534	24,018	148,2	-31,1
S386	500	1,93E-03	64,538	23,999	148,1	-29,3
S386	525	2,71E-03	69,775	26,472	144,8	-24,9
S386	550	2,66E-03	59,927	32,917	137,8	-33,7
S386	575	2,83E-03	43,819	14,506	162,9	-47,8
S386	600	2,05E-03	50,346	18,937	155,7	-42
S386	625	2,61E-03	48,932	18,643	156,2	-43,4
S386	650	2,63E-03	61,631	30,145	141,1	-32,2
S386	675	7,16E-04	49,588	-15,637	197,5	-31,7
oh2_1	0	1,14E-02	155,1	15,4	155,1	15,4
oh2_1	100	1,05E-02	156,5	12,6	156,5	12,6
oh2_1	150	8,77E-03	158,1	12,1	158,1	12,1
oh2_1	200	7,68E-03	154,4	10,1	154,4	10,1
oh2_1	225	7,07E-03	158,5	9,6	158,5	9,6
oh2_1	250	6,07E-03	153,9	8,8	153,9	8,8
oh2_1	275	5,53E-03	153,7	8	153,7	8
oh2_1	300	5,08E-03	151,5	12,8	151,5	12,8
oh2_1	325	4,47E-03	148,2	8,2	148,2	8,2
oh2_1	350	4,06E-03	149,3	6,6	149,3	6,6
oh2_1	375	3,70E-03	149,2	9,6	149,2	9,6
oh2_1	400	3,31E-03	145,6	10,6	145,6	10,6
oh2_1	425	3,19E-03	145,8	9,7	145,8	9,7
oh2_1	450	2,88E-03	157,1	-17,7	157,1	-17,7
oh2_1	475	2,51E-03	150,5	-0,9	150,5	-0,9
oh2_1	500	3,02E-03	145,3	25,6	145,3	25,6
oh2_1	525	1,86E-03	140	4,1	140	4,1
oh2_1	550	1,42E-03	163,3	47,1	163,3	47,1
oh2_1	575	1,26E-03	189,7	-45,9	189,7	-45,9
oh2_1	600	1,38E-03	131,8	32,2	131,8	32,2
oh2_1	625	9,04E-04	144,5	0,4	144,5	0,4
oh2_1	650	1,28E-03	57,8	-46,3	57,8	-46,3
oh2_1	675	9,45E-04	81,7	31,7	81,7	31,7
oh2_6	0	3,19E-04	324	-40,5	324	-40,5
oh2_6	100	2,91E-04	333,8	-24,4	333,8	-24,4
oh2_6	150	2,28E-04	307	-55,8	307	-55,8
oh2_6	200	2,94E-04	329	-47,2	329	-47,2
oh2_6	225	2,91E-04	330,3	-51,8	330,3	-51,8
oh2_6	250	2,52E-04	329,4	-43,9	329,4	-43,9
oh2_6	275	2,80E-04	342,2	-47,7	342,2	-47,7
oh2_6	300	1,91E-04	0,6	-24,9	0,6	-24,9
oh2_6	325	2,80E-04	345,5	-49,8	345,5	-49,8
oh2_6	350	2,56E-04	334,3	-46,1	334,3	-46,1
oh2_6	375	2,51E-04	357,2	-19,5	357,2	-19,5
oh2_6	400	2,45E-04	342	-50,9	342	-50,9
oh2_6	425	4,57E-04	285,3	-46,2	285,3	-46,2
oh2_6	450	1,42E-03	215,4	60,7	215,4	60,7
oh2_6	475	3,72E-04	305,6	-23	305,6	-23
oh2_6	500	2,65E-04	19,8	15,4	19,8	15,4
oh2_6	525	1,15E-03	233,9	-57,4	233,9	-57,4
oh2_6	550	9,81E-04	239,6	-55,1	239,6	-55,1
oh2_6	575	5,94E-04	238,9	4,3	238,9	4,3
oh2_6	600	1,17E-03	204,1	-78,9	204,1	-78,9
oh2_6	625	5,25E-04	198,7	1	198,7	1
oh2_6	650	1,84E-04	168,2	-1,4	168,2	-1,4
oh2_6	675	1,51E-03	94,9	-75,1	94,9	-75,1
S409	0	3,18E-03	127,66	29,577	123,9	31,5
S409	100	2,77E-03	118,97	22,299	134,4	26,2
S409	150	1,58E-03	101,14	34,884	124,5	8,5
S409	200	1,04E-03	133,57	56,899	95,1	21,2
S409	250	1,00E-03	152,05	47,115	93,2	36
S409	300	1,78E-03	177,54	23,442	75,4	65,4
S409	350	9,79E-04	187,63	15,892	46,3	71,5
S409	400	2,81E-04	120,5	47,715	107,9	19,2
S409	400	4,07E-04	199,47	72,406	64	15,6
S409	425	1,29E-03	159,94	24,897	105,5	57,6
S409	450	1,55E-03	247,29	60,587	42,6	10
S409	475	1,18E-03	259,74	52,077	32,6	5,5
S409	500	1,52E-03	231,99	43,643	30,7	25,7
S409	550	1,23E-03	256,27	53,501	34,4	7,3
S409	575	1,58E-03	276,24	44,264	24,4	-5,2
S409	600	1,69E-03	236,2	48,664	34,1	20,8
S409	625	1,02E-03	288,31	-30,186	308,3	-15,2
S409	650	5,92E-04	181,5	-25,735	253,2	65,2
S436	0	2,85E-03	71,501	5,6375	140,5	-17,4
S436	100	2,72E-03	60,096	-8,673	154,4	-30,6
S436	150	2,82E-03	47,056	-12,267	158,3	-43,5
S436	200	2,90E-03	49,376	-22,075	171,3	-40,5
S436	250	2,81E-03	53,606	-19,749	167,7	-36,9
S436	300	2,50E-03	44,912	-22,726	173,1	-44,5
S436	350	2,39E-03	40,938	-23,304	175,1	-47,9
S436	400	2,78E-03	31,122	-27,15	185,9	-54,9
S436	450	2,89E-03	24,744	-28,761	193,9	-58,8
S436	475	2,77E-03	36,69	-23,383	176,9	-51,6

S436	500	2,96E-03	48,25	-14,091	160,8	-42,3
S436	525	2,53E-03	40,32	-16,528	165,2	-49,8
S436	550	2,84E-03	44,883	-13,289	159,9	-45,6
S436	575	2,26E-03	40,31	-22,15	173,7	-48,8
S436	600	3,55E-03	41,898	-35,884	191,5	-42,7
S436	625	3,19E-03	37,809	-17,127	166,7	-52,1
S436	650	2,43E-03	17,763	-0,23621	126,2	-70,7
S436	650	8,64E-04	202,32	0,33168	311,1	66,5
S436	675	1,00E-03	38,279	-50,697	209,6	-36,9
S453	0	1,54E-03	141,82	61,17	77,7	21,3
S453	100	1,37E-03	136,84	58,955	81,2	21,2
S453	150	9,11E-04	126,54	51,841	91,1	20,7
S453	200	8,11E-04	98,242	44,452	104,2	5,2
S453	250	6,28E-04	105,56	58,534	89,5	7,2
S453	300	7,90E-04	57,636	51,56	93	-20,3
S453	350	6,49E-04	35,142	70,289	70,7	-17
S453	400	6,18E-04	252,55	27,049	357,4	15
S453	425	4,73E-04	229,96	37,342	14,3	30,1
S453	450	4,83E-04	154,28	28,038	97,4	51,9
S453	475	6,59E-04	53,276	76,2	70,2	-9,2
S453	500	5,51E-04	171,54	57,124	64,4	31,5
S453	550	1,53E-03	1,7993	68,057	59,7	-22,9
S453	575	7,12E-04	330,96	32,346	20,9	-48,4
S453	600	7,37E-04	13,285	66,327	64,8	-24
S453	625	7,67E-04	110,65	79,56	68,8	2,7
S453	650	3,06E-04	47,203	-16,106	171,3	-40,4
S453	675	1,58E-04	77,758	21,681	127,1	-11,7
S471	0	7,73E-04	307,2	20,441	353,1	-34,5
S471	100	7,51E-04	279,93	8,6577	336,8	-9,8
S471	150	6,62E-04	250,85	-16,763	310,3	18,3
S471	200	7,86E-04	269,92	-17,558	310,4	0,1
S471	250	7,74E-04	274,35	-27,725	300,2	-3,9
S471	300	8,43E-04	274,56	-29,962	298	-3,9
S471	350	8,36E-04	252,9	-21,985	305,1	15,8
S471	400	9,23E-04	269,4	-21,95	306	0,6
S471	450	1,02E-03	279,23	-14,844	313	-8,9
S471	475	1,02E-03	261,04	-14,185	313,6	8,7
S471	500	1,04E-03	263,56	-5,8558	322,1	6,4
S471	525	9,03E-04	261,74	-23,367	304,4	7,6
S471	550	6,52E-04	298,11	23,397	354,1	-25,6
S471	575	1,66E-03	278,54	-50,985	276,7	-5,4
S471	600	6,24E-04	245,18	-32,042	293,4	20,8
S471	625	6,22E-04	263,62	-32,912	294,9	5,3
S471	650	5,25E-04	257,15	10,198	338,5	12,6
S471	675	2,69E-04	113,63	-86,272	234,6	1,5
S486	0	1,09E-03	154,27	43,339	96,3	49,9
S486	100	7,79E-04	114,52	-2,7956	164,5	23,6
S486	150	6,48E-04	117,38	-34,307	197,3	16
S486	200	4,81E-04	117,96	-43,33	205,8	12,5
S486	250	4,81E-04	163,27	-37,382	229,6	40,1
S486	300	4,95E-04	74,148	-31,698	187,2	-18,7
S486	350	1,77E-04	86,332	-47,296	203,6	-9,8
S486	400	3,75E-04	67,949	-76,856	234,4	-14,7
S486	425	5,60E-04	154,4	-70,674	238,7	7,5
S486	450	4,51E-04	122,88	-74,837	234,3	-1,6
S486	475	7,50E-04	194,01	-39,58	260,8	38,7
S486	500	5,43E-04	246,13	-62,903	271,6	1,5
S486	525	7,03E-04	117,15	-75,563	234,2	-3,2
S486	550	5,68E-04	114,13	-57,452	217,5	4
S486	575	7,20E-04	120,14	-69,608	229,5	0,5
S510	0	1,28E-04	77,814	43,932	126,8	1,2
S510	100	1,94E-04	44,716	-35,76	208,8	-44,5
S510	150	2,62E-04	15,542	-47,88	244,3	-53,7
S510	200	3,33E-04	2,0214	-59,257	260,5	-44,7
S510	250	3,93E-04	43,264	-65,404	242,6	-30,9
S510	300	4,02E-04	36,781	-71,349	249,4	-28,5
S510	350	3,88E-04	306,32	-68,033	281,6	-26,1
S510	400	3,80E-04	343,63	-54,647	275,9	-47,4
S510	450	3,60E-04	325,45	-34,552	312,4	-52,7
S510	475	5,55E-04	309,95	-51,461	297,8	-35,3
S510	500	3,78E-04	311,16	-59,851	288,5	-32
S510	525	4,42E-04	314,82	-59,754	287,4	-33,6
S510	550	7,82E-04	337,93	18,402	47,5	-51
S510	575	7,62E-04	15,043	-5,0446	142,3	-72,7
S510	600	3,29E-04	282,99	-71,058	281,4	-17,4
S510	625	4,33E-04	10,036	-33,357	238,5	-68,6
S510	650	4,25E-04	309,88	-41,89	309	-38,7
S510	675	5,90E-04	12,48	-30,346	228,9	-70
S520	0	1,32E-03	209,34	56,629	46,1	43,7
S520	100	7,05E-04	247,01	43,708	19,4	27,5
S520	150	7,73E-04	259,25	33,699	9	17,6
S520	200	7,16E-04	255,26	33,977	8,9	20,9
S520	250	7,49E-04	254,74	29,853	4,4	20,9
S520	300	7,61E-04	264,64	23,869	359,9	11,2
S520	350	8,44E-04	266,23	17,302	353,7	8,2
S520	400	7,49E-04	267,26	18,198	354,9	7,5
S520	425	8,59E-04	273,26	10,73	349,2	-0,1
S520	450	7,55E-04	260,65	5,5521	340,8	10,5
S520	475	1,12E-03	249,83	4,8126	337,1	20,7
S520	500	6,50E-04	261,77	12,251	347,7	11,1
S520	525	5,90E-04	272,81	-14,138	325,1	-6,5
S520	550	9,74E-04	254,31	-5,4817	328,2	13,4
S520	575	6,95E-04	231,94	-6,5226	318,4	33,9
S520	600	9,82E-04	246,12	-6,6652	324,2	20,8
S520	625	6,28E-04	230,29	-31,19	293,4	22,5
S548	0	1,35E-03	189,04	4,8734	29,4	72,6
S548	100	1,00E-03	186,08	-2,4607	21,9	80,3
S548	150	8,07E-04	178,87	-19,779	234,8	80,2
S548	200	7,77E-04	197,52	-19,86	298,7	70,4
S548	250	7,75E-04	197,17	-22,698	291,2	69,2
S548	300	7,57E-04	200,5	-21,802	297,6	67,1
S548	350	7,51E-04	201,62	-15,929	313	68,1
S548	400	6,93E-04	203,65	-17,559	310,2	65,8
S548	450	7,64E-04	209,17	-7,7481	333,1	61,1
S548	475	6,72E-04	200,25	-20,459	300,4	67,9
S548	500	7,84E-04	205,48	-46,09	267,4	47,9

Table S2. Thermal demagnetization data.

(1) Sample = sample name
(2) Temperature (°C)
(3) NRM intensity (A/m)
(4) Core Declination (°E)
(5) Core Inclination (°)
(6) Geographic Declination (°E)
(7) Geographic Inclination (°)

S548	525	8,79E-04	186,52	-25,687	261,7	73,1
S548	550	5,69E-04	214,57	-8,9948	329,7	55,9
S548	575	7,94E-04	175,86	-29,412	230,3	70,2
S548	600	4,92E-04	219,39	-13,769	321,4	51,3
S548	625	8,10E-04	196,17	-14,946	312	73,5
S548	675	4,64E-04	314,73	17,053	345,2	-45,5
S565	0	4,06E-04	77,905	-17,65	171,8	-14,4
S565	100	7,08E-04	45,135	-32,159	191	-42,9
S565	150	5,99E-04	43,888	-24,052	180,4	-46
S565	200	5,23E-04	42,205	-14,047	165,9	-48,6
S565	250	4,84E-04	26,342	-18,295	177	-63,2
S565	300	4,19E-04	48,196	1,6422	145,1	-40,6
S565	350	5,56E-04	31,285	28,448	103,3	-41,1
S565	400	1,11E-03	42,51	25,063	114,9	-35,7
S565	425	6,54E-04	238,3	-35,183	293,3	18,8
S565	450	6,06E-04	8,4652	4,1625	97,2	-73,5
S565	475	5,76E-04	14,809	3,3834	114,5	-70,1
S565	500	5,74E-04	16,236	7,5131	109,3	-66,2
S565	550	3,29E-04	54,638	36,095	110,9	-21
S565	575	8,43E-04	21,505	11,289	111,7	-59,8
S565	600	5,97E-04	43,084	16,857	124,1	-39,6
S565	625	7,99E-04	13,942	23,077	88,3	-54,2
S565	650	4,19E-04	286,36	0,41026	339,3	-16
S598	0	5,21E-03	102,36	9,3916	189	13,3
S598	100	4,92E-03	97,502	4,2003	193,7	7,9
S598	150	2,64E-03	89,779	11,14	185,9	1,1
S598	200	2,34E-03	97,431	8,3393	189,5	8,3
S598	250	2,04E-03	97,765	11,319	186,6	8,9
S598	300	2,21E-03	90,528	10,954	186,2	1,8
S598	350	9,60E-04	114,65	8,3284	191,1	25,3
S598	400	5,15E-04	172,91	46,201	114,7	50,3
S598	400	5,25E-04	147,96	52,625	130,9	37,4
S598	425	8,07E-04	85,375	-20,445	216,9	-6,8
S598	450	6,58E-04	95,993	56,407	140,9	9,1
S598	450	3,61E-04	210,53	41,102	73,3	46,4
S598	475	6,43E-04	138,35	35,91	153,6	42,2
S598	500	7,68E-04	149,59	26,446	159,5	55,2
S598	550	6,28E-04	177,38	29,561	112,9	67,3
S598	575	4,35E-04	157,48	18,374	167,5	65,3
S598	600	4,13E-04	140,41	24,638	167,4	48,2
S598	625	3,65E-04	182,92	74,423	106,2	22,6
S598	650	4,05E-04	296,63	3,6838	24,6	-25,9
S605	0	9,57E-04	256,58	19,843	74,9	13,3
S605	100	9,00E-04	269,56	28,757	83,7	1,3
S605	150	1,16E-03	274,87	36,363	91,6	-2,7
S605	200	7,75E-04	294,8	60,905	118,4	-10
S605	250	1,05E-03	275,04	65,079	120,2	-0,3
S605	300	1,06E-03	264,01	32,185	87,2	6,1
S605	350	1,28E-03	288,08	55,357	111,9	-8,5
S605	400	1,24E-03	334,56	60,31	131,5	-24,6
S605	425	1,09E-03	286,96	9,4627	65,5	-16,4
S605	450	1,21E-03	294,02	51,261	109,1	-13,1
S605	475	1,04E-03	282,14	45,458	101,3	-7
S605	500	9,09E-04	269,46	45,935	100,9	1,8
S605	550	1,00E-03	276,24	43,492	98,8	-3,1
S605	575	1,13E-03	300,91	33,668	93,5	-24,1
S605	600	1,28E-03	290,66	39,218	96,5	-14,5
S605	625	1,35E-03	298,26	40,229	99,4	-19,8
S605	650	2,85E-04	334,56	36,694	116	-44,7
S625	0	1,18E-03	229,47	-55,916	357,2	21,4
S625	100	8,01E-04	226,1	-46,521	215,3	28,5
S625	150	5,88E-04	262,52	-70,144	349,7	2,5
S625	200	5,22E-04	260,13	-63,44	356,2	4,4
S625	250	5,86E-04	270,14	-47,213	12,8	-0,1
S625	300	3,22E-04	311,41	-10,926	45,6	-40,5
S625	350	8,06E-04	276,66	-62,64	357,2	-3,1
S625	400	4,09E-04	185,37	-19,735	338,4	67,6
S625	425	1,12E-03	238,08	-28,999	26,9	27,5
S625	450	4,32E-04	234,25	-22,024	33,5	32,8
S625	475	2,39E-04	279,46	-6,7315	53,2	-9,4
S625	500	4,17E-04	265,03	-67,176	352,7	1,9
S625	550	3,46E-04	135,13	-28,834	278	38,4
S625	575	5,30E-04	319,21	-35,147	12,9	-38,2
S625	600	5,21E-04	49,764	-51,584	298,8	-23,7
S663	0	7,70E-04	166,75	19,817	178,1	68
S663	100	6,63E-04	149,97	25,271	191,6	52,9
S663	150	6,70E-04	151,77	22,915	193,4	55,6
S663	200	5,89E-04	152,26	14,946	206,1	59,7
S663	250	5,27E-04	156,52	12,279	208	64,6
S663	300	5,42E-04	164,21	-4,5497	255,6	72,9
S663	350	5,79E-04	150,73	21,068	197,1	55,7
S663	400	4,53E-04	148,26	26,065	191,9	51,2
S663	425	4,34E-04	157,36	20,808	190,9	61
S663	450	5,52E-04	191,78	18,171	108,2	70,1
S663	475	5,92E-04	182,43	6,3025	113,6	85,1
S663	500	4,98E-04	198,07	27,502	110,5	59,2
S663	525	5,46E-04	219,48	38,742	103,6	38,6
S663	550	4,13E-04	162,36	27,42	175	59,5
S663	575	6,37E-04	220,36	8,2196	63,3	49,3
S709	0	1,07E-03	270,85	-79,036	333	-2,1
S709	100	8,08E-04	320,94	-70,74	334,5	-16,8
S709	150	7,00E-04	337,04	-42,878	352,5	-44,3
S709	200	7,53E-04	319,73	-40,312	0,2	-37,2
S709	250	7,30E-04	324,39	-41,548	356,2	-39,1
S709	300	7,13E-04	323,19	-36,799	1,8	-41,4
S709	350	9,16E-04	324,74	-38,313	359,2	-41,4
S709	400	7,94E-04	324,54	-36,813	0,9	-42,3
S709	450	7,32E-04	307,84	-23,695	24,2	-35,1
S709	475	5,25E-04	314,91	-30,313	13,7	-38,8
S709	500	8,19E-04	335,88	-16,319	19,5	-62,3
S709	525	1,02E-03	318,08	-14,603	32,7	-46,8
S709	550	1,12E-03	327,12	-10,778	35,5	-56,2
S709	575	2,33E-04	266,78	-72,238	339,7	-0,9
S709	600	5,85E-04	321,68	-30,303	10,1	-44
S709	625	9,53E-04	332,7	-18,287	18,9	-58,7
S709	650	8,45E-04	14,573	-27,257	294,4	-61,1
S709	675	6,43E-04	258,15	62,979	115,4	7,1
S762	0	7,83E-04	248,23	-3,2944	38,5	20,4
S762	100	4,16E-04	231,86	5,238	43,4	38,3
S762	150	4,89E-04	232,15	-1,7574	35	36,2
S762	200	6,40E-04	232,16	-12,552	22,8	32,3
S762	250	6,27E-04	259,05	2,7419	47,2	11,3
S762	300	6,64E-04	260,81	-2,8472	42,1	8,3
S762	350	7,09E-04	261,86	18,85	63,9	11,7
S762	400	8,11E-04	267,62	26,83	72,8	7,9
S762	425	1,25E-03	265,52	39,139	84,9	11,6
S762	450	6,31E-04	252,95	11,984	190,2	12,9
S762	450	2,78E-03	96,311	38,232	55,4	19
S762	475	6,91E-04	282,46	16,478	66	-7,9
S762	500	7,04E-04	269,05	19,076	65,4	5,1
S762	550	6,47E-04	272,21	16,068	63,1	1,5
S762	575	8,17E-04	223,33	33,764	82,4	45,6
S762	600	9,00E-04	284,41	9,3373	59,6	-11,7
S762	625	8,04E-04	289,78	22,681	74,2	-12,6
S762	650	2,17E-04	3,4602	7,6681	146,6	-69
S762	675	3,17E-04	46,548	7,796	205,4	-39,3
S810	0	5,80E-04	324,78	-48,691	326,9	-44
S810	100	5,31E-04	343,96	-37,076	323	-62
S810	150	4,73E-04	357,93	-20,303	309,9	-82,4
S810	200	4,84E-04	353,93	-12,64	27,8	-84,1
S810	250	5,05E-04	358,82	-16,316	313,9	-86,5
S810	300	4,42E-04	2,0309	-16,826	268,1	-85,7
S810	350	3,56E-04	2,5448	-27,623	286,1	-75,2
S810	400	5,45E-04	350,61	-11,761	31,7	-80,7
S810	450	5,57E-04	316,04	-12,76	20,2	-47,2
S810	475	4,23E-04	332,24	30,266	83,8	-39,1
S810	500	5,58E-04	9,8569	-10,018	188,9	-79,9
S810	525	7,61E-04	19,071	-30,643	253,1	-65,1
S810	550	7,23E-04	355,07	18,296	106,1	-58,3
S810	575	5,20E-04	321,98	-19,91	9,1	-53
S810	600	8,05E-04	10,399	-35,714	274,4	-65,4
S810	625	3,53E-04	9,9792	-11,43	196,9	-80,1
S810	650	7,63E-04	325,93	4,8851	50,7	-51,7
S810	675	1,57E-04	120,2	56,613	147,3	27,2
S863	0	6,15E-04	90,194	-16,521	223,4	-5,9
S863	100	4,68E-04	115,29	-25,052	241,3	11,6
S863	150	4,80E-04	120,92	-14,467	234,7	21,6
S863	200	2,97E-04	117,5	-21,976	239,7	14,9
S863	250	3,69E-04	132,18	-20,766	236,5	38
S863	300	3,17E-04	33,286	29,867	151	-29,1
S863	350	7,16E-04	131,81	-50,297	269,4	6,1
S863	400	6,69E-04	153,94	-52,962	282,3	11,7
S863	425	3,56E-04	127,97	24,587	194,2	42,4
S863	425	3,80E-04	130,79	27,457	190,6	45,2
S863	450	1,39E-04	63,104	-56,298	261,8	-33
S863	475	3,14E-04	72,374	2,3728	198,9	-15,4
S863	500	1,24E-04	16,684	-53,549	280,2	-56
S863	550	3,83E-04	56,894	-5,8473	200,5	-32,8
S863	575	8,55E-04	87,996	14,012	194,3	3,4
S863	600	2,62E-04	136,58	-28,002	256,1	24,8
S939	0	7,38E-04	165,7	-3,2624	273,2	60,3
S939	100	6,76E-04	143,89	-10,837	252,8	41,1
S939	150	4,47E-04	121,52	-18,247	243,8	19,6
S939	200	4,57E-04	113,65	-10,85	233	16,8
S939	250	4,28E-04	122,09	3,3485	223,3	30,7
S939	300	5,03E-04	107,78	7,8919	212,5	19,4
S939	350	6,14E-04	125,92	-1,6801	230,6	31,9
S939	400	7,78E-04	105,75	-15,893	233,9	7,7
S939	450	6,19E-04	127,85	-0,46258	230,4	34,2
S939	475	6,17E-04	133,1	-14,944	248,1	30,5
S939	500	6,24E-04	98,587	-14,955	230,1	1,8
S939	525	8,97E-04	165,23	40,454	155,1	68,6
S939	550	7,32E-04	145,66	-4,3068	247,8	46,8
S939	575	9,77E-04	102,72	18,384	200,3	18,4
S939	600	6,80E-04	136,91	-4,4712	240,6	39,8
S939	625	6,71E-04	119,5	8,1411	217	30,3
S939	650	6,15E-04	127,52	-2,4246	232,2	32,9
S939	675	4,09E-04	165,21	4,6299	262,9	66,7
S987	0	1,08E-03	201,89	26,94	64,6	68,4
S987	100	4,49E-04	196,97	-20,316	332,8	47,3
S987	150	1,47E-04	185,67	-15,839	318,5	54,7
S987	200	4,19E-04	190,44	-8,926	330,1	60,2
S987	250	7,29E-05	191,8	-34,335	321	35,5
S987	300	1,03E-04	118,7	27,057	198,6	33,5
S987	350	4,17E-04	238,88	63,529	102,7	30,6
S987	400	5,11E-04	146,22	-5,2763	251,8	48,8
S987	400	3,56E-04	211,7	47,645	94,3	51,5
S987	425	3,30E-04	252,29	-40,714	355,2	0,3
S987	475	3,15E-04	203,42	10,793	22,4	66
S987	500	3,91E-04	196,03	15,436	28,4	74,3
S987	550	3,44E-04	161,34	44,06	156,8	60,5
S987	575	1,32E-04	184,72	51,333	123,5	57,5
S987	600	3,81E-04	200,4	-6,0287	349,7	57,9
S1044	0	1,10E-03	242,74	-25,888	18,3	16
S1044	100	8,52E-04	264,78	-30,143	21,6	-3,6
S1044	150	7,89E-04	268,58	-39,797	13,1	-9,1
S1044	200	6,61E-04	277,26	-41,998	12,1	-15,9
S1044	250	7,31E-04	280,69	-41,87	12,5	-18,5
S1044	300	7,54E-04	282,24	-38,629	16	-19,3
S1044	350	7,70E-04	279,38	-42,867	11,3	-17,6
S1044	400	7,02E-04	283,38	-31,893	23,2	-19,5
S1044	450	6,32E-04	307,91	-41,263	11,5	-38,7
S1044	475	5,84E-04	291,4	-30,884	25	-26,3
S1044	500	6,63E-04	291,76	-33,919	21,6	-26,7
S1044	525	5,75E-04	314,63	-19,494	40,4	-46,8
S1044	550	5,87E-04	278,85	-49,177	4,7	-17,8
S1044	575	4,61E-04	274,03	-22,053	31,6	-9,6
S1044	600	5,19E-04	283,83	-38,607	16,1	-20,6
S1044	625	6,60E-04	295,63	-28,9	27,5	-29,8
S1044	650	5,84E-04	317,97	-47,305	0,7	-43,4
S1044	675	6,53E-04	37,852	-10,672	228,6	-52,8
S1099	0	7,98E-04	235,71	26,266	70,8	37,1

Table S2. Thermal demagnetization data.

(1) Sample = sample name
(2) Temperature (°C)
(3) NRM intensity (A/m)
(4) Core Declination (°E)
(5) Core Inclination (°)
(6) Geographic Declination (°E)
(7) Geographic Inclination (°)

S1099	100	6,48E-04	307,77	15,759	77,6	-29,9
S1099	150	6,33E-04	308,66	13,713	76	-31,7
S1099	200	5,05E-04	329,69	8,3186	84,8	-52
S1099	250	5,35E-04	330,72	8,1657	85,7	-52,9
S1099	300	4,86E-04	335,71	5,5485	87,7	-58,4
S1099	350	4,68E-04	333,27	-13,333	49,3	-64
S1099	400	5,03E-04	306,09	-16,028	40,2	-38,2
S1099	425	6,54E-04	319,34	0,43815	66,4	-47
S1099	450	4,26E-04	321,19	-14,257	45	-52,5
S1099	475	5,84E-04	313,98	-28,4	23,5	-45,5
S1099	500	6,22E-04	302,14	-36,249	15	-34,6
S1099	525	7,53E-04	286,21	-25,989	26,5	-20,8
S1099	550	4,17E-04	265,46	-20,336	28,2	-1
S1099	575	5,73E-04	258,48	-23,543	23,3	4,2
S1099	600	4,78E-04	282,12	-29,879	21,9	-17,7
S1099	625	3,67E-04	203,2	-29,232	345,8	40,4
S1171	0	4,32E-04	115,5	33,575	190,7	24,5
S1171	100	4,40E-04	113,43	26,894	198,2	23,6
S1171	150	5,10E-04	105,17	16,407	209,6	16,2
S1171	200	4,84E-04	104,43	19,055	206,9	15,6
S1171	250	4,72E-04	106,56	10,995	215,3	17,3
S1171	300	4,70E-04	111,42	26,841	198,5	21,8
S1171	350	3,60E-04	128,56	-3,3444	234	37,8
S1171	400	4,27E-04	112,79	20,419	205,3	23,4
S1171	450	4,35E-04	117,29	35,605	188,2	25,6
S1171	475	3,68E-04	145,84	42,723	169	42,5
S1171	500	4,51E-04	116,15	46,453	176,9	22,2
S1171	525	3,27E-04	138,19	48,715	167,3	34,6
S1171	550	4,51E-04	163,5	60,051	144,9	34,5
S1171	575	3,93E-04	151,77	30,721	178,5	53,8
S1171	600	5,51E-04	110,52	-25,705	253,8	15,6
S1171	625	5,81E-04	297,39	80,735	126,8	1,7
S1171	650	3,23E-04	236,75	76,962	123,8	13
S1171	675	1,13E-04	13,034	14,341	167,3	-65,9
S1238	0	2,72E-04	350,28	47,585	109,2	-32,8
S1238	100	3,55E-04	351,55	38,127	108	-42,2
S1238	150	3,34E-04	359,45	51,205	116,6	-29,8
S1238	200	3,93E-04	7,9243	45,45	123,8	-35,1
S1238	250	3,81E-04	349,11	23,655	99,1	-55,7
S1238	300	3,98E-04	347,01	39,616	103,9	-39,9
S1238	350	2,74E-04	275,13	55,157	82,2	4,5
S1238	400	9,27E-04	335,01	75,813	111	-3,9
S1238	425	2,53E-04	284,42	0,22649	29,5	-14,2
S1238	450	3,98E-04	299,86	62,046	92,9	-5,3
S1238	475	6,16E-04	325,45	46,385	91,1	-26,6
S1238	500	7,44E-04	339,47	51,92	103	-26,6
S1238	550	4,75E-04	324,59	40,584	86,2	-30,6
S1281	0	4,24E-04	65,818	20,275	199,2	-18,1
S1281	100	4,90E-04	62,613	17,459	200,9	-21,9
S1281	150	5,72E-04	53,835	14,997	200,1	-30,7
S1281	200	5,92E-04	60,984	13,867	204	-24,6
S1281	250	5,28E-04	62,34	13,015	205,4	-23,6
S1281	300	5,43E-04	61,692	12,774	205,4	-24,3
S1281	350	5,35E-04	64,316	11,966	207,1	-22,1
S1281	400	5,51E-04	64,007	26,618	192,4	-17,4
S1281	450	5,13E-04	57,837	13,178	203,6	-27,7
S1281	475	4,69E-04	35,495	5,8718	201,2	-50,8
S1281	500	5,64E-04	25,044	26,223	167,8	-45,5
S1281	525	6,60E-04	28,59	4,5168	197,9	-57,6
S1281	550	3,59E-04	68,824	5,2796	215,3	-19,6
S1281	575	4,76E-04	51,746	13,115	201,2	-33,3
S1281	600	1,98E-04	86,077	27,676	197,1	1,7
S1281	625	2,05E-04	46,763	51,878	162,8	-15,4
S1281	650	5,54E-04	59,017	22,632	194,4	-23,1
S1281	675	1,48E-04	165,43	-34,479	298,7	42,5
S1309	0	2,97E-04	212,72	-5,7877	8,4	45,7
S1309	100	2,98E-04	228,01	-74,291	330	-14
S1309	150	3,16E-04	29,745	-87,079	316,4	-27,5
S1309	200	3,14E-04	25,641	-79,824	312,7	-34,1
S1309	250	2,65E-04	28,413	-74,842	308,9	-38,1
S1309	300	2,91E-04	104,88	-76,83	304,4	-21
S1309	350	2,52E-04	71,565	-82,791	310,3	-27,1
S1309	400	2,04E-04	181,97	-73,506	318,6	-8,5
S1309	425	2,17E-04	62,7	-80,741	308,6	-28,9
S1309	450	1,67E-04	194,43	-32,655	332,1	30,7
S1309	475	2,68E-04	300,4	-71,033	337,6	-33,3
S1309	500	3,51E-04	242,51	-19,444	17,9	14,7
S1309	525	3,93E-04	315,12	-34,015	24,1	-50,2
S1353	0	1,84E-04	149,23	-18,584	276,9	44,8
S1353	100	4,01E-04	116,27	64,27	165,4	24,8
S1353	150	3,77E-04	76,122	45,895	182,5	1,4
S1353	200	2,31E-04	74,784	55,854	172,9	4,1
S1353	250	3,23E-04	47,346	-24,525	249,8	-44,7
S1353	300	7,28E-04	11,388	5,6772	169,4	-66,4
S1353	350	4,27E-04	243,17	-24,168	18,3	17
S1353	400	6,17E-04	90,928	-45,522	274,7	-10
S1353	425	5,28E-04	322,79	-39,638	5,5	-49,2
S1353	450	2,13E-04	16,798	58,685	148,9	-15
S1353	475	1,49E-04	351,25	57,94	135,2	-16,7
S1353	500	2,78E-04	344,34	-36,921	349,5	-64
S1353	525	3,12E-04	345,47	3,8637	101,5	-66,3
S1353	550	5,66E-04	182,01	40,968	136,5	64
S1353	575	3,21E-04	33,838	47,84	163,5	-20,3
S1375	0	6,40E-04	211,98	-30,915	353,2	25,4
S1375	100	4,92E-04	232,25	-56,074	349,2	-3,2
S1375	150	4,04E-04	222,98	-63,508	340,8	-5,7
S1375	200	4,23E-04	236,71	-62,032	346,4	-9
S1375	250	3,92E-04	230,6	-62,497	344,1	-7,2
S1375	300	3,70E-04	250,65	-61,208	350,9	-13,9
S1375	350	3,56E-04	276,64	-57,701	358,9	-25,2
S1375	400	3,27E-04	263,53	-64,311	350,4	-20,6
S1375	450	2,58E-04	290,27	-43,332	16,4	-31,8
S1375	475	4,35E-04	265,24	-46,207	358,8	-4,7
S1375	500	4,53E-04	257,21	-68,346	345,4	-19,5
S1375	525	5,75E-04	281,13	13,468	70	-3,8
S1375	550	4,68E-04	343,97	-14,613	87,1	-71,2
S1375	575	4,10E-04	326,1	-25,121	47,1	-59,5
S1375	600	2,26E-04	313,36	-8,9266	65,4	-42,7
S1375	625	4,42E-04	214,41	-41,523	349	15,3
S1375	650	7,26E-04	298,12	-11,683	54,3	-30,2
S1375	675	1,07E-04	84,652	-65,759	296	-25,7
S1436	0	6,34E-04	242,8	-67,49	349,2	-8,7
S1436	100	2,26E-04	292,04	-38,92	24,8	-29,3
S1436	150	2,56E-04	262,23	-17,498	39,9	1
S1436	200	3,52E-04	286,4	-33,423	30,4	-24,2
S1436	250	5,04E-04	343,17	-64,842	338,8	-43,8
S1436	300	5,04E-04	254,07	-12,716	41,4	10,2
S1436	350	5,49E-04	317,01	-72,919	342,6	-31,9
S1436	400	5,94E-04	299,53	8,0289	78,1	-24,3
S1436	425	3,28E-04	220,99	-39,419	1,5	19,3
S1436	450	4,28E-04	299,05	-30,001	35,7	-34,5
S1436	475	2,73E-04	316,23	-32,776	31,6	-49,1
S1436	500	1,94E-04	305,98	-32,497	32,8	-40,5
S1436	550	2,35E-04	290,07	-8,056	58	-21,5
S1436	575	3,75E-04	291,83	-4,2838	62,5	-22
S1436	600	2,53E-04	320,55	10,704	93,8	-40,5
S1436	625	8,45E-05	233,13	-52,452	358,3	4,2
S1525	0	1,37E-03	195,66	10,324	5	63,1
S1525	100	6,50E-04	180,35	-5,0346	329,6	52
S1525	150	5,96E-04	158,52	8,1034	285,6	58,1
S1525	200	1,24E-04	120,05	3,1486	253,7	26,7
S1525	250	2,24E-04	122,93	-13,606	269,7	18,4
S1525	300	2,17E-04	64,29	-34,158	259,3	-37,4
S1525	350	1,80E-04	146,68	-25,118	296,1	23,8
S1525	400	1,53E-04	92,265	-57,947	292,7	-26,4
S1525	450	2,28E-04	108,43	-55,265	294,5	-17,2
S1525	475	2,88E-04	350,85	-65,478	336	-57,1
S1525	500	3,40E-04	118,98	32,53	221,8	39,5
S1525	525	1,12E-03	166,7	-74,446	325,3	-17,8
S1525	550	2,46E-04	84,13	-29,153	261,5	-19,9
S1525	575	5,88E-04	131,16	-9,4875	272,5	27
S1525	600	3,84E-04	202,21	-41,372	345,9	12,9
S1525	625	3,14E-04	212,01	-21,466	2,8	27,6
S1525	650	5,24E-04	74,34	-61,701	294,7	-35,9
S1525	675	4,64E-04	266,2	-44,413	18,3	-20
S1569	0	3,59E-03	184,48	-38,35	332,4	37,5
S1569	100	1,60E-03	186,94	-52,316	332,6	23,4
S1569	150	1,48E-03	52,643	-48,124	287,6	-35
S1569	200	2,05E-03	167,43	-45,527	317,9	29,4
S1569	250	1,05E-03	148,83	-46,785	305,3	23,1
S1569	300	1,78E-03	116,88	-30,755	275,6	14,7
S1569	350	1,01E-03	345,2	-63,406	336,5	-39,5
S1569	400	1,54E-03	51,756	-70,403	311	-25,4
S1569	425	1,31E-03	254,49	-49,784	6,5	-1
S1569	450	6,69E-04	14,83	-40,643	304,9	-60,4
S1569	475	7,86E-04	42,332	-59,849	303,7	-34,7
S1569	500	1,03E-03	60,601	-64,067	303,1	-25,2
S1569	550	8,82E-04	44,268	-46,338	288,4	-40,9
S1569	575	1,20E-03	55,244	-84,271	323,1	-17,2
S1569	600	7,71E-04	69,896	-54,757	292	-23
S1569	625	9,50E-04	68,153	-60,888	298,7	-22,8
S1569	650	2,59E-04	6,2178	2,2152	169,3	-72,7
S1589	0	8,68E-04	200,44	14,824	29,3	68,7
S1589	100	8,00E-04	201,67	45,526	107,2	62,3
S1589	150	7,71E-04	205,89	46,231	104,4	59,6
S1589	200	8,30E-04	214,16	42,428	92,6	56,3
S1589	250	4,50E-04	224,78	34,536	76,8	49,9
S1589	300	7,19E-04	195,63	44,094	112,4	66,2
S1589	350	2,07E-04	201,04	61,952	125,9	49,5
S1589	400	7,95E-04	220,38	69,314	123,9	38,8
S1589	425	6,79E-04	190,32	50,738	127	62,1
S1589	450	5,03E-04	216,37	23,303	57,3	56,8
S1589	475	6,13E-04	220,57	48,913	99,6	49,7
S1589	500	6,45E-04	212,6	50,706	106,1	53,3
S1589	550	7,33E-04	221,28	45,646	94,6	50,4
S1589	575	4,05E-04	176,27	46,942	147,5	66,9
S1589	600	7,13E-04	191,18	43,648	118,7	68,3
S1599	0	1,18E-02	202,62	-19,041	359,7	40,8
S1599	100	3,06E-03	217,65	16,536	54,9	53,9
S1599	150	3,04E-03	216,07	8,5132	41,7	51,9
S1599	200	1,54E-03	254,87	35,701	90,3	26
S1599	250	1,50E-03	259,25	35,645	91,2	22,6
S1599	300	1,38E-03	258,51	36,373	91,8	23,3
S1599	350	1,34E-03	273,57	49,724	108,7	16,6
S1599	400	1,02E-03	278,9	36,027	97,3	7,8
S1599	425	1,90E-03	279,43	29,971	92	4,7
S1599	450	1,54E-03	271,04	41,307	99,8	15,5
S1599	475	1,28E-03	283,65	43,891	106	8
S1599	500	1,48E-03	284,28	31,876	95,6	1,9
S1599	525	1,45E-03	293,4	7,9048	78,7	-17,4
S1599	550	1,16E-03	301,33	40,503	110,3	-4,8
S1599	575	1,31E-03	312,17	37,991	114,2	-12,7
S1599	600	8,88E-04	256,27	44,216	99,8	26,7
S1634	0	2,01E-02	200,37	-33,391	356,8	35,1
S1634	100	5,72E-03	193,52	6,2211	25,9	72,3
S1634	150	4,51E-03	300,79	49,85	122,3	-4,5
S1634	200	4,26E-03	143,28	24,522	228,7	55,3
S1634	250	3,84E-03	129,26	48,444	197,4	39,1
S1634	300	6,54E-03	197,98	12,716	51,6	71,9
S1634	350	1,36E-03	180,59	-7,4998	330,3	62,5
S1634	400	4,18E-03	180	77,991	156	30
S1634	425	3,72E-03	180,78	78,689	155,8	29,3
S1634	450	2,53E-03	195,03	63,774	147	43,1
S1634	475	1,30E-03	203,28	34,373	108,9	63,6
S1634	500	1,16E-03	164,66	69,523	162,7	37,6
S1634	550	1,14E-03	40,397	76,189	165	7,3
S1634	575	1,04E-03	243,43	84,815	151,1	20,3
S1634	600	9,98E-04	52,181	68,729	172,7	4,4
S1634	625	1,68E-03	52,846	77,569	166	10,3
S1634	650	1,05E-03	1,3744	8,0816	159,1	-63,9
S1701	0	1,80E-02	194,31	0,19056	13,8	67,3
S1701	100	7,29E-03	193,28	-11,632	358,8	57,6

Table S2. Thermal demagnetization data.

(1) Sample = sample name
(2) Temperature (°C)
(3) NRM intensity (A/m)
(4) Core Declination (°E)
(5) Core Inclination (°)
(6) Geographic Declination (°E)
(7) Geographic Inclination (°)

S1701	150	3,28E-03	264,69	-55,953	8,7	-11,9
S1701	200	1,46E-03	190,41	-26,451	347,1	44,4
S1701	250	4,62E-03	216,52	-55,952	353,8	9,9
S1701	300	4,94E-03	180,94	-42,535	334,8	29,5
S1701	350	4,12E-03	172,5	-46,532	328,3	25,1
S1701	400	4,12E-03	251,9	-82,363	341,5	-15,5
S1701	425	1,82E-03	222,68	-21,308	22,6	32,6
S1701	450	8,45E-04	212,87	31,533	94,8	57,4
S1701	475	7,96E-04	187,37	-21,531	344,7	49,8
S1701	500	4,40E-04	208,08	-27,918	5,2	36,6
S1701	550	3,19E-04	141,25	17,12	239,3	53,1
S1701	575	6,18E-04	267,67	6,1302	69,1	4,1
S1701	600	4,29E-04	224,37	41,659	105,8	45,5

OH3-B

3BOH30	0	1,22E-03	197,08	48,95	331,3	44,7
3BOH30	100	7,00E-04	204,6	21,617	292,7	61,5
3BOH30	150	3,64E-04	157,43	17,761	46,9	65
3BOH30	200	4,61E-04	203,09	24,192	298,8	61,3
3BOH30	250	3,18E-04	116,65	17,74	60,1	27,2
3BOH30	300	2,25E-04	123,23	72,047	2,5	15,5
3BOH30	350	3,16E-04	244,01	64,794	323,5	16,3
3BOH30	400	3,90E-04	45,327	17,633	48,3	-39,4
3BOH30	425	3,47E-04	322,45	-21,855	229,6	-50,4
3BOH30	450	3,96E-04	224,03	-77,831	175,4	2,8
3BOH30	475	2,11E-04	265,64	1,6319	258,2	4,5
3BOH30	500	1,56E-04	296,43	14,306	275,5	-23,8
3BOH30	550	1,33E-04	289,61	3,3747	262,7	-19,1
3BOH30	575	3,58E-04	168,35	33,946	6,6	60
3BOH30	600	3,52E-04	261,8	20,478	276,8	9,8
3BOH30	625	1,04E-03	40,537	-73,801	156	-18,1
3BOH57	0	9,30E-04	189,5	67,5	337,2	17,2
3BOH58	100	5,72E-04	238,6	74,4	327,7	3,2
3BOH59	150	2,66E-04	334,8	54,4	323	-36,6
3BOH60	200	2,09E-04	328,4	68,6	329	-23
3BOH61	250	2,47E-04	333	62,4	327	-29,2
3BOH62	300	2,50E-04	329,3	67,9	328,9	-23,8
3BOH63	350	3,48E-04	204,9	74,5	334,5	9
3BOH64	400	2,40E-04	314,4	56,8	314,9	-27
3BOH65	425	3,58E-04	12,5	34,5	0,9	-58,3
3BOH66	450	4,62E-04	301	41	294,7	-26,4
3BOH67	475	3,96E-04	319,5	31,8	291,3	-43,6
3BOH68	500	3,12E-04	310,5	29,8	285	-37,2
3BOH69	525	5,80E-04	301,9	46,2	300,4	-25,3
3BOH70	550	3,16E-04	310,5	-3,6	242,1	-39,8
3BOH71	575	4,99E-04	310,2	4,1	252,2	-40,4
3BOH72	600	6,68E-04	306,5	14,3	265,1	-36,6
3BOH73	625	3,63E-04	316,7	44,6	304,3	-35,3
3BOH94	0	1,02E-03	225,39	43,696	330	28,9
3BOH94	100	6,51E-04	236,22	56,066	337,1	16,3
3BOH94	150	5,69E-04	241,65	54,468	334,1	14,3
3BOH94	200	4,31E-04	253,93	73,441	350,1	2,6
3BOH94	250	4,02E-04	315,91	70,574	352,1	-15,8
3BOH94	300	3,23E-04	300,21	55,498	335	-18,3
3BOH94	350	3,47E-04	353,2	59,256	1,9	-32,5
3BOH94	400	4,20E-04	332,29	74,564	358,6	-15,6
3BOH94	450	3,82E-04	316,25	20,845	303,2	-43,4
3BOH94	475	6,75E-04	331,56	58,277	349,3	-29,5
3BOH94	500	6,33E-04	314,02	66,995	348,8	-17,7
3BOH94	525	1,11E-03	338,34	13,976	306,4	-65,5
3BOH94	550	3,94E-04	337,07	39,756	340	-46,9
3BOH94	575	6,85E-04	3,3558	22,669	14,7	-69,1
3BOH94	600	4,90E-04	355,66	30,269	358,2	-61,4
3BOH94	625	2,31E-04	301,01	-0,74403	273,9	-31
3BOH94	650	9,25E-04	227,03	52,822	337,4	22,6
3BOH94	675	2,89E-04	309,17	15,257	293,9	-38,2
3BOH136	0	1,67E-03	219,31	58,211	330,6	36
3BOH136	100	9,69E-04	218,32	28,484	294,6	51,2
3BOH136	150	9,87E-04	215,71	30,572	298,9	52,7
3BOH136	200	8,38E-04	232,25	21,634	282,5	39,6
3BOH136	250	4,23E-04	282,51	22,062	289,4	-6,4
3BOH136	300	4,37E-04	252,53	10,286	271,5	19,1
3BOH136	350	4,16E-04	210,06	14,462	271,4	60,8
3BOH136	400	6,37E-04	193,51	23,986	307,6	73,1
3BOH136	400	6,48E-04	202,37	17,695	280,1	67,9
3BOH136	425	4,58E-04	246,92	-4,5085	254,8	21,3
3BOH136	450	5,29E-04	231,84	9,0222	266,1	39
3BOH136	475	7,59E-04	191,13	-12,628	198,7	62,1
3BOH136	500	9,66E-04	246,35	-35,077	224,8	11
3BOH136	550	6,22E-04	255,37	-20,797	241,2	8,6
3BOH136	575	1,44E-03	157,79	-38,871	154,2	34,1
3BOH136	600	5,25E-04	190,37	10,53	252,4	79,5
3BOH136	625	5,36E-04	270	-16,034	249,4	-3,6
3BOH178	0	1,32E-03	261,73	47,777	327,6	9,3
3BOH178	100	7,09E-04	268,28	27,025	306,8	3,8
3BOH178	150	5,76E-04	267,66	26,724	306,5	4,3
3BOH178	200	4,12E-04	320,74	37,858	333,1	-33,7
3BOH178	250	4,69E-04	323,55	34,713	332	-37,5
3BOH178	300	7,56E-04	324,72	37,581	335,4	-36,2
3BOH178	350	8,85E-04	338,76	44,928	351,4	-36,6
3BOH178	400	6,01E-04	322,68	32,368	329,1	-38,5
3BOH178	425	6,34E-04	321,9	30,217	326,4	-39,3
3BOH178	450	8,75E-04	328,76	42,772	338,5	-43,2
3BOH178	475	6,64E-04	333,34	33,582	338,7	-43,9
3BOH178	500	3,74E-04	349,96	14,723	343,4	-67,9
3BOH178	525	4,22E-04	287,88	46,04	328,1	-8,6
3BOH178	550	5,15E-04	344,78	21,188	340,8	-59,9
3BOH221	0	1,52E-03	222,18	-5,8175	309,1	46,7
3BOH221	100	1,10E-03	210,53	-14,604	289,8	54,5
3BOH221	150	7,88E-04	197,47	-44,437	248	39,1
3BOH221	200	6,33E-04	178,9	-54,699	231,3	31,3
3BOH221	250	7,88E-04	148,51	-36,189	198,5	40,2
3BOH221	300	6,12E-04	140,89	-56,544	210	21,6
3BOH221	350	7,47E-04	111,99	-72,096	215,4	2,8
3BOH221	400	6,70E-04	61,119	-64,56	209	-15,7
3BOH221	425	5,11E-04	161,17	-57,328	220,7	26,8

3BOH221	450	4,84E-04	74,899	-66,16	208,7	-9,7
3BOH221	475	3,32E-04	28,896	-58,046	214,6	-31,4
3BOH221	500	3,98E-04	52,896	-42,516	189,6	-29,4
3BOH221	550	5,88E-04	49,943	-37,276	185	-33,6
3BOH221	575	6,15E-04	330,43	-48,275	257	-39
3BOH221	600	5,31E-04	29,393	-60,311	215,8	-29,4
3BOH221	625	6,66E-04	158,49	-63,742	222	20,4
3BOH221	650	4,72E-04	249,9	-19,543	300,1	17,4
3BOH221	650	8,66E-05	18,544	39,965	71,4	-42,8
3BOH281	0	8,26E-04	198,7	75,569	12,3	13,7
3BOH281	100	7,59E-04	125,11	84,084	21,8	3,4
3BOH281	150	7,94E-04	132,67	83,704	21,6	4,3
3BOH281	200	8,52E-04	49,821	82,055	23,1	-5,1
	250	8,45E-04	38,919	75,82	26	-11
3BOH281	300	8,58E-04	18,36	73,66	22,3	-15,5
3BOH281	350	8,87E-04	17,669	72,937	22,3	-16,2
3BOH281	400	8,12E-04	2,6838	76,309	17,7	-13,7
3BOH281	450	8,34E-04	339,76	72,443	20,7	-18,4
3BOH281	475	1,07E-03	342,52	29,298	348,8	-56,3
3BOH281	500	5,23E-04	326,49	59,84	359,2	-24,8
3BOH281	525	1,39E-03	10,437	67,01	21,4	-22,6
3BOH281	550	9,96E-04	349,28	64,073	11,8	-25,4
3BOH281	575	8,43E-04	325,7	60,223	359,1	-24,2
3BOH281	600	7,86E-04	353,69	72,503	15	-17,4
3BOH281	625	1,01E-03	331,19	83,496	13,9	-5,7
3BOH281	650	5,57E-04	286,61	-11,698	274,8	-16,3
3BOH281	675	1,57E-04	328,14	32,313	337,2	-45,9
3BOH308	0	1,02E-03	141,03	27,832	58,3	42,8
3BOH308	100	6,01E-04	123,35	-22,185	124,5	31
3BOH308	150	5,68E-04	113,42	-23,547	124	21,8
3BOH308	200	5,30E-04	112,42	-18,953	119	21,5
3BOH308	250	4,65E-04	109,53	-14,969	114,5	19,1
3BOH308	300	5,97E-04	111,21	-5,5745	104,6	21,2
3BOH308	350	6,03E-04	110,85	4,1847	94,1	20,7
3BOH308	400	5,35E-04	112,26	-7,7283	106,9	22,2
3BOH308	425	5,07E-04	110,44	-2,0337	100,8	20,5
3BOH308	450	2,03E-04	92,741	-22,25	121,2	2,9
3BOH308	475	2,81E-04	100,91	5,1011	93,6	10,8
3BOH308	500	3,42E-04	72,827	-28,107	128,4	-14,6
3BOH308	525	2,93E-04	112,98	17,497	79,7	21,5
3BOH308	550	3,12E-04	114,54	-13,529	113,4	24,1
3BOH388	0	1,00E-03	181,88	-19,332	264,1	69,6
3BOH388	100	8,87E-04	165,14	-31,53	236,9	54,5
3BOH388	150	9,57E-04	140,1	-31,214	213	40,3
3BOH388	200	9,02E-04	138,41	-36,385	217,5	36,3
3BOH388	250	1,07E-03	143,85	-22,034	204,4	47,9
3BOH388	300	1,14E-03	147,83	-20,554	205,2	51,8
3BOH388	350	1,05E-03	129,16	-25,059	200,7	34,4
3BOH388	400	1,08E-03	139,66	-20,115	199,4	45,2
3BOH388	425	8,61E-04	137,39	-25,24	204,6	41,2
3BOH388	450	9,64E-04	138,13	-23,988	203,5	42,3
3BOH388	475	7,00E-04	144,73	-21,106	203,7	49
3BOH388	500	8,62E-04	136,96	-33,049	213,2	37,1
3BOH388	550	1,14E-03	115,11	-16,603	187,6	23,7
3BOH388	575	7,42E-04	139,99	-23,082	203,4	44,2
3BOH388	600	7,81E-04	125,87	-27,711	202,5	30,7
3BOH388	625	1,12E-03	150,45	-45,597	233,5	36,6
3BOH388	650	1,57E-04	157,66	-27,306	223,5	54,5
3BOH388	675	2,75E-04	295,2	65,814	56,9	-9,1
3BOH428	0	1,06E-03	202,6	47,668	333,7	38,4
3BOH428	100	7,17E-04	221,96	65,856	336,3	17,7
3BOH428	150	5,61E-04	211,82	57,973	334,7	26,8
3BOH428	200	6,38E-04	254,77	46,175	310,2	10,5
3BOH428	250	4,15E-04	239,26	71,853	337,3	9,2
3BOH428	300	6,30E-04	245,66	72,97	337,4	6,9
3BOH428	350	6,69E-04	267,45	76,408	339,4	0,6
3BOH428	400	5,62E-04	292,09	70,939	335,2	-7,1
3BOH428	425	4,65E-04	237,34	53,308	320,9	18,8
3BOH428	450	4,54E-04	275,92	68,705	331,8	-2,1
3BOH428	475	5,38E-04	263,31	32,237	295,4	5,7
3BOH428	500	6,04E-04	319,27	54,496	328	-26,1
3BOH428	525	5,86E-04	279,96	28,63	292	-8,7
3BOH428	550	3,83E-04	281,83	47,49	311,1	-8
3BOH428	575	5,45E-04	292,53	30,431	295,5	-19,3
3BOH428	600	4,86E-04	258,58	30,4	293,9	9,8
3BOH428	625	4,23E-04	272,53	15,372	278,4	-2,4
3BOH428	650	3,72E-04	302,89	45,695	313,7	-22,3
3BOH428	675	1,93E-04	282,24	0,29626	263,3	-12,2
3BOH463	0	1,76E-03	167,74	57,555	21,7	31,6
3BOH463	100	1,07E-03	146,25	19,541	71,4	51,6
3BOH463	150	7,79E-04	135,31	2,5764	100,3	45,3
3BOH463	200	5,90E-04	138,16	-2,0412	107,1	48,1
3BOH463	250	5,83E-04	136,35	-10,882	119,6	45,3
3BOH463	300	5,65E-04	124,97	-23,123	131,5	31,8
3BOH463	350	5,67E-04	126,02	2,9317	100,4	36
3BOH463	400	5,32E-04	116,12	-29,393	136,1	22,6
3BOH463	425	4,92E-04	112,1	-43,67	149,9	15,8
3BOH463	450	5,78E-04	95,375	-37,404	141,5	4,3
3BOH463	475	6,22E-04	129,23	-16,143	124,5	37,4
3BOH463	500	5,42E-04	91,645	-44,988	149	1,2
3BOH463	525	6,24E-04	78,836	-30,546	135	-9,6
3BOH463	550	3,72E-04	60,474	-45,683	153,6	-20,1
3BOH463	575	5,13E-04	128,36	-41,355	152,3	27,8
3BOH486	0	5,10E-04	170,52	-1,4603	113,9	80,5
3BOH486	100	3,25E-04	148,25	16,616	85,1	53,5
3BOH486	150	2,79E-04	145,67	13,275	91,9	52,7
3BOH486	200	1,98E-04	126,6	-13,375	132,1	36
3BOH486	250	1,14E-04	140,42	-30,051	157,9	43,2
3BOH486	300	3,24E-04	175,75	-0,88442	102,3	85,6
3BOH486	350	7,14E-05	36,041	31,509	72,2	-45
3BOH486	400	9,00E-05	111,3	29,284	85,4	17,4
3BOH486	450	8,81E-05	5,5115	-82,534	206,3	-5,4
3BOH486	475	9,33E-05	62,994	5,2259	112,2	-27,1
3BOH486	500	3,38E-04	46,836	-49,231	175,3	-24,8
3BOH486	525	9,05E-05	7,4543	-53,38	201,6	-34,3
3BOH486	550	1,29E-04	107,39	-36,584	154,5	15,1

Table S2. Thermal demagnetization data.

(1) Sample = sample name
(2) Temperature (°C)
(3) NRM intensity (A/m)
(4) Core Declination (°E)
(5) Core Inclination (°)
(6) Geographic Declination (°E)
(7) Geographic Inclination (°)

3BOH486	575	7,31E-04	252,1	-59,217	236,7	10,8
3BOH486	600	3,97E-04	336,5	7,6678	311,4	-65,9
3BOH486	625	1,97E-04	347,88	-8,1568	256,8	-74,2
3BOH486	650	1,88E-04	315,74	54,557	0,2	-26,3
3BOH486	675	1,32E-04	309,68	43,624	347,4	-29,1
3BOH510	0	1,33E-03	205,06	43,102	10,8	31,3
3BOH510	100	5,35E-04	207,43	52,543	14,4	22,2
3BOH510	150	6,35E-04	237,05	59,535	6,7	6,1
3BOH510	200	4,35E-04	238,5	65,286	11,1	2,4
3BOH510	250	6,36E-04	334,42	65,939	20	-32,4
3BOH510	300	3,36E-04	119,98	57,619	59,8	5,8
3BOH510	350	3,94E-04	227,66	62,377	11,8	7,9
3BOH510	400	7,02E-04	295,51	61,132	4	-21,8
3BOH510	425	4,72E-04	241,05	70,053	14,6	-1
3BOH510	450	4,97E-04	296,18	66,046	9,1	-20,5
3BOH510	475	3,19E-04	8,0091	50,981	39,8	-49,5
3BOH510	500	4,57E-04	261,6	54,272	356,6	-4,1
3BOH510	525	4,72E-04	321,8	48,907	359,6	-40,6
3BOH510	550	5,85E-04	341,62	57,872	19,2	-41,1
3BOH510	575	5,20E-04	8,9117	48,886	41,4	-51,4
3BOH510	600	6,90E-04	27,53	46,166	60,4	-47,8
3BOH510	625	7,03E-04	312,35	34,303	338,2	-40,8
3BOH510	650	9,23E-05	148,11	4,7849	97,2	54,5
3BOH510	675	4,55E-04	292,72	5,0444	302,8	-23,2
**OH3-A**						
3OH28	0	6,00E-04	185,27	3,7281	278,9	69,1
3OH28	100	4,65E-04	173,39	30,668	123,9	81,1
3OH28	150	4,24E-04	169,73	37,487	114,7	73,9
3OH28	200	4,72E-04	157,99	39,275	129,4	65,9
3OH28	250	3,91E-04	155,22	54,146	109,1	54,6
3OH28	300	4,44E-04	144,38	40,41	136,5	56
3OH28	350	4,57E-04	150,61	45,895	123,8	57,8
3OH28	400	5,34E-04	171,11	26,337	155,9	81,6
3OH28	425	3,75E-04	146,47	57,233	111,1	49
3OH28	450	3,41E-04	155,71	43,342	123,9	62,2
3OH28	475	2,02E-04	55,125	78,516	93,8	17,1
3OH28	500	4,34E-04	133,88	48,312	128,1	46,5
3OH28	550	3,71E-04	170,73	36,571	114,2	75,1
3OH28	575	3,64E-04	104,4	31,795	148,3	24
3OH28	600	2,69E-04	156,44	23,172	171,4	68,4
3OH28	625	2,75E-04	189,95	65,07	77,7	48,4
3OH51	0	4,39E-04	196,21	36,951	48,6	62
3OH51	100	2,50E-04	165,07	-82,864	255,2	-6,1
3OH51	150	2,58E-04	171,38	-82,572	255,9	-5,7
3OH51	200	1,40E-04	131,69	-20,934	202	31,7
3OH51	250	2,16E-04	123,07	-24,607	201,2	22,9
3OH51	300	3,27E-04	133,33	-27,078	208,9	29,5
3OH51	350	5,72E-05	118,83	-18,234	193,1	22,1
3OH51	400	1,68E-04	102,06	-38,069	206,6	1,2
3OH51	425	7,42E-05	13,317	8,5262	109,3	-64,7
3OH51	450	1,48E-04	47,931	-35,467	203,2	-41,5
3OH51	475	1,72E-04	111,61	-4,6797	176,9	19,8
3OH51	500	2,67E-04	66,65	-41,605	207,2	-26
3OH51	525	1,53E-04	58,25	-12,047	173	-33,3
3OH94	0	8,62E-04	293,09	16,645	3	-15,2
3OH94	100	5,94E-04	308,48	5,6968	359,8	-33,6
3OH94	150	6,02E-04	308,99	2,9537	357,1	-35,3
3OH94	200	6,22E-04	307,55	-2,0262	350,8	-36
3OH94	250	7,41E-04	315,71	2,3203	0,2	-41,5
3OH94	300	6,09E-04	310,92	-5,1849	348,6	-40,3
3OH94	350	5,55E-04	311,93	3,2042	359	-37,8
3OH94	400	5,84E-04	311,18	3,3362	358,7	-37,1
3OH94	450	6,67E-04	311,9	3,1781	358,9	-37,8
3OH94	475	5,08E-04	304,25	1,3528	353	-31,6
3OH94	500	5,93E-04	302,32	-0,67646	349,9	-30,6
3OH94	525	7,26E-04	282,81	10,803	353,5	-8,3
3OH94	550	6,50E-04	300,86	27,078	16,2	-16,5
3OH94	575	8,52E-04	325,56	-13,362	343,4	-56,5
3OH94	600	3,63E-04	292,13	13,231	359,4	-15,8
3OH94	625	6,49E-04	307,26	9,1312	2,7	-30,9
3OH94	650	4,16E-04	4,1748	48,726	72	-22,2
3OH94	675	7,86E-04	338,1	6,6519	26,9	-56,5
3OH121	0	3,51E-03	284,18	73,788	42,6	16,3
3OH121	100	1,37E-03	23,252	51,906	73,6	-14,3
3OH121	150	1,02E-03	42,638	13,288	113,4	-35,9
3OH121	200	1,69E-03	63,435	-7,7961	146,9	-27,5
3OH121	250	9,20E-04	72,367	-21,088	163,8	-23,1
3OH121	300	1,49E-03	53,322	-15,894	152,7	-39,4
3OH121	350	1,65E-03	70,701	-44,706	189,5	-28,1
3OH121	400	1,46E-03	73,442	-21,234	164,2	-22,2
3OH121	425	1,32E-03	52,531	-22,048	160,4	-41,4
3OH121	450	1,47E-03	58,271	-27,913	168,8	-37
3OH121	475	1,50E-03	62,992	-15,859	155,6	-30,4
3OH121	500	1,92E-03	72,629	-18,027	160,7	-22,1
3OH121	550	2,55E-03	75,739	-13,401	156,9	-17,9
3OH121	575	1,48E-03	91,725	-26,104	174,1	-7,6
3OH121	600	2,20E-03	75,964	-7,5594	151,2	-15,8
3OH121	625	9,75E-04	98,763	36,736	46,4	-39,8
3OH121	625	1,92E-04	349,09	28,029	116	19,2
3OH144	0	3,28E-04	227,59	17,393	340,5	43,3
3OH144	100	1,94E-04	215,12	12,209	333	55,5
3OH144	150	1,68E-04	199,72	33,961	22	60
3OH144	200	2,40E-04	159,44	16,195	126,6	69,1
3OH144	250	2,68E-04	158,77	14,056	132,8	68,9
3OH144	300	2,43E-04	154,05	30,981	101,2	58,1
3OH144	350	2,21E-04	163,02	25,792	99,3	67,5
3OH144	400	1,74E-04	168,5	39,812	73	58,5
3OH144	450	1,10E-04	355,41	72,891	54,6	-7,1
3OH144	475	7,58E-05	275,39	45,409	11,6	3,4
3OH144	500	2,12E-04	265,45	57,655	23,1	10,9
3OH144	525	2,54E-04	279,46	56,578	23	3,2
3OH144	550	1,06E-04	199,73	25,303	7,8	65,8
3OH144	575	5,95E-04	203,28	61,809	42,7	35,5
3OH144	600	3,03E-04	264,78	43,543	8,7	10,6
3OH144	625	3,37E-04	204,61	-59,635	248,8	17,6
3OH144	650	2,43E-04	70,866	31,272	111,3	-10,7
3OH144	675	1,33E-04	343,79	-7,3902	334	-73,8
3OH172	0	5,63E-04	303,86	-35,003	313,1	-23,9
3OH172	100	3,96E-04	316,26	-13,869	330,9	-42,7
3OH172	150	4,09E-04	304,07	-22,887	325,5	-28,7
3OH172	200	4,16E-04	308,57	-9,4039	339,3	-36,8
3OH172	250	3,78E-04	307,36	-29,304	317,4	-29
3OH172	300	4,06E-04	302,41	-23,21	325,7	-27,2
3OH172	350	3,32E-04	325,68	-24,751	311,7	-45,3
3OH172	400	3,64E-04	322,97	-38,231	300,2	-34,8
3OH172	425	3,93E-04	321,91	-24,331	314,9	-42,7
3OH172	450	3,97E-04	325,07	-14,309	325,4	-50,3
3OH172	475	5,53E-04	342,1	-23,339	295,9	-56,7
3OH172	500	3,88E-04	339,95	-22,282	300	-56,4
3OH172	525	4,75E-04	331,75	-16,132	317,4	-55
3OH172	550	5,32E-04	322,7	-7,0179	337,4	-50,9
3OH172	575	5,65E-04	315,98	-59,189	286,8	-17
3OH212	0	4,27E-04	210,84	-67,713	246,9	19
3OH212	100	4,68E-04	126,06	-74,191	222,1	9,2
3OH212	150	4,57E-04	104,04	-80,125	225,4	2,4
3OH212	200	7,51E-04	339,57	-81,666	237,9	-7,8
3OH212	250	7,91E-04	2,8624	-85,646	234,8	-4,3
3OH212	300	6,30E-04	341,57	-77,833	238,9	-11,5
3OH212	350	6,37E-04	284,83	-80,463	244,2	-2,4
3OH212	400	7,19E-04	24,928	-83,751	232,4	-5,7
3OH212	450	6,83E-04	272,77	-84,784	240,2	-0,3
3OH212	475	6,71E-04	309,66	-81,544	241,5	-5,4
3OH212	500	6,92E-04	228,18	-83,656	239,7	4,2
3OH212	525	7,50E-04	331,06	-50,663	256,6	-33,7
3OH212	550	7,45E-04	288,86	-69,829	254,2	-6,4
3OH212	575	6,02E-04	348,77	-76,688	237,6	-13,1
3OH212	600	9,09E-04	122,32	-86,342	231,9	2
3OH212	625	4,31E-04	33,83	-74,783	226,4	-12,6
3OH212	650	5,62E-04	152,96	-73,165	227,2	14,9
3OH212	675	3,29E-04	232,4	-0,86956	323,9	37,6
3OH229	0	1,16E-03	286,08	30,111	319,1	-11,2
3OH229	100	8,38E-04	309,5	9,475	303	-37,6
3OH229	150	8,00E-04	315,71	2,15	295,1	-45,2
3OH229	200	7,82E-04	320,93	8,9715	306,6	-48,6
3OH229	250	7,60E-04	318,09	9,8529	306,6	-45,7
3OH229	300	7,60E-04	314,68	5,4359	299,4	-43,6
3OH229	350	8,05E-04	323,2	-7,778	280,7	-53,3
3OH229	400	4,79E-04	305,76	-18,87	267,4	-35,4
3OH229	425	8,23E-04	331,61	8,7314	312,8	-58,5
3OH229	450	7,52E-04	319,75	1,0664	294,5	-49,3
3OH229	475	7,48E-04	315,87	2,5302	295,7	-45,3
3OH229	500	8,97E-04	316,54	-2,749	288,3	-46,6
3OH229	550	7,04E-04	322,05	-4,1521	286,7	-52,2
3OH229	575	9,13E-04	316,4	-18,579	265,5	-45,4
3OH229	600	8,59E-04	335,73	1,0672	300,4	-65
3OH229	625	8,79E-04	330,68	-15,913	264	-59,2
3OH229	650	2,03E-04	340,58	-7,6331	278,2	-70,5
3OH246	0	1,27E-03	139,38	-53,901	230,8	32,8
3OH246	100	1,13E-03	127,34	-52,246	224,6	27,7
3OH246	150	9,53E-04	118,01	-56,025	226,1	21,2
3OH246	200	7,64E-04	105,99	-59,751	228,1	14,1
3OH246	250	7,26E-04	84,5	-68,048	236,1	4,4
3OH246	300	7,55E-04	84,199	-65,193	233,3	3,9
3OH246	350	7,16E-04	52,741	-57,65	232,1	-12,6
3OH246	400	5,23E-04	6,3144	-62,016	254,8	-20,8
3OH246	450	5,65E-04	1,8328	-63,741	257,1	-19,2
3OH246	475	5,68E-04	1,206	-70,444	257,6	-12,6
3OH246	500	6,39E-04	312,77	-44,619	292,6	-23,2
3OH246	525	4,87E-04	295,44	-56,009	288,6	-7,9
3OH246	550	6,79E-04	340,58	-53,624	270,8	-27,2
3OH246	575	5,77E-04	328,26	-58,44	275	-19,8
3OH246	600	1,07E-03	321,13	-43,385	289,3	-28,5
3OH246	625	6,60E-04	315,38	-49,159	287,6	-21,7
3OH246	650	2,63E-04	294,97	72,145	61,3	-14,2
3OH246	675	7,74E-04	324,71	33,417	31,9	-48
3OH313	0	9,43E-04	201,07	-47,287	318,2	57,8
3OH313	100	4,63E-04	140,83	37,199	143,8	21,9
3OH313	150	4,13E-04	124,36	41,36	150	9,9
3OH313	200	3,50E-04	133,51	45,526	142,3	12,1
3OH313	250	2,63E-04	119,3	52,67	142,9	0,4
3OH313	300	9,28E-05	151,78	31,745	139,2	31,6
3OH313	350	2,41E-04	87	17,885	185,1	-8,7
3OH313	400	2,15E-04	114,62	60,546	137,7	-6
3OH313	425	2,48E-04	191,63	32,95	98,9	35,9
3OH313	450	3,16E-04	276,17	83,241	103,8	-20,6
3OH313	475	4,06E-04	1,7062	54,614	112,7	-55,4
3OH313	500	2,80E-04	159,79	51,582	123,9	16,3
3OH313	525	2,26E-04	334,73	15,399	14,3	-65,5
3OH339	0	1,25E-03	249,24	-37,245	11,3	26,5
3OH339	100	6,97E-04	264,51	-32,838	14,4	13,6
3OH339	150	7,34E-04	267,79	-35,599	11	11,5
3OH339	200	6,36E-04	281,16	-46,318	357,8	4,8
3OH339	250	6,37E-04	278,66	-41,345	3,1	4,9
3OH339	300	5,38E-04	275,46	-32,86	12	4,7
3OH339	350	6,02E-04	287,57	-12,966	27,1	-12,5
3OH339	400	6,02E-04	272,84	-25,047	20,2	4,6
3OH339	450	6,82E-04	278,7	-26,941	16,8	0,2
3OH339	475	5,17E-04	285,25	0,22156	40,7	-14,6
3OH339	500	6,31E-04	297,23	-24,425	12,4	-16,1
3OH339	525	3,33E-04	290,78	0,17225	38,8	-19,9
3OH339	550	6,30E-04	288,52	-14,066	25,7	-12,9
3OH339	575	8,43E-04	304,2	15,828	52,4	-36,6
3OH339	600	5,72E-04	277,37	-6,2218	36,9	-5,2
3OH339	625	9,20E-04	285,62	28,91	70	-21,5
3OH339	650	2,66E-04	288,06	79,537	124,4	-20
3OH339	675	2,73E-04	346,83	-3,3645	348,8	-65,8
3OH379	0	7,48E-04	209,51	-53,503	324,7	40,5
3OH379	100	2,78E-04	177,46	-35,894	297,3	64
3OH379	150	1,30E-04	140,58	-13,886	221,8	51,3
3OH379	200	2,30E-04	117,09	30,041	175,9	17,5
3OH379	250	2,51E-04	83,29	61,535	150,9	-12

Table S2. Thermal demagnetization data.

(1) Sample = sample name
(2) Temperature (°C)
(3) NRM intensity (A/m)
(4) Core Declination (°E)
(5) Core Inclination (°)
(6) Geographic Declination (°E)
(7) Geographic Inclination (°)

3OH379	300	2,30E-04	229,79	19,514	63,1	32,8
3OH379	350	8,91E-05	191,45	56,735	115,2	22,6
3OH379	400	4,19E-04	115,88	69,197	140,6	-0,6
3OH379	425	4,19E-04	279,4	70,37	102,1	-12,6
3OH379	450	3,85E-04	77,471	87,258	124,7	-10,6
3OH379	475	4,29E-04	12,967	68,697	127,4	-30,7
3OH379	500	3,26E-04	68,199	69,717	141,7	-16,8
3OH379	550	4,38E-04	48,211	60,675	146,5	-28,2
3OH379	575	3,90E-04	19,163	67,479	130,4	-31,1
3OH379	600	3,12E-04	102,94	75,932	135,8	-6,6
3OH379	625	2,69E-04	75,809	0,21282	214,3	-14
3OH379	650	4,41E-04	107,42	-31,912	242,5	20
3OH379	675	1,00E-04	286,04	-3,4946	25,6	-15,1
3OH405	0	7,36E-03	204,74	28,82	34,1	44,4
3OH405	100	5,70E-03	204,92	32,248	36,6	41,5
3OH405	150	5,62E-03	202,62	31,174	38	43,5
3OH405	200	4,81E-03	201,13	31,336	39,7	44
3OH405	250	3,74E-03	204,13	32,365	37,4	41,8
3OH405	300	3,16E-03	209,77	33,406	33,2	38,2
3OH405	350	3,20E-03	207,8	32,33	34	40
3OH405	400	2,64E-03	204,14	28,474	34,4	45
3OH405	450	1,67E-03	203,31	26,195	33,4	47,3
3OH405	475	8,88E-04	200,54	24,33	35	50,2
3OH405	475	1,11E-03	197,05	27,487	41,6	49
3OH405	500	1,18E-03	200,96	33,18	41,1	42,4
3OH405	525	1,22E-03	182,17	12,152	59,4	67,7
3OH405	550	7,10E-04	209,53	23,771	24,9	45,6
3OH405	575	7,56E-04	193,96	12,532	33,1	63,5
3OH405	600	8,52E-04	206,63	18,548	22,3	51,2
3OH405	625	4,53E-04	248,94	-27,767	309	23,2
3OH405	650	8,45E-04	53,093	68,387	83,6	-22,3
3OH405	675	1,24E-04	332,11	48,954	39,4	-44,6
3OH462	0	4,29E-03	276,24	9,2537	9,8	-5,3
3OH462	100	3,17E-03	278,62	8,7038	9,5	-7,7
3OH462	150	3,04E-03	279,28	-2,0725	358,7	-9,4
3OH462	200	2,54E-03	287,34	-12,49	348,5	-18
3OH462	250	2,15E-03	283,16	-16,463	344,3	-14
3OH462	300	2,00E-03	278,98	-13,126	347,5	-9,9
3OH462	350	1,95E-03	290,73	5,0274	7,2	-20,1
3OH462	400	1,92E-03	284,27	-1,0133	0,2	-14,3
3OH462	425	1,98E-03	282,99	1,6782	2,9	-12,8
3OH462	450	2,11E-03	289,98	0,27195	2,1	-19,9
3OH462	475	2,36E-03	294,83	-1,4577	0,7	-24,9
3OH462	500	2,31E-03	287,89	-0,24785	1,4	-17,8
3OH462	525	1,94E-03	290,92	-3,7897	357,9	-21,1
3OH462	550	1,81E-03	286,85	8,59	10,4	-15,8
3OH462	575	1,90E-03	281,95	0,15052	1,2	-11,9
3OH462	600	1,72E-03	303,18	-7,6999	354,1	-33,5
3OH462	625	1,07E-03	326,63	-21,349	330,1	-53,8
3OH462	650	6,61E-04	261,91	-11,966	347,3	6,8
3OH490	0	6,23E-03	235,58	-12,328	321,6	28,6
3OH490	100	4,42E-03	227,77	-16,548	313,1	33,3
3OH490	150	3,46E-03	228,83	-18,69	311,6	31,3
3OH490	200	2,67E-03	236,75	-18,129	316,3	25
3OH490	250	2,66E-03	233,99	-17,725	315,3	27,5
3OH490	300	1,14E-03	241,69	-19,675	317	20,1
3OH490	350	1,17E-03	234,17	-6,0663	327,6	32,3
3OH490	400	9,02E-04	213,91	-14,912	304,8	45,1
3OH490	425	5,06E-04	241,49	-30,624	306,6	15,4
3OH490	450	6,75E-04	230,43	-17,949	313,2	30,4
3OH490	475	6,68E-04	231,74	-10,517	321,6	32,7
3OH490	500	1,35E-04	282,15	46,512	32,7	2,7
3OH490	550	3,84E-04	8,9816	29,831	85,9	-44,3
3OH490	575	3,61E-04	41,269	-25,432	186,9	-50,1
3OH490	600	2,60E-04	30,407	18,158	118,1	-45,3
3OH490	625	4,70E-04	23,601	5,8676	125	-58,7
3OH490	650	5,09E-04	215,78	-9,0389	313,1	47,2
**OH4**						
4OH8	0	4,04E-02	4,808	-37,029	230,2	-52,7
4OH8	100	9,08E-02	37,794	-34,947	195,3	-40,4
4OH8	150	1,08E-01	43,666	-35,581	192,5	-36
4OH8	200	1,08E-01	38,984	-37,44	197,1	-38,1
4OH8	250	1,08E-01	46,561	-35,341	190,8	-34,1
4OH8	300	1,17E-01	45,042	-34,364	190,5	-35,7
4OH8	350	1,21E-01	49,363	-31,689	185,6	-33,7
4OH8	400	1,11E-01	40,819	-32,31	190,6	-39,8
4OH8	450	1,26E-01	45,111	-29,576	185,2	-37,9
4OH8	475	1,19E-01	47,866	-28,536	182,8	-36,1
4OH8	500	1,21E-01	46,23	-29,118	184,1	-37,2
4OH8	525	1,24E-01	47,425	-29,234	183,7	-36,2
4OH8	550	1,26E-01	48,804	-30,369	184,4	-34,6
4OH8	575	1,27E-01	46,695	-31,91	187,1	-35,6
4OH8	600	1,19E-01	47,303	-29,485	184,1	-36,2
4OH8	625	1,17E-01	46,229	-29,235	184,3	-37,1
4OH8	650	1,16E-01	48,16	-29,184	183,4	-35,6
4OH8	675	1,99E-02	253,01	7,2993	334,1	16,8
4OH29	0	1,57E-02	2,2546	-13,245	224,4	-79,5
4OH29	100	2,79E-02	33,376	-25,033	184	-51,1
4OH29	150	3,04E-02	31,982	-31,072	193	-48,8
4OH29	200	3,55E-02	35,961	-27,5	185,6	-47,8
4OH29	250	3,69E-02	41,072	-26,545	181,5	-44,2
4OH29	300	3,63E-02	34,774	-27,228	186	-48,9
4OH29	350	3,63E-02	37,051	-29,039	186,9	-46,2
4OH29	400	3,76E-02	36,663	-28,226	186,1	-46,9
4OH29	425	3,91E-02	38,558	-25,586	181,5	-46,6
4OH29	450	3,63E-02	33,837	-26,349	185,5	-50
4OH29	475	3,73E-02	33,548	-26,1	185,4	-50,4
4OH29	500	3,68E-02	35,077	-25,426	183,4	-49,5
4OH29	525	4,05E-02	35,754	-8,8004	157,4	-54
4OH29	550	3,67E-02	32,735	-24,969	184,4	-51,6
4OH29	575	2,89E-02	33,509	-24,555	183,3	-51,2
4OH29	600	2,83E-02	31,724	-23,786	183,5	-53
4OH29	625	3,40E-03	273,86	-58,401	268,1	-4,6
4OH59	0	2,57E-02	87,011	-14,396	203,4	-8,7
4OH59	100	4,83E-02	67,88	-14,743	196,4	-25,9
4OH59	150	5,06E-02	72,375	-13,264	196,6	-21,3
4OH59	200	5,39E-02	68,097	-14,275	196	-25,6
4OH59	250	5,33E-02	72,128	-14,797	198,1	-22,1
4OH59	300	5,83E-02	68,781	-13,693	195,7	-24,8
4OH59	350	6,03E-02	66,354	-12,459	193,4	-26,5
4OH59	400	6,24E-02	66,901	-13,443	194,7	-26,4
4OH59	450	6,38E-02	62,246	-11,576	190,8	-29,9
4OH59	475	6,16E-02	65,941	-11,703	192,5	-26,6
4OH59	500	6,30E-02	65,161	-8,1211	188,5	-25,9
4OH59	525	6,29E-02	63,559	-9,7044	189,4	-28
4OH59	550	6,27E-02	64,222	-9,5441	189,5	-27,3
4OH59	575	5,94E-02	66,918	-11,564	192,7	-25,6
4OH59	600	6,12E-02	64,455	-9,7866	189,9	-27,2
4OH59	625	6,01E-02	67,71	-11,91	193,4	-25,1
4OH59	650	1,02E-02	38,283	1,6258	161,5	-44,4
4OH59	675	8,70E-03	324,04	14,655	56,1	-37,1
oh4_7	0	1,26E-02	262,3	-34,1	262,3	-34,1
oh4_7	100	2,39E-02	209	-38,3	209	-38,3
oh4_7	150	2,58E-02	190,4	-28,5	190,4	-28,5
oh4_7	200	3,26E-02	192,9	-33,6	192,9	-33,6
oh4_7	225	3,47E-02	193,4	-31,5	193,4	-31,5
oh4_7	250	3,73E-02	192,2	-32,1	192,2	-32,1
oh4_7	275	3,97E-02	188,9	-31,7	188,9	-31,7
oh4_7	300	4,30E-02	185,7	-35,6	185,7	-35,6
oh4_7	325	4,13E-02	189,5	-28,2	189,5	-28,2
oh4_7	350	4,36E-02	189,5	-31	189,5	-31
oh4_7	375	4,25E-02	191,1	-29,5	191,1	-29,5
oh4_7	400	4,31E-02	187,1	-28,4	187,1	-28,4
oh4_7	425	4,35E-02	187,5	-31,2	187,5	-31,2
oh4_7	450	4,57E-02	181	-38,6	181	-38,6
oh4_7	475	4,48E-02	187	-27,4	187	-27,4
oh4_7	500	4,80E-02	170,5	-32,1	170,5	-32,1
oh4_7	525	4,39E-02	178,4	-29,6	178,4	-29,6
oh4_7	550	4,48E-02	181	-29	181	-29
oh4_7	575	4,52E-02	174,2	-26,9	174,2	-26,9
oh4_7	600	4,28E-02	180,5	-22,2	180,5	-22,2
oh4_7	625	4,44E-02	175,1	-28,6	175,1	-28,6
oh4_7	650	9,40E-03	171,6	0,8	171,6	0,8
oh4_7	675	9,51E-03	171,6	-25,4	171,6	-25,4
4OH96	0	1,23E-02	60,119	-70,237	210,7	-16,4
4OH96	100	2,62E-02	51,921	-61,922	204,7	-23,3
4OH96	150	3,27E-02	65,746	-52,377	192,1	-20,2
4OH96	200	3,54E-02	49,582	-54,837	198,7	-28,1
4OH96	250	4,01E-02	56,919	-58,588	200,2	-22,7
4OH96	300	4,17E-02	54,75	-52,338	194,6	-26,5
4OH96	350	3,92E-02	60,286	-54,448	195,3	-22,7
4OH96	400	4,21E-02	56,993	-48,465	190	-26,7
4OH96	425	4,09E-02	60,195	-54,514	195,4	-22,7
4OH96	450	4,04E-02	57,819	-53,135	194,6	-24,5
4OH96	475	4,11E-02	52,705	-53,843	196,7	-27
4OH96	500	3,99E-02	62,374	-52,782	193,2	-22,1
4OH96	525	4,00E-02	65,273	-51,182	191	-20,8
4OH96	550	3,98E-02	66,484	-49,109	188,6	-20,6
4OH96	575	3,85E-02	64,699	-50,765	190,6	-21,3
4OH96	600	4,03E-02	63,736	-50,701	190,8	-21,9
4OH96	625	2,23E-02	63,78	-42,929	182,6	-23,8
4OH96	650	5,56E-03	312,5	-6,4078	316,2	-42,8
4OH116	0	3,80E-03	284,46	-58,879	252	-16,9
4OH116	100	7,19E-03	349,81	-65,378	225,7	-35,2
4OH116	150	8,08E-03	4,1803	-68,053	218,6	-32,9
4OH116	200	8,18E-03	346,4	-67,044	226,8	-33,2
4OH116	250	8,44E-03	356,21	-61,011	222,9	-39,9
4OH116	300	8,58E-03	1,2575	-61,458	219,7	-39,5
4OH116	350	8,90E-03	15,893	-59,679	210,1	-39,9
4OH116	400	8,87E-03	4,4917	-57,826	217,2	-43
4OH116	450	8,78E-03	14,094	-56,828	210	-42,9
4OH116	475	8,12E-03	359,5	-55,585	220,9	-45,4
4OH116	500	8,09E-03	2,2329	-57,351	218,8	-43,6
4OH116	525	9,39E-03	358,99	-56,865	221,3	-44,1
4OH116	550	8,62E-03	1,4354	-59,382	219,5	-41,6
4OH116	575	8,41E-03	0,8814	-57,233	219,8	-43,8
4OH116	600	7,37E-03	5,3662	-45,866	214	-54,8
4OH116	625	7,83E-03	349,38	-55,852	228,8	-44,4
4OH116	650	3,27E-03	112,17	-26,758	162	14,2
4OH116	675	1,76E-03	137,51	-53,413	195,7	16,2
4OH145	0	1,67E-03	187,26	-29,093	249,2	65,9
4OH145	100	5,10E-03	33,69	-62,645	218,1	-16,7
4OH145	150	5,79E-03	36,561	-50,181	208,5	-25,5
4OH145	200	6,53E-03	49,399	-47,325	200	-21,2
4OH145	250	6,38E-03	39,472	-46,306	204	-27
4OH145	300	8,47E-03	53,711	-43,774	195	-20,7
4OH145	350	7,92E-03	52,231	-48,714	200,1	-18,9
4OH145	400	9,10E-03	35,168	-47,677	207,4	-28
4OH145	425	8,70E-03	51,39	-52,161	203,4	-17,3
4OH145	450	8,54E-03	53,13	-46,281	197,5	-19,7
4OH145	475	8,39E-03	50,581	-45,387	197,8	-21,7
4OH145	500	8,41E-03	56,851	-48,48	198,2	-16,4
4OH145	525	9,20E-03	61,674	-48,342	196,5	-13,6
4OH145	550	8,59E-03	63,703	-48,1	195,7	-12,5
4OH145	575	8,70E-03	65,607	-50,389	197,3	-10,4
4OH145	600	8,41E-03	59,161	-48,997	197,9	-14,8
4OH145	625	8,28E-03	69,645	-52,84	198,7	-7,2
4OH145	650	2,74E-03	262,18	-15,028	309,2	9,1
4OH145	675	1,88E-03	303,04	-7,4158	311	-31,6
oh4_8	0	8,64E-03	181,1	-65,2	181,1	-65,2
oh4_8	100	1,30E-02	199,4	-47,7	199,4	-47,7
oh4_8	150	1,81E-02	184,5	-39,8	184,5	-39,8
oh4_8	200	2,08E-02	185,7	-40,1	185,7	-40,1
oh4_8	225	2,06E-02	181,2	-29,1	181,2	-29,1
oh4_8	250	2,35E-02	183,7	-33,2	183,7	-33,2
oh4_8	275	2,22E-02	183,4	-28,1	183,4	-28,1
oh4_8	300	2,56E-02	187,3	-26	187,3	-26
oh4_8	325	2,51E-02	185	-32,2	185	-32,2
oh4_8	350	2,61E-02	182,8	-33	182,8	-33
oh4_8	375	2,62E-02	184,8	-31,6	184,8	-31,6
oh4_8	400	2,74E-02	182,3	-33,9	182,3	-33,9

Table S2. Thermal demagnetization data.

(1) Sample = sample name
(2) Temperature (°C)
(3) NRM intensity (A/m)
(4) Core Declination (°E)
(5) Core Inclination (°)
(6) Geographic Declination (°E)
(7) Geographic Inclination (°)

oh4_8	425	2,45E-02	185,8	-31,9	185,8	-31,9
oh4_8	450	2,30E-02	175	-23	175	-23
oh4_8	475	2,71E-02	184,6	-32,1	184,6	-32,1
oh4_8	500	2,73E-02	178,2	-28,2	178,2	-28,2
oh4_8	525	2,68E-02	175,1	-30,1	175,1	-30,1
oh4_8	550	2,70E-02	176,9	-29,1	176,9	-29,1
oh4_8	575	2,73E-02	176,3	-30	176,3	-30
oh4_8	600	6,47E-03	178,5	1,9	178,5	1,9
oh4_8	625	2,20E-03	200	36,1	200	36,1
oh4_8	650	1,81E-03	115,1	12,2	115,1	12,2
oh4_8	675	2,58E-03	330	63,7	330	63,7
4OH166	0	3,31E-03	243,75	-5,0214	284,3	24,4
4OH166	100	5,47E-03	321,63	-83,579	209,7	-17
4OH166	150	6,49E-03	3,6745	-80,315	204,8	-21,7
4OH166	200	6,98E-03	346,94	-84,172	206,9	-17,7
4OH166	250	8,10E-03	52,001	-84,246	200,8	-15,5
4OH166	300	7,95E-03	84,76	-73,186	188,3	-13
4OH166	350	8,74E-03	48,331	-72,162	191,1	-23,4
4OH166	400	9,08E-03	49,451	-74,884	193,2	-21,5
4OH166	450	9,66E-03	61,715	-74,799	191,4	-18,8
4OH166	475	9,28E-03	52,237	-73,121	191,2	-21,9
4OH166	500	9,55E-03	53,836	-71,162	189,1	-22,5
4OH166	525	1,02E-02	64,627	-72,907	189,2	-18,8
4OH166	550	1,10E-02	64,959	-74,934	191,2	-18
4OH166	575	9,95E-03	66,335	-74,005	190,1	-17,9
4OH166	600	1,04E-02	68,59	-66,858	182,7	-19,4
4OH166	625	1,00E-02	45,264	-72,203	191,7	-24,1
4OH166	650	4,49E-03	325,56	-79,106	212,1	-20,9
4OH166	675	5,84E-03	341,33	-67,033	214,1	-33,6
4OH198	0	4,66E-03	220,27	-39,394	254	28,1
4OH198	100	5,54E-03	36,158	-86,667	217,5	-12,7
4OH198	150	5,86E-03	17,684	-82,907	217,3	-16,7
4OH198	200	7,21E-03	51,384	-73,613	205,9	-19,9
4OH198	250	7,54E-03	61,15	-75,362	206,1	-16,7
4OH198	300	7,76E-03	76,515	-74,938	204,5	-13,1
4OH198	350	7,81E-03	75,051	-74,15	203,7	-13,7
4OH198	400	7,70E-03	69,91	-72,624	202,6	-15,5
4OH198	425	7,12E-03	75,572	-72,63	202,2	-13,8
4OH198	450	7,37E-03	53,302	-63,157	196	-24,9
4OH198	475	7,44E-03	58,072	-71,628	203	-19,2
4OH198	500	7,29E-03	51,216	-60,466	193,9	-27,1
4OH198	525	7,99E-03	50,75	-40,535	172,9	-35,9
4OH198	550	6,53E-03	62,739	-66,763	197,6	-19,7
4OH198	575	7,39E-03	57,717	-61,728	193,6	-23,7
4OH198	600	9,84E-04	301,47	-58,944	248,4	-24,5
4OH198	625	7,17E-04	204,25	26,325	348,2	61,9
oh4_9	0	5,98E-03	300,4	81	300,4	81
oh4_9	100	4,17E-03	195,7	47,1	195,7	47,1
oh4_9	150	4,58E-03	192,5	17,6	192,5	17,6
oh4_9	200	5,67E-03	178,5	4,7	178,5	4,7
oh4_9	225	5,23E-03	190,2	3	190,2	3
oh4_9	250	6,18E-03	184,8	-3	184,8	-3
oh4_9	275	5,95E-03	186,5	1	186,5	1
oh4_9	300	6,51E-03	181,8	-4,4	181,8	-4,4
oh4_9	325	8,11E-03	183,7	-15,9	183,7	-15,9
oh4_9	350	6,93E-03	186,7	-6,5	186,7	-6,5
oh4_9	375	7,32E-03	182,6	0,1	182,6	0,1
oh4_9	400	6,70E-03	181,1	-1,6	181,1	-1,6
oh4_9	425	7,59E-03	178,2	-2,3	178,2	-2,3
oh4_9	450	7,88E-03	188,3	-32,3	188,3	-32,3
oh4_9	475	8,06E-03	179,8	-19,6	179,8	-19,6
oh4_9	500	7,22E-03	172,8	-10	172,8	-10
oh4_9	525	7,51E-03	173	-14,2	173	-14,2
oh4_9	550	7,85E-03	180,9	-16,1	180,9	-16,1
oh4_9	575	6,95E-03	172,8	-38,2	172,8	-38,2
oh4_9	600	7,81E-03	176,2	-5,6	176,2	-5,6
oh4_9	625	8,31E-03	166,9	-9,8	166,9	-9,8
oh4_9	650	1,69E-03	146,1	-8,8	146,1	-8,8
oh4_9	675	2,64E-03	165,1	27,6	165,1	27,6
4OH237	0	4,82E-03	307,18	-39,105	314,4	-34,8
4OH237	100	5,89E-03	12,867	-46,709	251,2	-51,7
4OH237	150	6,32E-03	22,102	-43,281	239,4	-51,6
4OH237	200	6,38E-03	26,508	-44,836	237,1	-48,4
4OH237	250	6,74E-03	31,491	-44,017	232,5	-46,4
4OH237	300	7,59E-03	33,549	-42,185	229	-46,4
4OH237	350	6,96E-03	48,493	-29,823	206,5	-40,7
4OH237	400	6,98E-03	34,937	-43,091	229,2	-45,1
4OH237	450	7,44E-03	32,826	-39,982	226,9	-48,2
4OH237	475	7,66E-03	29,674	-38,219	227,1	-51,2
4OH237	500	7,51E-03	30,628	-42,274	231,2	-48,1
4OH237	525	7,58E-03	39,161	-34,382	216	-46,7
4OH237	550	7,21E-03	35,88	-33,325	216,4	-49,7
4OH237	575	7,39E-03	45,072	-40,304	220,7	-40
4OH237	600	8,82E-03	33,822	-35,069	220	-50,3
4OH237	625	9,14E-03	34,104	-32,999	217	-51,1
4OH237	650	7,86E-03	39,885	-34,143	215,3	-46,3
4OH237	675	6,45E-03	59,487	-32,309	207,3	-31
4OH263	0	5,32E-03	215,25	25,505	38,5	45,5
4OH263	100	2,38E-03	156,21	24,64	125,1	54
4OH263	150	1,74E-03	167,9	23,354	110	61,1
4OH263	200	3,12E-03	129,46	15,44	155,7	36,7
4OH263	250	2,58E-03	111,68	5,3312	169,6	21,2
4OH263	300	3,04E-03	100,23	27,382	148,4	7,7
4OH263	350	3,68E-03	104,32	21,011	154,2	12,2
4OH263	400	2,08E-03	97,09	13,064	163	6,2
4OH263	425	3,32E-03	101,24	13,568	162,1	10,2
4OH263	450	4,19E-03	97,503	9,4726	166,6	6,9
4OH263	475	4,44E-03	88,956	8,4209	168,1	-1,5
4OH263	500	4,88E-03	88,809	9,9039	166,7	-1,7
4OH263	525	4,63E-03	90,252	11,455	165	-0,3
4OH263	550	4,51E-03	95,255	19,167	157	4
4OH263	575	4,05E-03	91,152	10,67	165,8	0,6
4OH263	600	4,11E-03	100,7	10,516	165,3	9,9
4OH263	625	5,00E-03	98,352	18,289	157,7	7
4OH263	650	2,72E-03	256,13	25,04	22,8	11,2
4OH263	675	1,50E-03	106,91	43,79	131	10
4OH303	0	3,23E-03	236,2	27,229	325,5	35,4
4OH303	100	2,00E-03	251,23	-12,724	283,6	15,1
4OH303	150	2,23E-03	265,72	-38,59	261,9	-4,2
4OH303	200	1,84E-03	264,22	-29,751	270,3	-1
4OH303	250	2,07E-03	297,15	-48,992	251,3	-26,7
4OH303	300	2,04E-03	287,24	-47,416	254,1	-20,4
4OH303	350	1,55E-03	297,4	-51,842	248,2	-26,2
4OH303	400	2,16E-03	305,01	-57,641	240,4	-28,4
4OH303	450	2,09E-03	344,07	-54,487	223,7	-45,7
4OH303	475	1,87E-03	321,71	-55,792	236,4	-37,1
4OH303	500	2,23E-03	330,26	-52,091	235,3	-43,3
4OH303	525	2,15E-03	327,49	-48,846	240,2	-44,4
4OH303	550	2,70E-03	351,52	-64,715	215	-37
4OH303	575	2,49E-03	356,85	-59,274	212,7	-42,7
4OH303	600	2,60E-03	16,526	-43,156	188,9	-55,7
4OH303	625	8,20E-04	346,03	-57,61	220,7	-43,2
4OH303	650	7,07E-04	193,11	4,3777	271,1	75
4OH303	675	1,33E-03	188,04	-17,87	225,5	59,1
4OH363	0	3,10E-02	213,6	53,98	361,2	40,3
4OH363	100	1,77E-02	222,87	66,562	8,6	28,4
4OH363	150	1,30E-02	178,31	45,237	28,7	56,7
4OH363	200	9,97E-03	327,13	77,725	19,9	1,6
4OH363	250	1,11E-02	280,46	83,141	19,6	10,7
4OH363	300	1,20E-02	21,352	65,563	35,3	-10,8
4OH363	350	9,25E-03	4,0567	72,209	27,7	-5,7
4OH363	400	6,90E-03	318,69	82,68	21,6	6,5
4OH363	425	7,78E-03	0,35959	52,099	26,7	-25,9
4OH363	450	6,28E-03	2,5378	54,892	28,1	-23,1
4OH363	475	7,40E-03	0,8443	50,092	27,1	-27,9
4OH363	500	6,43E-03	10,587	70,209	30,1	-7,5
4OH363	525	6,24E-03	26,277	44,481	47,6	-28,7
4OH363	550	7,06E-03	28,183	49,396	46,1	-23,8
4OH363	575	7,44E-03	25,305	44,688	46,8	-28,8
4OH363	600	8,72E-03	18,342	47,609	40,4	-28,2
4OH363	625	6,08E-03	23,732	3,6807	84,3	-61,7
4OH363	650	4,98E-03	329,12	47,111	3,9	-24,8
4OH363	675	4,54E-03	198,35	-13,379	242,7	58,8
4OH403	0	9,80E-03	171,93	-38,217	292,3	46,1
4OH403	100	8,74E-03	109,82	-45,116	259,1	10,2
4OH403	150	1,15E-02	81,495	-32,445	243,6	-9,9
4OH403	200	1,22E-02	85,482	-32,499	243,7	-6,5
4OH403	250	1,27E-02	76,685	-29,834	241	-14
4OH403	300	1,31E-02	71,883	-29,611	241,1	-18,2
4OH403	350	1,28E-02	73,129	-31,947	243,4	-16,9
4OH403	400	1,30E-02	73,814	-30,878	242,3	-16,4
4OH403	425	1,35E-02	67,322	-26,303	237,9	-22,5
4OH403	450	1,30E-02	68,885	-26,425	237,9	-21,1
4OH403	475	1,28E-02	69,574	-27,573	239,1	-20,4
4OH403	500	1,28E-02	67,718	-30,095	242	-21,7
4OH403	525	1,38E-02	60,872	-9,558	219,7	-29,5
4OH403	550	1,35E-02	64,416	-27,062	239,1	-25
4OH403	575	1,24E-02	80,852	-31,947	243,1	-10,4
4OH403	600	2,36E-03	73,202	48,901	160,9	-7,1
4OH403	625	1,86E-03	141,99	50,632	149,8	34,4
4OH403	650	6,11E-03	317,7	57,308	98,9	-18,9
4OH433	0	7,27E-03	106,94	-14,657	359	10,9
4OH433	100	5,46E-03	350,71	-36,296	91,8	-69,9
4OH433	150	7,32E-03	351,66	-26,463	110,4	-78,6
4OH433	200	7,61E-03	324,64	-23,72	143,5	-56,6
4OH433	250	7,87E-03	337,25	-17,064	158,5	-68,3
4OH433	300	7,17E-03	331,7	-18,121	154,8	-63,1
4OH433	350	9,04E-03	340,31	-7,4991	185,7	-68,1
4OH433	400	8,96E-03	328,04	-12,444	165,1	-58,7
4OH433	425	8,76E-03	320,29	-3,0751	175,8	-48,3
4OH433	450	1,02E-02	321,14	-9,2626	167,3	-51,3
4OH433	475	1,02E-02	324,3	-9,4029	168,6	-54,3
4OH433	500	1,06E-02	325,4	-10,755	166,9	-55,8
4OH433	500	4,10E-03	319,47	5,1839	185,8	-43,8
4OH433	550	9,90E-03	321,11	-14,694	158,7	-52,6
4OH433	575	8,36E-03	325,76	-13,071	163	-56,7
4OH433	600	9,89E-03	327,08	-12,32	164,9	-57,8
4OH433	625	1,02E-02	327,2	-15,616	158,9	-58,6
4OH433	650	7,11E-03	325,14	-23,091	144,6	-57
4OH433	675	3,40E-03	15,973	-32,373	27,5	-69,7
4OH443	0	1,92E-02	356,51	6,964	247,9	-62,8
4OH443	100	9,50E-03	355,64	14,129	248	-55,6
4OH443	150	5,06E-03	328,02	-7,3744	183,1	-56,6
4OH443	200	8,15E-03	338,92	0,91438	208,4	-60,6
4OH443	250	6,18E-03	350,71	10,729	238,1	-57,9
4OH443	300	2,67E-03	315,46	3,6487	188,9	-40,3
4OH443	350	3,96E-03	341,3	-31,7	126,9	-69,6
4OH443	400	5,04E-03	339,95	11,804	222	-52,6
4OH443	425	3,85E-03	346,94	-1,7855	218,9	-67,8
4OH443	450	3,81E-03	325,67	24,174	216,8	-34,6
4OH443	475	3,31E-03	344,89	12,937	230	-53,9
4OH443	550	4,52E-03	331,79	3,9362	203,2	-53,4
4OH443	575	5,39E-03	323,75	-14,943	167,9	-55,1
4OH443	600	5,31E-03	317,19	-12,728	168,8	-48,4
4OH454	0	2,58E-02	130,48	27,069	317,4	38,5
4OH454	100	9,96E-03	122,93	32,628	312,9	30,8
4OH454	150	1,19E-02	95,497	52,888	294,8	8,1
4OH454	200	8,48E-03	98,584	79,074	268,4	7,5
4OH454	250	5,64E-03	116,87	63,459	282,1	17,1
4OH454	300	8,20E-03	75,336	82,243	265	4
4OH454	350	7,29E-03	298,89	87,395	255,2	4,7
4OH454	400	6,92E-03	15,061	65,709	263,9	-17,4
4OH454	425	8,25E-03	323,08	74,066	247,9	-6,8
4OH454	450	8,04E-03	326,82	72,003	247,6	-9,1
4OH454	475	7,60E-03	280,06	74,27	242	3,1
4OH454	500	8,30E-03	294,79	72,789	241,9	-1,3
4OH454	525	7,69E-03	291,7	74,068	242,7	0
4OH454	550	6,56E-03	298,99	66,243	236,8	-5,7
4OH454	575	6,67E-03	278	67,86	235,6	2,6
4OH454	600	6,03E-03	299,55	64,711	235,5	-6,6
4OH454	625	2,36E-03	109,82	39,178	308	19,1
4OH454	650	1,66E-03	255,07	39,757	207,1	15,3

Table S2. Thermal demagnetization data.

(1) Sample = sample name
(2) Temperature (°C)
(3) NRM intensity (A/m)
(4) Core Declination (°E)
(5) Core Inclination (°)
(6) Geographic Declination (°E)
(7) Geographic Inclination (°)

4OH454	675	4,96E-03	275,12	28,283	196,2	-1,6
4OH468	0	1,33E-02	132,59	-15,314	12,7	40,4
4OH468	100	8,46E-03	140,02	-33,749	38,2	38,9
4OH468	150	7,76E-03	142,52	-41,845	47,7	35,4
4OH468	200	6,83E-03	161,89	-41,902	62,7	44,1
4OH468	250	5,59E-03	183,79	-60,812	83,6	28,1
4OH468	300	5,99E-03	208,15	-57,236	98,2	27,5
4OH468	350	6,83E-03	212,13	-69,866	92,5	16
4OH468	400	5,19E-03	201,65	-56,377	95,1	30
4OH468	425	4,69E-03	215,24	-52,131	105,4	29,2
4OH468	450	4,62E-03	217,2	-58,738	101,5	23,5
4OH468	475	5,64E-03	215,12	-63,07	97,7	20,8
4OH468	500	5,06E-03	233,18	-65,045	101,8	13,7
4OH468	550	5,21E-03	188,2	-63,645	85,5	25,1
4OH468	575	5,65E-03	243,97	-59,179	109,6	12,1
4OH468	600	4,84E-03	215,9	-57,632	101,7	24,8
4OH468	625	5,47E-03	213,42	-50,372	105,8	31,3
4OH468	650	3,03E-03	218,1	-37,159	120,2	38,1
4OH468	675	1,18E-03	243,5	-0,82776	170,1	26,5
4OH483	0	5,41E-03	132,06	76,769	354,4	6,8
4OH483	100	3,51E-03	45,599	54,64	11,8	-25,7
4OH483	150	4,19E-03	12,369	56,149	352,9	-34,9
4OH483	200	2,96E-03	340,95	6,2095	267,6	-70,5
4OH483	250	3,04E-03	354,7	4,5244	280,2	-84,1
4OH483	300	2,29E-03	343,01	-9,0563	221,1	-69,8
4OH483	350	3,14E-03	353,03	-27,048	177,1	-60,2
4OH483	400	2,40E-03	9,6599	-6,2246	115	-77,3
4OH483	425	3,63E-03	358,42	-2,2078	185,1	-85,5
4OH483	450	3,02E-03	0,57678	9,3371	348,9	-82,6
4OH483	475	3,56E-03	14,273	-6,9473	106,8	-73,2
4OH483	500	3,11E-03	357,97	5,1579	311,8	-86,2
4OH483	550	3,97E-03	10,913	-2,4571	96,7	-78,2
4OH483	575	3,44E-03	2,6864	7,5094	10,3	-83,9
4OH483	600	3,01E-03	0,3807	0	153,7	-88
4OH484	0	6,04E-03	272,98	-27,086	314,4	-2,7
4OH484	100	3,43E-03	5,1783	-20,479	237,9	-68,9
4OH484	150	3,67E-03	355,28	-7,1978	284,6	-81,4
4OH484	200	4,01E-03	18,957	-17,997	206,5	-64,1
4OH484	250	3,63E-03	27,553	-1,8924	165,6	-62,4
4OH484	300	4,49E-03	29,33	-7,8045	177,1	-59,7
4OH484	350	3,35E-03	40,135	4,628	154,3	-49,6
4OH484	400	4,43E-03	25,753	-4,7863	172,4	-63,8
4OH484	425	4,04E-03	43,191	-2,9811	165,9	-46,7
4OH484	450	3,43E-03	30,935	-6,3693	173,8	-58,5
4OH484	450	3,67E-03	44,007	-0,78046	162,6	-46
4OH484	475	3,33E-03	45,249	-12,145	178,4	-43,5
4OH484	500	3,69E-03	29,482	-17,525	194,2	-56,1
4OH484	525	3,24E-03	41,917	-20,988	191,4	-44
4OH484	550	3,23E-03	27,121	-3,5492	169,2	-62,7
4OH484	575	3,11E-03	34,1	-4,2383	169	-55,7
4OH484	600	2,85E-03	36,505	-7,6636	174,2	-52,8
4OH484	625	1,03E-03	28,172	8,6327	143,7	-60,6
4OH484	650	1,28E-03	24,825	-19,473	201,6	-58,8
4OH484	675	1,63E-03	328,51	8,7718	358	-57,4
4OH508	0	5,70E-03	258,89	-53,747	286,5	9
4OH508	100	3,00E-03	304,35	-71,379	265,9	-7,5
4OH508	150	3,76E-03	55,088	-71,845	235,6	-7,4
4OH508	200	4,50E-03	41,9	-40,954	214,1	-31,8
4OH508	250	5,95E-03	45,659	-51,676	221,7	-23,1
4OH508	300	5,62E-03	55,849	-47,564	214,1	-19,9
4OH508	350	5,44E-03	60,328	-44,564	209,8	-18,4
4OH508	400	5,34E-03	52,82	-46,289	214	-22,3
4OH508	425	5,96E-03	60,779	-43,214	208,3	-18,6
4OH508	450	6,61E-03	65,124	-38,681	202,7	-17,2
4OH508	475	6,33E-03	64,99	-38,696	202,7	-17,3
4OH508	500	6,48E-03	66,977	-37,475	201	-16,1
4OH508	525	6,65E-03	65,611	-38,754	202,6	-16,8
4OH508	550	6,69E-03	73,593	-35,438	197,6	-11,5
4OH508	575	6,69E-03	69,113	-35,212	198,3	-15,1
4OH508	600	6,56E-03	67,868	-38,254	201,6	-15,3
4OH508	625	6,86E-03	64,045	-37,3	201,6	-18,4
4OH508	650	5,30E-04	289,96	-40,312	297,9	-13,1
4OH528	0	1,68E-02	233,71	-4,857	338,9	36,5
4OH528	100	8,70E-03	248,31	14,586	358,2	19,2
4OH528	150	8,98E-03	256,68	5,3039	347,3	12,6
4OH528	200	4,78E-03	233,86	40,602	29	22,2
4OH528	250	6,07E-03	247,77	18,954	2,9	18,8
4OH528	300	6,14E-03	252,91	19,599	2,4	13,9
4OH528	350	4,37E-03	255,93	-3,8047	338,1	14,4
4OH528	400	5,42E-03	239,48	24,854	11,3	24,5
4OH528	425	4,69E-03	259,63	27,395	9	6,4
4OH528	450	4,08E-03	250,85	29,862	13,2	13,4
4OH528	475	5,14E-03	245,86	27,723	12,3	18,1
4OH528	500	3,85E-03	250,93	27,176	10,5	14
4OH528	550	3,91E-03	250	27,221	10,7	14,8
4OH528	575	3,10E-03	252,61	26,259	9,2	12,7
4OH528	600	3,13E-03	240,09	33,566	19,9	20,8
4OH528	625	3,60E-03	240,1	51,807	37,1	13
4OH548	0	2,25E-02	232,57	-19,158	324,2	35,8
4OH548	100	8,06E-03	252,43	-33,918	311,7	15,7
4OH548	150	6,65E-03	258,94	-36,877	309,4	10
4OH548	200	6,68E-03	260,79	-37,409	309	8,5
4OH548	250	6,98E-03	267,73	-46,66	299,9	3
4OH548	300	6,11E-03	271,18	-37,54	308,9	0,3
4OH548	350	5,22E-03	271,94	-42,73	303,7	-0,1
4OH548	400	5,14E-03	282,47	-37,52	308,1	-8,6
4OH548	425	4,39E-03	276,21	-27,833	318,4	-4,5
4OH548	450	4,12E-03	270,15	-24,513	322	0,7
4OH548	475	4,44E-03	282,53	-31,65	313,9	-9,6
4OH548	500	4,93E-03	272,24	-28,054	318,4	-1
4OH548	525	4,08E-03	290,87	-43,16	301	-13,6
4OH548	550	4,32E-03	268,96	-27,273	319,3	1,8
4OH578	0	2,57E-02	262,11	-20,743	321,8	8,1
4OH578	100	8,53E-03	247,85	-26,77	314,6	20,6
4OH578	150	1,11E-02	277,77	-20,558	321,5	-6,6
4OH578	200	7,03E-03	249,17	-29,368	312	19,1
4OH578	250	5,92E-03	259,53	-55,484	286,7	7,6
4OH578	300	4,39E-03	265,29	-63,708	278,8	3,9
4OH578	350	4,20E-03	234,85	-31,111	307	30,7
4OH578	400	6,28E-03	266,26	-44,874	297,6	4,1
4OH578	425	4,53E-03	259,98	-58,668	283,6	6,9
4OH578	450	3,85E-03	248,65	-68,198	273	9,6
4OH578	475	3,21E-03	239,9	-75,616	265,1	9,1
4OH578	500	3,81E-03	229,88	-72,223	266,4	13,3
4OH578	550	5,44E-03	224,3	-77,68	261,2	10,8
4OH578	575	4,89E-03	211,66	-73,235	261,6	16,2
4OH578	600	2,47E-03	191,69	-61,371	258,9	30
4OH578	625	2,42E-03	222,33	-38,095	294,1	37,1

OH5

5OH130	0	1,79E-02	67,04	63,749	5,1	24,6
5OH130	100	1,03E-02	60,573	58,446	7,3	18,8
5OH130	150	6,68E-03	45,292	51,414	5,1	7,8
5OH130	200	5,04E-03	32,113	55,915	356	7,8
5OH130	250	4,23E-03	38,876	66,928	353,6	19
5OH130	300	3,24E-03	20,045	60,896	348,3	10,2
5OH130	350	1,44E-03	64,867	71,953	357,2	28,8
5OH130	400	2,13E-03	2,596	55,871	340	3,9
5OH130	425	1,90E-03	322	77,684	329,9	28
5OH130	450	1,78E-03	5,69	71,878	340,4	20
5OH130	475	1,67E-03	326,79	63,884	324	15,2
5OH130	500	1,72E-03	303,55	58,405	311,3	17,2
5OH130	525	1,19E-03	265,12	56,401	297,2	33,4
5OH130	550	1,00E-03	300,47	31,681	291,3	-1
5OH130	575	1,21E-03	280,55	57,595	302,5	26,3
5OH130	600	1,30E-03	271,96	42,056	284,7	23,1
5OH130	625	1,31E-03	282,87	59,906	305,4	26,4
5OH130	650	8,21E-04	219,22	45,509	275,7	60,1
5OH130	675	8,65E-04	222,66	48,582	281,5	57,7
5OH181	0	1,46E-02	250,24	42,374	315,4	29,6
5OH181	100	6,75E-03	260,1	39,908	314	21,8
5OH181	150	5,37E-03	258	35,49	309	22,5
5OH181	200	5,21E-03	261,76	35,192	309,4	19,5
5OH181	250	3,02E-03	254,15	33,152	305,7	25,1
5OH181	300	2,52E-03	245,47	34,526	305,9	32,4
5OH181	350	2,12E-03	259,84	29,743	303,4	19,6
5OH181	400	1,76E-03	249,96	28,027	299,3	27,5
5OH181	425	1,44E-03	230,02	16,79	280,5	42,8
5OH181	450	9,74E-04	250,84	22,196	293,2	25,3
5OH181	475	1,23E-03	238,55	29,816	299,3	37,7
5OH181	500	1,04E-03	254,41	-1,214	271,1	13,8
5OH181	525	1,02E-03	266,68	1,801	278,9	3,8
5OH181	550	1,13E-03	257,41	-3,8174	270	10
5OH181	575	3,30E-04	306,68	42,25	331,6	-8,3
5OH181	600	5,59E-04	222,09	26,323	292,4	51,8
5OH215	0	9,47E-03	136,92	26,669	80,6	44,6
5OH215	100	5,38E-03	142,16	28,896	75,1	48,2
5OH215	150	4,03E-03	139,79	35,112	68,4	43,6
5OH215	200	3,25E-03	132,22	31,706	75,8	39,2
5OH215	250	2,52E-03	148,58	35,656	62,1	49,4
5OH215	300	2,13E-03	133,97	40,603	64,7	37,1
5OH215	350	1,60E-03	143,96	22,864	83	51,9
5OH215	400	1,38E-03	135,58	65,877	39,7	23,6
5OH215	425	1,47E-03	161,76	75,302	26,4	20,9
5OH215	450	1,05E-03	181,42	72,113	21	24,9
5OH215	475	1,12E-03	139,09	55,438	47,4	31,7
5OH215	500	1,40E-03	150,35	70,191	32,1	24
5OH215	550	1,40E-03	245,04	74,083	6,7	13,4
5OH215	575	9,78E-04	334,18	87,981	20,6	5,2
5OH215	600	1,06E-03	259,9	85,983	17,5	7,7
5OH215	625	8,04E-04	20,134	14,337	64,6	-60,8
5OH244	0	5,47E-03	233,54	24,045	320,5	37,6
5OH244	100	2,63E-03	222,55	35,765	338,5	44,3
5OH244	150	2,57E-03	216,44	19,323	316,3	53,9
5OH244	200	1,62E-03	207	39,427	353	52,8
5OH244	250	1,56E-03	171,41	55,456	35,4	45
5OH244	300	1,53E-03	173,09	29,342	46,9	70,6
5OH244	350	1,47E-03	154,83	45,736	55,5	49,2
5OH244	400	1,03E-03	66,267	82,911	35,1	8,1
5OH244	425	9,51E-04	153,91	37,262	65,9	54,8
5OH244	450	9,74E-04	145,09	22,459	95	54,8
5OH244	475	8,71E-04	128,45	33,662	84,2	37,9
5OH244	500	9,46E-04	118,79	31,395	88,4	30,2
5OH244	525	8,77E-04	132,79	16,222	106,5	43,9
5OH244	550	1,22E-03	128,64	22,035	98,9	39,8
5OH244	575	1,03E-03	102,1	5,5956	115,2	12,9
5OH244	600	1,54E-03	115,96	20,631	100,9	28
5OH244	625	1,01E-03	78,304	22,174	94,4	-6,4
5OH244	675	4,98E-04	357,4	74,609	27,8	-4,4
5OH266	0	1,04E-02	249,29	25,712	325,3	24,8
5OH266	100	4,66E-03	247,54	20,218	319	25,8
5OH266	150	3,31E-03	245,24	19,051	317,4	27,8
5OH266	200	3,62E-03	244,11	18,511	316,7	28,8
5OH266	250	2,31E-03	233,55	16,604	313	38,6
5OH266	300	2,09E-03	228,62	25,684	324,9	43,4
5OH266	350	2,01E-03	220,54	17,029	312,2	51,1
5OH266	400	1,23E-03	187,35	19,768	338,8	81,5
5OH266	425	1,23E-03	201	15,019	306,3	69,7
5OH266	450	1,05E-03	180,29	18,366	28,9	86,6
5OH266	475	1,05E-03	193,06	10,127	284,1	76,4
5OH266	500	9,99E-04	188,67	14,202	299,2	81,6
5OH266	525	1,04E-03	197,63	12,478	297,3	72,7
5OH266	550	6,64E-04	177,76	15,827	102,3	87,7
5OH266	575	5,91E-04	237,53	37,41	338,9	34,7
5OH266	600	8,35E-04	273,24	26,928	330,3	3,9
5OH266	625	3,64E-04	206,71	17,587	313	64,3
5OH291	0	1,27E-02	272,53	36,518	332,3	8,1
5OH291	100	4,56E-03	280,44	49,187	346,2	6,2
5OH291	150	3,74E-03	258,76	31,77	325,1	18,2
5OH291	200	3,25E-03	288,68	46,913	346,2	0,2
5OH291	250	2,46E-03	327,38	40,907	359,9	-24,7
5OH291	300	1,85E-03	302,33	65,455	5,9	3,1

Table S2. Thermal demagnetization data.

(1) Sample = sample name
(2) Temperature (°C)
(3) NRM intensity (A/m)
(4) Core Declination (°E)
(5) Core Inclination (°)
(6) Geographic Declination (°E)
(7) Geographic Inclination (°)

5OH291	350	1,85E-03	331,47	55,605	10,4	-13,5
5OH291	400	1,97E-03	337,41	32,588	2,9	-35,9
5OH291	425	2,03E-03	341,62	35,775	8,4	-34,4
5OH291	450	1,42E-03	359,56	24,117	25,9	-48,9
5OH291	475	1,47E-03	19,921	15,638	58,6	-51,9
5OH291	500	1,66E-03	32,013	19,929	68,2	-41,5
5OH291	550	2,07E-03	43,444	14,644	82,6	-36,7
5OH291	575	2,08E-03	26,409	18,304	64,1	-46,2
5OH291	600	1,66E-03	44,469	14,627	83,3	-35,9
5OH291	625	2,15E-03	43,434	8,7599	88,9	-39,9
5OH291	650	6,07E-04	221,76	22,045	310,8	50,4
5OH291	675	4,55E-04	268,34	40,712	335,7	12,2
5OH326	0	1,40E-02	227,13	59,744	314,7	31,9
5OH326	100	8,08E-03	229,83	57,266	311,4	31,9
5OH326	150	5,81E-03	216,27	55,855	315,3	38,8
5OH326	200	3,79E-03	211,34	51,375	313,7	44
5OH326	250	3,85E-03	221,44	55,887	313	36,6
5OH326	300	2,64E-03	210,72	40,601	302	51,5
5OH326	350	1,51E-03	201,51	36,623	305	59,5
5OH326	400	1,60E-03	221,18	44,147	300,2	43,1
5OH326	425	1,50E-03	179,86	37,321	340,8	65,7
5OH326	450	6,97E-04	184,89	46,409	334,4	56,3
5OH326	475	8,50E-04	159,42	37,199	14,1	59,6
5OH326	500	8,76E-04	195,48	29,709	302,3	68
5OH326	525	8,05E-04	174,37	-0,35592	137,4	75,5
5OH326	550	1,05E-03	159,93	-1,5248	105,2	65,4
5OH326	575	6,88E-04	139,55	4,1691	79,3	49,1
5OH326	600	1,15E-03	159,44	-6,0825	112,3	62,1
5OH326	625	8,04E-04	244,58	0,92657	245,4	24,9
5OH326	650	4,56E-04	87,557	34,607	259,9	32,8
5OH326	650	5,47E-04	239,07	14,817	36,2	5,4
5OH326	675	4,42E-04	188,33	25,044	308,6	75,6
5OH351	0	1,96E-02	244,94	56,261	317,8	21,3
5OH351	100	1,04E-02	256,47	61,724	322,1	14,3
5OH351	150	9,36E-03	256,6	60,755	321,1	14,4
5OH351	200	9,33E-03	258,35	60,442	320,7	13,6
5OH351	250	8,41E-03	255,1	58,788	319,2	15,4
5OH351	300	3,87E-03	257,04	68,386	328,9	13,1
5OH351	350	2,74E-03	250,91	72,451	333,4	14,3
5OH351	400	2,40E-03	263,16	77,905	338,3	10,2
5OH351	425	2,28E-03	259,44	74,741	335,2	11,5
5OH351	450	1,91E-03	240,26	70,286	332,5	18,2
5OH351	475	1,87E-03	254,32	78,246	338,9	12
5OH351	500	1,91E-03	268,22	83,243	343,7	0,2
5OH351	525	1,04E-03	184,45	79,972	349,7	19
5OH351	550	9,78E-04	70,279	49,555	28,3	-5,6
5OH377	0	1,19E-02	243,16	-1,2982	327,6	26
5OH377	100	5,64E-03	245,85	-4,9812	324,3	22,6
5OH377	150	3,79E-03	262,19	8,1839	341,1	9,2
5OH377	200	3,03E-03	257,03	-9,8783	322,1	10,6
5OH377	250	2,40E-03	256,91	-13,223	318,8	9,9
5OH377	300	1,88E-03	245,52	-14,082	314,8	20,4
5OH377	350	1,96E-03	270,3	-11,482	323,3	-2,5
5OH377	400	1,52E-03	259,37	-19,217	313,5	6,2
5OH377	425	1,09E-03	269,68	-0,15784	334,3	0,3
5OH377	450	7,72E-04	255,36	-8,3423	323,3	12,6
5OH377	475	7,63E-04	242,39	-21,046	306,8	20,9
5OH377	500	8,14E-04	268,7	-35,948	298,9	-5,4
5OH377	525	5,55E-04	221,92	-22,438	295,2	37

Rhino Cave

R108	0	5,76E-03	147,05	34,501	295,9	46,8
R108	100	2,68E-03	143,7	47,057	285,2	36,8
R108	150	3,37E-03	68,488	64,08	279,1	-5,6
R108	200	1,74E-03	292,43	70,203	236,7	-3,6
R108	250	2,29E-03	56,937	54,128	285,6	-15,2
R108	300	1,07E-03	23,869	41,021	278,5	-40
R108	350	1,82E-03	39,782	47,861	284	-27,6
R108	400	1,86E-03	37,511	58,744	274,7	-20,5
R108	425	2,10E-03	65,834	32,182	309,3	-18
R108	450	2,19E-03	69,929	46,491	296,1	-10,7
R108	475	2,40E-03	73,464	44,256	299	-8,9
R108	500	1,93E-03	84,947	48,359	296,4	-0,4
R195	0	9,84E-04	199,16	32,595	225,1	64,5
R195	100	1,81E-03	289,71	13,536	193,5	-15,2
R195	150	2,17E-03	298,81	14,494	197,7	-23,1
R195	200	2,14E-03	303,27	12,66	197,8	-27,8
R195	250	2,28E-03	304,53	10,6	196,1	-29,7
R195	300	2,29E-03	302,37	7,3383	191,8	-29
R195	350	2,50E-03	300,77	-1,6025	181,4	-30,2
R195	400	2,80E-03	306,58	10,297	196,8	-31,7
R195	450	2,13E-03	302,46	9,2341	193,8	-28,4
R195	475	2,21E-03	310,48	11,533	200	-34,7
R195	500	2,15E-03	307,39	9,3708	196,1	-32,8
R195	525	1,96E-03	309,08	7,5697	194,9	-35,1
R195	550	1,93E-03	304,38	13,019	198,6	-28,6
R195	575	1,93E-03	305,31	13,157	199,2	-29,4
R195	600	2,22E-03	304,73	11,124	196,8	-29,7
R195	625	1,54E-03	314,48	16,007	207,1	-35,9
R216	0	3,42E-03	144,95	59,023	286,9	29,6
R216	100	1,69E-03	225	64,543	247,8	22,4
R216	150	1,57E-03	258,79	63,306	240,5	9,5
R216	200	1,59E-03	279,83	52,388	230	-2
R216	250	1,21E-03	281,69	38,806	216,9	-5,9
R216	300	1,16E-03	296,38	43,877	225	-15
R216	350	1,25E-03	284,42	40,183	218,7	-7,7
R216	400	8,87E-04	281,56	30,571	208,7	-7,3
R216	425	1,15E-03	278,54	44,539	222,1	-2,5
R216	450	1,25E-03	282,22	47,987	226	-4,4
R216	475	1,21E-03	280,48	45,194	223	-3,8
R216	500	8,78E-04	279,14	55,478	233	-1
R216	525	1,01E-03	273,65	50,225	227,3	1,5
R216	550	7,04E-04	304,11	77,719	256,9	-1,9
R216	575	7,18E-04	277,88	43,878	221,4	-2,2
R246	0	5,34E-03	164	-30,365	53,5	57,8
R246	100	4,73E-04	167,38	-4,2463	0,4	77,2
R246	150	1,56E-03	63,814	-61,501	54,2	-10,4
R246	200	6,29E-04	352,61	-25,435	94,2	-61,6
R246	250	6,57E-04	9,2902	-0,34903	4,1	-80,4
R246	300	4,13E-04	10,925	-23,223	57,7	-62,6
R246	350	5,24E-04	0,24538	-26,907	79,5	-61,1
R246	400	5,86E-04	336,21	-6,3671	150,4	-64,8
R246	450	4,51E-04	349,59	-56,476	86,7	-30,9
R246	475	1,15E-03	15,969	-73,235	75,3	-14,1
R246	500	6,06E-04	355,95	-47,273	83,6	-40,6
R246	525	4,20E-04	84,103	-59,325	49,5	-1,3
R246	550	4,46E-04	94,203	-31,16	21,1	4,6
R246	575	3,47E-04	113,2	9,4431	338,9	22,5
R246	600	5,09E-04	92,396	-19,986	9,9	2,9
R246	625	6,25E-04	91,554	-27,746	17,7	2,3
R246	650	7,50E-04	88,873	-35,55	25,6	0,2
R246	675	9,13E-04	69,157	4,2707	346,2	-20,9
R276	0	5,90E-03	165,3	-31,155	47,1	50,3
R276	100	3,68E-03	273,74	0,46735	157,9	-3,7
R276	150	2,14E-03	331,1	-12,11	142,8	-60,8
R276	200	3,62E-03	296,49	4,2785	164,7	-25,8
R276	250	3,21E-03	310,42	15,161	180,9	-36,5
R276	300	4,03E-03	298,61	6,2624	167,3	-27,5
R276	350	2,71E-03	304,9	22,592	187	-29
R276	400	1,99E-03	291,66	-10,654	147,9	-22,3
R276	425	1,99E-03	320,05	-8,9434	150,2	-50,3
R276	450	1,46E-03	315,59	-7,0358	153,1	-45,9
R276	475	1,57E-03	314,12	-3,6544	157,7	-44,2
R276	500	1,09E-03	316,19	-13,577	143,6	-46,2
R276	525	1,09E-03	305,64	-3,0032	157,6	-35,7
R276	550	1,14E-03	310,38	-19,341	136,7	-40
R276	575	1,54E-03	312,34	-34,581	117,2	-37,7
R276	600	9,29E-04	324,76	-25,497	123	-51,1
R276	625	5,52E-04	343,72	-56,671	78,2	-37,7
R276	650	1,04E-03	130,49	-17,407	3,5	35,8
R276	675	6,72E-04	149,14	9,5906	328,3	59,2
R316	0	2,70E-03	196,39	-23,136	98,1	44
R316	100	2,82E-03	308,15	9,5801	192,2	-31
R316	150	3,65E-03	315,78	2,6725	189,5	-41,1
R316	200	3,59E-03	323,13	16,318	209,4	-38,7
R316	250	2,94E-03	323,84	-10,172	177,3	-53,8
R316	300	3,00E-03	319,82	11,329	201,9	-39,6
R316	350	3,05E-03	318,13	12,299	201,5	-37,6
R316	400	2,67E-03	322,91	15,65	208,6	-39
R316	450	2,42E-03	311,8	11,209	196,1	-33,2
R316	475	2,04E-03	319,09	27,185	215,5	-28,4
R316	500	2,00E-03	300,02	24,318	201,5	-16,7
R316	525	1,56E-03	286,48	30,431	201,1	-3,2
R316	550	1,68E-03	283,31	26,453	196,3	-2,4
R316	575	1,43E-03	300,14	16,4	194,4	-20,9
R316	600	1,50E-03	281,11	36,813	205,1	3,4
R316	625	1,51E-03	278,88	42,257	209,5	7
R316	650	1,39E-03	256,69	26,782	189,1	20,3
R316	675	1,40E-03	271,79	38,845	204,5	11
R342	0	2,76E-03	143,83	18,132	314,8	52,7
R342	100	1,19E-03	127,19	74,239	260	15,3
R342	150	1,08E-03	344,93	71,076	242,1	-12,3
R342	200	1,10E-03	324,79	75,133	238,4	-6,2
R342	250	6,68E-04	1,5911	86,909	247,1	2,9
R342	300	8,20E-04	351,85	61,785	242,9	-21,9
R342	350	4,50E-04	332,99	49,8	227,3	-29,5
R342	400	8,35E-04	32,082	55,37	266,2	-23,1
R342	400	7,46E-04	34,34	60,515	264	-18,3
R342	450	6,58E-04	62,103	63,978	270	-6,3
R342	475	7,71E-04	76,623	64,057	272,2	-0,4
R342	500	6,24E-04	58,577	46,769	284,5	-16,2
R342	525	8,19E-04	99,97	50,606	286,5	11
R342	550	6,95E-04	93,384	55,921	281,3	6,9
R342	575	8,91E-04	106,44	42,83	294,1	16,1
R342	600	6,68E-04	113,25	44,219	291,8	20,7
R342	625	7,64E-04	111,36	53,619	282,4	17,4
R342	650	4,78E-04	195,84	52,207	234	42
R342	675	2,21E-04	4,316	-15,51	43,3	-79,6
R392	0	2,17E-03	156,21	-20,252	32,8	54,1
R392	100	9,91E-04	125,13	38,564	301,6	31,5
R392	150	1,25E-03	314,49	17,494	192,2	-38,8
R392	200	1,02E-03	313,44	13,751	187,2	-39,3
R392	250	1,10E-03	320,24	6,948	181,6	-48
R392	300	8,86E-04	337,47	12,909	204,6	-60
R392	350	8,49E-04	347,3	-16,494	124,5	-74,4
R392	400	7,83E-04	339,71	-33,3	105,8	-57,7
R392	425	7,14E-04	340,5	-3,0512	173,5	-70,2
R392	450	7,43E-04	337,54	-6,8764	161,9	-67,7
R392	475	7,13E-04	316,8	-11,731	153,3	-47,2
R392	500	4,60E-04	315,11	-37,449	119,3	-39,2
R392	525	4,21E-04	39,995	-34,104	25	-44,3
R392	550	2,31E-04	56,217	-42,399	28,3	-29,3
R417	0	5,06E-03	193,79	26,511	220,7	61,2
R417	100	2,27E-03	229,69	79,017	238,6	8,1
R417	150	1,87E-03	119,25	66,859	267,5	12
R417	200	1,62E-03	252,64	66,008	223,9	7,9
R417	250	1,47E-03	275,12	79,459	236,5	0
R417	300	1,14E-03	223,88	86,366	244,5	3,6
R417	350	1,01E-03	321,6	76,191	238,3	-9,8
R417	400	8,75E-04	254,7	44,39	202,3	11,6
R417	425	6,88E-04	341,57	71,22	240,9	-16,8
R417	450	9,06E-04	34,223	72,756	256,9	-13,2
R417	475	1,08E-03	38,756	69,869	259,9	-14,6
R417	500	9,90E-04	51,757	81,275	253,9	-4,4
R417	525	9,30E-04	58,861	79,581	255,9	-4,4
R417	550	8,77E-04	50,425	73,139	260,1	-9,7
R417	575	1,05E-03	59,21	71,033	263,4	-8,6
R417	600	1,03E-03	75,553	72,88	263,6	-3,3
R417	625	1,12E-03	45,265	65,818	264,6	-15,8
R417	650	4,19E-04	345,82	45,392	233,6	-41,9
R417	675	2,01E-04	183,32	39,282	242,9	51,6
R431	0	3,67E-03	155,07	25,885	291,6	51,6

Table S2. Thermal demagnetization data.

(1) Sample = sample name
(2) Temperature (°C)
(3) NRM intensity (A/m)
(4) Core Declination (°E)
(5) Core Inclination (°)
(6) Geographic Declination (°E)
(7) Geographic Inclination (°)

R431	100	1,93E-03	210,8	9,6367	188,1	56,4
R431	150	2,16E-03	189,64	5,8415	209,6	76,2
R431	200	1,80E-03	210,54	13,224	194,1	55,1
R431	250	1,31E-03	213,8	-23,917	129,4	51,8
R431	300	1,09E-03	209,71	-1,3121	168,4	60,2
R431	350	1,37E-03	205,01	-4,6832	161,5	65,1
R431	400	9,33E-04	207,58	-1,7803	167,8	62,4
R431	425	1,10E-03	193,35	5,0595	198,1	73,9
R431	450	7,76E-04	206,1	-2,069	167,5	63,9
R431	475	1,20E-03	202,02	22,26	215,6	56
R431	500	8,65E-04	186,75	13,235	232,8	71,5
R459	0	3,03E-03	187,58	28,989	242,6	54,3
R459	100	1,91E-03	199,69	18,053	215,4	59,1
R459	150	1,58E-03	201,67	7,2945	195,3	64,6
R459	200	1,39E-03	205,35	0,61638	177,8	63,8
R459	250	1,41E-03	209,29	-1,541	171,5	60,4
R459	300	1,32E-03	202,84	-2,5665	171,6	67
R459	350	1,48E-03	211,95	-12,498	150	57,8
R459	400	1,28E-03	210,11	-6,8932	160,6	60,1
R459	425	1,37E-03	214,32	-6,6164	161,1	55,9
R459	450	1,41E-03	223,5	-11,378	153,8	46,7
R459	475	1,58E-03	222,87	-16,299	146,5	46,8
R459	500	1,40E-03	208,91	-20,902	133,2	58,3
**Sidi Abderrahmane**						
SA1	0	5,53E-04	13,859	-31,493	297,6	-56,8
SA1	100	6,77E-04	15,11	-12,808	268,4	-70,9
SA1	150	7,01E-04	25,68	-7,6259	244,7	-63,6
SA1	200	4,26E-04	13,279	-7,1535	254,8	-75,4
SA1	250	5,84E-04	27,805	7,9661	211,1	-60,9
SA1	300	6,16E-04	35,603	-3,1646	233,5	-54,4
SA1	350	3,22E-04	345,52	-27,998	345,4	-59,7
SA1	400	1,20E-03	313,75	19,815	76,8	-40,1
SA1	425	3,73E-04	320,28	-25,053	14,1	-44,8
SA1	450	1,59E-04	329,82	-36,943	353,8	-44,5
SA1	475	2,89E-04	332,66	-24,129	6,2	-54,9
SA1	500	3,10E-04	333,94	-7,784	34,1	-63,2
SA1	525	4,03E-04	5,0131	-31,696	311,2	-58,9
SA1	550	5,79E-04	320,67	15,224	73,8	-47,9
SA1	575	2,54E-04	206,9	-72,271	327,7	14,8
SA1	600	3,57E-04	3,4474	21,199	147,9	-67,5
SA1	625	4,94E-04	7,0962	-2,5511	241,9	-82,7
SA1	650	5,45E-04	4,862	27,33	148,5	-61,3
SA2	0	9,06E-04	248,58	7,6737	50,3	20,1
SA2	100	4,34E-04	241,01	13,044	57,8	26,2
SA2	150	8,11E-04	263,91	2,3313	42,6	5,8
SA2	200	8,65E-04	229,47	4,71	51,5	39,3
SA2	250	7,50E-04	261,54	-8,821	31,7	9,4
SA2	300	3,78E-04	190,83	29,284	114	52,2
SA2	350	5,82E-04	255,72	4,4372	45,8	13,6
SA2	400	1,10E-03	193,61	6,244	83,5	71
SA2	425	5,07E-04	235,42	42,258	89,2	19,6
SA2	450	4,64E-04	223,66	11,315	62,1	42,9
SA2	475	5,30E-04	245,62	32,685	76,5	16,2
SA2	500	3,02E-04	279,73	19,929	58,5	-11,5
SA2	525	6,97E-04	249,87	54,291	96,1	5,8
SA2	550	8,82E-04	238,68	26,027	72,2	24,2
SA2	575	6,55E-04	240,27	12,44	57,3	27
SA3	0	7,03E-04	284,38	35,57	67,2	-14
SA3	100	5,52E-04	330,13	4,5731	33,7	-60,2
SA3	150	5,45E-04	335,26	11,972	51	-64,3
SA3	200	5,31E-04	325,93	18,558	57,8	-53,7
SA3	250	6,28E-04	324,79	29,807	73,2	-47,9
SA3	300	6,85E-04	309,43	28,113	63,8	-36,3
SA3	350	7,25E-04	309,7	23,404	58,2	-37,8
SA3	400	5,72E-04	299,09	7,1297	37,4	-29,3
SA3	425	7,86E-04	290,45	22,294	53,8	-20,4
SA3	450	7,62E-04	287,55	14,12	45,1	-18
SA3	475	6,88E-04	307,73	18,286	51,4	-37
SA3	500	6,37E-04	283,16	18,011	49,1	-13,7
SA3	525	6,91E-04	281,85	9,4959	40,4	-12,3
SA3	550	6,27E-04	282,92	7,4227	38,2	-13,3
SA3	575	8,40E-04	296,9	4,029	34	-27,1
SA3	600	1,22E-04	41,673	2,8195	211,8	-48,4
SA3	625	2,14E-04	25,346	38,336	152	-48,6
SA3	650	5,45E-04	295,47	9,4069	40	-25,8
SA4	0	2,23E-03	211,52	-50,88	357,8	29,8
SA4	100	1,08E-03	198,64	-37,272	357	46,2
SA4	150	8,37E-04	202,12	-24,356	12,5	55,2
SA4	200	8,33E-04	205,58	-25,31	15,2	52,4
SA4	250	5,49E-04	212,93	-24,211	23,3	48
SA4	300	6,31E-04	248,2	20,034	85,9	21,5
SA4	350	5,14E-04	205,51	-6,3672	45,3	62,9
SA4	400	5,12E-04	230,49	34,637	105,9	33,5
SA4	450	3,21E-04	269,08	54,521	120	3
SA4	475	7,43E-04	217,22	79,87	149,3	11
SA4	500	4,12E-04	282,33	14,773	81,2	-11,1
SA4	525	7,08E-04	317,09	72,488	143,5	-9,8
SA4	550	8,77E-04	45	82,957	160,5	-2
SA4	575	8,29E-04	341,09	53,196	142,3	-31,6
SA4	600	5,49E-04	231,56	53,287	124,6	24,4
SA4	625	6,74E-04	294,73	74,363	141,3	-3,6
SA4	650	3,77E-04	312,38	25,629	100,3	-35,7
SA4	675	4,12E-04	33,425	81,647	160,1	-4
SA5	0	7,43E-04	210,98	20,082	46,7	59,2
SA5	100	5,91E-04	184,75	50,744	112,7	51,1
SA5	150	5,94E-04	176,04	43,018	123,1	58,8
SA5	200	6,64E-04	177,95	44,127	120,3	57,8
SA5	250	2,50E-04	165,47	63,523	125,6	37,5
SA5	300	4,76E-04	211,56	30,401	64,7	55,5
SA5	350	4,20E-04	250,83	58,015	85,3	20,3
SA5	400	1,00E-03	258,22	36,458	62,3	16,5
SA5	425	4,09E-04	315,55	68,941	102,9	-3,3
SA5	450	5,06E-04	263,9	78,181	105,4	13
SA5	475	5,45E-04	313,75	83,142	112,5	7,2
SA5	500	5,81E-04	70,769	85,503	121,8	10,5
SA5	525	5,55E-04	299,74	76,565	105,8	5,1
SA5	550	7,08E-04	29,796	38,322	145	-32,5
SA5	575	8,00E-04	127,69	69,66	135	23,8
SA5	600	4,90E-04	69,717	84,261	123	10
SA5	625	7,29E-04	13,403	70,652	121,9	-6,8
SA5	650	3,85E-04	58,352	14,125	184,9	-26,5
SA5	675	6,26E-04	208,55	38,543	78,8	53,3
SA6	0	1,83E-03	228,74	-38,434	333,9	31,1
SA6	100	1,22E-03	232,87	-37,683	336,4	28,5
SA6	150	1,00E-03	236,57	-36,777	338,6	26,2
SA6	200	9,44E-04	235,05	-35,346	339,6	27,9
SA6	250	7,92E-04	245,78	-37,973	339,9	18,9
SA6	300	6,69E-04	252,02	-48,845	330,2	11,7
SA6	350	4,64E-04	253,82	-37,144	342,2	12,8
SA6	400	5,71E-04	254,61	-32,309	347,2	13
SA6	450	5,41E-04	269,59	-38,543	342	0,3
SA6	475	4,91E-04	288,69	-4,5521	15,7	-18,6
SA6	500	4,98E-04	337,56	2,7608	27,7	-67,4
SA6	525	4,03E-04	300,74	-7,5649	11,7	-30,4
SA6	550	3,32E-04	297,74	-23,081	354,8	-25,4
SA6	575	4,02E-04	299,37	-0,9985	19,4	-29,4
SA6	600	4,36E-04	3,1798	24,53	117,4	-65,3
SA6	625	5,18E-04	316,78	-14,978	359,2	-44,7
SA6	650	2,84E-04	210,14	82,756	106,8	6,3
SA6	675	5,93E-04	353,58	5,0309	58,7	-81,9
SA7	0	1,48E-04	190,06	75,22	168,5	32,5
SA7	100	2,57E-04	3,2491	27,026	175,6	-44,9
SA7	150	2,53E-04	4,7636	17,946	179,2	-53,8
SA7	200	1,96E-04	7,7119	8,5126	188,2	-62,4
SA7	250	1,99E-04	2,8293	5,4092	178,6	-66,4
SA7	300	2,30E-04	352,59	11,561	157,1	-59,5
SA7	350	4,00E-04	351,9	40,224	164,3	-31,3
SA7	400	3,78E-04	310	25,567	122	-24,7
SA7	425	2,96E-04	347,42	29,752	157,1	-40,7
SA7	450	2,87E-04	321,25	3,6018	107,2	-46,1
SA7	475	4,18E-04	274,95	23,841	105,7	2,9
SA7	500	3,53E-04	236,64	19,368	92,6	36,6
SA7	525	6,52E-04	315,16	-37,418	45,7	-46,3
SA8	0	7,33E-04	85,314	65,322	155,6	11,7
SA8	100	6,47E-04	87,325	60,239	160,9	11,7
SA8	150	5,60E-04	107,99	49,653	172,5	23
SA8	200	6,16E-04	133,47	30,767	191,8	44,7
SA8	250	6,26E-04	128,19	48,155	171,1	36,2
SA8	300	5,74E-04	118,49	39,965	182,5	31,3
SA8	350	2,84E-04	132,99	-7,8849	243,5	38,1
SA8	400	1,08E-03	133,23	-2,3447	237,1	40,6
SA8	425	3,15E-04	146,92	8,5807	227,9	57
SA8	450	2,44E-04	116,35	16,407	209,8	29
SA8	475	1,23E-04	169,27	-26,747	296,4	47
SA8	500	2,60E-04	145,75	-12,43	257,7	46,4
SA8	525	1,42E-04	47,441	-22,641	237,7	-44,6
SA8	550	2,72E-04	235,51	-17,126	12,2	26,5
SA8	575	5,61E-05	43,675	11,304	191,7	-39,4
SA8	600	1,95E-04	291,16	70,034	111,8	7,1
SA8	625	2,76E-04	217,49	-2,0749	19	49,2
SA9	0	1,92E-04	14,826	-79,458	319,8	0,8
SA9	100	1,93E-04	342,24	-55,942	333,1	-21,4
SA9	150	2,60E-04	54,583	-50,507	290,4	-12,4
SA9	200	3,14E-04	59,487	-13,803	253,9	-26
SA9	250	9,46E-04	57,279	-38,683	279,1	-17,2
SA9	300	3,79E-04	316,67	-50,096	350,1	-18,2
SA9	350	3,10E-04	30,622	-33,266	290,3	-37
SA9	400	6,64E-04	246,57	-39,104	14,3	25
SA9	425	2,53E-04	325,16	-44,63	349,4	-26,1
SA9	450	2,67E-04	21,071	-21,997	290,5	-51,1
SA9	475	2,40E-04	10,192	-67,838	318,6	-10,8
SA9	500	8,07E-04	289,18	-29,391	19,4	-10,8
SA9	525	2,87E-04	19,395	-9,033	277,8	-62,2
SA9	550	1,72E-04	11,788	-47,005	313,1	-31
SA9	575	9,09E-04	27,553	3,2149	246,3	-61,6
SA10	0	1,02E-03	149,12	-53,705	316,5	41,6
SA10	100	7,93E-04	133,3	-55,141	310,5	33,6
SA10	150	7,33E-04	123,47	-54,64	307,1	28,8
SA10	200	7,55E-04	125,01	-55,075	307,9	29,5
SA10	250	6,87E-04	121,04	-56,577	308,6	26,8
SA10	300	6,92E-04	133,55	-49,943	305,1	36,4
SA10	350	5,22E-04	103,42	-49,326	298,8	17,8
SA10	400	5,24E-04	101,89	-58,752	308,6	16,4
SA10	450	4,94E-04	98,215	-54,481	304,1	14,5
SA10	475	3,63E-04	42,675	-39,762	12,6	-58,2
SA10	500	2,39E-04	46,219	-13,065	277,7	-37,8
SA10	525	5,31E-04	73,301	-32,318	285,8	-7,3
SA10	550	5,95E-04	8,111	-44,37	333,6	-33,2
SA10	575	4,49E-04	80,734	-41,623	292,9	1,2
SA10	600	3,39E-04	112,18	-59,944	310,6	21,4
SA10	625	3,50E-04	137,57	-19,183	266	48,6
SA10	650	3,80E-04	50,339	-66,408	322,5	-3,4
SA10	675	3,40E-04	287,1	2,0211	68,9	-17,1
SA11	0	8,67E-03	72,65	10,639	361,3	-1,3
SA11	100	6,91E-03	69,829	10,926	358,8	-2,6
SA11	150	5,26E-03	72,718	9,7416	1,8	-2
SA11	200	5,28E-03	67,422	9,3745	357,7	-5,2
SA11	250	3,37E-03	65,298	11,469	354,8	-4,6
SA11	300	3,38E-03	64,514	10,401	354,7	-5,9
SA11	350	1,76E-03	64,818	21,85	348,8	4
SA11	400	1,24E-03	58,168	16,892	346	-3,5
SA11	450	7,60E-04	359,53	-15,499	290,8	-51,5
SA11	475	9,03E-04	35,07	21,374	324,3	-8,8
SA11	500	2,56E-04	258,34	-29,512	174,6	-17,2
SA11	525	6,48E-04	22,907	5,3157	317,4	-27,7
SA11	550	3,32E-04	307,26	11,465	238,9	-10,8
SA11	575	1,49E-03	342,57	45,133	279,1	10,2
SA11	600	1,80E-04	320,04	12,548	251	-15,3
SA11	625	3,29E-04	165,66	32,127	80,7	65,9
SA11	650	4,65E-04	211,59	32,34	170,3	58,8
SA11	675	1,50E-05	204,66	43,962	179,5	71,1

Table S2. Thermal demagnetization data.

(1) Sample = sample name
(2) Temperature (°C)
(3) NRM intensity (A/m)
(4) Core Declination (°E)
(5) Core Inclination (°)
(6) Geographic Declination (°E)
(7) Geographic Inclination (°)

SA12	0	5,88E-03	63,865	35,892	352,3	-4,3
SA12	100	4,84E-03	59,775	34,361	351,6	-7,9
SA12	150	3,93E-03	55,283	34,23	349,3	-10,9
SA12	200	3,24E-03	53,216	34,406	348	-12,1
SA12	250	2,40E-03	34,29	47,553	328,3	-11,2
SA12	300	1,88E-03	53,307	73,989	318,7	14,9
SA12	350	1,87E-03	29,252	52,97	322,8	-8
SA12	400	3,35E-03	315,95	27,198	263,4	-22,7
SA12	425	9,09E-04	314,03	38,427	270,1	-13,3
SA12	450	8,45E-04	330,78	42,083	283,1	-17,7
SA12	475	7,04E-04	296,68	52,153	272,1	4,8
SA12	500	5,53E-04	317,09	39,569	272,8	-14
SA12	525	8,88E-04	308,37	32,708	262,6	-14,2
SA12	550	9,07E-04	281,13	50,648	265,9	12,5
SA12	575	9,63E-04	329,46	15,409	265,9	-39,8
SA12	600	8,96E-04	320,43	26,877	266,4	-25,6
SA12	625	1,04E-03	302,54	12,781	242,7	-22,5
SA12	650	7,33E-04	260,33	7,6843	218,5	12
SA13	0	7,64E-03	65,029	27,936	345,5	-8
SA13	100	6,26E-03	61,431	28,612	343,1	-10,3
SA13	150	5,02E-03	65,501	30,137	343,9	-6,5
SA13	200	4,68E-03	65,591	30,284	343,8	-6,3
SA13	250	3,56E-03	61,239	31,645	340,5	-8,6
SA13	300	3,64E-03	57,857	32,051	338,4	-10,6
SA13	350	2,09E-03	30,137	47,384	311,9	-12,7
SA13	400	2,11E-03	38,911	46,324	317,7	-10,4
SA13	450	1,64E-03	12,653	58,734	298,1	-5,6
SA13	475	1,24E-03	12,661	46,683	300,6	-17,4
SA13	500	6,88E-04	340,14	53,802	279,8	-9,3
SA13	525	1,65E-03	332,98	56,545	276,9	-5,3
SA13	550	1,19E-03	27,728	68,315	301,4	5,5
SA13	575	1,64E-03	310,03	59,478	268,6	3,9
SA13	600	1,26E-03	332,11	-6,7388	231,2	-57,7
SA13	625	5,75E-04	226,6	45,308	242,2	47,6
SA13	650	4,93E-04	165,22	44,286	319,7	67,3
SA13	675	3,53E-04	254,39	-1,7847	193,1	13,3
SA14	0	4,98E-03	68,288	17,28	6,3	-8,6
SA14	100	4,09E-03	71,518	18,39	7,2	-5,5
SA14	150	4,05E-03	68,973	18,408	5,8	-7,4
SA14	200	2,61E-03	65,273	15,582	5,9	-11,9
SA14	250	1,56E-03	66,477	14,959	7,1	-11,4
SA14	300	1,55E-03	59,823	14,915	3,1	-16,5
SA14	350	1,48E-03	61,115	11,447	6,7	-17,7
SA14	400	1,56E-03	65,054	13,255	7,7	-13,5
SA14	425	7,31E-04	53,288	-6,3662	17,7	-34,5
SA14	450	8,51E-04	48,048	-0,20195	7,8	-35,1
SA14	475	3,91E-04	352,76	-24,016	258,2	-80,5
SA14	500	2,16E-04	154,28	11,206	66,6	59,1
SA14	525	2,77E-04	279	-18,943	200,3	-17,1
SA14	550	2,86E-04	16,469	-2,0045	333,9	-57,1
SA15	0	4,69E-03	69,631	23,345	1,5	-6,5
SA15	100	3,92E-03	59,903	22,32	357,3	-14,5
SA15	150	2,97E-03	55,125	18,827	357,3	-20,2
SA15	200	3,15E-03	53,092	16,8	357,7	-23
SA15	250	2,40E-03	48,847	19,447	352,7	-24,3
SA15	300	1,67E-03	40,847	17,571	348,2	-31,1
SA15	350	1,67E-03	44,053	18,017	350,4	-28,6
SA15	400	1,50E-03	36,454	20,436	342,3	-31,6
SA15	450	6,83E-04	8,676	2,3482	319,3	-60,4
SA15	475	6,35E-04	344,17	3,6998	271,9	-56,6
SA15	500	1,20E-03	330,43	23,067	268,6	-33,2
SA15	525	6,96E-04	299,88	24,27	247,2	-13,2
SA15	550	6,38E-04	293,66	27,627	246,7	-6,7
SA15	575	6,42E-04	305,2	27,561	253	-14,9
SA15	600	4,23E-04	315,3	16,498	250,9	-29,2
SA15	625	7,96E-04	298,34	10,792	234,8	-19,7
SA15	650	3,65E-04	315,56	37,442	266,5	-14,1
SA15	675	3,60E-04	316,23	34,674	265,1	-16,5
SA16	0	5,32E-03	63,71	29,112	359	9
SA16	100	3,87E-03	62,069	22,954	361,1	3
SA16	150	3,45E-03	61,712	25,207	359,6	4,7
SA16	200	2,89E-03	76,128	28,476	8,4	14,7
SA16	250	4,16E-03	14,036	-66,648	105,1	-74,7
SA16	300	4,57E-03	289,92	43,964	258,7	24
SA16	350	1,31E-03	26,91	42,281	326,3	8,1
SA16	400	4,88E-03	342,79	4,2296	286,4	-31
SA16	425	8,55E-04	317,02	10,035	261,9	-17,1
SA16	450	1,14E-03	301,46	11,89	249	-8,2
SA16	475	8,69E-04	299,91	9,0687	246,2	-9,8
SA16	500	5,96E-04	334,21	-5,8726	273	-38,4
SA16	525	8,29E-04	335,42	4,4255	151,2	-28,9
SA16	550	8,20E-04	277,22	-1,3282	221,5	-5,4
SA16	575	5,97E-04	265,65	21,941	226,8	19,9
SA16	600	5,97E-04	313,08	23,402	264,3	-3,4
SA16	625	3,03E-04	263,47	41,054	240,1	35,2
SA16	650	1,39E-04	208,99	-52,437	144,6	-18,2
**Oulad Hamida**						
oh220	0	2,87E-05	193,81	-60,55	327,4	18,6
oh220	100	5,94E-06	153,34	-31,59	289,8	41,2
oh220	150	1,41E-05	60,25	15,5	207,8	-25,1
oh220	200	1,68E-05	54,28	26,14	194,5	-26,1
oh220	225	1,48E-05	58,5	29,4	193,2	-21,3
oh220	250	1,51E-05	64,27	35,86	189,1	-14,2
oh220	275	9,54E-06	70	48,32	179,2	-5,4
oh220	300	1,31E-05	66,87	58,07	169,4	-3,3
oh220	325	9,00E-06	59,57	66,58	160,3	-2,2
oh220	350	1,16E-05	261,81	79,11	129,3	11,4
oh220	375	4,69E-06	258,33	40,26	89,5	15,3
oh220	400	5,97E-05	63,42	-37,27	267,2	-27,1
oh220	425	8,80E-06	291,66	-7,3	46,5	-22,5
oh220	450	9,80E-06	319,56	-31,92	13,7	-46,7
oh220	475	1,55E-05	14,74	-19,79	266,5	-72,7
oh220	500	1,10E-05	327,91	-16,22	35,6	-58,2
oh220	525	1,46E-05	328,72	-34,03	5,4	-52,6
oh220	550	2,47E-05	1,07	8,05	143,7	-71,9
oh220	575	2,82E-05	8,76	26,73	153,1	-52,3
oh220	600	2,86E-05	345,94	11,32	106,7	-64,5
oh230	25	3,88E-05	227,48	-14,86	14,9	40,1
oh230	100	2,87E-05	250,75	-5,74	29,5	18,9
oh230	150	2,17E-05	264,98	6,46	42,6	5,2
oh230	200	1,96E-05	273,83	9,07	45,5	-3,5
oh230	225	1,72E-05	271,78	15,61	51,9	-1,2
oh230	250	1,57E-05	267,94	4,8	41	2,2
oh230	275	2,13E-05	273,91	2,89	39,3	-3,8
oh230	300	1,84E-05	276,09	-1,13	35,4	-6,1
oh230	325	1,96E-05	268,13	-18,56	17,7	1,1
oh230	350	1,93E-05	259,3	-28,38	7,2	8,4
oh230	375	1,60E-05	258,95	-43,02	352,5	6,7
oh230	400	1,76E-05	264,1	-46,65	349,4	2,6
oh230	425	1,66E-05	253,92	-48,07	346,9	9,1
oh230	450	1,86E-05	259,06	-26,67	8,9	8,8
oh230	475	1,73E-05	249,22	-21,85	12,4	18,4
oh230	500	2,10E-05	263,02	-22,21	13,7	5,7
oh230	525	2,53E-05	270,31	-9,84	26,5	-0,6
oh230	550	9,41E-06	240,56	-26,83	5,3	25
oh230	575	1,29E-05	241,63	-19,91	13	25,8
oh230	600	9,13E-06	230,76	-71,44	320,8	9,7
oh230	625	2,26E-05	292,69	-20,24	15,2	-21,9
oh230	650	1,69E-05	244,19	-42,45	350,4	17,3
oh230	675	1,93E-05	3,09	-27,02	299,8	-64,8
oh237	0	4,07E-05	197,46	-49,24	320,9	33,7
oh237	100	2,66E-05	151,59	-60,72	292,9	20,6
oh237	150	2,24E-05	106,86	-61,16	279,7	3,6
oh237	200	2,15E-05	82,24	-67,1	284,4	-7,6
oh237	225	2,29E-05	82,44	-67,56	284,8	-7,5
oh237	250	2,22E-05	66,71	-72,35	290,8	-11,7
oh237	275	2,78E-05	90,12	-76,49	293,7	-4,8
oh237	300	3,00E-05	91,32	-76,16	293,4	-4,5
oh237	325	2,99E-05	110,9	-72,06	290,6	1,5
oh237	350	3,41E-05	127,85	-66,31	288,5	9,5
oh237	375	3,48E-05	121,22	-63,34	284,4	8,8
oh237	400	3,74E-05	126,36	-65,63	287,6	9,5
oh237	425	3,30E-05	130,17	-64,77	287,9	11,2
oh237	450	3,24E-05	139,24	-55,89	284,3	20,5
oh237	475	2,51E-05	106,74	-82,52	300,1	-2,8
oh237	500	2,80E-05	122,32	-74,24	294	3,5
oh237	525	2,96E-05	101,18	-75,34	292,9	-2
oh237	550	2,62E-05	23,89	-50,46	287,5	-40,3
oh237	575	3,94E-05	30,28	-50,53	283,3	-37,9
oh237	600	2,69E-05	226,42	-55,11	333,2	18,7
oh237	625	2,91E-05	325,36	-37,62	346,5	-44,6
oh237	650	1,39E-05	297,82	21,48	63,4	-23,6
oh237	675	2,35E-05	274,09	-18,18	19,4	-5,4
oh249	0	4,44E-05	219,13	4,87	9,6	46,7
oh249	100	2,21E-05	214,67	10,6	13,3	53,5
oh249	150	1,36E-05	214,39	19,59	27,5	57,7
oh249	200	1,40E-05	215,21	34,86	56,7	59
oh249	225	1,18E-05	217,96	41,9	69	55,7
oh249	250	1,31E-05	221,75	51,8	83,3	50,2
oh249	275	1,29E-05	316,78	81,14	116,8	20,4
oh249	300	1,76E-05	13,3	68,9	128,1	6,4
oh249	325	1,78E-05	326,18	58,14	106,2	-0,3
oh249	350	2,63E-05	341,3	62,04	114,6	0,3
oh249	375	2,20E-05	291,55	58,52	93,5	12,5
oh249	400	2,39E-05	259,17	35,88	62,9	23,7
oh249	425	2,41E-05	283,22	36,94	71,8	6,3
oh249	450	2,07E-05	268,65	23,7	54,1	11,6
oh249	475	2,30E-05	265,69	-6,15	25,8	1
oh249	500	1,37E-05	327,11	15,25	82,3	-37
oh249	525	1,44E-05	272,74	7,54	41,2	1
oh249	550	3,28E-05	294,78	14,69	58,3	-14,2
oh249	575	3,78E-05	322,66	11,64	75,2	-37
oh249	600	3,50E-05	200,67	0,97	344	57,3
oh279	0	2,73E-05	219,69	-61,06	307,9	28,4
oh279	100	1,19E-05	269,87	-88,39	288,9	7
oh279	150	1,16E-05	35,84	-49,8	262,6	-25,2
oh279	200	1,05E-05	40,15	-41,68	253,9	-29,1
oh279	225	1,04E-05	49,11	-26,25	234,3	-31,9
oh279	250	1,17E-05	46,52	-29,83	239,3	-32,1
oh279	275	9,34E-06	42,91	-37,23	248,4	-30,3
oh279	300	2,28E-05	17,26	-37,36	268,5	-42,8
oh279	325	1,84E-05	15,86	-41,21	271,8	-39,6
oh279	350	2,21E-05	342,42	-37,72	306,2	-42,4
oh279	375	1,75E-05	344,73	4,22	7,7	-74,6
oh279	400	2,70E-05	8,61	-14,14	265,3	-67,2
oh279	425	2,00E-05	315,42	-18,9	346	-39
oh279	450	2,48E-05	341,52	-13,15	329,7	-62,7
oh279	475	2,12E-05	331,7	-21,3	331,1	-50,3
oh279	500	3,09E-05	332,29	-20,06	332	-51,6
oh279	525	7,25E-06	284,3	-42,48	333,2	-5,7
oh279	550	9,49E-06	305,18	-16,42	353,4	-30,9
oh279	575	3,23E-05	338,43	-14,86	331,6	-59,4
oh279	600	1,12E-05	202,44	-0,23	33,2	66,6
oh298	0	1,70E-05	239,2	-23,99	336,7	23
oh298	100	1,17E-05	235,46	-45,33	315,2	15,6
oh298	150	8,82E-06	249,34	-47,15	318,1	6,3
oh298	200	8,90E-06	256,53	-62,25	305,2	-2,7
oh298	225	7,62E-06	270,44	-48,29	320,5	-7,7
oh298	250	6,29E-06	265,48	-40,44	327,7	-3,1
oh298	275	5,87E-06	267,89	-15,33	352,8	-0,6
oh298	300	3,80E-06	237,39	13,56	18	33,8
oh298	325	4,32E-06	232,92	-15,58	342,9	31,7
oh298	350	4,78E-06	257,5	-20,46	345,8	8
oh298	375	6,42E-06	244,56	-6,42	356,7	23,6
oh298	400	6,55E-06	239,64	-5,46	356,4	28,6
oh298	425	4,54E-06	231,93	-21,1	336,5	30,3
oh298	450	5,78E-06	207,04	-33,25	307,9	39,7
oh298	475	6,71E-06	230,58	-25,8	331,1	29,2
oh298	500	9,49E-06	260,15	-36,29	330,9	1,9
oh298	525	1,32E-05	212,87	-22,89	322,3	44
oh298	550	1,72E-05	235,03	-19,56	339,5	28,3

Table S2. Thermal demagnetization data.

(1) Sample = sample name
(2) Temperature (°C)
(3) NRM intensity (A/m)
(4) Core Declination (°E)
(5) Core Inclination (°)
(6) Geographic Declination (°E)
(7) Geographic Inclination (°)

oh298	575	1,70E-05	101,26	-43,37	232,8	1,2
oh298	600	1,94E-05	172,52	4,54	224,1	80,8
oh305	0	3,61E-05	239,81	27,05	25	38,2
oh305	100	3,16E-05	238,5	61,03	69,6	41,9
oh305	150	3,30E-05	276,13	80,2	92,1	29,5
oh305	200	3,42E-05	321,06	78,91	95,8	22,2
oh305	225	3,79E-05	314,56	77,3	93,6	21,7
oh305	250	4,00E-05	321,45	77,35	94,9	20,8
oh305	275	3,80E-05	336,75	77,64	98,1	19,5
oh305	300	3,37E-05	333,75	67,68	93,4	10,6
oh305	325	3,04E-05	315,05	70,72	89,2	16,6
oh305	350	2,98E-05	318,12	73,81	92	18,5
oh305	375	2,89E-05	315,11	58,35	81,4	6,9
oh305	400	2,94E-05	314,36	56,98	80,2	6
oh305	425	2,85E-05	334,56	58,21	90,2	1,7
oh305	450	2,91E-05	331,54	62,19	90,4	6
oh305	475	2,55E-05	334,23	52,31	87,8	-3,7
oh305	500	1,69E-05	338,33	54,6	90,9	-2,4
oh305	525	2,69E-05	324,76	43,44	78,2	-8,9
oh305	550	1,38E-05	336,25	66,91	94,1	9,6
oh305	575	1,69E-05	237,09	62,63	71,9	42,2
oh305	600	1,27E-05	311,77	70,12	87,9	16,9
oh305	625	2,65E-05	292,6	41,09	59	5,2
oh305	650	9,86E-06	352,21	-14,28	78,4	-71,8
oh305	675	1,11E-05	357,04	72,07	102,3	13,1
oh325	0	1,83E-04	211,33	-29,06	333,1	23,8
oh325	100	1,58E-04	211,96	-34,83	330,6	18,5
oh325	150	1,29E-04	218,12	-35,2	334,9	15,6
oh325	200	1,20E-04	208,81	-34,94	328,1	19,6
oh325	225	1,09E-04	215,93	-31,18	335,6	19,9
oh325	250	1,14E-04	210,98	-31,19	331,7	22,1
oh325	275	1,02E-04	209,6	-30,22	331	23,5
oh325	300	9,27E-05	214,6	-25,36	337,9	25,5
oh325	325	8,70E-05	206,42	-21,5	332,7	32,6
oh325	350	9,40E-05	196,2	-18,66	323,1	38,9
oh325	375	9,94E-05	214,35	-14,83	344,6	34,3
oh325	400	9,27E-05	213,16	-20,08	339,9	30,6
oh325	425	9,37E-05	206,79	-19,02	334,5	34,6
oh325	450	9,73E-05	224,59	-19,64	349,8	24,4
oh325	475	1,05E-04	229,7	-14,87	357,3	24,4
oh325	500	9,26E-05	237,04	-15,32	2	18,8
oh325	525	9,40E-05	229,04	-28,79	346,5	14,9
oh325	550	1,02E-04	254,15	-16,56	11	4,8
oh325	575	9,02E-05	258,14	-32,02	359,9	-6,6
oh325	600	1,17E-04	247,46	-24,16	1,1	5,6
oh325	625	9,42E-05	249,81	-35,21	353,4	-2,5
oh325	650	1,04E-04	246	-24,77	359,9	6,3
oh325	675	1,05E-04	260,25	-18,09	12,8	-0,9
oh345	0	1,43E-04	178,28	-25,2	296	47,8
oh345	100	1,35E-04	176,85	-24,07	293,9	48,8
oh345	150	1,21E-04	172,69	-23,6	288,1	48,8
oh345	200	1,16E-04	172,73	-23,07	288	49,3
oh345	225	1,13E-04	172,37	-23,83	287,7	48,5
oh345	250	1,16E-04	181,21	-19,07	300,2	53,9
oh345	275	1,10E-04	183,16	-14,35	304,1	58,5
oh345	300	1,11E-04	174,19	0,24	278,8	72,3
oh345	325	1,07E-04	172,86	-12,9	284,6	59,3
oh345	350	1,05E-04	180,36	-26,01	298,8	47
oh345	375	1,07E-04	174,55	-13,25	287,9	59,3
oh345	400	1,10E-04	175,25	-16,6	290,1	56,1
oh345	425	1,07E-04	176,65	-3,09	288,6	69,6
oh345	450	9,59E-05	176,28	-3,54	287,8	69,1
oh345	475	1,00E-04	177,59	-10,62	293,2	62,3
oh345	500	9,86E-05	177,74	3,28	288,9	76,1
oh345	525	8,17E-05	180,04	11,55	298,7	84,5
oh345	550	7,68E-05	192,18	-2,29	331,4	67,3
oh345	575	8,33E-05	185,58	11,24	342,2	82,1
oh345	600	8,53E-05	172,19	2,47	269,6	73,6
oh345	625	8,36E-05	175,13	23,93	150,8	81,7
oh345	650	8,53E-05	171,99	5,35	263,4	76
oh345	675	8,81E-05	183,17	16,49	19,2	86,9

Table S3. Magnetostratigraphic data.

Sample = sample name.
cm = elevation of sample from section base in cm.
NRM = natural remanent magnetization of sample at room temperature in mA/m (10E-3 A/m) and 10cc volume.
SUS = volume magnetic susceptibility in 10E-6 SI.
A_LT = low unblocking temperature in °C of magnetic component A.
A_HT = high unblocking temperature in °C of magnetic component A.
A_Dec = mean declination in °E of magnetic component A.
A_Inc = mean inclination in ° of magnetic component A.
C_LT = low unblocking temperature in °C of characteristic magnetic component C.
C_HT = high unblocking temperature in °C of characteristic magnetic component C.
C_MAD = maximum angular deviation of characteristic magnetic component C.
C_Dec = mean declination in °E of characteristic magnetic component C.
C_Inc = mean inclination in ° of characteristic magnetic component C.
C_VGP = virtual geomagnetic pole latitude in ° of characteristic magnetic component C.

Sample	cm	NRM	SUS	A_LT	A_HT	A_Dec	A_Inc	C_LT	C_HT	C_MAD	C_Dec	C_Inc	C_VGP
**OH1 Bed1 (below trench base):**													
S-30	-30.0	0.066	10.1										
S-41	-41.0	0.300	9.9					100	425	9.2	246.3	-9.0	-22.2
S-62	-62.0	0.311	8.6	0	100	352.9	7.1	100	450	26.5	171.4	-33.5	-72.9
S-77	-77.0	0.252	11.5	0	100	353.4	36.0	100	350	7.0	227.1	-53.8	-51.3
S-91	-91.0	0.159	10.8										
S-109	-109.0	0.335	17.7										
S-134	-134.0	0.218	13.6	0	100	27.1	22.8						
S-180	-180.0	0.376	13.3	0	100	314.4	14.6	100	400	31.1	195.8	-21.5	-63.3
**OH1 Bed2 Trench South Wall:**													
s004	4.0	0.172	153.3										
s010	10.0	0.276	132.9					350	575	11.3	176.1	-18.5	-65.6
s014	14.0	0.274	157.1										
s040	40.0	0.272	180.3					275	475	10.3	156.0	-21.0	-58.4
s043	43.0	0.333	185.7										
s050	50.0	0.375	171.9										
s054	54.0	0.134	170.7					150	425	12.7	150.8	-31.1	-58.9
s058	58.0	0.157	160.3					150	500	20.1	187.4	-27.0	-69.6
s061	61.0	0.101	104.1					100	475	17.1	162.7	-33.5	-68.3
s110	110.0	0.230	132.1	0	100	45.0	13.7	150	650	7.5	181.4	1.4	-55.7
s113	113.0	0.140	94.6					250	500	11.6	153.6	10.2	-43.9
s117	117.0	0.244	110.8					150	600	17.5	177.1	14.4	-49.0
s124	124.0	0.123	83.8					150	425	9.9	169.3	12.4	-48.9
s131	131.0	0.157	89.3					225	400	13.6	143.9	-21.0	-49.9
s135	135.0	0.108	87.3	0	100	320.0	67.0	225	325	12.2	173.0	-2.0	-56.8
s140	140.0	0.077	73.6					150	400	14.8	150.8	13.9	-40.8
s146	146.0	0.120	65.4					225	600	18.3	156.2	8.7	-45.9
s152	152.0	0.197	104.9					200	575	16.3	164.6	-8.1	-57.2
s160	160.0	0.183	97.4					150	425	13.6	150.2	4.7	-44.4
s164	164.0	0.117	95.0					150	350	13.2	161.6	3.9	-50.5
s171	171.0	0.146	104.4					150	400	11.9	151.0	8.5	-43.3
s176	176.0	0.176	110.8					225	400	22.9	165.4	-20.5	-63.4
s181	181.0	0.396	111.7					250	500	10.0	181.1	5.6	-53.6
s187	187.0	0.370	128.8					200	525	5.1	156.6	1.9	-49.0
s204	204.0	0.588	181.3					225	400	7.1	159.2	12.7	-45.4
s215	215.0	0.535	182.9					250	400	14.4	167.0	0.9	-53.8
s221	221.0	0.765	214.3					200	425	6.1	171.0	-1.3	-56.0
s230	230.0	0.758	226.4	0	100	33.9	38.3	275	425	7.9	172.5	1.1	-55.1
s234	234.0	0.840	180.4					200	350	5.7	169.2	7.3	-51.4
s240	240.0	1.098	220.1					225	475	7.6	160.7	4.6	-49.8
s253	253.0	1.434	323.9	0	100	336.5	7.5	225	550	4.3	162.2	3.4	-50.9
s268	268.0	2.739	447.2					275	525	2.9	160.9	4.6	-49.8
s273	273.0	2.255	418.5					275	600	5.0	160.2	-0.9	-52.0
s281	281.0	2.658	488.2	0	100	317.4	51.3	250	575	2.2	159.7	6.7	-48.4
s287	287.0	3.634	526.2					275	625	5.3	156.8	-0.8	-50.3
s299	299.0	3.936	549.6					250	650	2.3	159.6	4.6	-49.3
s314	314.0	6.043	669.3					200	550	3.0	160.8	2.5	-50.8
**OH1 Bed2 Trench North Wall:**													
n007	7.0	0.086	108.1					150	525	22.3	163.7	-1.8	-53.9
n011	11.0	0.021	89.0	0	150	8.6	71.2						
n015	15.0	0.100	103.8					150	300	20.2	214.3	-3.9	-45.0
n019	19.0	0.114	109.0	0	100	6.7	14.8						
n023	23.0	0.050	140.6	0	150	39.5	68.9						
n069	69.0	0.250	191.8					250	475	25.3	139.6	-25.2	-48.1
n072	72.0	0.139	183.8					250	450	11.4	134.8	-18.4	-42.1
n074	74.0	0.117	192.9					225	350	17.6	198.6	-39.6	-70.2
n077	77.0	0.159	190.2	0	100	308.8	29.3	225	525	18.9	161.4	-27.7	-64.7
n082	82.0	0.155	188.7	0	100	38.2	0.6	300	400	20.1	148.3	-21.2	-53.2
**OH2:**													
s333	333.0	0.798	164.3					200	525	7.5	180.4	-1.3	-57.1
OH2 Hand Sample 03.19 (oh2_3)													
	347.0	10.980	144.4					100	550	5.6	171.1	-6.7	-58.6
OH2 Hand Sample 04.19 (oh2_4)													
	351.0	5.885	168.8	0	100	339.2	51.5	150	475	7.6	346.2	15.4	61.3
S352	352.0	10.830	186.0					100	675	7.2	183.5	8.1	-52.2
S372	372.0	6.896	203.9	0	100	49.0	17.1	200	650	18.6	174.2	-34.5	-74.5
OH2 Hand Sample 02.19 (oh2_2)													
	376.0	19.660	168.1					150	650	10.2	164.8	3.7	-51.8
OH2 Hand Sample 05.19 (oh2_5)													
	383.0	4.418	161.0					150	425	16.7	205.6	2.3	-47.7

S386	386.0	7.428	194.4					150	675	12.8	155.9	-24.0	-59.6
OH2 Hand Sample 01.19 (oh2_1)													
	391.0	11.370	131.7					100	500	8.2	162.7	11.2	-47.5
OH2 Hand Sample 06.19 (oh2_6)													
	401.0	0.319	19.3										
S409	409.0	3.177	218.1					100	600	26.7	165.4	16.7	-45.7
S436	436.0	2.855	249.7					100	650	12.5	170.4	-47.9	-80.6
S453	453.0	1.544	91.5	0	150	60.3	20.5	200	425	20.2	130.0	-16.4	-37.6
S471	471.0	0.773	96.2										
S486	486.0	1.093	115.3										
S510	510.0	0.128	48.0										
S520	520.0	1.324	91.8										
S548	548.0	1.354	94.3	0	150	35.7	43.2	150	450	23.2	171.2	20.0	-45.3
S565	565.0	0.405	48.1	0	100	52.2	62.7	100	350	19.4	231.6	-12.6	-35.1
S598	598.0	5.212	137.1					100	400	8.3	196.5	2.0	-52.1
S605	605.0	0.957	96.0	0	200	25.5	25.0						
S625	625.0	1.181	102.0	0	200	1.4	35.7						
S663	663.0	0.770	60.0	0	100	41.0	53.5	150	300	12.9	169.8	5.6	-52.4
S709	709.0	1.067	80.1	0	150	316.9	36.0						
S762	762.0	0.783	36.9										
S810	810.0	0.580	43.1	0	150	329.8	10.3						
S863	863.0	0.615	48.1										
S939	939.0	0.738	81.5	0	200	351.9	64.9						
S987	987.0	1.077	107.9										
S1044	1044.0	1.101	106.8	0	100	5.0	58.9						
S1099	1099.0	0.798	125.3	0	100	30.8	82.7	200	525	24.4	172.8	-11.7	-61.5
S1171	1171.0	0.432	25.2										
S1238	1238.0	0.272	12.2	0	200	334.1	34.4						
S1281	1281.0	0.425	13.8	0	150	22.9	61.9	200	350	17.9	183.0	-36.6	-76.5
S1309	1309.0	0.298	29.0	100	200	35.2	49.4						
S1353	1353.0	0.184	22.0										
S1375	1375.0	0.640	24.7	0	100	14.1	72.6	100	450	19.0	322.7	23.5	49.9
S1436	1436.0	0.634	30.3										
S1525	1525.0	1.367	81.1	0	100	43.5	62.2						
S1569	1569.0	3.591	264.5	0	150	354.9	54.4						
S1589	1589.0	0.868	43.4	0	100	332.4	13.0						
S1599	1599.0	11.770	435.6	0	150	349.6	33.8						
S1634	1634.0	20.090	790.1	0	150	347.4	29.9						
S1701	1701.0	18.040	714.0	0	150	19.9	77.2						
**OH3-B:**													
3BOH30	30.0	1.218	36.8	0	200	327.8	32.5						
3BOH57	57.0	0.930	28.3	0	250	339.0	29.2						
3BOH94	94.0	1.016	46.2	0	300	320.2	45.7						
3BOH136	136.0	1.666	55.2	0	300	347.1	42.2						
3BOH178	178.0	1.325	49.3	0	250	325.5	26.1						
3BOH221	221.0	1.519	51.2	0	200	341.7	36.5						
3BOH281	281.0	0.826	29.0										
3BOH308	308.0	1.019	35.4	0	150	16.6	31.3	150	425	24.0	210.6	-3.4	-47.1
3BOH388	388.0	0.999	41.4	0	150	4.9	28.7	350	650	16.5	198.1	36.9	-33.1
3BOH428	428.0	1.055	55.5	0	250	330.4	54.6						
3BOH463	463.0	1.765	58.6	100	425	31.1	44.0						
3BOH486	486.0	0.510	35.2	100	200	29.8	44.7						
3BOH510	510.0	1.326	42.5	0	200	10.5	24.7						
**OH3-A:**													
3OH28	28.0	0.600	27.1	0	150	284.7	23.4						
3OH51	51.0	0.439	29.3										
3OH94	94.0	0.862	37.9	0	150	9.8	18.2						
3OH121	121.0	3.505	294.8	0	150	27.8	27.1	300	625	34.4	166.7	-21.0	-64.2
3OH144	144.0	0.328	27.3	0	150	327.0	24.1						
3OH172	172.0	0.563	36.6										
3OH212	212.0	0.427	23.0	0	150	342.7	27.1						
3OH229	229.0	1.156	50.4	0	150	339.3	27.7						
3OH246	246.0	1.272	38.5	0	100	284.8	51.4	350	500	10.9	180.5	8.6	-52.1
3OH313	313.0	0.943	35.0	0	100	320.8	33.9	150	300	14.4	150.1	5.2	-44.1
3OH339	339.0	1.251	37.9	0	300	18.1	43.5						
3OH379	379.0	0.748	46.5	0	200	333.0	29.9						
3OH405	405.0	7.364	42.5	0	200	23.3	45.6	250	475	3.2	34.9	41.6	58.1
3OH462	462.0	4.294	161.7	0	100	10.6	1.4	150	625	31.9	1.9	7.4	60.1
3OH490	490.0	6.235	53.1	0	100	338.3	16.2	150	500	8.8	312.6	28.6	43.6
**OH4:**													
4OH8	8.0	40.350	8380.1	0	150	358.5	25.1	250	675	8.2	178.6	-33.9	-74.9
4OH29	29.0	15.670	2124.6	0	200	0.8	25.5	200	625	3.1	183.0	-48.6	-85.2
4OH59	59.0	25.740	3319.5	0	150	6.4	37.8	300	675	6.9	194.6	-21.5	-63.9
OH4 Hand Sample 07.19 (oh4_7)													
	91.0	12.580	4049.4	0	250	351.8	22.2	275	675	6.8	182.7	-30.1	-72.4
4OH96	96.0	12.340	2312.6	0	250	10.1	25.1	300	650	12.5	188.7	-18.4	-64.6
4OH116	116.0	3.794	593.8	0	150	10.0	25.5	250	675	8.5	216.8	-43.2	-57.0
4OH145	145.0	1.666	683.8	0	150	25.2	37.8	300	675	15.5	184.3	-13.4	-62.9
OH4 Hand Sample 08.19 (oh4_8)													
	148.0	8.638	1942.8	0	200	3.7	23.2	275	650	5.3	181.2	-30.0	-72.5
4OH166	166.0	3.313	1103.2	0	200	356.9	29.2	350	675	6.2	189.5	-21.0	-65.7
4OH198	198.0	4.659	723.7	0	200	347.5	44.2	250	625	6.7	199.2	-18.7	-60.2
OH4 Hand Sample 09.19 (oh4_9)													
	204.0	5.979	1097.0	0	200	353.8	42.1	250	675	9.3	178.9	-8.3	-60.6
4OH237	237.0	4.817	648.6	0	250	6.6	21.9	250	675	7.2	219.4	-46.8	-55.9
4OH263	263.0	5.317	606.9	0	300	11.3	27.2	450	650	11.7	176.4	-2.9	-57.7
4OH303	303.0	3.231	401.3	0	150	13.9	38.1	550	675	20.0	205.7	-47.5	-67.5
4OH363	363.0	31.040	3156.3	0	150	348.7	31.7						

4OH403	403.0	9.800	1386.8	0	150	25.6	46.7	250	625	11.3	248.0	-21.0	-24.3
4OH433	433.0	7.275	1324.3	0	200	345.1	39.9	200	675	24.0	174.3	-35.9	-75.4
4OH443	443.0	19.210	1682.5					200	600	16.6	202.2	-58.7	-71.3
4OH454	454.0	25.780	1610.0	0	150	331.9	50.5	425	625	21.6	227.6	-9.5	-37.3
4OH468	468.0	13.260	937.2	0	250	342.4	30.2						
4OH483	483.0	5.411	636.0	0	250	2.3	31.0	350	575	32.2	172.4	24.7	-42.9
4OH484	484.0	6.043	573.0	0	150	320.4	28.8	200	675	12.2	173.6	-56.6	-83.7
4OH508	508.0	5.704	631.7	0	250	336.6	29.0	450	650	3.1	199.5	-16.0	-58.8
4OH528	528.0	16.760	836.7	0	150	316.5	53.3	250	625	9.0	8.5	16.2	63.5
4OH548	548.0	22.490	1139.0	0	100	331.6	45.5	100	500	20.9	300.4	35.7	35.7
4OH578	578.0	25.680	1175.0	0	150	322.6	7.8	200	550	31.9	345.3	13.9	60.2

OH5:

5OH130	130.0	17.860	390.7	0	150	3.2	33.9	200	675	19.3	344.4	15.0	60.3
5OH181	181.0	14.570	371.8	0	200	317.5	34.5	250	550	20.0	320.7	29.4	50.4
5OH215	215.0	9.466	270.5										
5OH244	244.0	5.479	160.7	0	200	311.7	29.1	250	525	28.3	351.3	43.2	78.7
5OH266	266.0	10.420	272.8	0	150	327.7	23.5	200	500	7.8	315.6	13.2	41.0
5OH291	291.0	9.466	396.6	0	200	328.0	8.0	200	625	17.5	321.4	17.8	46.9
5OH326	326.0	13.970	423.8	0	150	313.8	27.9	200	500	10.9	310.4	45.5	47.1
5OH351	351.0	19.640	566.0	0	150	312.3	27.9	350	525	6.5	335.5	11.8	54.2
5OH377	377.0	11.910	370.9	0	150	321.3	32.2	200	525	9.5	318.0	9.3	41.5

Sidi Abderrhamane:

SA1	177.0	0.553	11.8	0	100	340.5	48.9						
SA2	206.0	0.906	35.6	0	100	42.6	14.3						
SA3	226.0	0.703	21.5										
SA4	276.0	2.231	45.8	0	100	356.8	15.3						
SA5	335.0	0.743	23.5	0	100	349.2	23.5						
SA6	361.0	1.829	38.4	0	100	327.1	36.1	100	350	12.6	336.0	41.7	67.0
SA7	419.0	0.148	17.9										
SA8	434.0	0.733	25.6										
SA9	459.0	0.192	17.1										
SA10	485.0	1.024	22.9	0	100	347.5	62.0	100	450	16.5	321.9	59.2	59.1
SA11	290.0	8.670	35.7					0	450	5.1	2.3	-0.1	56.3
SA12	304.0	5.882	21.1					0	425	7.0	2.1	-3.9	54.4
SA13	290.0	7.645	30.0					0	450	6.2	355.0	-7.1	52.5
SA14	301.0	4.978	15.9					100	475	3.8	4.8	-0.9	55.7
SA15	303.0	4.697	14.3					100	500	9.7	5.2	-7.1	52.5
SA16	305.0	5.326	20.5										

Rhino Cave:

R108	108.0	5.756	443.6	0	150	340.2	73.4						
R195	195.0	0.984	190.8	0	150	8.9	46.0	400	575	21.7	195.3	-34.7	-70.1
R216	216.0	3.417	250.7	0	200	318.3	33.5	250	550	20.7	189.0	-10.8	-60.6
R246	246.0	5.341	523.9					250	575	33.0	165.9	-57.4	-77.8
R276	276.0	5.901	655.0	0	100	13.0	37.9	200	625	20.2	181.7	-21.3	-67.4
R316	316.0	2.694	579.4	0	150	46.1	49.7	300	675	14.5	208.4	-67.3	-63.4
R342	342.0	2.761	321.1	0	200	352.1	53.9	250	575	30.7	158.0	-36.8	-66.5
R392	392.0	2.167	262.7	0	150	23.9	49.0	200	550	30.0	196.6	-30.2	-67.1
R417	417.0	5.058	282.0										
R431	431.0	3.672	294.1	0	100	313.9	24.8						
R459	459.0	3.034	166.2										

Oulad Hamida:

oh220	220.0	0.029	59.2	0	100	334.0	12.4
oh230	230.0	0.039	39.0	0	200	335.9	57.6
oh237	237.0	0.041	51.4	0	150	2.3	42.4
oh249	249.0	0.044	58.1	0	275	352.1	37.7
oh279	279.0	0.027	63.8	0	225	330.4	41.1
oh298	298.0	0.017	59.5	0	100	15.4	27.8
oh305	305.0	0.036	81.2	0	200	326.6	14.0
oh325	325.0	0.183	147.4	0	100	353.4	51.4
oh345	345.0	0.143	66.4	0	150	329.2	36.7

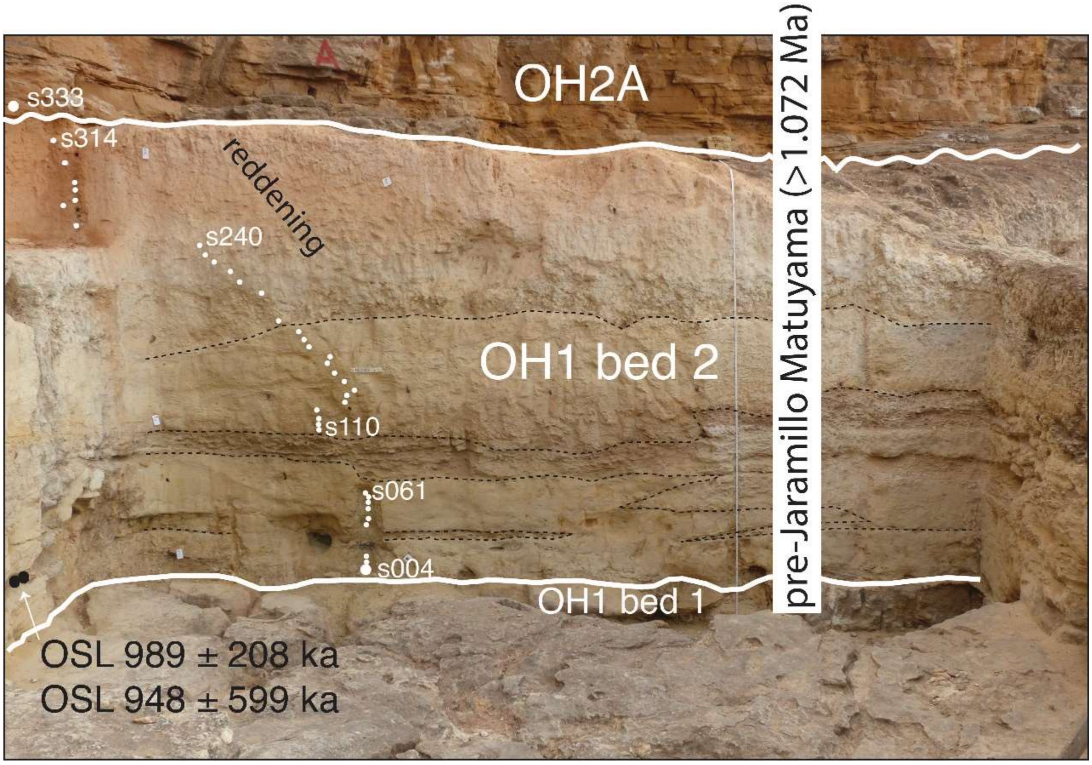

Fig. S8. Photograph of the stratigraphy of archeological section in unit OH1 Bed 2, trench south side with position of samples for magnetostratigraphy. OH1 Bed 2 is dated to the pre-Jaramillo Matuyama (>1.072 Ma) probably prior to the Cobb Mountain subchron. See main text for discussion.

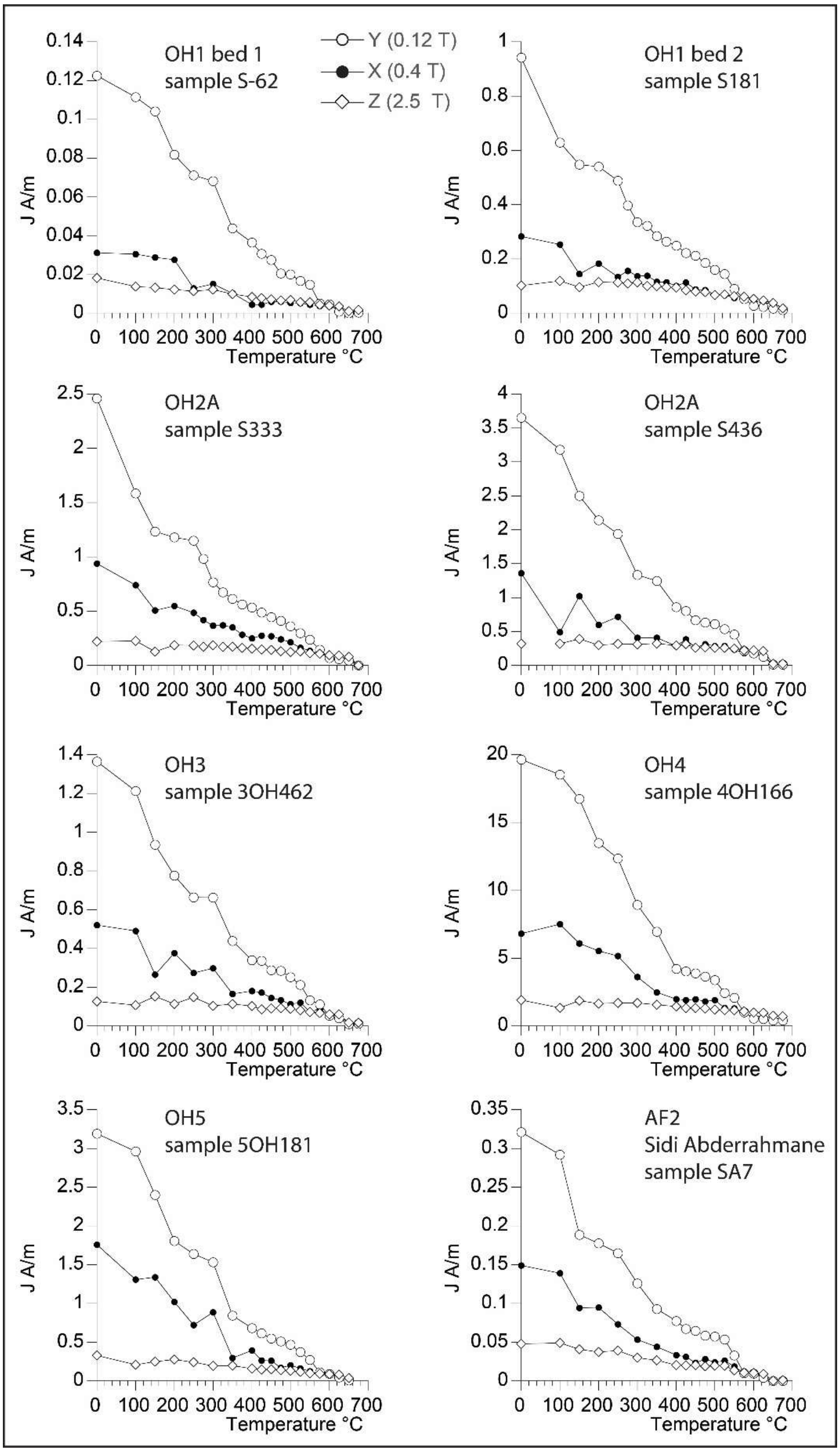

Fig. S9**.** Thermal demagnetization of a three component isothermal remanent magnetization (IRM) using fields of 2.5T, 0.4T, and 0.12T of selected samples from the investigated sedimentary units showing the predominance of magnetite as the carrier of the remanence coexisting with subsidiary hematite. See text for discussion.

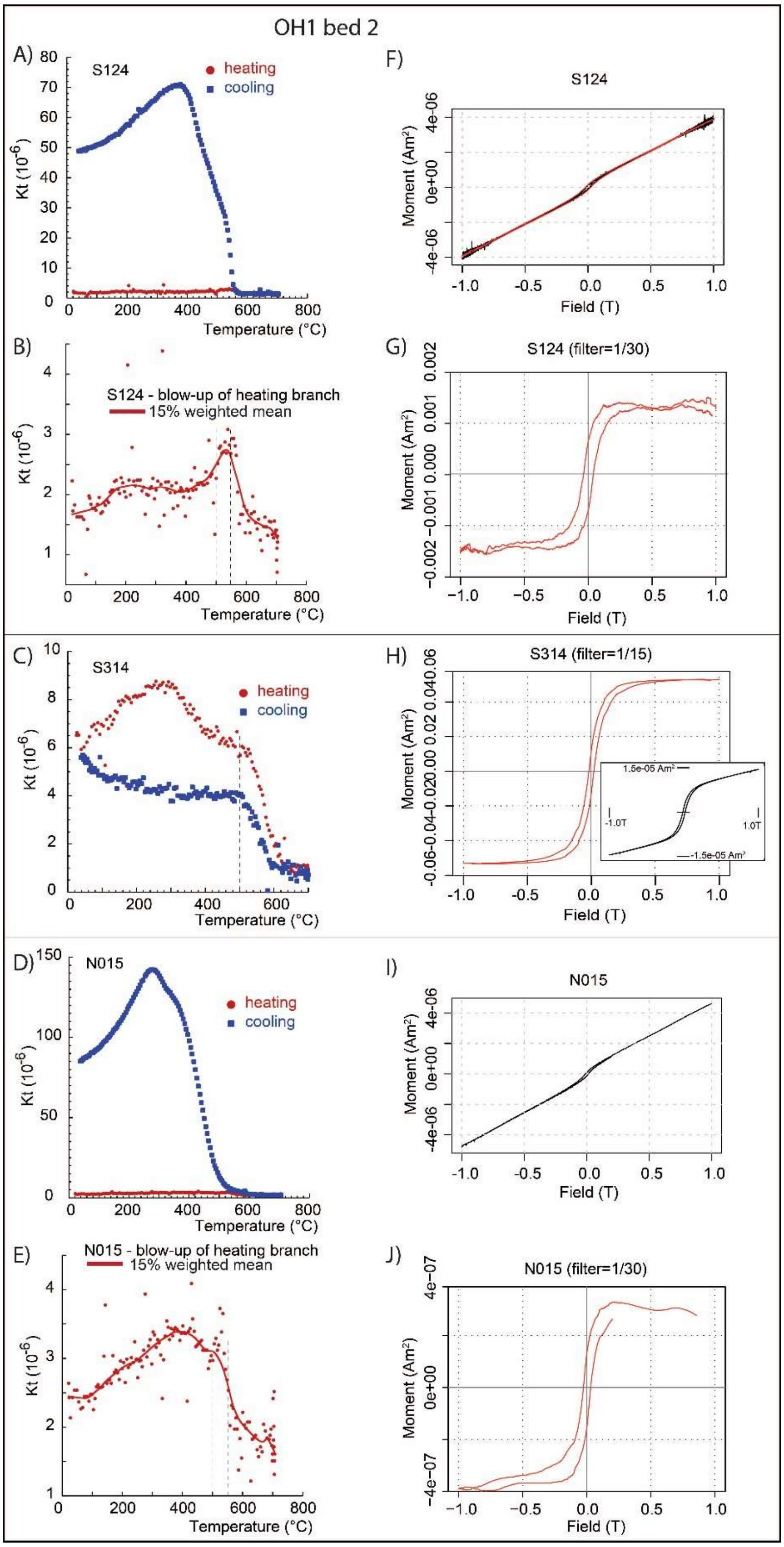

Fig. S10. Thermomagnetic (heating-cooling) cycles of magnetic susceptibility (A–E) and hysteresis experiments (F–J) on selected samples from OH1 Bed 2.

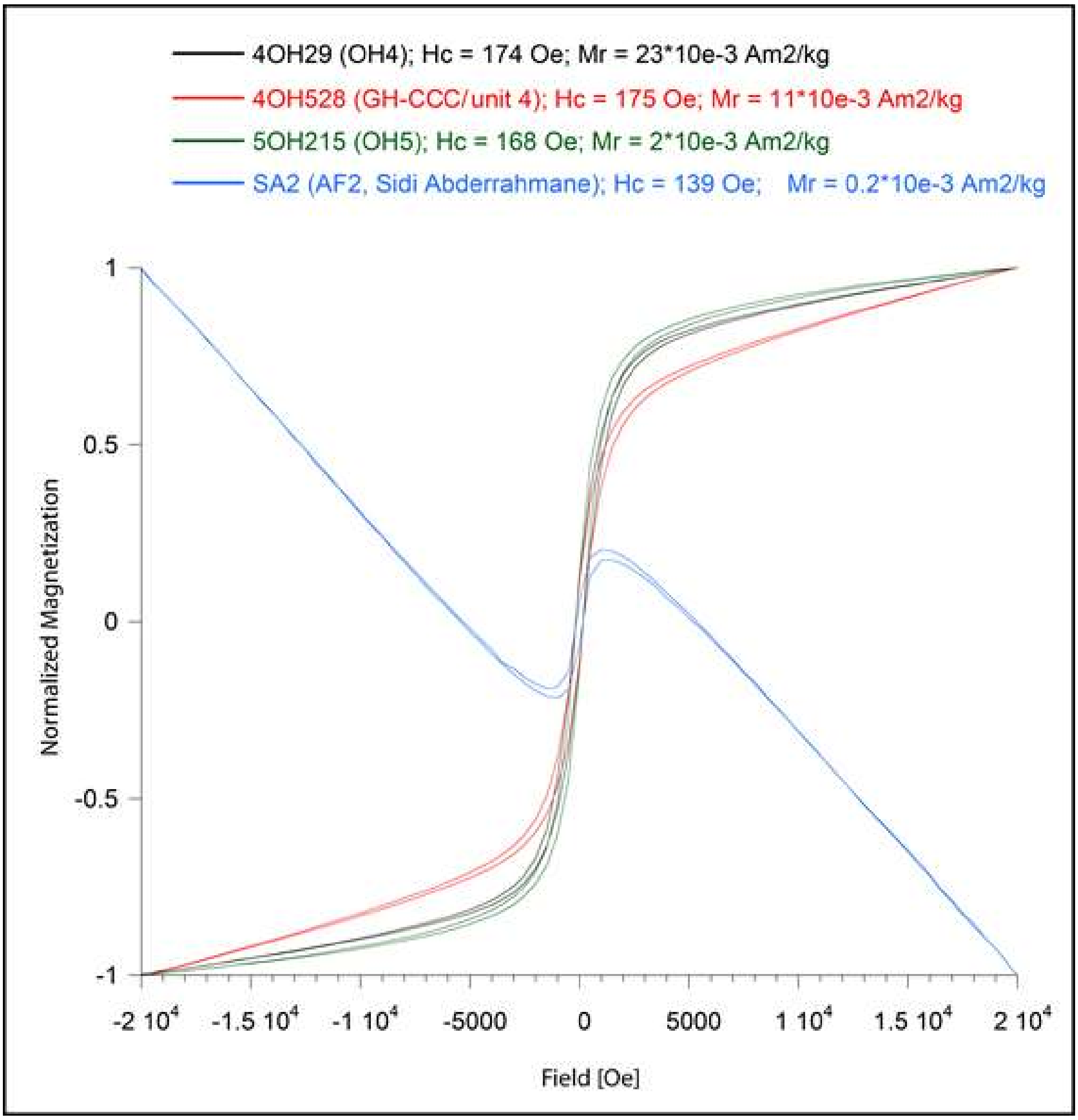

Fig. S11. Hysteresis experiments on samples from OH4, GH-CCC, OH5, and AF2 at Sidi Abderrahmane quarry. Analyses are non-corrected for paramagnetic (positive slope) or diamagnetic (negative slope) contributions.

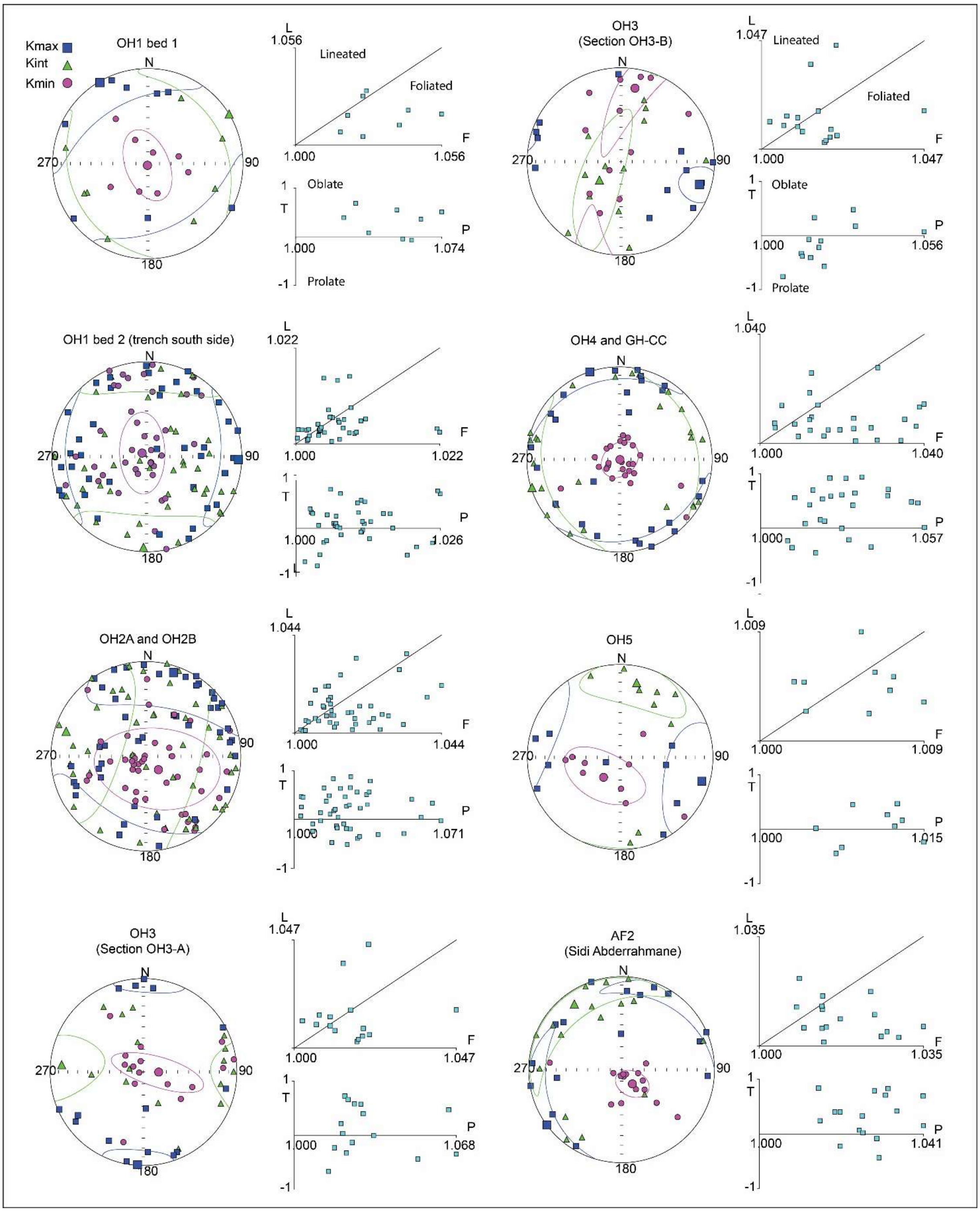

Fig. S12. Anisotropy of magnetic susceptibility data. See Material and Methods for anisotropy parameters definition and text for discussion.

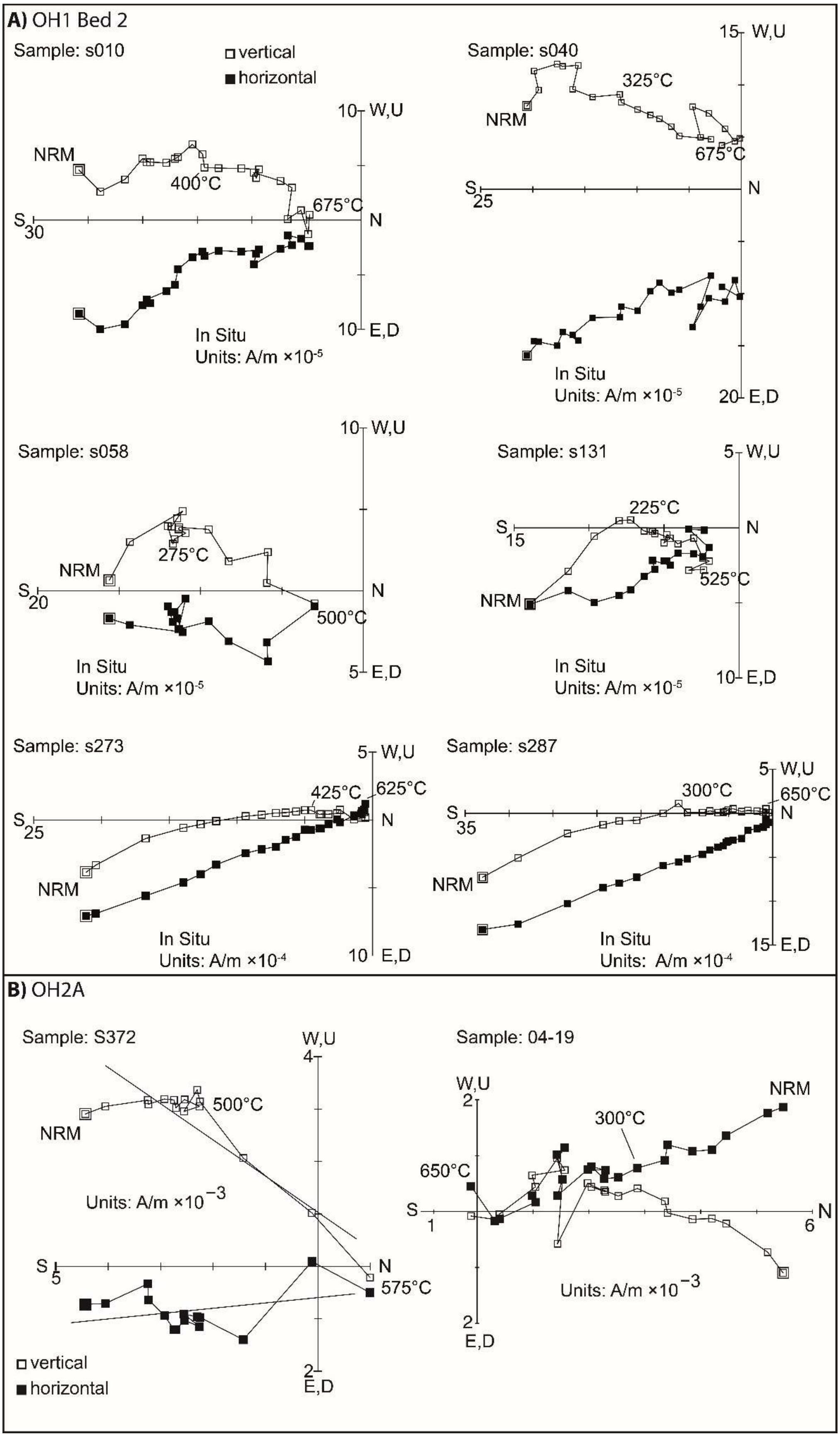
A) OH1 Bed 2
Sample: s010
vertical
horizontal
NRM
400°C
675°C
In Situ
Units: A/m ×10-5
Sample: s040
325°C
675°C
Sample: s058
275°C
500°C
Sample: s131
225°C
525°C
Sample: s273
425°C
625°C
Units: A/m ×10-4
Sample: s287
300°C
650°C
B) OH2A
Sample: S372
500°C
575°C
Units: A/m ×10−3
Sample: 04-19
300°C
650°C
W,U
E,D
S
N

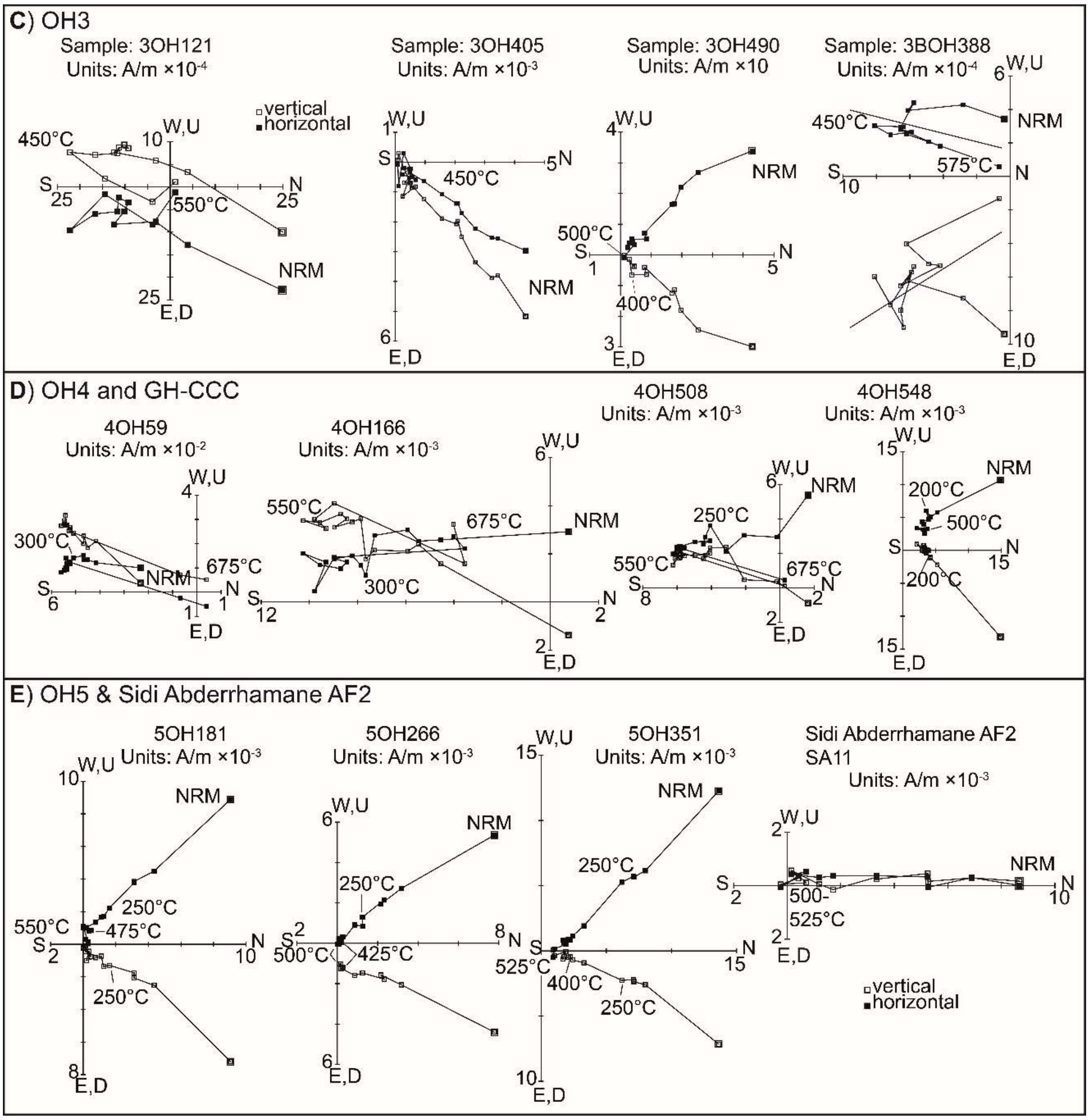

Fig. S13. Vector end-point demagnetization diagrams of representative samples from the main units investigated. (A) OH1 Bed 2, (B) OH2A, (C) OH3 (samples 3OH121, 3OH405, 3OH490 from section OH3-A, and sample 3BOH388 from section OH3-B), (D) OH4 and GH-CCC, (E) OH5, and AF2 at Sidi Abderrahmane Quarry. Filled/open symbols are projections onto the horizontal/vertical plane.

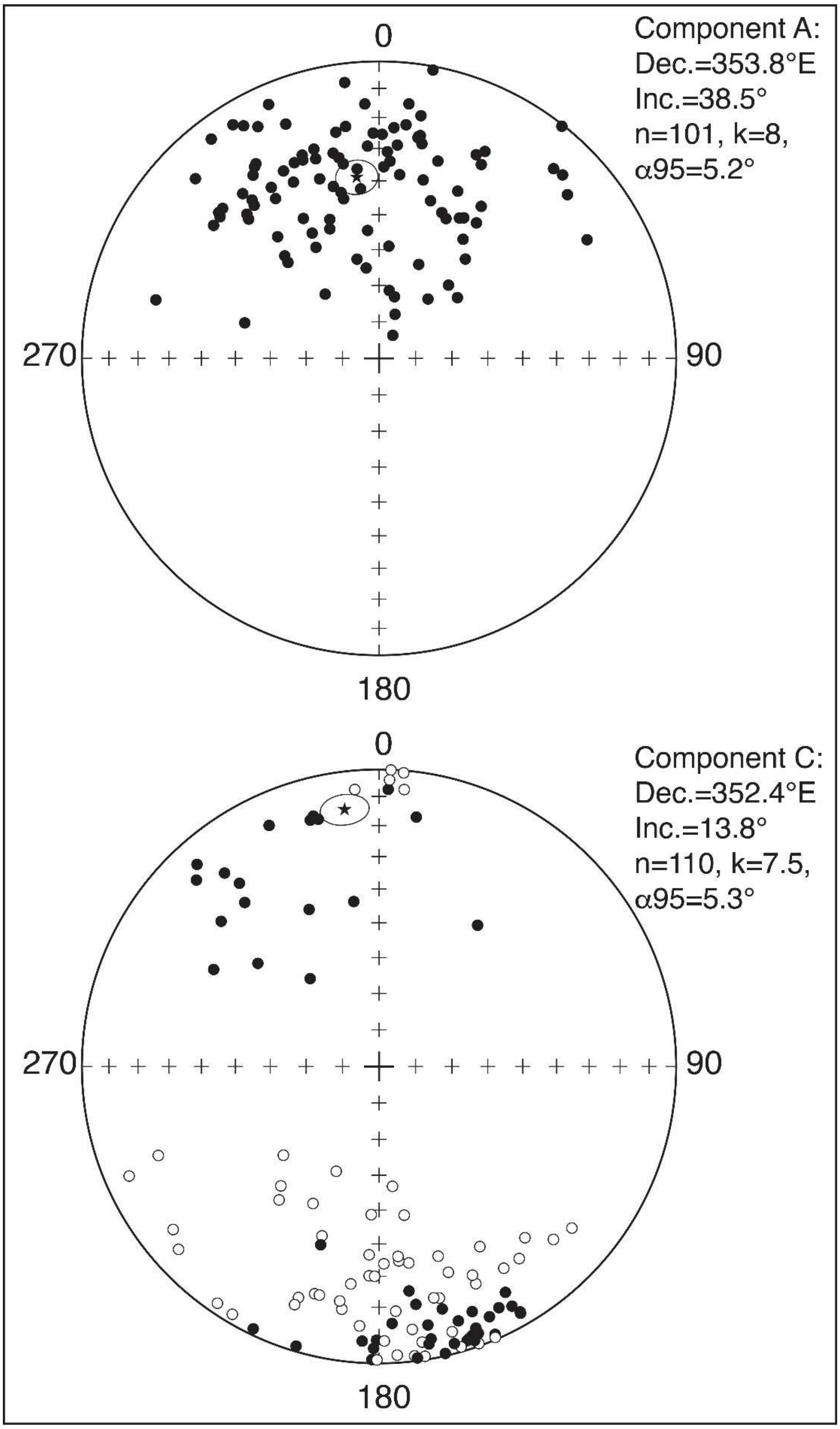

Fig. S14. Equal area plots and standard Fisher statistics of the A and C component directions from the studied sections. Filled/open symbols are projections on lower/upper hemisphere.

5. Geochemistry

Table S4. Elemental concentrations in the sediments of unit L from ThI at Casablanca.

Elevation (m asl)	Si (ppm)	K (ppm)	Ti (ppm)	Ca (ppm)	Mn (ppm)	Fe (ppm)	Zn (ppm)	Rb (ppm)	Sr (ppm)	Zr (ppm)
35.545	77149.8 ± 4067.82	16416.89 ± 454.75	3398 ± 63	208606.05 ± 3469.93	360 ± 7	21234.21 ± 492.28	18 ± 2	23.3 ± 1	362 ± 4	156 ± 3
35.495	61654.94 ± 3833.35	18513.31 ± 493.08	3155 ± 60	201851.37 ± 3463.41	393 ± 7	23105.03 ± 534.19	18 ± 2	25 ± 1	338 ± 4	180 ± 3
35.445	68138.75 ± 3944.82	17674.33 ± 495.12	2045 ± 53	196469.22 ± 3417.81	303 ± 7	21060.27 ± 508.05	18 ± 2	26.9 ± 1	328 ± 4	202 ± 3
35.395	65639.54 ± 4203.59	17522.37 ± 480.34	2196 ± 55	214413.21 ± 3831.63	300 ± 7	17829.56 ± 457.91	65 ± 3	24.7 ± 1	368 ± 5	175 ± 3
35.345	71562.66 ± 4180.67	16444.75 ± 443.28	2041 ± 51	222256.59 ± 3841.43	281 ± 6	17757.01 ± 446.39	45 ± 3	26.5 ± 1	335 ± 4	170 ± 3
35.295	73291.18 ± 4310.46	18250.44 ± 490.55	2042 ± 54	225529.04 ± 3960.91	288 ± 6	18393.48 ± 467.29	46 ± 3	26.9 ± 1	331 ± 4	156 ± 3
35.245	76411.8 ± 4437.09	16680.39 ± 469.41	1840 ± 52	242117.13 ± 4262.85	240 ± 6	16628.78 ± 442.25	34 ± 3	24.6 ± 1	287 ± 4	134 ± 2
35.195	71250.7 ± 4158.16	18223.7 ± 484.8	2412 ± 57	235223.83 ± 4003.93	287 ± 6	16789.7 ± 429.2	37 ± 3	26 ± 1	405 ± 5	191 ± 3
35.145	61735.91 ± 4047.23	17785.37 ± 474.98	2073 ± 53	226849.19 ± 3883.46	273 ± 6	15836.03 ± 409.46	36 ± 2	25.4 ± 1	394 ± 5	170 ± 3
35.095	85375.01 ± 4250.74	19196.35 ± 489.93	2655 ± 55	230325.56 ± 3780.57	309 ± 6	19004.98 ± 456.78	35 ± 2	29.4 ± 1	369 ± 5	205 ± 3
35.045	69144.83 ± 4087.91	17478.27 ± 467.2	2264 ± 53	230663.93 ± 3877	214 ± 6	17102.29 ± 428.55	33 ± 2	26.1 ± 1	363 ± 5	157 ± 3
34.995	81187.49 ± 4196.5	18866.65 ± 489.48	2321 ± 54	244832.65 ± 3989.01	243 ± 6	17188.08 ± 428.45	31 ± 2	26.6 ± 1	374 ± 5	178 ± 3
34.945	72577.81 ± 6016.02	19063.5 ± 1197.64	2772 ± 57	259247.16 ± 15383.06	212 ± 5	15912.08 ± 985.24	30 ± 2	23.2 ± 0.9	381 ± 5	193 ± 3
34.895	53970.24 ± 3686.64	14619.11 ± 416.42	1982 ± 52	233609.1 ± 3938.45	212 ± 6	16760.76 ± 423.31	32 ± 2	24.9 ± 1	421 ± 5	125 ± 2
34.845	56254.28 ± 3876.46	14940.69 ± 427.56	1834 ± 52	247159.73 ± 4125.53	188 ± 5	16612.72 ± 420.68	23 ± 2	22.9 ± 1	424 ± 5	181 ± 3
34.795	50079.35 ± 3755.99	13799.21 ± 406.97	2139 ± 54	250941.5 ± 4178.63	200 ± 5	17087.38 ± 428.19	28 ± 2	24.4 ± 1	450 ± 5	156 ± 3
34.745	63138.25 ± 4018.31	15062.47 ± 424.79	2057 ± 53	257071.37 ± 4293.22	202 ± 5	14772.7 ± 390.16	25 ± 2	19.5 ± 0.9	441 ± 5	169 ± 3
34.695	72562.14 ± 3954.18	14796.21 ± 404.27	2359 ± 54	273967.75 ± 4445.88	182 ± 5	14669.73 ± 388.22	25 ± 2	22.2 ± 0.9	413 ± 5	150 ± 3
34.645	56108.88 ± 3835.02	12333.04 ± 374.3	1945 ± 52	271541.8 ± 4449.91	235 ± 6	12835.12 ± 352.28	23 ± 2	18.6 ± 0.9	424 ± 5	119 ± 2
34.595	54404.41 ± 3946.71	11853.71 ± 384.44	2761 ± 60	257550.25 ± 4496.39	183 ± 5	13036.55 ± 374.28	19 ± 2	18.3 ± 0.9	383 ± 5	96 ± 2
34.545	47867.8 ± 3687.04	11551.96 ± 354.85	1817 ± 51	291833.43 ± 4702.12	160 ± 5	12981.48 ± 355.38	19 ± 2	17.4 ± 0.9	411 ± 5	133 ± 3
34.495	47851.17 ± 3720.12	12441.33 ± 385.55	1902 ± 52	271836.6 ± 4525.95	142 ± 5	13003.1 ± 362.12	15 ± 2	20.7 ± 0.9	413 ± 5	183 ± 3
34.445	51223.25 ± 3786.09	13629.79 ± 404.99	2408 ± 56	263789.3 ± 4394.47	165 ± 5	14133.37 ± 381.13	19 ± 2	19.7 ± 0.9	401 ± 5	148 ± 3
34.395	46083.65 ± 3708.39	11652.14 ± 364.16	1545 ± 50	310208.91 ± 4923.14	125 ± 5	10544.53 ± 312.48	9 ± 2	19.7 ± 0.9	338 ± 4	145 ± 3
34.345	58916.43 ± 3782.03	14071.73 ± 402.66	2365 ± 56	277447.71 ± 4413.26	198 ± 5	13464.38 ± 359.39	21 ± 2	21 ± 0.9	345 ± 4	112 ± 2
34.295	63029.06 ± 3895	15053.48 ± 423.93	2401 ± 54	264610.1 ± 4288.59	130 ± 4	13719.58 ± 367.28	27 ± 2	24.8 ± 1	338 ± 4	145 ± 3
34.245	76400.18 ± 4041.08	18669.82 ± 483.87	3605 ± 64	251346.5 ± 4018.19	176 ± 5	19169.66 ± 457.91	30 ± 2	26.1 ± 1	317 ± 4	184 ± 3
34.195	69586.49 ± 4049.22	18225.34 ± 483.92	2962 ± 59	220749.81 ± 3714.78	193 ± 5	17429.86 ± 433.89	24 ± 2	27.5 ± 1	355 ± 4	178 ± 3
34.145	56625.49 ± 4051.3	17720.2 ± 487.94	2861 ± 62	274342.44 ± 4728.3	223 ± 6	19274.65 ± 491.02	22 ± 2	25.7 ± 1	395 ± 5	205 ± 3
34.095	65656.26 ± 4118.57	17036.41 ± 456.4	3092 ± 62	250599.92 ± 4290.07	291 ± 6	19959.57 ± 487.9	28 ± 2	26.8 ± 1	373 ± 5	180 ± 3
34.04	61085.28 ± 4192.44	16086.67 ± 457.19	2791 ± 61	234284.74 ± 4198.3	189 ± 6	20163.59 ± 508.03	21 ± 2	25 ± 1	343 ± 4	147 ± 3
33.99	39237.62 ± 3652.39	13142.77 ± 395.43	1460 ± 53	303538.07 ± 4941.5	273 ± 6	17743.7 ± 448.03	26 ± 3	20.1 ± 1	420 ± 5	125 ± 3
33.945	30114.09 ± 3597.56	11166.22 ± 371.35	807 ± 48	324166.87 ± 5388.59	242 ± 6	12497.27 ± 365.05	15 ± 2	19.2 ± 1	470 ± 6	81 ± 2
33.895	41691.36 ± 3473.6	11536.59 ± 364.34	1387 ± 50	323067.54 ± 4690.57	302 ± 6	12549.63 ± 329.78	25 ± 3	19.3 ± 1	472 ± 6	62 ± 2
33.845	36397.94 ± 3678.23	12005.43 ± 380.9	1860 ± 53	328742.18 ± 5308.35	280 ± 6	12797.79 ± 365.02	23 ± 2	19.4 ± 1	464 ± 6	97 ± 2
33.795	30124.98 ± 3615.13	9784.15 ± 348.72	938 ± 47	292648.87 ± 5053.96	256 ± 6	11728.25 ± 351.39	23 ± 2	17 ± 0.9	531 ± 6	92 ± 2
33.745	31971.85 ± 3644.61	11057.73 ± 367.26	877 ± 46	323113.34 ± 5347.09	310 ± 6	11560.67 ± 346.35	27 ± 3	17.3 ± 0.9	499 ± 6	95 ± 2
33.695	28531.62 ± 3468.14	10085.59 ± 346.03	831 ± 46	333079.79 ± 5386.89	268 ± 6	10794.32 ± 327.55	25 ± 3	14.9 ± 0.9	506 ± 6	101 ± 2
33.645	28153.5 ± 3552.2	9800.69 ± 341.73	843 ± 47	338008.96 ± 5458.28	269 ± 6	10343.1 ± 319.85	22 ± 2	17.6 ± 0.9	481 ± 6	75 ± 2
33.595	32661.9 ± 3483.38	10754.4 ± 356.44	984 ± 49	320954.4 ± 5070.06	310 ± 7	13546.81 ± 370.38	26 ± 3	18.9 ± 1	487 ± 6	79 ± 2
33.545	32886.15 ± 3571.72	9773.29 ± 350.2	1282 ± 50	321279.69 ± 5275.27	275 ± 6	14852.34 ± 406.5	21 ± 2	15.8 ± 0.9	467 ± 6	119 ± 3
33.495	35012.26 ± 3689.57	8690.06 ± 330.69	1197 ± 51	312491.95 ± 5234.78	213 ± 6	12592.23 ± 365.53	11 ± 2	13.7 ± 0.9	450 ± 6	117 ± 2
33.445	34196.64 ± 3511.75	8652.15 ± 318.28	1505 ± 52	306086.69 ± 4880.44	238 ± 6	16860.29 ± 423.71	14 ± 2	12.8 ± 0.9	435 ± 5	163 ± 3
33.405	47082.37 ± 3728.35	6922.94 ± 292	2332 ± 55	284951.36 ± 4803.54	247 ± 6	13699.85 ± 381.05	15 ± 2	10.1 ± 0.8	456 ± 6	151 ± 3
33.365	60046.75 ± 3987.63	7072.07 ± 291.35	3792 ± 67	258958.31 ± 4363.67	258 ± 6	12525.32 ± 350.7	10 ± 2	11.3 ± 0.8	368 ± 5	218 ± 3
33.315	64485.72 ± 3964.72	7858.34 ± 294.9	4207 ± 68	276023.38 ± 4423.55	239 ± 6	12973.22 ± 348.63	13 ± 2	10.9 ± 0.8	382 ± 5	270 ± 4
33.215	98919.57 ± 4360.87	9230.95 ± 324.54	5323 ± 74	218609.02 ± 3452.32	283 ± 6	17043.37 ± 402.6	17 ± 2	13.1 ± 0.8	319 ± 4	236 ± 3
33.165	70075.51 ± 4202.47	10723.56 ± 366.47	2551 ± 56	254166.11 ± 4330.88	211 ± 6	16299.15 ± 428.46	18 ± 2	14.8 ± 0.9	366 ± 5	274 ± 4
33.115	61989.16 ± 4085.72	8985.1 ± 340.32	2374 ± 57	287437.53 ± 4823.63	198 ± 5	12438.15 ± 362.76	7 ± 2	13.6 ± 0.9	327 ± 4	136 ± 3
33.065	46849.6 ± 3723.74	9672.94 ± 336.66	2202 ± 54	282121.02 ± 4672.68	177 ± 5	12539.15 ± 353.14	18 ± 2	16.6 ± 0.9	382 ± 5	132 ± 3
33.015	51713.85 ± 3667.82	8603.69 ± 327.9	2555 ± 56	279524.95 ± 4480.37	216 ± 5	13059.42 ± 358.19	16 ± 2	14.1 ± 0.9	341 ± 4	185 ± 3
32.93	52122.12 ± 3750.94	8185.52 ± 324.28	2294 ± 51	266313.24 ± 4395.75	218 ± 5	11799.56 ± 338.83	16 ± 2	13.7 ± 0.8	330 ± 4	138 ± 2
32.89	68810.14 ± 3921.67	7726.63 ± 311.36	2336 ± 52	274471.22 ± 4311.88	216 ± 5	11563.78 ± 325.83	12 ± 2	13.7 ± 0.8	286 ± 4	174 ± 3
32.845	53297.93 ± 3575.37	8944.41 ± 325.19	1926 ± 51	261307.02 ± 4149.11	232 ± 6	11278.1 ± 316.14	13 ± 2	15.9 ± 0.9	303 ± 4	147 ± 3
32.795	29550.83 ± 3461.17	8065.48 ± 328.79	1668 ± 51	266992.35 ± 4659.86	239 ± 6	11143.65 ± 338.27	18 ± 2	15.9 ± 0.9	319 ± 4	90 ± 2
32.75	44504.25 ± 3636.08	10316.86 ± 363.58	1564 ± 49	263755.56 ± 4454.29	196 ± 5	11849.26 ± 343.27	20 ± 2	19.1 ± 0.9	372 ± 5	119 ± 2
32.71	67465.21 ± 3728.07	10114.22 ± 340.99	2646 ± 55	249731.71 ± 3881.04	241 ± 6	10924.58 ± 303.46	18 ± 2	16.9 ± 0.9	364 ± 4	166 ± 3
32.67	42266.4 ± 3347.5	11891.73 ± 366.58	1651 ± 50	273807.99 ± 4285.9	224 ± 6	11214.47 ± 313.77	21 ± 2	20 ± 0.9	374 ± 5	163 ± 3
32.625	34566.11 ± 3530.29	11075.77 ± 383.44	1293 ± 47	282601.74 ± 4779.88	187 ± 5	11977.91 ± 353.04	27 ± 2	20.4 ± 0.9	359 ± 5	115 ± 2
32.575	43284.87 ± 3503.23	11281.31 ± 372.16	1242 ± 49	283732.56 ± 4498.05	191 ± 5	11979.31 ± 336.53	18 ± 2	20.6 ± 1	365 ± 5	110 ± 2
32.51	48819.53 ± 3587.4	10501.44 ± 351.16	1303 ± 46	267551.51 ± 4299.8	189 ± 5	9558.9 ± 288.02	17 ± 2	19 ± 0.9	343 ± 4	116 ± 2
32.47	49366.02 ± 3493.18	12154.87 ± 379.28	1506 ± 48	261326.58 ± 4112.39	257 ± 6	11623.73 ± 321.13	31 ± 2	22.2 ± 0.9	358 ± 5	119 ± 2
32.43	44535.53 ± 3468.46	12171.74 ± 384.75	1679 ± 50	246571.75 ± 4028.43	233 ± 6	12284.46 ± 338.11	30 ± 2	26.1 ± 1	363 ± 5	207 ± 3
32.39	57649.3 ± 3612.45	13920.45 ± 408.43	1661 ± 48	248117.62 ± 3938.42	209 ± 5	14111.63 ± 365.25	26 ± 2	27.2 ± 1	358 ± 4	194 ± 3

32.35	61010.44 ± 3800.47	14798.13 ± 438.78	1660 ± 49	246037.87 ± 4042.97	268 ± 6	13639.01 ± 370.25	29 ± 2	25.9 ± 1	361 ± 5	186 ± 3
32.31	56394.36 ± 3680.83	14483.62 ± 417.91	1836 ± 49	246038.46 ± 3886.15	266 ± 6	12675.35 ± 336.91	31 ± 2	27.4 ± 1	365 ± 5	187 ± 3
32.27	52203.14 ± 3636.66	12280.72 ± 389.78	1677 ± 49	260887.9 ± 4217.13	189 ± 5	10687.31 ± 313.18	23 ± 2	22.7 ± 0.9	344 ± 4	163 ± 3
32.23	52163.37 ± 3665.88	12892.18 ± 400.77	1868 ± 49	251822.6 ± 4108.25	226 ± 5	11758.74 ± 331.48	26 ± 2	26.8 ± 1	341 ± 4	152 ± 3
32.19	54322.8 ± 3674.52	12487.89 ± 384.14	2781 ± 57	258227.44 ± 4169.5	247 ± 6	11297.98 ± 320.06	28 ± 2	21.9 ± 0.9	416 ± 5	201 ± 3
32.15	67671.32 ± 3657.86	11828.21 ± 365.69	3075 ± 57	244089.75 ± 3780.51	257 ± 6	11665.06 ± 316.19	27 ± 2	21.9 ± 0.9	285 ± 4	209 ± 3
32.11	45066.72 ± 3490.59	10334.55 ± 345.22	1651 ± 48	263743.81 ± 4132.33	198 ± 5	12401.92 ± 330.32	26 ± 2	20.8 ± 0.9	300 ± 4	151 ± 3

Table S5. Parameters of coastal deposits related to sea-level high-stands used to estimate uplift in the area of Casablanca from ca. 2.5 to 1 Ma. Coastal deposits identified from Lefèvre and Raynal[1]. Chronological data from this study for OH3 and from Geraads[14] for AAO. Minimum and maximum sea-level high-stand estimated from minimum and maximum relative sea level in Supplementary Fig. S17A, respectively. Uplift of coastal deposits related to sea-level high-stand is calculated by subtracting the mean relative sea level from the mean elevation of intertidal deposits. Uncertainties of uplift for a sea-level high-stand = $[(\text{Maximum relative sea level} - \text{Minimum relative sea level})^2 + (\text{Maximum elevation of intertidal deposits} - \text{Minimum elevation of intertidal deposits})^2]^{0.5}$.

Coastal deposits related to sea-level high-stand	Chronological data of intertidal deposits	Minimum / maximum elevation of intertidal deposits (m asl)	Minimum / maximum sea-level high-stand (m)	Estimated uplift (m)
OH3 (Oulad Hamida Formation)	1075-1070 ka (Slightly earlier than Matuyama-Jaramillo transition)	34 / 37	-15 / 0	43.0 ± 7.6
AAO (Ahl al Oughlam Formation)	2500-2400 ka (biostratigraphy)	100 / 105	-35 / 15	112.5 ± 25.1

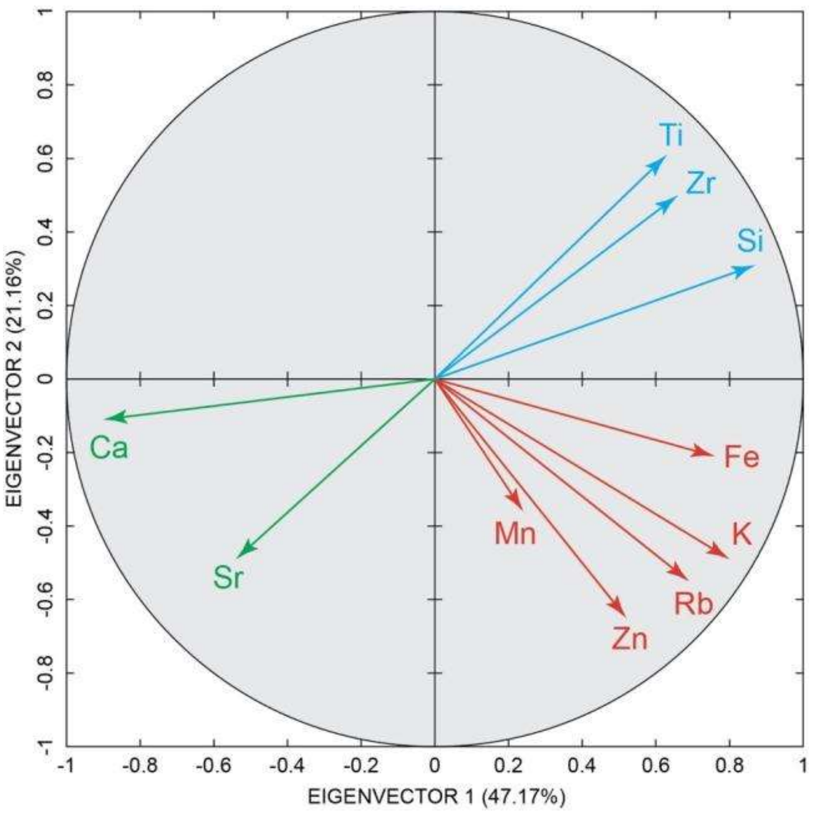

Fig. S15. Correlations between the initial variables (element concentrations) and the two first eigenvectors of the principal component analysis of geochemical data from the sediments of ThI-L. The principal component analysis shows three groups of elements. The first group (Ca, Sr) can be related to detrital sediments eroded from local carbonate rocks (calcarenite). The second group (K, Mn, Fe, Zn, Rb) is representative of chemically weathered sediments, with carbonate dissolution leading to an enrichment in K and Rb relatively to Ca and Sr[66-68], while Mn, Fe and Zn are typically associated with redox processes[69-73]. The third group (Si, Ti, Zr) is supposed to be representative of Saharan dust input in the sediments of unit L, given that these three elements are typical of aeolian dust transported from the Sahara Desert[69-70].

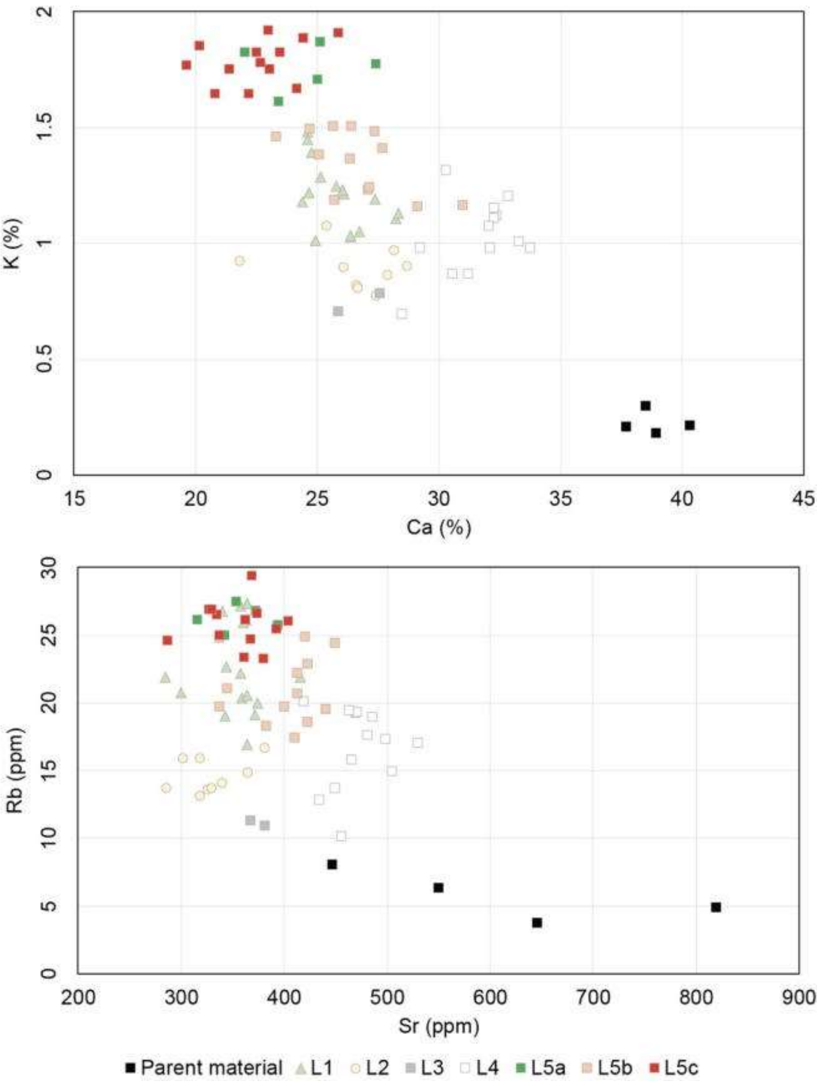

Fig. S16. Plots of K vs. Ca and Rb vs. Sr for the sediments of unit L. The samples of parent material were collected in calcarenite underlying unit L. Elements representative of parent rocks (Ca, Sr) are depleted in weathered subunits. K and Rb are increasingly enriched relatively to Ca and Sr, respectively, with an increasing degree of weathering.

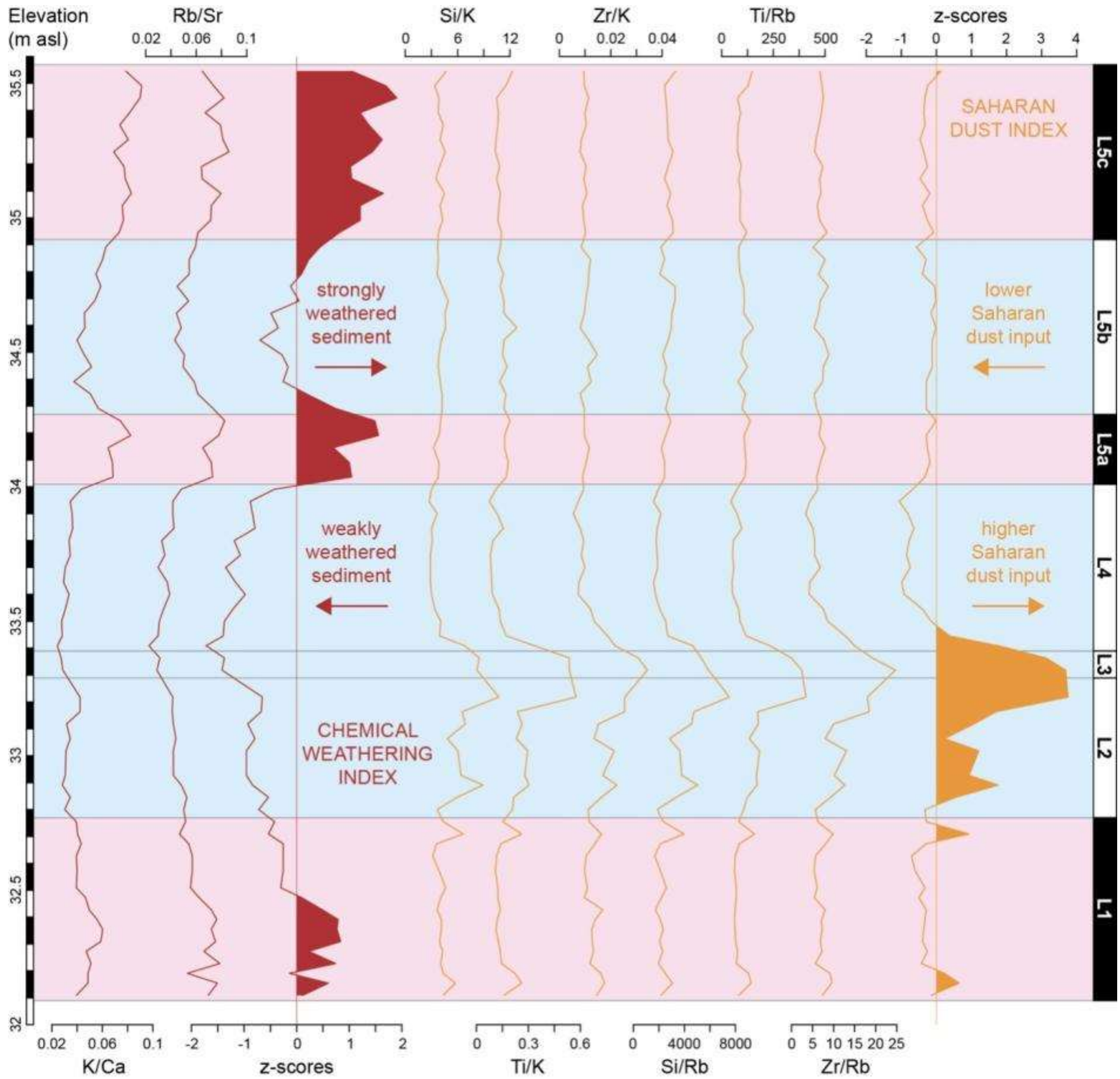

Fig. S17. Elemental ratios for the sediments of ThI-L. Rb/Sr and K/Ca ratios are useful for estimating the degree of chemical weathering of Pleistocene sediments[66,67,74,75]. A chemical weathering index was calculated from the average of z-scores of Rb/Sr and K/Ca ratios. The elements representative of eolian input from the Sahara (Si, Ti, Zr) were normalized to K and Rb, which can be associated with fluvial input[69,70,76,77]. An index of Saharan dust input was calculated from the average of z-scores of Si/K, Ti/K, Zr/K, Si/Rb, Ti/Rb and Zr/Rb ratios.

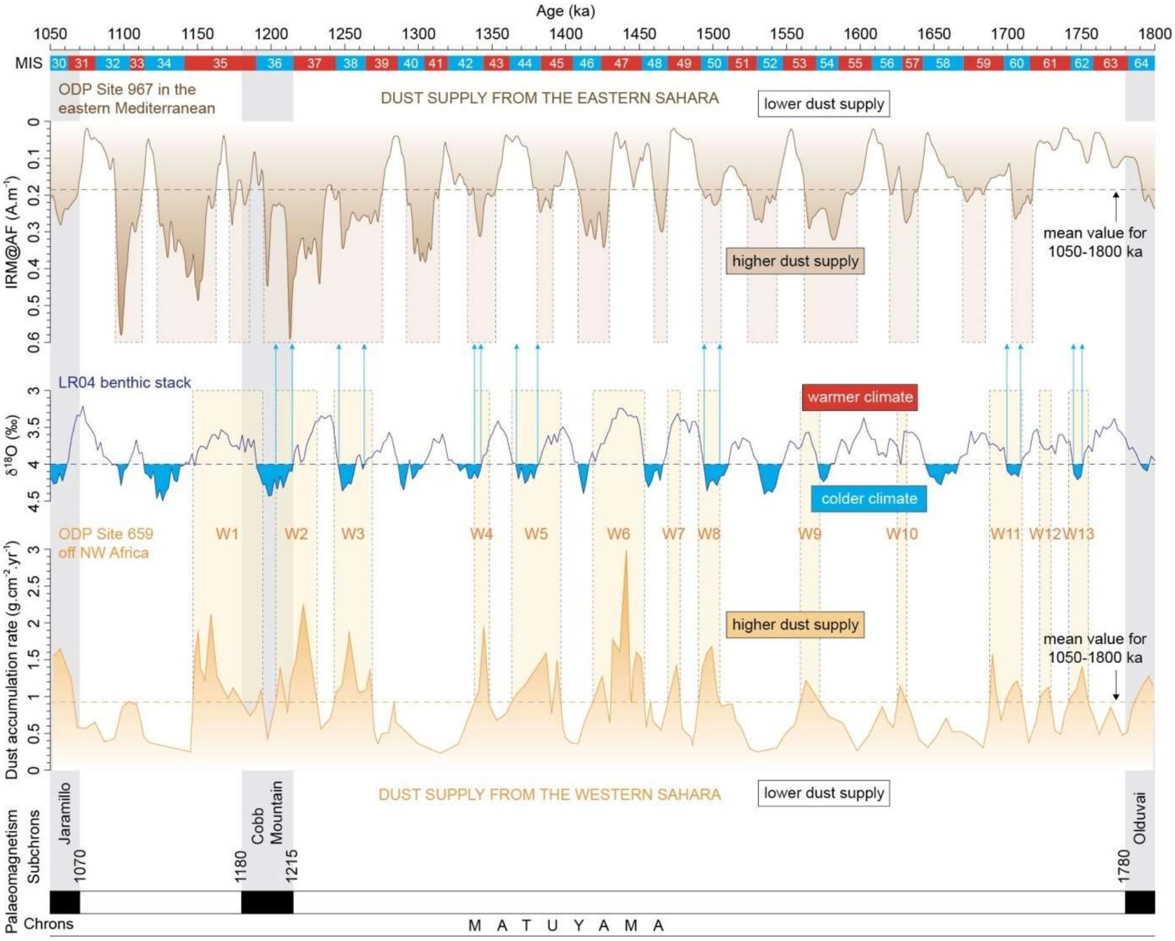

Fig. S18. Dust supply from the western and eastern Sahara between the Jaramillo and Olduvai subchrons. The most proximal marine records of Saharan dust supply around Morocco are located off northwestern Africa (ODP Site 659) and in the eastern Mediterranean (ODP Site 967), and are representative of dust flux from the western Sahara in northwestern Africa and from the eastern Sahara in northeastern Africa, respectively[78]. Hence, the dust record from ODP Site 659 should be preferentially used for comparison of Saharan dust input in unit L with Saharan dust supply recorded in marine sediments, which allows to distinguish thirteen periods of higher than average dust supply from the western Sahara between the Jaramillo and Olduvai subchrons (W1-W13, pale yellow vertical bands). However, dust supply from this $CaCO_3$-based proxy record is potentially biased and overestimated during glacial maximum owing to deep ocean circulation changes that led to increased dissolution of calcium carbonate[79]. Seven periods (W2-W5, W8, W11 and W13, blue arrows) occurred partly during glacial maximum characterized by values of the LR04 $\delta^{18}O$ benthic stack higher than 4 ‰. The reliability of these periods was controlled and validated by a comparison with the periods of higher than average dust supply highlighted by the dust record from ODP Site 967 (light brown vertical bands). Amongst the seven above-mentioned periods, only W13 and the later half of W5 were not associated with periods of high Saharan dust supply at ODP Site 967 and could thus be artifacts. Saharan dust proxy record from ODP Site 659 (dust accumulation rate) from Tiedemann et al.[80]. Saharan dust proxy record from ODP Site 967 (IRM@AF) from Larrasoaña et al.[81] with age model from Grant et al.[82]. Marine Isotopic Stages (MIS) and LR04 benthic stack from Lisiecki and Raymo[83]. Palaeomagnetic record from Channell et al.[84].

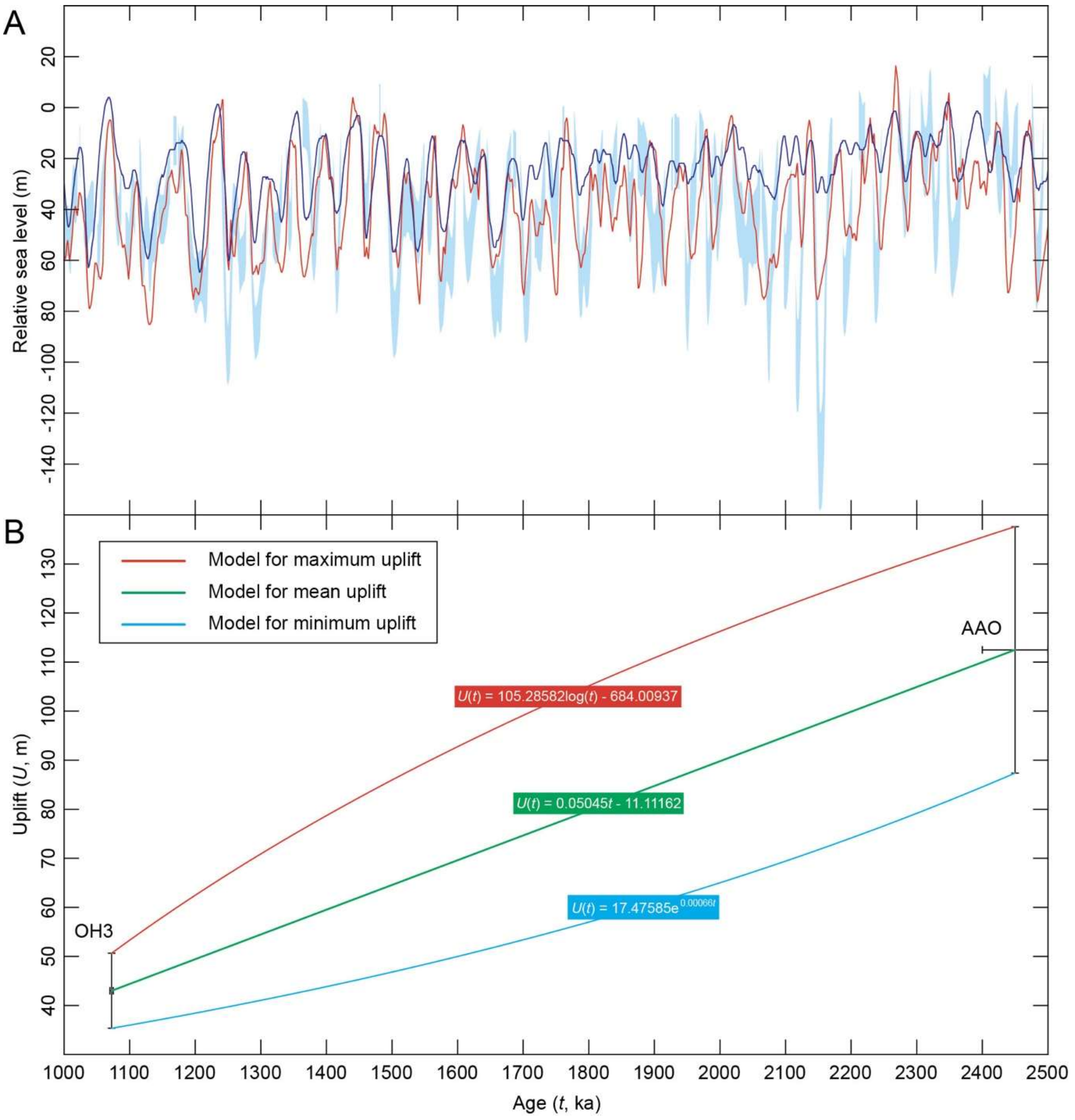

Fig. S19. Estimates of relative sea-level (RSL) changes (A) and uplift in the area of Casablanca from ca. 2.5 to 1 Ma (B). A: blue shaded area: RSL from Rohling et al.[85] corrected by Amies[86]; dark blue line: RSL from de Boer et al.[87]; red line: RSL from SPECMAP data[88] rescaled with 121 m sea level fall at 17 ka[89] corresponding to a $\delta^{18}O$ value of ca. 1.78. All RSL data were resampled at 1 ka interval. B: Maximum, mean and minimum uplift estimated for sea-level high-stands OH3 and AAO were used in order to test three uplift models based on logarithmic, linear and exponential regressions, respectively, assuming no subsidence during the Quaternary[4,90]. OH3: sea-level high-stand of the Oulad Hamida formation; AAO: sea-level high-stand of the Ahl al Oughlam Formation. The parameters of sea-level high-stands are listed in Supplementary Table S5.

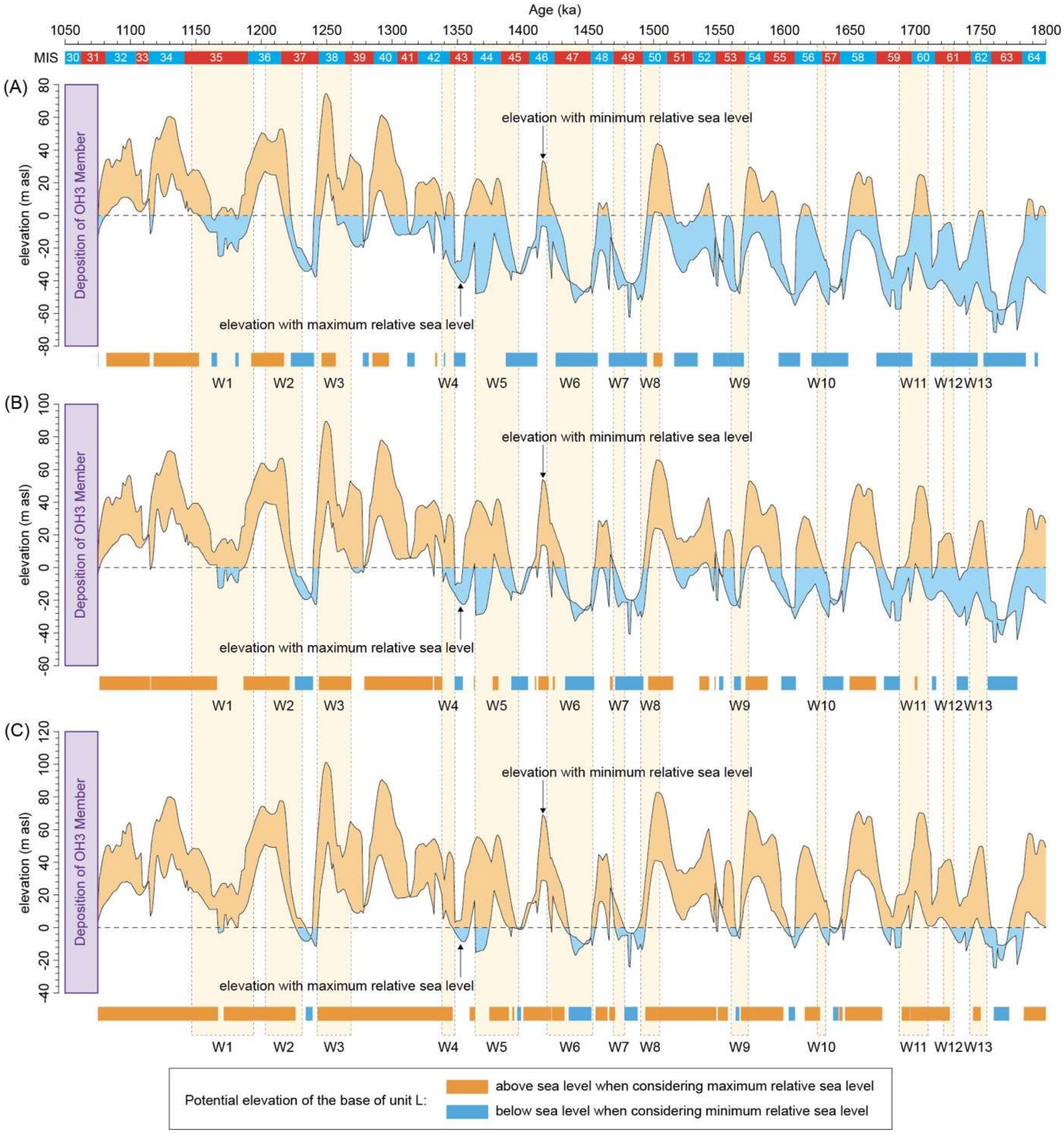

Fig. S20. Potential elevation of the base of unit L during the periods of higher than average dust supply from the western Sahara (W1-W13, pale yellow vertical bands) for different uplift models. A: Logarithmic model for maximum uplift. B: Linear model for mean uplift. C: Exponential model for minimum uplift. Elevation was calculated by subtracting relative sea level and uplift from the present elevation of the base of unit L (ca. 32 m above sea level). Maximum and minimum relative sea level were determined from Supplementary Fig. S19A. Uplift was estimated from models in Supplementary Fig. S19B. Palaeo-elevation reconstruction is supposed to give potential elevations because it does not take into account potential periods of sedimentation and erosion at the site of Thomas Quarry I before the deposition of unit L. Marine Isotopic Stages (MIS) from Lisiecki and Raymo[83].

6. Palynology

Fossil pollen data from Th-L1 (Supplementary Table S6) show that the vegetal landscape was dominated by a steppe (66.8%) with Compositae (42.5 %), Poaceae (4 %) Chenopodiaceae (3.7 %) and varied plants: *Convolvulus*, *Helianthemum*, and *Reseda*. Regionally, an open Mediterranean forest (Arboreal Pollen: 26.7 %) was present. It consists mainly of deciduous *Quercus* (9.9 %), *Pinus* (1.2 %) and *Olea* (0.3 %). *Myrtus* (11.2%) could develop either in the oak wood undergrowth or in wet depressions[91]. Edible plants were identified in the pollen assemblage (Supplementary Table S6). They correspond to 76% of all the identified phanerogams. They could constitute a significant part of the potential diet of the hominins.

6.1 Methods. The Casablanca region is located in the sclerophyllous forest ecoregion[92] which is nowadays degraded by human activities.
Of the seven samples taken from profile 19 of the ThI-L, only sample CT 19-01 was polliniferous. The sporo-pollinic material was extracted from the sediment using chemical treatment (HCl acid, NaOH 10% and acetolysis), a flotation in heavy liquid (d = 2) and 160 + 10 µm sieving. The identification was carried out with a photonic microscope (x500 magnification). The palynological identifications were based on the pollen reference collection of IMBE (CNRS, Aix-en-Provence, France) and the bibliography[93-96]. The pollen percentages were calculated on a Pollen Sum (PS: 322) including only the phanerogams. The weight of the pollen sample is: 15.6 g. The analysed volume is 45 µl. The Absolute pollen Frequency is of 20.6 pollen grain/gram. The biodiversity is high: in total, 42 taxa were identified, among them 38 phanerogams et 4 non pollen palynomorphs (charcoal (very few) and unidentifiable insect, chironomid and fungi).

Table S6. Phanerogams and edible plants (in bold) of the pollen sample CT 19-1. Values are in counting values and in percentages (calculated on a pollen sum including all the phanerogams). The systematic is from Cronquist[97].

Sample	Counting value	%	Edible organ	Sample	Counting value	%	Edible organ
Acer sp	2	0.62		***Hippophae rhamnoides***	4	1.24	Fruit
***Ammi* t.**	14	4.35	Seed	***Hypericum* sp.**	1	0.31	Leaf
**Apiaceae**	7	2.17	Leaf, stem, root	***Linum usitatissimum***	2	0.62	Seed
***Artemisia* sp.**	31	9.63	Leaf, shoot	***Myrtus* sp.**	36	11.18	Fruit, bud
***Aster* t.**	2	0.62	Leaf, stem	*Odontites* sp.	3	0.93	
***Atriplex* t.**	7	2.17	All	***Olea* sp**	1	0.31	Fruit
Blackstonia t.	3	0.93		***Pimpinella* sp.**	1	0.31	Seed
Bupleurum sp.	1	0.31		***Pinus sylvestris* t.**	2	0.62	Seed, young shoot
***Calendula* t.**	1	0.31	Leaf, flower	***Pinus* (mediterranean)**	2	0.62	Seed, young shoot
***Carduus* t.**	2	0.62	Leaf	**Poaceae**	13	4.04	Young shoot, leaf
**Chenopodiaceae**	5	1.55	Leaf	***Polygonum aviculare* sp.**	2	0.62	Seed, young leaf and plant
**Cichorioideae**	100	31.06	Leaf, flower, stem	***Prunus* sp.**	4	1.24	Fruit
***Cirsium* t.**	1	0.31	Leaf, flower, stem	***Quercus* sp. (deciduous)**	32	9.94	Fruit
Convolvulus sp	*1*	0.31	Leaf, young shoot	*Ranunculus* sp.	2	0.62	
**Cyperaceae**	3	0.93	Root	***Reseda* sp.**	5	1.55	Young shoot, leaf
Euphorbia sp.	7	2.17		*Scutellaria* t.	12	3.73	
***Galium* sp**	1	0.31	Leaf	***Sinapis* t.**	3	0.93	Seed, leaf
Gentiana sp.	3	0.93		***Urtica* sp.**	1	0.31	Leaf
Helianthemum sp.	2	0.62		***Vitis* sp.**	3	0.93	Fruit

References

1. Lefèvre, D. & Raynal, J.-P. Les formations plio-pléistocènes de Casablanca et la chronostratigraphie du Quaternaire marin du Maroc revisitées, *Quaternaire* **13**, 9-21 (2002).

2. Lecointre, G. *Recherches sur le Néogène et le Quaternaire marins de la côte atlantique du Maroc* (Mémoires du Service Géologique du Maroc, 99, t.1 : Stratigraphie, 1952).

3. Biberson, P. *Le Paléolithique inférieur du Maroc atlantique* (Publications du Service des Antiquités du Maroc 17, 1961).

4. Stearns, C. E. Pliocene-Pleistocene emergence of the Moroccan Meseta. *Geol. Soc. Am. Bull.* **89**, 1630-1644 (1978).

5. Texier, J.-P., Lefèvre, D., Raynal, J.-P. & El Graoui, M. Lithostratigraphy of the littoral deposits of the last one million years in the Casablanca region (Maroc). *Quaternaire* **13**, 23-41 (2002).

6. Texier, J.-P. *et al.* Contribution pour un nouveau cadre stratigraphique des formations littorales Quaternaires de la région de Casablanca (Maroc). *Cr. Acad. Sci. Paris* **318**, séries II, 1247-1253 (1994).

7. Biberson, P. *Le cadre paléogéographique de la Préhistoire du Maroc atlantique* (Publications du Service des Antiquités du Maroc 16, 1961).

8. Lefèvre, D., Raynal, J.-P. & El Graoui, M. Le Quaternaire d'Oulad Hamida in P*réhistoire de Casablanca. I - La grotte des Rhinocéros (fouilles 1991 et 1996)* (eds. Raynal, J.-P. & Mohib, A.) 45-59 (Institut National des Sciences de l'Archéologie et du Patrimoine, VESAM volume VI, 2016).

9. Raynal, J.-P. & Texier, J.-P. Découverte d'Acheuléen ancien dans la carrière Thomas 1 à Casablanca et problème de l'ancienneté de la présence humaine au Maroc, *C. R, Acad. Sci.* II **308**, 1743-1749 (1989).

10. Raynal, J.-P., Sbihi-Alaoui, F.Z., Geraads, D., Magoga, L. & Mohib, A. The earliest occupation of North-Africa: the Moroccan perspective. *Quat. Int.* **75**, 65-75 (2001).

11. Raynal, J.-P., Sbihi-Alaoui, F.Z., Magoga, L., Mohib, A. & Zouak, M. The Lower Palaeolithic Sequence of Atlantic Morocco Revisited After Recent Excavations at Casablanca. *Bulletin d'archéologie marocaine* **20**, 44-76, *(*2004).

12. Raynal, J.-P. *et al.* Contextes et âge des nouveaux restes dentaires humains du Pléistocène moyen de la carrière Thomas I à Casablanca (Maroc). *Bull. Soc. Prehist. Fr.* **108**, 645-669 (2011).

13. Raynal, J.-P. *et al.* Hominid Cave at Thomas Quarry I (Casablanca, Morocco): recent findings and their context. *Quat. Int.* **223-224**, 369-382 (2010).

14. Geraads, D. Plio-Pleistocene mammalian biostratigraphy of Atlantic Morocco. *Quaternaire* **13**, 43-53 (2002).

15. Geraads, D. Biochronologie mammalienne du Quaternaire du Maroc atlantique, dans son cadre régional, *L'Anthropologie* ***114***, 324-340 (2010).

16. Rhodes, E. J., Singarayer, J., Raynal, J.-P., Westaway, K. & Sbihi-Alaoui, F.-Z. New age estimations for the Palaeolithic assemblages and Pleistocene succession of Casablanca, Morocco. *Quat. Sci. Rev.* **25,** 2569-2585 (2006).

17. Guilloré, P. *Méthode de fabrication mécanique et en série des lames minces* (Document du département des sols de l'I.N.A. Paris-Grignon, 1980).

18. El Graoui, M. PhD dissertation (Université de Bordeaux 1, 1994).

19. Cremaschi, M., Trombino, L. & Zerboni, A. Palaeosoils and relict soils, a systematic review in *Interpretation of micromorphological features of soils and regoliths – Revised Edition* (eds. Stoops, G., Marcelino, V. & Mees, F.) 863–894 (Elsevier, 2018).

20. Courty, M.-A. Microfacies analysis assisting archaeological stratigraphy in *Earth sciences and archaeology* (eds. Goldberg, P., Holliday, V. T. & Ferring C.R.) 205–239 (Kluwer Academic/Plenum Publishers, 2001).

21. Goldberg, P. & Macphail, R. I. *Practical and theoretical geoarchaeology* (Blackwell Publishing, 2006).

22. Goldberg, P., Berna, F. Micromorphology and context. *Quat. Int.* **214**, 56–62 (2010).

23. Stoops, G. Fluorescence microscopy in *Archaeological Soil and Sediment Micromorphology* (eds. Nicosia, C. & Stoops G.) 393–397 (John Wiley & Sons, 2017).

24. Stoops, G. *Guidelines for Analysis and Description of Soil and Regolith Thin Sections* (Soil Science Society of America Inc., 2003).

25. Courty, M. A., Goldberg, P. & Macphail, R. I. *Soils and Micromorphology in Archaeology* (University Press, 1989).

26. Nicosia, C. & Stoops, G. *Archaeological Soil and Sediment Micromorphology* (John Wiley & Sons, 2017).

27. Stoops, G., Marcelino, V. & Mees, F. *Interpretation of Micromorphological Features of Soils and Regoliths,* (Elsevier, 2nd ed, 2018).

28. Ward, W. C. Petrology and diagenesis of carbonate eolianites on northwestern Yucatan Peninsula, Mexico in *Belize Shelf--Carbonate Sediments, Clastic Sediments, and Ecology* (eds. Pusey, W. C. & Wantland K. F.) 500-571 (AAPG Special Volumes, 1975).

29. Wieder, M. & Yaalon, D. H. Effect of matrix composition of carbonate nodule crystallisation. *Geoderma* **11**, 95–121 (1974).

30. Monger, H. C., Daugherty, L. A. & Gile, L. H. A microscopic examination of pedogenic calcite in an Aridisol of southern New Mexico in *Occurrence, Characteristics, and Genesis of Carbonate, Gypsum, and Silica Accumulations in Soils* (ed. Nettleton, W. D.) 37–60 (Soil Science Society of America Journal Special Publication 26, 1991).

31. Verrecchia, E. P. Lacustrine and palustrine geochemical sediments, in *Geochemical Sediments and Landscapes (*eds. Nash, D. J. & McLaren, S. J.) 298–329 (Blackwell, 2007).

32. Durand, N., Monger, H. C., Canti, M. G. & Verrecchia, E. P. Calcium carbonate features in *Interpretation of micromorphological features of soils and regoliths – Revised Edition* (eds. Stoops, G., Marcelino, V. & Mees, F.) 205–258 (Elsevier, 2018).

33. Tsatskin, A. & Ronen, A. Micromorphology of a Mousterian paleosol in aeolianites at the site Habonim, Israel. *Catena* **34**, 365–384 (1999).

34. Zhao, Q. *et al*. Sedimentary facies and evolution of aeolianites on Shidao Island, Xisha Islands. *Chin. J. Oceanol. Limn.* **29**, 398–413 (2011).

35. Li, R. *et al.* Composition and diagenesis of Pleistocene aeolianites at Shidao, Xisha Islands: Implications for palaeoceanography and palaeoclimate during the last glacial period. *Palaeogeogr. Palaeocl. Palaeoecol.* **490**, 604–616 (2018).

36. Shtienberg, G. *et al.*, New perspectives on coastal landscape reconstruction during the Late Quaternary: A test case from central Israel. *Palaeogeogr. Palaeocl. Palaeoecol.* **468**, 503–519 (2017).

37. Brooke, B. The distribution of carbonate eolianite. *Earth Sci. Rev.* **55**, 135–164 (2001).

38. Simpson, E. L. & Eriksson, K. A. Thin eolianites interbedded within a fluvial and marine succession: early Proterozoic Whitworth Formation, Mount Isa Inlier, Australia. *Sediment. Geol.* **87**, 39–62 (1993).

39. Hearty, P. J. Stratigraphy and timing of eolianite deposition on Rottnest Island, Western Australia. *Quat. Res.* **60**, 211–222 (2003).

40. Assal, E. M., Adbel-Fattah, Z. A. & El-Asmar, H. M. Facies architecture and controlling factors induced depositional model of the Quaternary carbonate eolianites in the northwestern Mediterranean coast of Egypt. *Int. J. Earth Sci.* **109**, 1659–1682 (2020).

41. Zerboni, A. Micromorphology reveals in situ Mesolithic living floors and archaeological features in multiphase sites in central Sudan. *Geoarchaeology* **26**, 365-391 (2011).

42. Miller, C. E. Trampling in *Encyclopedia of Geoarchaeology* (ed. Gilbert, A.S.) 981–982 (Springer, 2017).

43. Villagran, X. S., Huisman, D. J., Mentzer, S. M., Miller, C. E. & Jans, M. M. Bone and Other Skeletal Tissues in *Archaeological Soil and Sediment Micromorphology* (eds. Nicosia, C. & Stoops, G.) 11–38 (John Wiley & Sons, 2017).

44. Karkanas, P. & Goldberg, P. Phosphatic features, in *Interpretation of Micromorphological Features of Soils and Regoliths* (eds. Stoops, G., Marcelino, V. & Mees, F.) 323–346 (Elsevier, 2nd ed, 2018).

45. Angelucci, D. E. The recognition and description of knapped lithic artifacts in thin section. *Geoarchaeology* **25**, 220–232 (2010).

46. Angelucci, D. E. Lithic artefacts in *Archaeological Soil and Sediment Micromorphology* (Nicosia, C. & Stoops, G.) 223–230 (John Wiley & Sons, 2017).

47. Macphail, R. I., & Goldberg, P. Archaeological materials in *Interpretation of Micromorphological Features of Soils and Regolith* (eds. Stoops, G., Marcelino, V. & Mees, F.) 779–819 (Elsevier, 2nd ed, 2018).

48. Souron, A. PhD dissertation (Université de Poitiers, 2012.

49. Suwa, G., Souron, A. & Asfaw, B. Fossil Suidae of the Konso Formation in *Konso-Gardula Research Project, Vol. 1: Paleontological Collections: Background and Fossil Aves, Cercopithecidae, and Suidae* (Suwa, G., Beyene, Y. & Asfaw, B.) 73-88 (The University Museum, The University of Tokyo, Bulletin N. 47**,** 2014).

50. Geraads, D., Alemseged, Z., Reed, D., Wynn, J. & Roman, D. C. The Pleistocene fauna (other than Primates) from Asbole, lower Awash Valley, Ethiopia, and its environmental and biochronological implications. *Geobios* **37**, 697-718 (2004).

51. Gilbert, H. 11 – Suidae in Homo erectus - *Pleistocene Evidence from the Middle Awash, Ethiopia* (Gilbert, H. & Asfaw, B.) 231-260 (University of California Press, 2008).

52. Ameur, R. PhD Dissertation (Université d'Oran, 1988).

53. Geraads, D. Biogeography of Miocene-Pliocene circum-mediterranean rodents: a revision based upon factor analysis and parsimony analysis of endemicity. *Palaeogeogr. Palaeocl. Palaeoecol.* **137**, 273-288 (1998).

54. Geraads, D. Biogeographic relationships of Pliocene and Pleistocene North-western African Mammals. *Quat. Int.* **212**, 159-168 (2010).

55. Chaid-Saoudi, Y., Geraads, D. & Raynal, J.-P. The fauna and associated artefacts from the Lower Pleistocene site of Mansourah (Constantine, Algeria). *C. R. Palevol* **5**, 963-971 (2006).

56. Sahnouni, M. *et al*. Further research at the Oldowan site of Aïn Hanech, north-eastern Algeria. *J. Hum. Evol*. **43**, 925–937 (2002).

57. Geraads, D. *et al*. The Pleistocene Hominid site of Ternifine, Algeria: new results on the environment, age and human industries. *Quat. Res*. **25**, 380-386 (1986).

58. Geraads, D. Pleistocene Carnivora (Mammalia) from Tighennif (Ternifine), Algeria. *Geobios* **49**, 445–458 (2016).

59. Pickford, M. The fossil Suidae (Mammalia, Artiodactyla) from Ternifine (Tighenif) Algeria. *Münchner Geowissenschafltiche Abhandlungen* **50**, 1–67 (2020).

60. Gilbert, W. H., Asfaw, B. Homo erectus - *Pleistocene evidence from the Middle Awash* (University of California Press, 2009).

61. Geraads, D. Bovidae et Giraffidae (Artiodactyla, Mammalia) du Pléistocène de Ternifine (Algérie). *B. Mus. Natl. His. Nat.* **4^{ème}sér. C 3**, 47–86 (1981).

62. Domínguez-Rodrigo, M. & Baquedano, E. Distinguishing Butchery Cut Marks from Crocodile Bite Marks through Machine Learning Methods. *Sci. Rep.* **8**, 1–8 (2018).

63. Njau, J. K. & Blumenschine, R. J. A Diagnosis of Crocodile Feeding Traces on Larger Mammal Bone, with Fossil Examples from the Plio-Pleistocene Olduvai Basin, Tanzania, *J. Hum. Evol.* **50**, 142–62 (2006).

64. Njau, J. K. & Blumenschine, R. J. Crocodylian and Mammalian Carnivore Feeding Traces on Hominid Fossils from FLK 22 and FLK NN 3, Plio-Pleistocene, Olduvai Gorge, Tanzania. *J. Hum. Evol.* **63**, 408–17 (2012).

65. Sahle, Y., El Zaatari, S. & White, T. D. Hominid Butchers and Biting Crocodiles in the African Plio–Pleistocene. *Proc Natl Acad Sci* **114**, 13164–13169 (2017).

66. Yang, S. Y., Li, C. X., Yang, D. Y. & Li, X. S. Chemical weathering of the loess deposits in the lower Changjiang Valley, China, and paleoclimatic implications. *Quat. Int.* **117**, 27-34 (2004).

67. Profe, J., Zolitschka, B., Schirmer, W., Frechen, M. & Ohlendorf, C. Geochemistry unravels MIS 3/2 paleoenvironmental dynamics at the loess–paleosol sequence Schwalbenberg II, Germany. *Palaeogeogr. Palaeoclimatol. Palaeoecol.* **459**, 537-551 (2016).

68. Hosek, J. *et al.* An integrated rock-magnetic and geochemical approach to loess/paleosol sequences from Bohemia and Moravia (Czech Republic): Implications for the Upper Pleistocene paleoenvironment in central Europe. *Palaeogeogr. Palaeocl. Palaeoecol.* **418**, 344-358 (2015).

69. Rodrigo-Gamiz, M., Martínez-Ruiz, F., Rodríguez-Tovar, F. J., Jiménez-Espejo, F.J. & Pardo-Igúzquiza, E. Millennial-to centennial-scale climate periodicities and forcing mechanisms in the westernmost Mediterranean for the past 20,000 yr. *Quat. Res.* **81**, 78-93 (2014).

70. Martinez-Ruiz, F. *et al.* Paleoclimate and paleoceanography over the past 20,000 yr in the Mediterranean Sea Basins as indicated by sediment elemental proxies. *Quat. Sci. Rev.* **107**, 25-46 (2015).

71. Scarciglia, F., Pelle, T., Pulice, I. & Robustelli, G. A comparison of Quaternary soil chronosequences from the Ionian and Tyrrhenian coasts of Calabria, southern Italy: Rates of soil development and geomorphic dynamics. *Quat. Int.* **376**, 146-162 (2015).

72. Scarciglia, F. *et al.*, Weathering profiles in granitoid rocks of the Sila Massif uplands, Calabria, southern Italy: New insights into their formation processes and rates. *Sedim. Geol.* **336**, 46-67 (2016).

73. Krauss, L. *et al.* A Multi-Proxy Analysis of two Loess-Paleosol Sequences in the Northern Harz Foreland, Germany. *Palaeogeogr. Palaeocl.* **461**, 401-417 (2016).

74. Buggle, B., Glaser, B., Hambach, U., Gerasimenko, N. & Markovic, S. An evaluation of geochemical weathering indices in loess-paleosol studies. *Quat. Int.* **240**, 12-21 (2011).

75. Degeai, J.-P. *et al.*, Chemical weathering of palaeosols from the Lower Palaeolithic site of Valle Giumentina, central Italy. *Quat. Sci. Rev.* **183**, 88-109 (2018).

76. Degeai, J.-P. *et al.* Fluvial response to the last Holocene rapid climate change in the Northwestern Mediterranean coastlands. *Glob. Planet. Chang.* **152**, 176-186 (2017).

77. Degeai, J.-P. *et al.* A new interpolation method to measure delta evolution and sediment flux: Application to the late Holocene coastal plain of the Argens River in the western Mediterranean. *Mar. Geol.* **424**, 106159 (2020).

78. Trauth, M. H., Larrasoaña, J. C. & Mudelsee, M. Trends, rhythms and events in Plio-Pleistocene African climate. *Quat. Sci. Rev.* **28**, 399-411 (2009).

79. Skonieczny, C. *et al.* Monsoon-driven Saharan dust variability over the past 240,000 years. *Sci. Adv.* **5**, eaav1887 (2019).

80. Tiedemann, R., Sarnthein, M., Shackleton, N. J. Astronomic timescale for the Pliocene Atlantic $\delta^{18}O$ and dust flux records of Ocean Drilling Program Site 659. *Paleoceanography* **9**, 619-638 (1994).

81. Larrasoana, J. C., Roberts, A. P., Rohling, E. J., Winklhofer, M. & Wehausen, R. Three million years of monsoon variability over the northern Sahara. *Clim. Dyn.* **21**, 689-698 (2003).

82. Grant, K. M. *et al.*, A 3 million year index for North African humidity/aridity and the implication of potential pan-African Humid periods. *Quat. Sci. Rev.* **171**, 100-118 (2017).

83. Lisiecki, L. E. & Raymo, M. E. A Pliocene-Pleistocene stack of 57 globally distributed benthic D18O records. *Paleoceanography* **20**, PA1003 (2005) doi:10.1029/2004PA001071.

84. Channell, J. E. T., Singer, B. S., Jicha, B. R. Timing of Quaternary geomagnetic reversals and excursions in volcanic and sedimentary archives. *Quat. Sci. Rev.* **228**, 106114 (2020).

85. Rohling, E. J. *et al.* Sea-level and deep-sea-temperature variability over the past 5.3 million years. *Nature* **508**, 477-482 (2014).

86. Amies, J. D. PhD Dissertation (The Australian National University, Research School of Earth Sciences, 2018).

87. De Boer, B., van de Wal, R. S. W., Bintanja, R., Lourens, L. J. & Tuenter, E. Cenozoic global ice-volume and temperature simulations with 1-D ice-sheet models forced by benthic $\delta^{18}O$ records. *Ann. Glaciol.* **51**, 23-33 (2010).

88. Shackleton, N. J. New data on the evolution of Pliocene climate variability in *Palaeoclimate and evolution, with emphasis on human origins* (eds. Denton, G. H., Vrba, E., Partridge, T. C. & Burckle, L. H.) 242-248 (Yale University Press, 1995).

89. Fairbanks, R. G. A 17,000-year glacio-eustatic sea level record: influence of glacial melting rates on the Younger Dryas event and deep-ocean circulation, *Nature* **342**, 637-642 (1989).

90. El Kadiri, K. *et al.* Eustatic and tectonic controls on Quaternary Ras Leona marine terraces (Strait of Gibraltar, northern Morocco). *Quat. Res.* **74**, 277-288 (2010).

91. Emberger, L. *Les arbres du Maroc et comment les reconnaître* (Larose, 1938).

92. Benabid, A. *Flore et écosystemes du Maroc, evaluation et préservation de la biodiversité* (Ibis Press, 2000).

93. Reille, M. *Pollen et spores d'Europe et d'Afrique du Nord* (Laboratoire de Botanique historique et de Palynologie, 1992).

94. M. Reille, *Pollen et spores d'Europe et d'Afrique du Nord*, *supplément 1* (Laboratoire de Botanique historique et de Palynologie, 1995).

95. Reille, M. *Pollen et spores d'Europe et d'Afrique du Nord*, *supplément 2* (Laboratoire de Botanique historique et de Palynologie, 1998).

96. Cugny, C., Mazier, F., Galop, D. Modern and fossil non-pollen palynomorphs from the Basque mountains (western Pyrenees, France): the use of coprophilous fungi to reconstruct pastoral activity. *Hist. Archaeobot.* **19**, 391-408 (2010).

97. Cronquist, A. *An integrated system of classification of flowering plants* (Columbia University Press, 1981).
